# *Carnobacterium inhibens* isolated in blood culture of an immunocompromised, metastatic cancer patient: a case report and literature review

**DOI:** 10.1186/s12879-021-06095-7

**Published:** 2021-05-01

**Authors:** Carson Ka-Lok Lo, Prameet M. Sheth

**Affiliations:** 1 Division of Infectious Diseases, Juravinski Cancer Centre, McMaster University Infectious Diseases Residency Program, 699 Concession Street, Hamilton, Ontario L8V 5C2 Canada; 2 Department of Pathology and Molecular Medicine, Queen’s University, Kingston, Ontario Canada; 3 Division of Microbiology, Kingston Health Sciences Centre, 76 Stuart Street, Kingston, Ontario K7L 2V7 Canada

**Keywords:** Bacteremia, *Carnobacterium inhibens*, *Carnobacterium* species, Case report, Immunocompromised, Sepsis

## Abstract

**Background:**

*Carnobacterium* species are lactic acid-producing Gram-positive bacteria that have been approved by the US Food and Drug Administration and Health Canada for use as a food bio-preservative. The use of live bacteria as a food additive and its potential risk of infections in immunocompromised patients are not well understood.

**Case presentation:**

An 81-year-old male with a history of metastatic prostate cancer on androgen deprivation therapy and chronic steroids presented to our hospital with a 2-week history of productive cough, dyspnea, altered mentation, and fever. Extensive computed tomography imaging revealed multifocal pneumonia without other foci of infection. He was diagnosed with pneumonia and empirically treated with ceftriaxone and vancomycin. Blood cultures from admission later returned positive for *Carnobacterium inhibens*. He achieved clinical recovery with step-down to oral amoxicillin/clavulanic acid for a total 7-day course of antibiotics.

**Conclusions:**

This is the fourth reported case of bacteremia with *Carnobacterium* spp. isolated from humans. This case highlights the need to better understand the pathogenicity and disease spectrum of bacteria used in the food industry for bio-preservation, especially in immunocompromised patients.

**Supplementary Information:**

The online version contains supplementary material available at 10.1186/s12879-021-06095-7.

## Background

*Carnobacterium* species are lactic acid-producing, Gram-positive rod-shaped bacteria that are rarely isolated in humans and often regarded as non-pathogenic [1]. Instead, they are frequently isolated from the environment and are currently approved for use as a bio-preservative in the food industry [1]. The use of live bacteria as food additives poses a potential risk for immunocompromised patients, including several studies highlighting cases of bacteremia/sepsis associated with lactic acid bacteria used in probiotics (e.g., *Lactobacillus* spp.) [2–5].

We report a case of *Carnobacterium inhibens* isolated in blood culture of an immunocompromised cancer patient with pneumonia. We also reviewed published reports on human infections with *Carnobacterium* spp.

## Case presentation

An 81-year-old male presented to the Emergency Department with a 2-week history of productive cough, exertional dyspnea, general malaise, altered mental status, and subjective fevers and chills. He had no recent sick contact exposures or travel history. He had no (farm) animal exposures and no history of the handling of, or heavy consumption of fish, dairy or meat products. His past medical history included castrate-resistant prostate cancer with liver and bone metastases on enzalutamide, leuprolide, and oral prednisone (10 mg daily) for at least 1 year. He also had triple-bypass cardiac surgery for a previous myocardial infarction. He had a previous history of smoking but no alcohol or recreational drug use.

On examination, his body temperature was 37.5 °C, with sinus tachycardia at 135 beats per minute, and blood pressure of 95/62 mmHg which improved to 121/79 mmHg with intravenous fluid resuscitation. He had a resting pulse oximeter saturation (SpO_2_) nadir of 93% on room air, but continued to require supplemental oxygen by nasal cannula due to intermittent episodes of desaturations. Lung auscultation demonstrated decreased air entry to the bases with diffuse crackles bilaterally. No murmurs on cardiac auscultation or other stigmata of endocarditis.

Sepsis workup showed peripheral white blood cell count of 10.7 × 10^9^/L with neutrophil count of 9.49 × 10^9^/L and elevated inflammatory markers (erythrocyte sedimentation rate 90 mm/hr., C-reactive protein 195.9 mg/L). Blood cultures were obtained prior to antibiotic administration on admission. Chest radiograph followed by a full-body computerized tomography revealed multifocal pneumonia without other foci of infection. Transthoracic echocardiography did not show any vegetations or hemodynamically significant valvular dysfunctions. A bone scintigraphy revealed prior known bony metastases without other foci of infection.

**Fig. 1:**
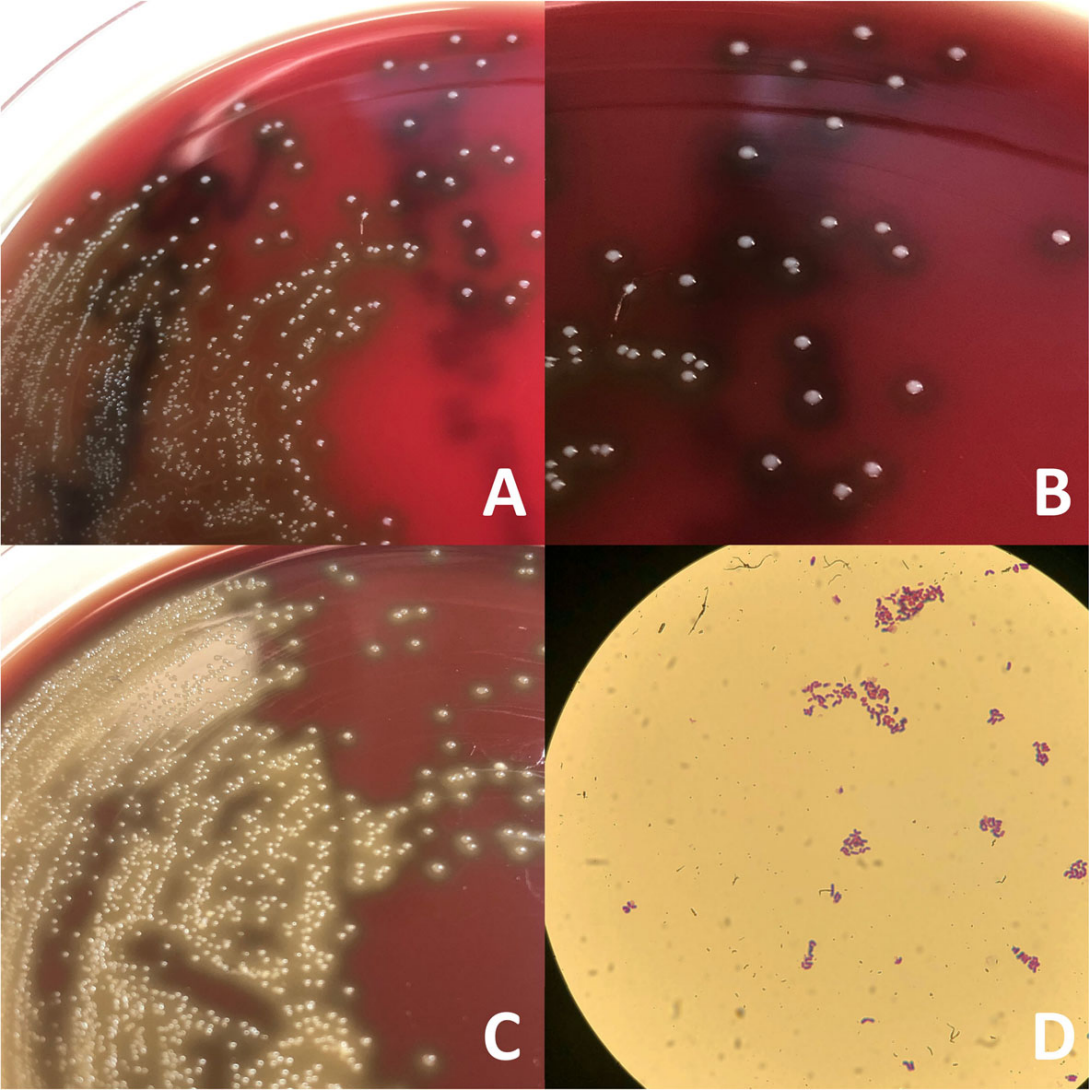
*Carnobacterium inhibens* isolated after incubation at 37 °C for 18 h. **a**, **b** 1–2 mm diameter, grey-colored, round, alpha-hemolytic colonies on 5% sheep blood agar. **c** Grey-colored, flat colonies surrounded by greenish discoloration around the colonies on chocolate agar. **d** Gram staining of blood culture isolate of *C. inhibens* depicting Gram-positive, asporogenous, lactobacillus-like rods, 100x

Given concerns for clinical deterioration and sepsis, he was admitted to hospital and started on empiric antibiotics of ceftriaxone and vancomycin for pneumonia, potentially secondary to aspiration. Two of 4 blood culture bottles (i.e., both aerobic and anaerobic bottles) flagged positive at 18 h with a Gram-positive bacillus that failed to identify by the VITEK-MS MALDI-TOF (Matrix-Assisted Laser Desorption Time-Of-Flight Mass Spectrometry, bioMérieux Clinical Diagnostics, Canada). The isolate was forwarded to the provincial reference laboratory (Public Health Ontario Laboratory, PHOL) for further testing and identification. The isolate was identified by PHOL to be *C. inhibens* with homology of 99% using 16S rRNA gene sequence analysis (Fig. 1a-d), though this information was only available after the patient was discharged home.

The patient clinically improved with the empiric antibiotic regimen and was stepped down to oral amoxicillin/clavulanic acid for a total 7-day course of antibiotics. He achieved complete clinical recovery upon finishing antibiotics and did not require any supplemental oxygen at the time of discharge. Repeat blood cultures obtained both while on antibiotics and after discharge from hospital were negative.

## Discussion and conclusions

*Carnobacterium* spp. belong to the order of lactic acid bacteria known as Lactobacillales, which includes genera such as *Lactobacillus* often seen in probiotic use [6]. *Carnobacterium* spp. are non-spore-forming, lactic acid-producing, Gram-positive rod-shaped bacteria [7, 8]. Most species can be found in both polar and temperate environments due to their cryophilic and cryotolerant properties; they can tolerate, grow, and reproduce at low temperatures (i.e., − 20 to + 10 °C) [1, 7, 8]. They are also known to tolerate high-pressure environments, such as the vacuum-packing process in food preservation [9–11].

The use of live lactic acid-producing bacteria such as *Carnobacterium* spp. in food and bio-preservation continues to be a growing area of research in the meat, dairy and seafood industry. Bacteriocins produced by these bacteria have antimicrobial properties that limit or inhibit the growth of foodborne pathogens [1]. In particular, bacteriocins produced by the species *C. divergens* and *C. maltaromaticum* are shown to inhibit the growth of *Listeria monocytogenes* in various food products [1, 12]. The additional unique properties of *Carnobacterium* spp. to survive under high-pressure vacuum-packing and grow at refrigeration temperatures make them the ideal candidate as an additive to prevent food spoilage, especially in the meat and seafood industry [1]. Both *C. divergens* and *C. maltaromaticum* are currently approved by Health Canada as food additives for bio-preservation of ready-to-eat smoked fish and vacuum-packed meat and poultry, respectively [13, 14]. *Carnobacterium* spp. are also used in the dairy industry and have been shown to reduce the growth of both *L. monocytogenes* and *Pseudomonas* spp. in soft unpasteurized cheeses, improving the safety and shelf-life of selected dairy products [15, 16].

*Carnobacterium* spp. are often considered non-pathogenic to humans [1]. Although the use of live lactic acid bacteria (e.g., *Lactobacillus* spp.) in probiotics has been approved by organizations such as the US Food and Drug Administration, current studies on its safety outcomes in immunocompromised populations remain limited [4]. Despite historical evidence of its safe use, recent studies have reported cases of infections associated with lactic acid bacteria used in probiotics [2–5]. For example, *Lactobacillus* spp. have been identified as the causal pathogen in several case reports, ranging from local (e.g., pneumonia, abscesses) to systemic infections (e.g., infective endocarditis, bacteremia and/or sepsis) [2–5]. To date, there are few studies on infections associated with *Carnobacterium* spp. in humans.

We performed a comprehensive search of all English-written articles published on human infections with *Carnobacterium* spp. isolated from any body site or culture. We searched for articles from inception to December 2020 using databases including OvidMEDLINE, EMBASE (Additional files 1 and 2 for search strategy), and Google Scholar. To date, only 5 cases of *Carnobacterium* spp. isolated from humans have been reported (Table 1). Two cases had *Carnobacterium* spp. identified amongst mixed flora containing other aerobic and anaerobic bacteria from traumatic wounds, in the setting of water exposure [17, 18]. Three cases were isolated from blood cultures; one was reported as suspected gastrointestinal source of infection in an immunocompetent man with 1 positive blood culture set who presented with fever and an extensive history of handling and consuming fish [19]. The remaining 2 cases were likely suspected gastrointestinal source or central line-associated bloodstream infection: a woman with diabetes and chronic alcohol use requiring parenteral nutrition post-esophagectomy for necrotizing esophagitis complicated by post-operative cardiac arrest and septic shock, with multiple positive blood cultures for *C. divergens* [20], and; a man receiving chemotherapy and parenteral nutrition presenting with febrile neutropenia and extensive oral mucositis with 1 positive blood culture set for *C. divergens* [21].

**Table 1:** Literature review of human infections with *Carnobacterium* spp. isolated in cultures

Case	Age/Sex	Country	Possible risk factors	Type of culture/body Site	Isolate	Presentation	Treatment	Outcome	Reference
1	35/M	Czech Republic	None	Wound swab of abscess	Mixed flora with *C. piscicola*	Traumatic hand from water sawmill	Amputation, debridement; Abx	Cured	[17]
2	13/F	China	None	Wound swab of gangrene	Mixed flora with *Carnobacterium* spp.	Traumatic hand with pool water exposure	Amputation, debridement; IPM	Cured	[18]
3	43/M	Austria	Extensive hx of handling and consuming fish	½ blood culture sets	*C. mobile* or *C. funditum*	Sepsis suspected from GI source	CRO/AMP ➔ MXF	Cured	[19]
4	57/F	France	DM; EtOH; TPN; post-cardiac arrest	4 blood culture sets	*C. divergens*	Septic shock post-cardiac arrest; esophagectomy for necrotizing esophagitis on TPN/EN	Broad spectrum Abx ➔ AMX	Cured	[20]
5	65/M	South Korea	Cancer; neutropenia on etoposide	½ blood culture sets	*C. divergens*	Febrile neutropenia with oral mucositis on TPN	TZP/VAN	Cured	[21]
6	81/M	Canada	Cancer; chronic steroid use	½ blood culture sets	*C. inhibens*	Sepsis with multifocal pna	CRO/VAN ➔ AMC	Cured	Our case

Age (years old) and sex (F, female; M, male). *Abbreviations*: *Abx* antibiotics (not specified), *AMC* amoxicillin/clavulanic acid, *AMP* ampicillin, *AMX* amoxicillin, *CRO* ceftriaxone, *DM* diabetes mellitus, *EN* enteral nutrition, *EtOH* chronic alcohol use, *GI* gastrointestinal, *hx* history, *IPM* imipenem, *MXF* moxifloxacin, pna pneumonia, *TPN* total parenteral nutrition, *TZP* piperacillin-tazobactam, *VAN* vancomycin, *½* 1 of 2 positive culture sets

Our case described an immunocompromised cancer patient on chronic steroids presenting with multifocal pneumonia with *C. inhibens* isolated in 2 of 4 blood culture bottles. Given the lack of clinical experience with this pathogen and its ability to cause disease in humans, as well the fact that *C. inhibens* was only isolated in 1 of 2 blood culture sets (with negative repeat blood cultures on antibiotics), there remained uncertainty whether it was the causative pathogen for the patient’s pneumonia or a contaminant. Our case was unique as unlike other published cases, there was no clear exposure history, prior traumatic wounds, central venous catheter access (for parenteral nutrition), or excessive consumption of meat, dairy or seafood products. Extensive investigations by imaging confirmed pneumonia as the primary infection, without other foci of infection. Given the altered mentation, we suspect our patient aspirated giving rise to multifocal pneumonia, due to mixed aerobic and anaerobic bacteria from oral and/or gastric flora. We postulate the mixed flora likely included *C. inhibens*, which was later isolated in blood culture during transient bacteremia secondary from aspiration pneumonia. Although invasive procedures for culture (e.g., bronchoalveolar lavage) were not pursued to confirm our hypothesis as patient improved on empiric antibiotic therapy, we believe the pneumonia was likely polymicrobial as opposed to *C. inhibens* as sole pathogen responsible for causing infection.

To date, there are no recommended interpretative criteria or breakpoints established by the Clinical and Laboratory Standards Institute (CLSI) for the susceptibility testing of antimicrobial agents against *Carnobacterium* spp. causing human infections. Some of the cases reported the minimum inhibitory concentration (MIC) of their isolates, which seemed to suggest susceptibility to penicillins, carbapenems, macrolides, but resistance to cephalosporins [19, 20]. Certain *Carnobacterium* spp. isolates such as *C. piscicola* demonstrated intrinsic resistance to many antibiotics including fluoroquinolones, aminoglycosides, trimethoprim, though the mechanisms of resistance remain not well understood [22]. In vitro susceptibilities to antibiotic classes including penicillins also varied across different *Carnobacterium* spp. strains [22–24]. Drug susceptibility testing performed on a *C. inhibens* strain in a 2002 study appeared to show in vitro sensitivity to several antibiotics including, but not limited to, penicillins, tetracycline, and vancomycin [24]. No susceptibility testing was performed for our isolate; our patient responded well to empiric parenteral followed by oral step-down antibiotics for aspiration pneumonia, prior to confirmation of the *C. inhibens* isolate as it required identification at a reference laboratory. Future considerations of antibacterial susceptibility breakpoints can be revisited once a better understanding of the infections associated with *Carnobacterium* spp. has been established.

The pathogenicity and disease spectrum of *Carnobacterium* spp. in humans remain unknown. The use of Gram-positive bacteria in the food industry for their bio-preservative or fermentative capacity presents a potential source of unique organisms leading to disease, especially in immunocompromised patients.

## Supplementary Information

**Additional file 1.** Database: OVID Medline Epub Ahead of Print, In-Process & Other Non-Indexed Citations, Ovid MEDLINE(R) Daily and Ovid MEDLINE(R) 1946 to Present – Search Strategy. Compilation of search strategy, search key terms, and full list of journal article titles and abstracts from initial literature search of Ovid MEDLINE database (inception to December 2020); list was used for screening of relevant articles for subsequent literature review (Table 1).

**Additional file 2.** Database: Embase <1974 to 2020 December 29> – Search Strategy. Compilation of search strategy, search key terms, and full list of journal article titles and abstracts from initial literature search of EMBASE database (inception to December 2020); list was used for screening of relevant articles for subsequent literature review (Table 1).

## Data Availability

All data generated and/or analysed during this study are included in this published article [and its supplementary information files]. Please see Table 1 for data extracted from literature review and Additional file 1 and Additional file 2 for our literature search strategies from databases.

## Abbreviations

*C. divergens*: *Carnobacterium divergens*
*C. funditum*: *Carnobacterium funditum*
*C. inhibens*: *Carnobacterium inhibens*
*C. maltaromaticum*: *Carnobacterium maltaromaticum*
*C. mobile*: *Carnobacterium mobile*
*C. piscicola*: *Carnobacterium piscicola*
CLSI: Clinical and Laboratory Standards Institute
*L. monocytogenes*: *Listeria monocytogenes*
MALDI-TOF-MS: Matrix-Assisted Laser Desorption Time-Of-Flight Mass Spectrometry
MIC: Minimum inhibitory concentration
PHOL: Public Health Ontario Laboratory
16S rRNA gene sequence analysis: 16S ribosomal RNA gene sequence analysis
