# Supplementary material for: Carnobacterium inhibens isolated in blood culture of an immunocompromised, metastatic cancer patient: a case report and literature review
Source: BMC Infect Dis. 2021 May 1;21:403. doi: 10.1186/s12879-021-06095-7 (PMC8088058; doi:10.1186/s12879-021-06095-7)
Supplement: Supplementary file 1 — Additional file 1. Database: OVID Medline Epub Ahead of Print, In-Process & Other Non-Indexed Citations, Ovid MEDLINE(R) Daily and Ovid MEDLINE(R) 1946 to Present – Search Strategy. Compilation of search strategy, search key terms, and full list of journal article titles and abstracts from initial literature search of Ovid MEDLINE database (inception to December 2020); list was used for screening of relevant articles for subsequent literature review (Table 1). [file 12879_2021_6095_MOESM1_ESM.doc]

Database: OVID Medline Epub Ahead of Print, In-Process & Other Non-Indexed Citations, Ovid MEDLINE(R) Daily and Ovid MEDLINE(R) 1946 to Present

Search Strategy:

--------------------------------------------------------------------------------

1 carnobacterium.mp. or exp Carnobacterium/ (557)

2 human.mp. or exp Humans/ (19575672)

3 1 and 2 (66)

***************************

1.

Antibiotic Resistance Genes and Bacterial Communities of Farmed Rainbow Trout Fillets (Oncorhynchus mykiss).

Helsens N; Calvez S; Prevost H; Bouju-Albert A; Maillet A; Rossero A; Hurtaud-Pessel D; Zagorec M; Magras C.

Frontiers in Microbiology. 11:590902, 2020.

[Journal Article]

UI: 33343530

The rise of antibiotic resistance is not only a challenge for human and animal health treatments, but is also posing the risk of spreading among bacterial populations in foodstuffs. Farmed fish-related foodstuffs, the food of animal origin most consumed worldwide, are suspected to be a reservoir of antibiotic resistance genes and resistant bacterial hazards. However, scant research has been devoted to the possible sources of diversity in fresh fillet bacterial ecosystems (farm environment including rivers and practices, and factory environment). In this study bacterial communities and the antibiotic resistance genes of fresh rainbow trout fillet were described using amplicon sequencing of the V3-V4 region of the 16S rRNA gene and high-throughput qPCR assay. The antibiotic residues were quantified using liquid chromatography/mass spectrometry methods. A total of 56 fillets (composed of muscle and skin tissue) from fish raised on two farms on the same river were collected and processed under either factory or laboratory sterile filleting conditions. We observed a core-bacterial community profile on the fresh rainbow trout fillets, but the processing conditions of the fillets has a great influence on their mean bacterial load (3.38 +/- 1.01 log CFU/g vs 2.29 +/- 0.72 log CFU/g) and on the inter-individual diversity of the bacterial community. The bacterial communities were dominated by Gamma- and Alpha-proteobacteria, Bacteroidetes, Firmicutes, and Actinobacteria. The most prevalent genera were Pseudomonas, Escherichia-Shigella, Chryseobacterium, and Carnobacterium. Of the 73 antibiotic residues searched, only oxytetracycline residues were detected in 13/56 fillets, all below the European Union maximum residue limit (6.40-40.20 mug/kg). Of the 248 antibiotic resistance genes searched, 11 were found to be present in at least 20% of the fish population (tetracycline resistance genes tetM and tetV, beta-lactam resistance genes bla DHA and bla ACC, macrolide resistance gene mphA, vancomycin resistance genes vanTG and vanWG and multidrug-resistance genes mdtE, mexF, vgaB and msrA) at relatively low abundances calculated proportionally to the 16S rRNA gene.

Copyright © 2020 Helsens, Calvez, Prevost, Bouju-Albert, Maillet, Rossero, Hurtaud-Pessel, Zagorec and Magras.

Version ID

1

Record Owner

From MEDLINE, a database of the U.S. National Library of Medicine.

Status

PubMed-not-MEDLINE

Authors Full Name

Helsens, Nicolas; Calvez, Segolene; Prevost, Herve; Bouju-Albert, Agnes; Maillet, Aurelien; Rossero, Albert; Hurtaud-Pessel, Dominique; Zagorec, Monique; Magras, Catherine.

Institution

Helsens, Nicolas. INRAE, Oniris, SECALIM, Nantes, France. Helsens, Nicolas. INRAE, Oniris, BIOEPAR, Nantes, France.

Calvez, Segolene. INRAE, Oniris, BIOEPAR, Nantes, France.

Prevost, Herve. INRAE, Oniris, SECALIM, Nantes, France.

Bouju-Albert, Agnes. INRAE, Oniris, SECALIM, Nantes, France.

Maillet, Aurelien. INRAE, Oniris, SECALIM, Nantes, France.

Rossero, Albert. INRAE, Oniris, SECALIM, Nantes, France.

Hurtaud-Pessel, Dominique. ANSES Laboratoire de Fougeres, Unite Analyse des Residus et Contaminants, Fougeres, France.

Zagorec, Monique. INRAE, Oniris, SECALIM, Nantes, France.

Magras, Catherine. INRAE, Oniris, SECALIM, Nantes, France.

Keyword Heading

antibiotic residues antibiotic resistance genes

bacterial communities

factory processing

fish fillet

raceway.

Year of Publication

2020

Link to the Ovid Full Text or citation:

[Click here for full text options](https://libaccess.mcmaster.ca/login?url=http://ovidsp.ovid.com/ovidweb.cgi?T=JS&CSC=Y&NEWS=N&PAGE=fulltext&D=prem&AN=33343530)

Link to the External Link Resolver:

[SFX](http://sfx.scholarsportal.info/mcmaster?sid=OVID:medline&id=pmid:33343530&id=doi:10.3389%2Ffmicb.2020.590902&issn=1664-302X&isbn=&volume=11&issue=&spage=590902&pages=590902&date=2020&title=Frontiers+in+Microbiology&atitle=Antibiotic+Resistance+Genes+and+Bacterial+Communities+of+Farmed+Rainbow+Trout+Fillets+(Oncorhynchus+mykiss).&aulast=Helsens&pid=<author>Helsens+N%3BCalvez+S%3BPrevost+H%3BBouju-Albert+A%3BMaillet+A%3BRossero+A%3BHurtaud-Pessel+D%3BZagorec+M%3BMagras+C<%2Fauthor><AN>33343530<%2FAN><DT>Journal+Article<%2FDT>)

2.

Changes in the Bacterial Diversity of Human Milk during Late Lactation Period (Weeks 21 to 48).

Marin-Gomez W; Grande MJ; Perez-Pulido R; Galvez A; Lucas R.

Foods. 9(9), 2020 Aug 27.

[Journal Article]

UI: 32867028

Breast milk from a single mother was collected during a 28-week lactation period. Bacterial diversity was studied by amplicon sequencing analysis of the V3-V4 variable region of the 16S rRNA gene. Firmicutes and Proteobacteria were the main phyla detected in the milk samples, followed by Actinobacteria and Bacteroidetes. The proportion of Firmicutes to Proteobacteria changed considerably depending on the sampling week. A total of 411 genera or higher taxons were detected in the set of samples. Genus Streptococcus was detected during the 28-week sampling period, at relative abundances between 2.0% and 68.8%, and it was the most abundant group in 14 of the samples. Carnobacterium and Lactobacillus had low relative abundances. At the genus level, bacterial diversity changed considerably at certain weeks within the studied period. The weeks or periods with lowest relative abundance of Streptococcus had more diverse bacterial compositions including genera belonging to Proteobacteria that were poorly represented in the rest of the samples.

Version ID

1

Record Owner

From MEDLINE, a database of the U.S. National Library of Medicine.

Status

PubMed-not-MEDLINE

Author NameID

Marin-Gomez, Wendy; ORCID: <https://orcid.org/0000-0001-5294-0326> Galvez, Antonio; ORCID: <https://orcid.org/0000-0002-5894-5029>

Lucas, Rosario; ORCID: <https://orcid.org/0000-0002-0180-9296>

Authors Full Name

Marin-Gomez, Wendy; Grande, M Jose; Perez-Pulido, Ruben; Galvez, Antonio; Lucas, Rosario.

Institution

Marin-Gomez, Wendy. Microbiology Division, Department of Health Sciences, Faculty of Experimental Sciences, University of Jaen, 23071 Jaen, Spain. Grande, M Jose. Microbiology Division, Department of Health Sciences, Faculty of Experimental Sciences, University of Jaen, 23071 Jaen, Spain.

Perez-Pulido, Ruben. Microbiology Division, Department of Health Sciences, Faculty of Experimental Sciences, University of Jaen, 23071 Jaen, Spain.

Galvez, Antonio. Microbiology Division, Department of Health Sciences, Faculty of Experimental Sciences, University of Jaen, 23071 Jaen, Spain.

Lucas, Rosario. Microbiology Division, Department of Health Sciences, Faculty of Experimental Sciences, University of Jaen, 23071 Jaen, Spain.

Keyword Heading

biodiversity breast milk

lactic acid bacteria

late lactation

metagenomics.

Year of Publication

2020

Link to the Ovid Full Text or citation:

[Click here for full text options](https://libaccess.mcmaster.ca/login?url=http://ovidsp.ovid.com/ovidweb.cgi?T=JS&CSC=Y&NEWS=N&PAGE=fulltext&D=prem6&AN=32867028)

Link to the External Link Resolver:

[SFX](http://sfx.scholarsportal.info/mcmaster?sid=OVID:medline&id=pmid:32867028&id=doi:10.3390%2Ffoods9091184&issn=2304-8158&isbn=&volume=9&issue=9&spage=&pages=&date=2020&title=Foods&atitle=Changes+in+the+Bacterial+Diversity+of+Human+Milk+during+Late+Lactation+Period+(Weeks+21+to+48).&aulast=Marin-Gomez&pid=<author>Marin-Gomez+W%3BGrande+MJ%3BPerez-Pulido+R%3BGalvez+A%3BLucas+R<%2Fauthor><AN>32867028<%2FAN><DT>Journal+Article<%2FDT>)

3.

Lactic Acid Bacteria in Finfish-An Update. [Review]

Ringo E; Hoseinifar SH; Ghosh K; Doan HV; Beck BR; Song SK.

Frontiers in Microbiology. 9:1818, 2018.

[Journal Article. Review]

UI: 30147679

A complex and dynamic community of microorganisms, play important roles within the fish gastrointestinal (GI) tract. Of the bacteria colonizing the GI tract, are lactic acid bacteria (LAB) generally considered as favorable microorganism due to their abilities to stimulating host GI development, digestive function, mucosal tolerance, stimulating immune response, and improved disease resistance. In early finfish studies, were culture-dependent methods used to enumerate bacterial population levels within the GI tract. However, due to limitations by using culture methods, culture-independent techniques have been used during the last decade. These investigations have revealed the presence of Lactobacillus, Lactococcus, Leuconostoc, Enterococcus, Streptococcus, Carnobacterium, Weissella, and Pediococcus as indigenous species. Numerous strains of LAB isolated from finfish are able to produce antibacterial substances toward different potential fish pathogenic bacteria as well as human pathogens. LAB are revealed be the most promising bacterial genera as probiotic in aquaculture. During the decade numerous investigations are performed on evaluation of probiotic properties of different genus and species of LAB. Except limited contradictory reports, most of administered strains displayed beneficial effects on both, growth-and reproductive performance, immune responses and disease resistance of finfish. This eventually led to industrial scale up and introduction LAB-based commercial probiotics. Pathogenic LAB belonging to the genera Streptococcus, Enterococcus, Lactobacillus, Carnobacterium, and Lactococcus have been detected from ascites, kidney, liver, heart, and spleen of several finfish species. These pathogenic bacteria will be addressed in present review which includes their impacts on finfish aquaculture, possible routes for treatment. Finfish share many common structures and functions of the immune system with warm-blooded animals, although apparent differences exist. This similarity in the immune system may result in many shared LAB effects between finfish and land animals. LAB-fed fish show an increase in innate immune activities leading to disease resistances: neutrophil activity, lysozyme secretion, phagocytosis, and production of pro-inflammatory cytokines (IL-1beta, IL-6, IL-8, and TNF-alpha). However, some LAB strains preferentially induces IL-10 instead, a potent anti-inflammatory cytokine. These results indicate that LAB may vary in their immunological effects depending on the species and hosts. So far, the immunological studies using LAB have been focused on their effects on innate immunity. However, these studies need to be further extended by investigating their involvement in the modulation of adaptive immunity. The present review paper focuses on recent findings in the field of isolation and detection of LAB, their administration as probiotic in aquaculture and their interaction with fish immune responses. Furthermore, the mode of action of probiotics on finfish are discussed.

Version ID

1

Record Owner

From MEDLINE, a database of the U.S. National Library of Medicine.

Status

PubMed-not-MEDLINE

Authors Full Name

Ringo, Einar; Hoseinifar, Seyed Hossein; Ghosh, Koushik; Doan, Hien Van; Beck, Bo Ram; Song, Seong Kyu.

Institution

Ringo, Einar. Faculty of Bioscience, Fisheries and Economics, Norwegian College of Fishery Science, UiT The Arctic University of Norway, Tromso, Norway. Hoseinifar, Seyed Hossein. Department of Fisheries, Faculty of Fisheries and Environmental Sciences, Gorgan University of Agricultural Sciences and Natural Resources, Gorgan, Iran.

Ghosh, Koushik. Aquaculture Laboratory, Department of Zoology, The University of Burdwan, Bardhaman, India.

Doan, Hien Van. Department of Animal and Aquatic Sciences, Faculty of Agriculture, Chiang Mai University, Chiang Mai, Thailand.

Beck, Bo Ram. School of Life Science, Handong University, Pohang, South Korea.

Song, Seong Kyu. School of Life Science, Handong University, Pohang, South Korea.

Keyword Heading

aquaculture finfish

fish immunity

lactic acid bacteria (LAB)

probiotic bacteria

probiotics.

Year of Publication

2018

Link to the Ovid Full Text or citation:

[Click here for full text options](https://libaccess.mcmaster.ca/login?url=http://ovidsp.ovid.com/ovidweb.cgi?T=JS&CSC=Y&NEWS=N&PAGE=fulltext&D=prem6&AN=30147679)

Link to the External Link Resolver:

[SFX](http://sfx.scholarsportal.info/mcmaster?sid=OVID:medline&id=pmid:30147679&id=doi:10.3389%2Ffmicb.2018.01818&issn=1664-302X&isbn=&volume=9&issue=&spage=1818&pages=1818&date=2018&title=Frontiers+in+Microbiology&atitle=Lactic+Acid+Bacteria+in+Finfish-An+Update.&aulast=Ringo&pid=<author>Ringo+E%3BHoseinifar+SH%3BGhosh+K%3BDoan+HV%3BBeck+BR%3BSong+SK<%2Fauthor><AN>30147679<%2FAN><DT>Journal+Article<%2FDT>)

4.

Bacteria as Potential Indicators of Heavy Metal Contamination in a Tropical Mangrove and the Implications on Environmental and Human Health.

De La Rosa-Acosta M; Jimenez-Collazo J; Maldonado-Roman M; Malave-Llamas K; Musa-Wasil JC.

Journal of Tropical Life Science. 5(3):100-116, 2015 Sep.

[Journal Article]

UI: 28835856

Heavy metal (HM) exposure has been associated with human health diseases like cancer, kidney and liver damage, neurological disorders, motor skills, low bone density and learning problems. With the beginning of the industrialization, the heavy metals in high concentration contribute to putting on the risk the humans in the vicinity. Our study site is located in Catano, Puerto Rico. This is a highly industrialized area. It is surrounded by a recreational park, a rum distillery, two thermoelectric factories, and was impacted by CAPECO (oil refinery) explosion in 2009. Las Cucharillas marsh is part of The San Juan Bay Estuary System, considered as a critical wildlife area. The mangrove marsh has three of the four mangrove species found in PR Laguncularia racemosa, Avicennia germinans and Rhizophora mangle. This study was aimed at seven different heavy metals: Arsenic (As), Cadmium (Cd), Chromium (Cr), Lead (Pb), Zinc (Zn), Mercury (Hg) and Copper (Cu). These metals at high concentrations are of human health concern due to their toxicity, persistence, bioaccumulative and bio magnification potentials. Contamination of surface sediments with HM affects the food chain, starting with marine organisms up to humans. The people who live near the contaminated area and the local fishermen are at high risk of exposure. Studies reveal that certain microorganisms can resist the toxicity of heavy metals even at high concentrations. Our study pretends to exploit the sensitive nature of some bacteria to HM and use them as bioindicators. The objective of this research is to assess the bacterial community on the mangrove marsh, identify these bacteria and correlate bacterial species with the type and concentration of the metals found on the site. Our preliminary results with the BIOLOG R identification were five bacteria that are: Carnobacterium inhibens, Cupriavidus gilardii, Enterococcus maloduratus, Microbacterium flavescens and Ralstonia pickettii. This study will continue with an assessment of the exposure of different concentrations of heavy metals to our identified bacteria and underlying the mechanisms of degradation, magnification and or bioconcentration of these heavy metals.

Version ID

1

Record Owner

From MEDLINE, a database of the U.S. National Library of Medicine.

Status

PubMed-not-MEDLINE

Authors Full Name

De La Rosa-Acosta, Melanie; Jimenez-Collazo, Johannys; Maldonado-Roman, Marixa; Malave-Llamas, Karlo; Musa-Wasil, Juan C.

Institution

De La Rosa-Acosta, Melanie. School of Science and Technology, URGREAT-MBRS-RISE, Universidad Del Este, Carolina, Puerto Rico. Jimenez-Collazo, Johannys. School of Science and Technology, Universidad Del Este, Carolina, Puerto Rico.

Maldonado-Roman, Marixa. School of Environmental Affairs, Universidad Metropolitana, San Juan, Puerto Rico.

Malave-Llamas, Karlo. School of Science and Technology, URGREAT-MBRS-RISE, Universidad Del Este, Carolina, Puerto Rico.

Musa-Wasil, Juan C. School of Environmental Affairs, Universidad Metropolitana, San Juan, Puerto Rico.

Keyword Heading

BIOLOG R Heavy metals

bioindicators

caribbean

tropical marsh.

Year of Publication

2015

Link to the Ovid Full Text or citation:

[Click here for full text options](https://libaccess.mcmaster.ca/login?url=http://ovidsp.ovid.com/ovidweb.cgi?T=JS&CSC=Y&NEWS=N&PAGE=fulltext&D=prem5&AN=28835856)

Link to the External Link Resolver:

[SFX](http://sfx.scholarsportal.info/mcmaster?sid=OVID:medline&id=pmid:28835856&id=doi:10.11594%2Fjtls.05.03.01&issn=2087-5517&isbn=&volume=5&issue=3&spage=100&pages=100-116&date=2015&title=Journal+of+Tropical+Life+Science&atitle=Bacteria+as+Potential+Indicators+of+Heavy+Metal+Contamination+in+a+Tropical+Mangrove+and+the+Implications+on+Environmental+and+Human+Health.&aulast=De+La+Rosa-Acosta&pid=<author>De+La+Rosa-Acosta+M%3BJimenez-Collazo+J%3BMaldonado-Roman+M%3BMalave-Llamas+K%3BMusa-Wasil+JC<%2Fauthor><AN>28835856<%2FAN><DT>Journal+Article<%2FDT>)

5.

Expression of bacteriocin divercin AS7 in Escherichia coli and its functional analysis.

Olejnik-Schmidt AK; Schmidt MT; Sip A; Szablewski T; Grajek W.

Annals of Microbiology. 64:1197-1202, 2014.

[Journal Article]

UI: 25100927

Bacteriocins are small peptides with antimicrobial activity, that are produced by bacteria. Four classes of bacteriocins produced by lactic acid bacteria have been defined. Class IIa bacteriocins are promising candidates for industrial applications due to their high biological activity and their physicochemical properties. Divercin AS7 is a class IIa bacteriocin produced by Carnobacterium divergens AS7. It shows antibacterial activity against pathogens and food spoilage flora including Listeria spp. Little is known about the impact of class IIa bacteriocins upon eukaryotic cells. The safe use of bacteriocins as food biopreservatives requires the absence of cytotoxicity to human cells. To analyze the impact of divercin AS7 on human enterocytes, we expressed the recombinant divercin AS7 in the Escherichia coli BL21DE3pLys strain and conducted in vitro studies to evaluate the safety of recombinant divercin AS7. No cytotoxic effect on differentiated monolayer Caco-2 cells and no apoptotic appearance were observed when recombinant divercin AS7 was used at a concentration of 2 mug ml-1. In our study, divercin AS7 also did not interfere with differentiated Caco-2 cells monolayer integrity. The obtained results suggest that divercin AS7 is a promising peptide for the food industry.

Version ID

1

Record Owner

From MEDLINE, a database of the U.S. National Library of Medicine.

Status

PubMed-not-MEDLINE

Authors Full Name

Olejnik-Schmidt, Agnieszka K; Schmidt, Marcin T; Sip, Anna; Szablewski, Tomasz; Grajek, Wlodzimierz.

Institution

Olejnik-Schmidt, Agnieszka K. Department of Biotechnology and Food Microbiology, Poznan University of Life Sciences, Wojska Polskiego Street No. 48, 60-627 Poznan, Poland. Schmidt, Marcin T. Department of Biotechnology and Food Microbiology, Poznan University of Life Sciences, Wojska Polskiego Street No. 48, 60-627 Poznan, Poland.

Sip, Anna. Department of Biotechnology and Food Microbiology, Poznan University of Life Sciences, Wojska Polskiego Street No. 48, 60-627 Poznan, Poland.

Szablewski, Tomasz. Department of Food Quality Management, Poznan University of Life Sciences, Wojska Polskiego Street No. 31, 60-624 Poznan, Poland.

Grajek, Wlodzimierz. Department of Biotechnology and Food Microbiology, Poznan University of Life Sciences, Wojska Polskiego Street No. 48, 60-627 Poznan, Poland.

Keyword Heading

Bacteriocins Biopreservatives

Divercin AS7

Human enterocytes.

Year of Publication

2014

Link to the Ovid Full Text or citation:

[Click here for full text options](https://libaccess.mcmaster.ca/login?url=http://ovidsp.ovid.com/ovidweb.cgi?T=JS&CSC=Y&NEWS=N&PAGE=fulltext&D=prem5&AN=25100927)

Link to the External Link Resolver:

[SFX](http://sfx.scholarsportal.info/mcmaster?sid=OVID:medline&id=pmid:25100927&id=doi:10.1007%2Fs13213-013-0759-x&issn=1590-4261&isbn=&volume=64&issue=3&spage=1197&pages=1197-1202&date=2014&title=Annals+of+Microbiology&atitle=Expression+of+bacteriocin+divercin+AS7+in+Escherichia+coli+and+its+functional+analysis.&aulast=Olejnik-Schmidt&pid=<author>Olejnik-Schmidt+AK%3BSchmidt+MT%3BSip+A%3BSzablewski+T%3BGrajek+W<%2Fauthor><AN>25100927<%2FAN><DT>Journal+Article<%2FDT>)

6.

Nested structure of intraspecific competition network in Carnobacterium maltaromaticum.

Ramia NE; Mangavel C; Gaiani C; Muller-Gueudin A; Taha S; Revol-Junelles AM; Borges F.

Scientific Reports. 10(1):7335, 2020 04 30.

[Journal Article]

UI: 32355239

While competition targeting food-borne pathogens is being widely documented, few studies have focused on competition among non-pathogenic food bacteria. Carnobacterium maltaromaticum is a genetically diverse lactic acid bacterium known for comprising several bacteriocinogenic strains with bioprotective potentialities against the food-borne pathogen Listeria monocytogenes. The aim of our study is to examine the network properties of competition among a collection of 73 strains of C. maltaromaticum and to characterize their individual interaction potential. The performed high-throughput competition assays, investigating 5 329 pairwise interactions, showed that intraspecific competition was major in C. maltaromaticum with approximately 56% of the sender strains antagonizing at least one receiver strain. A high diversity of inhibitory and sensitivity spectra was identified along with a majority of narrow inhibitory as well as sensitivity spectra. Through network analysis approach, we determined the highly nested architecture of C. maltaromaticum competition network, thus showing that competition in this species is determined by both the spectrum width of the inhibitory activity of sender strains and the spectrum width of the sensitivity of receiver strains. This study provides knowledge of the competition network in C. maltaromaticum that could be used in rational assembly of compatible microbial strains for the design of mixed starter cultures.

Version ID

1

Record Owner

From MEDLINE, a database of the U.S. National Library of Medicine.

Status

MEDLINE

Author NameID

Mangavel, Cecile; ORCID: <https://orcid.org/0000-0002-4582-8675> Gaiani, Claire; ORCID: <https://orcid.org/0000-0003-0434-8453>

Authors Full Name

Ramia, Nancy E; Mangavel, Cecile; Gaiani, Claire; Muller-Gueudin, Aurelie; Taha, Samir; Revol-Junelles, Anne-Marie; Borges, Frederic.

Institution

Ramia, Nancy E. Universite de Lorraine, LIBio, F-54000, Nancy, France. Ramia, Nancy E. Laboratoire de Biotechnologies Appliquees, EDST, Universite Libanaise, Tripoli, Lebanon.

Mangavel, Cecile. Universite de Lorraine, LIBio, F-54000, Nancy, France.

Gaiani, Claire. Universite de Lorraine, LIBio, F-54000, Nancy, France.

Muller-Gueudin, Aurelie. Universite de Lorraine, CNRS, Inria, IECL, F-54000, Nancy, France.

Taha, Samir. Laboratoire de Biotechnologies Appliquees, EDST, Universite Libanaise, Tripoli, Lebanon.

Revol-Junelles, Anne-Marie. Universite de Lorraine, LIBio, F-54000, Nancy, France.

Borges, Frederic. Universite de Lorraine, LIBio, F-54000, Nancy, France. frederic.borges@univ-lorraine.fr.

MeSH Heading

Animals. *Antibiosis. Bacteriocins. Binding, Competitive. *Carnobacterium/ph [Physiology]. Fish Products. Fishes/mi [Microbiology]. *Food Contamination. *Food Microbiology. Humans. Lactic Acid/me [Metabolism]. *Listeria monocytogenes/ph [Physiology]. Meat Products. *Microbial Sensitivity Tests. Species Specificity.

Registry Number/Name of Substance

0 (Bacteriocins). 33X04XA5AT (Lactic Acid).

Year of Publication

2020

Link to the Ovid Full Text or citation:

[Click here for full text options](https://libaccess.mcmaster.ca/login?url=http://ovidsp.ovid.com/ovidweb.cgi?T=JS&CSC=Y&NEWS=N&PAGE=fulltext&D=medl&AN=32355239)

Link to the External Link Resolver:

[SFX](http://sfx.scholarsportal.info/mcmaster?sid=OVID:medline&id=pmid:32355239&id=doi:10.1038%2Fs41598-020-63844-5&issn=2045-2322&isbn=&volume=10&issue=1&spage=7335&pages=7335&date=2020&title=Scientific+Reports&atitle=Nested+structure+of+intraspecific+competition+network+in+Carnobacterium+maltaromaticum.&aulast=Ramia&pid=<author>Ramia+NE%3BMangavel+C%3BGaiani+C%3BMuller-Gueudin+A%3BTaha+S%3BRevol-Junelles+AM%3BBorges+F<%2Fauthor><AN>32355239<%2FAN><DT>Journal+Article<%2FDT>)

7.

Gastric mucosal microbiota in a Mongolian population with gastric cancer and precursor conditions.

Gantuya B; El Serag HB; Matsumoto T; Ajami NJ; Uchida T; Oyuntsetseg K; Bolor D; Yamaoka Y.

Alimentary Pharmacology & Therapeutics. 51(8):770-780, 2020 04.

[Journal Article. Research Support, N.I.H., Extramural. Research Support, Non-U.S. Gov't]

UI: 32133670

BACKGROUND: Incidence and mortality of gastric cancer (GC) are high in Mongolia despite Helicobacter pylori in the Mongolian population being less virulent.

AIM: To evaluate gastric bacterial microbiota profiles in patients with GC and its precursor histological conditions.

METHODS: We conducted a case-control study among 48 GC and 120 noncancer patients (20 normal gastric mucosa [control], 20 gastritis, 40 with atrophy and 40 intestinal metaplasia [IM]). We performed 16S rRNA gene amplicon sequencing and compared taxonomic and functional prediction profiles based on the diagnosis group and H pylori infection status.

RESULTS: The highest overall bacterial alpha diversity metrics were observed in the control group, followed by the IM and cancer groups. The gastritis and atrophy groups had the least diversity. Lactobacilli and Enterococci were the dominant genus in several cancer patients especially in the absence of H pylori. In addition, Carnobacterium, Glutamicibacter, Paeniglutamicibacter, Fusobacterium and Parvimonas were associated with GC regardless of H pylori infection. Firmicutes were decreased in the gastritis and atrophy groups and increased in the IM and cancer groups. The functional metabolic activity of the Embden-Meyerhof-Parnas pathway and the utilization of sugar, were significantly increased in cancer group compared with the noncancer group.

CONCLUSION: Microbial factors other than H pylori may play a role in Mongolian GC. We identified novel associations between GC and the genera Enterococcus, Lactobacillus, Carnobacterium, Glutamicibacter, Paeniglutamicibacter, Fusobacterium, and Parvimonas.

Copyright © 2020 John Wiley & Sons Ltd.

Version ID

1

Record Owner

From MEDLINE, a database of the U.S. National Library of Medicine.

Status

MEDLINE

Author NameID

Gantuya, Boldbaatar; ORCID: <https://orcid.org/0000-0003-4293-0532> El Serag, Hashem B; ORCID: <https://orcid.org/0000-0001-5964-7579>

Yamaoka, Yoshio; ORCID: <https://orcid.org/0000-0002-1222-5819>

Authors Full Name

Gantuya, Boldbaatar; El Serag, Hashem B; Matsumoto, Takashi; Ajami, Nadim J; Uchida, Tomohisa; Oyuntsetseg, Khasag; Bolor, Dashdorj; Yamaoka, Yoshio.

Institution

Gantuya, Boldbaatar. Department of Gastroenterology, Mongolian National University of Medical Sciences, Ulaanbaatar, Mongolia. Gantuya, Boldbaatar. Endoscopy Unit, Mongolia-Japan Teaching Hospital, Mongolian National University of Medical Sciences, Ulaanbaatar, Mongolia.

El Serag, Hashem B. Department of Medicine, Gastroenterology and Hepatology Section, Baylor College of Medicine, Houston, TX, USA.

Matsumoto, Takashi. Department of Environmental and Preventive Medicine, Oita University of Medicine, Yufu, Japan.

Ajami, Nadim J. Department of Molecular Virology and Microbiology, Baylor College of Medicine, Houston, TX, USA.

Uchida, Tomohisa. Department of Molecular Pathology, Oita University of Medicine, Yufu, Japan.

Oyuntsetseg, Khasag. Department of Gastroenterology, Mongolian National University of Medical Sciences, Ulaanbaatar, Mongolia.

Oyuntsetseg, Khasag. Endoscopy Unit, Mongolia-Japan Teaching Hospital, Mongolian National University of Medical Sciences, Ulaanbaatar, Mongolia.

Bolor, Dashdorj. Department of Endoscopy, Mongolian National Cancer Center Hospital, Ulaanbaatar, Mongolia.

Yamaoka, Yoshio. Department of Medicine, Gastroenterology and Hepatology Section, Baylor College of Medicine, Houston, TX, USA.

Yamaoka, Yoshio. Department of Environmental and Preventive Medicine, Oita University of Medicine, Yufu, Japan.

Comments

Comment in (CIN) Comment in (CIN)

MeSH Heading

Adult. Aged. Asian Continental Ancestry Group/sn [Statistics & Numerical Data]. Case-Control Studies. Cross-Sectional Studies. Female. Gastric Mucosa/mi [Microbiology]. *Gastric Mucosa/pa [Pathology]. Gastritis/ep [Epidemiology]. Gastritis/mi [Microbiology]. Gastrointestinal Microbiome/ge [Genetics]. *Gastrointestinal Microbiome. Helicobacter Infections/co [Complications]. Helicobacter Infections/ep [Epidemiology]. Helicobacter pylori/ge [Genetics]. Humans. Incidence. Male. Metaplasia/co [Complications]. Metaplasia/ep [Epidemiology]. Metaplasia/pa [Pathology]. Middle Aged. Mongolia/ep [Epidemiology]. *Precancerous Conditions/ep [Epidemiology]. *Precancerous Conditions/mi [Microbiology]. RNA, Ribosomal, 16S/an [Analysis]. RNA, Ribosomal, 16S/ge [Genetics]. *Stomach Neoplasms/ep [Epidemiology]. *Stomach Neoplasms/mi [Microbiology]. Stomach Neoplasms/pa [Pathology].

Registry Number/Name of Substance

0 (RNA, Ribosomal, 16S).

Year of Publication

2020

Link to the Ovid Full Text or citation:

[Click here for full text options](https://libaccess.mcmaster.ca/login?url=http://ovidsp.ovid.com/ovidweb.cgi?T=JS&CSC=Y&NEWS=N&PAGE=fulltext&D=medl&AN=32133670)

Link to the External Link Resolver:

[SFX](http://sfx.scholarsportal.info/mcmaster?sid=OVID:medline&id=pmid:32133670&id=doi:10.1111%2Fapt.15675&issn=0269-2813&isbn=&volume=51&issue=8&spage=770&pages=770-780&date=2020&title=Alimentary+Pharmacology+%26+Therapeutics&atitle=Gastric+mucosal+microbiota+in+a+Mongolian+population+with+gastric+cancer+and+precursor+conditions.&aulast=Gantuya&pid=<author>Gantuya+B%3BEl+Serag+HB%3BMatsumoto+T%3BAjami+NJ%3BUchida+T%3BOyuntsetseg+K%3BBolor+D%3BYamaoka+Y<%2Fauthor><AN>32133670<%2FAN><DT>Journal+Article<%2FDT>)

8.

Shelf-life and microbial community dynamics of super-chilled beef imported from Australia to China.

Chen X; Zhang Y; Yang X; Hopkins DL; Zhu L; Dong P; Liang R; Luo X.

Food Research International. 120:784-792, 2019 06.

[Journal Article. Research Support, Non-U.S. Gov't]

UI: 31000298

The aim of this study was to investigate the shelf-life and microbial community dynamics of super-chilled vacuum-packaged beef striploins imported from Australia to China after approximately five weeks of shipping time and an additional 15weeks of storage at -1degreeC+/-0.5degreeC. Data analysis using a mixed model (REML) with time as the fixed effect and portion as a random effect, showed that the only beef quality trait that changed during storage was total volatile basic nitrogen (TVBN; P<.05), which reached the threshold of 15mg/100g between 15 and 20weeks (including 5weeks of transport). The total viable count (TVC) accounted for 78% of the variance in TVBN, when storage time was included in the model. Sensory scores decreased as storage time extended (P<.05), but were still acceptable at 20weeks. After 9weeks, Carnobacterium spp. and Lactobacillus spp. dominated alternately and then Lactobacillus became the most prevalent bacteria. An operational taxominc unit based hierarchical cluster analysis using the unweighted pair-group method with arithmetic means was performed and it was shown that the bacterial communities tended to be consistent as storage time extended. Overall indications are that beef which is safe can be imported into China from Australia and aged for extended periods.

Copyright © 2018 Elsevier Ltd. All rights reserved.

Version ID

1

Record Owner

From MEDLINE, a database of the U.S. National Library of Medicine.

Status

MEDLINE

Authors Full Name

Chen, Xue; Zhang, Yimin; Yang, Xiaoyin; Hopkins, David L; Zhu, Lixian; Dong, Pengcheng; Liang, Rongrong; Luo, Xin.

Institution

Chen, Xue. Lab of Beef Processing and Quality Control, College of Food Science and Engineering, Shandong Agricultural University, Tai'an, Shandong 271018, PR China. Zhang, Yimin. Lab of Beef Processing and Quality Control, College of Food Science and Engineering, Shandong Agricultural University, Tai'an, Shandong 271018, PR China. Electronic address: ymzhang@sdau.edu.cn.

Yang, Xiaoyin. Lab of Beef Processing and Quality Control, College of Food Science and Engineering, Shandong Agricultural University, Tai'an, Shandong 271018, PR China.

Hopkins, David L. Lab of Beef Processing and Quality Control, College of Food Science and Engineering, Shandong Agricultural University, Tai'an, Shandong 271018, PR China; NSW Department of Primary Industries, Centre for Red Meat and Sheep Development, PO Box 129, Cowra, NSW 2794, Australia.

Zhu, Lixian. Lab of Beef Processing and Quality Control, College of Food Science and Engineering, Shandong Agricultural University, Tai'an, Shandong 271018, PR China.

Dong, Pengcheng. Lab of Beef Processing and Quality Control, College of Food Science and Engineering, Shandong Agricultural University, Tai'an, Shandong 271018, PR China.

Liang, Rongrong. Lab of Beef Processing and Quality Control, College of Food Science and Engineering, Shandong Agricultural University, Tai'an, Shandong 271018, PR China.

Luo, Xin. Lab of Beef Processing and Quality Control, College of Food Science and Engineering, Shandong Agricultural University, Tai'an, Shandong 271018, PR China; Jiangsu Synergetic Innovation Center of Meat Production and Processing Quality and Safety Control, Nanjing, Jiangsu 210000, PR China.

MeSH Heading

Australia. China. Color. *Food Microbiology/mt [Methods]. *Food Preservation/mt [Methods]. *Food Quality. *Food Storage/mt [Methods]. Humans. Hydrogen-Ion Concentration. Lipids. *Meat-Packing Industry/mt [Methods]. *Microbiota. Oxidation-Reduction. Red Meat.

Keyword Heading

*Beef *High-throughput sequencing

*Microbial diversity

*Shelf-life

*Super-chilled.

Registry Number/Name of Substance

0 (Lipids).

Year of Publication

2019

Link to the Ovid Full Text or citation:

[Click here for full text options](https://libaccess.mcmaster.ca/login?url=http://ovidsp.ovid.com/ovidweb.cgi?T=JS&CSC=Y&NEWS=N&PAGE=fulltext&D=medl&AN=31000298)

Link to the External Link Resolver:

[SFX](http://sfx.scholarsportal.info/mcmaster?sid=OVID:medline&id=pmid:31000298&id=doi:10.1016%2Fj.foodres.2018.11.039&issn=0963-9969&isbn=&volume=120&issue=&spage=784&pages=784-792&date=2019&title=Food+Research+International&atitle=Shelf-life+and+microbial+community+dynamics+of+super-chilled+beef+imported+from+Australia+to+China.&aulast=Chen&pid=<author>Chen+X%3BZhang+Y%3BYang+X%3BHopkins+DL%3BZhu+L%3BDong+P%3BLiang+R%3BLuo+X<%2Fauthor><AN>31000298<%2FAN><DT>Journal+Article<%2FDT>)

9.

Changes in intestinal microflora in digestive tract diseases during pregnancy.

Jin M; Li D; Ji R; Liu W; Xu X; Li Y.

Archives of Gynecology & Obstetrics. 301(1):243-249, 2020 01.

[Journal Article. Research Support, Non-U.S. Gov't]

UI: 31776707

PURPOSE: This study aimed to investigate the gut microbiome composition in pregnant women with digestive diseases to analyze the relationships between the microflora changes and digestive diseases during pregnancy.

METHODS: Fecal samples obtained from 71 pregnant women [six acute fatty liver (AF group), 21 constipation (C group), 24 excessive vomiting (V group) and 20 normal pregnancy (CP group)] and 26 non-pregnant (NP group) women were subjected to 16 s rRNA sequencing. Differential analysis of intestinal flora at the genera level was performed.

RESULTS: The relative abundance of Coprobacillus, Acinetobacter, Enterococcus, Weissella and Lysinibacillus was increased in the digestive diseases (AF, C and V) groups compared with CP group, whereas that of five common genera, including Terrisporobacter, Dysgonomonas, Adlercreutzia, Fusicatenibacter and Blautia, was decreased in digestive diseases groups. Additionally, in digestive diseases (AF, C and V) groups, the abundance of 13 common genera, such as Carnobacterium, Coprobacillus and Psychrobacter, was higher than NP group, whereas that of 27 common genera, such as Blautia and Terrisporobacter, was lower than NP group. About 69 genera were differentially abundant between AF and C groups; two genera (Aerococcus and Senegalimassilia) were identified between AF and V groups; moreover, total 63 genera were obtained between C and V groups.

CONCLUSION: Our data revealed that the abundance of Acinetobacter, Enterococci, Paenibacillus, Blautia and Collinsella might be associated with the digestive diseases during pregnancy. These findings further supported the idea that targeting the gut microbiota could be a new prevention or therapeutic approach for improving digestive diseases during pregnancy.

Version ID

1

Record Owner

From MEDLINE, a database of the U.S. National Library of Medicine.

Status

MEDLINE

Author NameID

Li, Yanqing; ORCID: <https://orcid.org/0000-0003-0575-0399>

Authors Full Name

Jin, Min; Li, Dong; Ji, Rui; Liu, Wen; Xu, Xiaofei; Li, Yanqing.

Institution

Jin, Min. Department of Anesthesiology, Qilu Hospital, Shandong University, Jinan, 250012, Shandong, China. Li, Dong. Stem Cell and Regenerative Medicine Center of Shandong University, Jinan, 250012, Shandong, China.

Ji, Rui. Department of Gastroenterology, Qilu Hospital, Shandong University, Jinan, 250012, Shandong, China.

Liu, Wen. Department of Obstetrics and Gynecology, Center for Reproductive Medicine, Qilu Hospital, Shandong University, Jinan, 250012, Shandong, China.

Xu, Xiaofei. Department of Obstetrics and Gynecology, Center for Reproductive Medicine, Qilu Hospital, Shandong University, Jinan, 250012, Shandong, China.

Li, Yanqing. Department of Gastroenterology, Laboratory of Translational Gastroenterology, Qilu Hospital, Shandong University, Jizhong Building, 107 Wen hua Xi Road, Lixia District, Jinan, 250012, Shandong, China. liyanqing@sdu.edu.cn.

MeSH Heading

Adult. Female. *Gastrointestinal Diseases/pa [Pathology]. *Gastrointestinal Microbiome/ph [Physiology]. Humans. Pregnancy. Young Adult.

Keyword Heading

*16 s rRNA sequencing *Digestive diseases

*Intestinal microflora

*Pregnancy.

Year of Publication

2020

Link to the Ovid Full Text or citation:

[Click here for full text options](https://libaccess.mcmaster.ca/login?url=http://ovidsp.ovid.com/ovidweb.cgi?T=JS&CSC=Y&NEWS=N&PAGE=fulltext&D=medl&AN=31776707)

Link to the External Link Resolver:

[SFX](http://sfx.scholarsportal.info/mcmaster?sid=OVID:medline&id=pmid:31776707&id=doi:10.1007%2Fs00404-019-05336-0&issn=0932-0067&isbn=&volume=301&issue=1&spage=243&pages=243-249&date=2020&title=Archives+of+Gynecology+%26+Obstetrics&atitle=Changes+in+intestinal+microflora+in+digestive+tract+diseases+during+pregnancy.&aulast=Jin&pid=<author>Jin+M%3BLi+D%3BJi+R%3BLiu+W%3BXu+X%3BLi+Y<%2Fauthor><AN>31776707<%2FAN><DT>Journal+Article<%2FDT>)

10.

Functional characterization of the alanine-serine-cysteine exchanger of Carnobacterium sp AT7.

Bartoccioni P; Fort J; Zorzano A; Errasti-Murugarren E; Palacin M.

Journal of General Physiology. 151(4):505-517, 2019 04 01.

[Journal Article. Research Support, Non-U.S. Gov't]

UI: 30696726

Many key cell processes require prior cell uptake of amino acids from the environment, which is facilitated by cell membrane amino acid transporters such as those of the L-type amino acid transporter (LAT) subfamily. Alterations in LAT subfamily amino acid transport are associated with several human diseases, including cancer, aminoacidurias, and neurodegenerative conditions. Therefore, from the perspective of human health, there is considerable interest in obtaining structural information about these transporter proteins. We recently solved the crystal structure of the first LAT transporter, the bacterial alanine-serine-cysteine exchanger of Carnobacterium sp AT7 (BasC). Here, we provide a complete functional characterization of detergent-purified, liposome-reconstituted BasC transporter to allow the extension of the structural insights into mechanistic understanding. BasC is a sodium- and proton-independent small neutral amino acid exchanger whose substrate and inhibitor selectivity are almost identical to those previously described for the human LAT subfamily member Asc-1. Additionally, we show that, like its human counterparts, this transporter has apparent affinity asymmetry for the intra- and extracellular substrate binding sites-a key feature in the physiological role played by these proteins. BasC is an excellent paradigm of human LAT transporters and will contribute to our understanding of the molecular mechanisms underlying substrate recognition and translocation at both sides of the plasma membrane.

Copyright © 2019 Bartoccioni et al.

Version ID

1

Record Owner

From MEDLINE, a database of the U.S. National Library of Medicine.

Status

MEDLINE

Author NameID

Fort, Joana; ORCID: <https://orcid.org/0000-0003-0399-2116> Errasti-Murugarren, Ekaitz; ORCID: <https://orcid.org/0000-0002-6054-7900>

Authors Full Name

Bartoccioni, Paola; Fort, Joana; Zorzano, Antonio; Errasti-Murugarren, Ekaitz; Palacin, Manuel.

Institution

Bartoccioni, Paola. Institute for Research in Biomedicine, Barcelona Institute of Science and Technology, Barcelona, Spain. Bartoccioni, Paola. Centro de Investigacion Biomedica en Red de Enfermedades Raras, Barcelona, Spain.

Fort, Joana. Institute for Research in Biomedicine, Barcelona Institute of Science and Technology, Barcelona, Spain.

Fort, Joana. Centro de Investigacion Biomedica en Red de Enfermedades Raras, Barcelona, Spain.

Fort, Joana. Department of Biochemistry and Molecular Biomedicine, Faculty of Biology, University of Barcelona, Barcelona, Spain.

Zorzano, Antonio. Institute for Research in Biomedicine, Barcelona Institute of Science and Technology, Barcelona, Spain.

Zorzano, Antonio. Department of Biochemistry and Molecular Biomedicine, Faculty of Biology, University of Barcelona, Barcelona, Spain.

Zorzano, Antonio. Centro de Investigacion Biomedica en Red de Diabetes y Enfermedades Metabolicas Asociadas, Barcelona, Spain.

Errasti-Murugarren, Ekaitz. Institute for Research in Biomedicine, Barcelona Institute of Science and Technology, Barcelona, Spain ekaitz.errasti@irbbarcelona.org.

Palacin, Manuel. Institute for Research in Biomedicine, Barcelona Institute of Science and Technology, Barcelona, Spain manuel.palacin@irbbarcelona.org.

Palacin, Manuel. Centro de Investigacion Biomedica en Red de Enfermedades Raras, Barcelona, Spain.

Palacin, Manuel. Department of Biochemistry and Molecular Biomedicine, Faculty of Biology, University of Barcelona, Barcelona, Spain.

MeSH Heading

*Amino Acid Transport System y+/ch [Chemistry]. *Amino Acid Transport System y+/me [Metabolism]. *Bacterial Proteins/ch [Chemistry]. Bacterial Proteins/me [Metabolism]. *Carnobacterium/me [Metabolism]. Cloning, Molecular. Gene Expression Regulation, Bacterial.

Registry Number/Name of Substance

0 (Amino Acid Transport System y+). 0 (Bacterial Proteins).

Year of Publication

2019

Link to the Ovid Full Text or citation:

[Click here for full text options](https://libaccess.mcmaster.ca/login?url=http://ovidsp.ovid.com/ovidweb.cgi?T=JS&CSC=Y&NEWS=N&PAGE=fulltext&D=medl&AN=30696726)

Link to the External Link Resolver:

[SFX](http://sfx.scholarsportal.info/mcmaster?sid=OVID:medline&id=pmid:30696726&id=doi:10.1085%2Fjgp.201812195&issn=0022-1295&isbn=&volume=151&issue=4&spage=505&pages=505-517&date=2019&title=Journal+of+General+Physiology&atitle=Functional+characterization+of+the+alanine-serine-cysteine+exchanger+of+Carnobacterium+sp+AT7.&aulast=Bartoccioni&pid=<author>Bartoccioni+P%3BFort+J%3BZorzano+A%3BErrasti-Murugarren+E%3BPalacin+M<%2Fauthor><AN>30696726<%2FAN><DT>Journal+Article<%2FDT>)

11.

Understanding the association between the human gut, oral and skin microbiome and the Ayurvedic concept of prakriti.

Chaudhari D; Dhotre D; Agarwal D; Gondhali A; Nagarkar A; Lad V; Patil U; Juvekar S; Sinkar V; Shouche Y.

Journal of Biosciences. 44(5), 2019 Oct.

[Journal Article]

UI: 31719221

Ayurveda is one of the ancient systems of medicine which is widely practised as a personalized scientific approach towards the general wellness. Ayurvedic prakriti is broadly defined as the phenotypes which are determined on the basis of physical, psychological and physiological traits irrespective of their social, ethnic, dietary and geographical stature. Prakriti is the constitution of a person, which comprises vata, pitta, and kapha and is a key determinant of how one individual is different from the other. Human microbiome is considered the 'latest discovered' human organ and microbiome research reiterates the fundamental principles of Ayurveda for creating a healthy gut environment by maintaining the individual-specific microbiome. Hence, it is important to understand the association of human microbiome with the Ayurvedic prakriti of an individual. Here, we provide a comprehensive analysis of human microbiome from the gut, oral and skin samples of healthy individuals (n=18) by 16S rRNA gene-based metagenomics using standard QIIME pipeline. In the three different prakriti samples differential abundance of Bacteroides, Desulfovibrio, Parabacteroides, Slackia, and Succinivibrio was observed in the gut microbiome. Analysis also revealed prakriti-specific presence of Mogibacterium, Propionibacterium, Pyramidobacter, Rhodococcus in the kapha prakriti individuals Planomicrobium, Hyphomicrobium, Novosphingobium in the pitta prakriti individuals and Carnobacterium, Robiginitalea, Cetobacterium, Psychrobacter in the vata prakriti individuals. Similarly, the oral and skin microbiome also revealed presence of prakriti-specific differential abundance of diverse bacterial genera. Prakriti-specific presence of bacterial taxa was recorded and only 42% microbiome in the oral samples and 52% microbiome in the skin samples were shared. Bacteria known for preventing gut inflammation by digesting the resistant starch were abundant in the pitta prakriti individuals, who are more prone to develop gut-inflammation-related disorders. In summary, human gut, oral and skin microbiome showed presence or high abundance of few bacterial taxa across three prakriti types, suggesting their specific physiological importance.

Version ID

1

Record Owner

From MEDLINE, a database of the U.S. National Library of Medicine.

Status

MEDLINE

Authors Full Name

Chaudhari, Diptaraj; Dhotre, Dhiraj; Agarwal, Dhiraj; Gondhali, Arun; Nagarkar, Anand; Lad, Vikas; Patil, Ulhas; Juvekar, Sanjay; Sinkar, Vilas; Shouche, Yogesh.

Institution

Chaudhari, Diptaraj. National Centre for Microbial Resource, National Centre for Cell Science, Central Tower, Sai Trinity Building Garware Circle, Sutarwadi, Pashan, Pune, India.

MeSH Heading

Female. Humans. *Intestines/mi [Microbiology]. Male. *Medicine, Ayurvedic. *Microbiota. *Mouth/mi [Microbiology]. *Skin/mi [Microbiology].

Year of Publication

2019

Link to the Ovid Full Text or citation:

[Click here for full text options](https://libaccess.mcmaster.ca/login?url=http://ovidsp.ovid.com/ovidweb.cgi?T=JS&CSC=Y&NEWS=N&PAGE=fulltext&D=medl&AN=31719221)

Link to the External Link Resolver:

[SFX](http://sfx.scholarsportal.info/mcmaster?sid=OVID:medline&id=pmid:31719221&id=doi:&issn=0250-5991&isbn=&volume=44&issue=5&spage=&pages=&date=2019&title=Journal+of+Biosciences&atitle=Understanding+the+association+between+the+human+gut%2C+oral+and+skin+microbiome+and+the+Ayurvedic+concept+of+prakriti.&aulast=Chaudhari&pid=<author>Chaudhari+D%3BDhotre+D%3BAgarwal+D%3BGondhali+A%3BNagarkar+A%3BLad+V%3BPatil+U%3BJuvekar+S%3BSinkar+V%3BShouche+Y<%2Fauthor><AN>31719221<%2FAN><DT>Journal+Article<%2FDT>)

12.

Changes in the microbial communities of air- and water-chilled yellow-feathered broilers during storage at 2 degreeC.

Wang H; Qin X; Li X; Wang X; Gao H; Zhang C.

Food Microbiology. 87:103390, 2020 May.

[Journal Article]

UI: 31948631

Carcass chilling is a critical step in broiler processing. Understanding the effect of chilling on the bacterial communities of broilers is important, as these communities may be largely responsible for the spoilage process. This study examined the effect of chilling systems (air chilling [AC] and water chilling [WC]) and subsequent aerobic storage on the microbiota of yellow-feathered broiler carcasses using a high-throughput sequencing technique targeting the V3-V4 region of the 16S RNA gene. Evidence of the clear differences in the microbiota structures between AC and WC carcasses was illustrated by principle coordinates and heat map clustered analyses. The distinctions between the AC and WC carcass bacterial communities were more pronounced during the later storage stages. The major genera on the spoiled AC carcasses were Pseudomonas, Psychrobacter and Shewanella, whereas the major genera on the spoiled WC carcasses were Psychrobacter, Pseudomonas and Carnobacterium. These data suggest that the chilling method has a marked effect on the microbiota composition of yellow-feathered broilers along the entire storage period. The chilling method was also of great importance for surface color. However, there was no significant difference in the sensorial shelf-life of chicken when comparing the chilling methods.

Copyright © 2019. Published by Elsevier Ltd.

Version ID

1

Record Owner

From MEDLINE, a database of the U.S. National Library of Medicine.

Status

MEDLINE

Authors Full Name

Wang, Hang; Qin, Xiaojie; Li, Xia; Wang, Xiaoling; Gao, Hongwei; Zhang, Chunhui.

Institution

Wang, Hang. Institute of Food Science and Technology, Chinese Academy of Agricultural Sciences, PR China. Qin, Xiaojie. Institute of Food Science and Technology, Chinese Academy of Agricultural Sciences, PR China.

Li, Xia. Institute of Food Science and Technology, Chinese Academy of Agricultural Sciences, PR China.

Wang, Xiaoling. Xinjiang Pagelang Food Co., Ltd, Urumchi City, Xinjiang Province, PR China.

Gao, Hongwei. Taikun Group Co., Ltd, Changji City, Xinjiang Province, PR China.

Zhang, Chunhui. Institute of Food Science and Technology, Chinese Academy of Agricultural Sciences, PR China. Electronic address: zhangchunhui@caas.cn.

MeSH Heading

Animals. Bacteria/cl [Classification]. Bacteria/ge [Genetics]. Bacteria/ip [Isolation & Purification]. Chickens/mi [Microbiology]. Cold Temperature. Colony Count, Microbial. Food Storage. Humans. *Meat/mi [Microbiology]. *Microbiota. Taste. Water/ch [Chemistry].

Keyword Heading

Bacterial communities Chilling method

High-throughput sequencing

Poultry

Storage.

Registry Number/Name of Substance

059QF0KO0R (Water).

Year of Publication

2020

Link to the Ovid Full Text or citation:

[Click here for full text options](https://libaccess.mcmaster.ca/login?url=http://ovidsp.ovid.com/ovidweb.cgi?T=JS&CSC=Y&NEWS=N&PAGE=fulltext&D=medl&AN=31948631)

Link to the External Link Resolver:

[SFX](http://sfx.scholarsportal.info/mcmaster?sid=OVID:medline&id=pmid:31948631&id=doi:10.1016%2Fj.fm.2019.103390&issn=0740-0020&isbn=&volume=87&issue=&spage=103390&pages=103390&date=2020&title=Food+Microbiology&atitle=Changes+in+the+microbial+communities+of+air-+and+water-chilled+yellow-feathered+broilers+during+storage+at+2+degreeC.&aulast=Wang&pid=<author>Wang+H%3BQin+X%3BLi+X%3BWang+X%3BGao+H%3BZhang+C<%2Fauthor><AN>31948631<%2FAN><DT>Journal+Article<%2FDT>)

13.

Isolation of Carnobacterium sp. from a human blood culture.

Hoenigl M; Grisold AJ; Valentin T; Leitner E; Zarfel G; Renner H; Krause R.

Journal of Medical Microbiology. 59(Pt 4):493-495, 2010 Apr.

[Case Reports. Journal Article]

UI: 20075110

Carnobacterium species have been isolated from the environment and are not regarded as human pathogens, although they are known to cause disease in fish. Only two reports describing isolation of Carnobacterium species from human pus were found in the literature. We report what we believe to be the first isolation of Carnobacterium sp. from a human blood culture.

Version ID

1

Record Owner

From MEDLINE, a database of the U.S. National Library of Medicine.

Status

MEDLINE

Authors Full Name

Hoenigl, Martin; Grisold, Andrea J; Valentin, Thomas; Leitner, Eva; Zarfel, Gernot; Renner, Heiko; Krause, Robert.

Institution

Hoenigl, Martin. Section of Infectious Diseases, Department of InternalMedicine, Medical University of Graz, A-8010 Graz, Austria. Grisold, Andrea J. Institute of Hygiene, Microbiology and EnvironmentalMedicine, Medical University of Graz, A-8010 Graz, Austria.

Valentin, Thomas. Section of Infectious Diseases, Department of InternalMedicine, Medical University of Graz, A-8010 Graz, Austria.

Leitner, Eva. Institute of Hygiene, Microbiology and EnvironmentalMedicine, Medical University of Graz, A-8010 Graz, Austria.

Zarfel, Gernot. Institute of Hygiene, Microbiology and EnvironmentalMedicine, Medical University of Graz, A-8010 Graz, Austria.

Renner, Heiko. Division of Thoracic Surgery, Medical Universityof Graz, A-8010 Graz, Austria.

Krause, Robert. Section of Infectious Diseases, Department of InternalMedicine, Medical University of Graz, A-8010 Graz, Austria.

MeSH Heading

Adult. *Bacteremia/mi [Microbiology]. Carnobacterium/de [Drug Effects]. *Carnobacterium/ip [Isolation & Purification]. Humans. Male. Microbial Sensitivity Tests.

Year of Publication

2010

Link to the Ovid Full Text or citation:

[Click here for full text options](https://libaccess.mcmaster.ca/login?url=http://ovidsp.ovid.com/ovidweb.cgi?T=JS&CSC=Y&NEWS=N&PAGE=fulltext&D=medc&AN=20075110)

Link to the External Link Resolver:

[SFX](http://sfx.scholarsportal.info/mcmaster?sid=OVID:medline&id=pmid:20075110&id=doi:10.1099%2Fjmm.0.016808-0&issn=0022-2615&isbn=&volume=59&issue=4&spage=493&pages=493-495&date=2010&title=Journal+of+Medical+Microbiology&atitle=Isolation+of+Carnobacterium+sp.+from+a+human+blood+culture.&aulast=Hoenigl&pid=<author>Hoenigl+M%3BGrisold+AJ%3BValentin+T%3BLeitner+E%3BZarfel+G%3BRenner+H%3BKrause+R<%2Fauthor><AN>20075110<%2FAN><DT>Case+Reports<%2FDT>)

14.

Perinatal factors affect the gut microbiota up to four years after birth.

Fouhy F; Watkins C; Hill CJ; O'Shea CA; Nagle B; Dempsey EM; O'Toole PW; Ross RP; Ryan CA; Stanton C.

Nature communications . 10(1):1517, 2019 04 03.

[Journal Article. Research Support, Non-U.S. Gov't]

UI: 30944304

Perinatal factors impact gut microbiota development in early life, however, little is known on the effects of these factors on microbes in later life. Here we sequence DNA from faecal samples of children over the first four years and reveal a perpetual evolution of the gut microbiota during this period. The significant impact of gestational age at birth and delivery mode on gut microbiota progression is evident in the first four years of life, while no measurable effects of antibiotics are found in the first year. Microbiota profiles are also characteristic in children dependant on gestational age and maturity. Full term delivery is characterised by Bacteroides (year one), Parabacteroides (year two) and Christensenellaceae (year four). Preterm delivery is characterised by Lactobacillus (year one), Streptococcus (year two) and Carnobacterium (year four). This study reveals that the gut retains distinct microbial profiles of perinatal factors up to four years of age.

Version ID

1

Record Owner

From MEDLINE, a database of the U.S. National Library of Medicine.

Status

MEDLINE

Author NameID

Fouhy, Fiona; ORCID: <http://orcid.org/0000-0002-8285-7694>

Authors Full Name

Fouhy, Fiona; Watkins, Claire; Hill, Cian J; O'Shea, Carol-Anne; Nagle, Brid; Dempsey, Eugene M; O'Toole, Paul W; Ross, R Paul; Ryan, C Anthony; Stanton, Catherine.

Institution

Fouhy, Fiona. APC Microbiome Ireland, Cork, T12 YT20, Ireland. Fouhy, Fiona. Teagasc Food Research Centre, Moorepark, Fermoy, Co, Cork, P61 C996, Ireland.

Watkins, Claire. APC Microbiome Ireland, Cork, T12 YT20, Ireland.

Watkins, Claire. Teagasc Food Research Centre, Moorepark, Fermoy, Co, Cork, P61 C996, Ireland.

Hill, Cian J. APC Microbiome Ireland, Cork, T12 YT20, Ireland.

O'Shea, Carol-Anne. Department of Neonatology, Cork University Maternity Hospital, Cork, T12 YE02, Ireland.

Nagle, Brid. Teagasc Food Research Centre, Moorepark, Fermoy, Co, Cork, P61 C996, Ireland.

Dempsey, Eugene M. Department of Neonatology, Cork University Maternity Hospital, Cork, T12 YE02, Ireland.

Dempsey, Eugene M. INFANT Centre, University College Cork, Cork, T12 YT20, Ireland.

O'Toole, Paul W. APC Microbiome Ireland, Cork, T12 YT20, Ireland.

O'Toole, Paul W. School of Microbiology, University College Cork, Cork, T12 YT20, Ireland.

Ross, R Paul. APC Microbiome Ireland, Cork, T12 YT20, Ireland.

Ross, R Paul. School of Microbiology, University College Cork, Cork, T12 YT20, Ireland.

Ryan, C Anthony. APC Microbiome Ireland, Cork, T12 YT20, Ireland.

Ryan, C Anthony. Department of Neonatology, Cork University Maternity Hospital, Cork, T12 YE02, Ireland.

Stanton, Catherine. APC Microbiome Ireland, Cork, T12 YT20, Ireland. catherine.stanton@teagasc.ie.

Stanton, Catherine. Teagasc Food Research Centre, Moorepark, Fermoy, Co, Cork, P61 C996, Ireland. catherine.stanton@teagasc.ie.

MeSH Heading

Anti-Bacterial Agents/pd [Pharmacology]. Bacteria/cl [Classification]. Bacteria/ge [Genetics]. Bacteria/ip [Isolation & Purification]. Child, Preschool. Feces/mi [Microbiology]. Female. Gastrointestinal Microbiome/de [Drug Effects]. Gastrointestinal Microbiome/ge [Genetics]. *Gastrointestinal Microbiome/ph [Physiology]. *Gastrointestinal Tract/mi [Microbiology]. Humans. Infant. Infant, Newborn. Male. *Microbiota/ph [Physiology]. Pregnancy/de [Drug Effects]. *Pregnancy/ph [Physiology]. Premature Birth. RNA, Ribosomal, 16S/ge [Genetics].

Registry Number/Name of Substance

0 (Anti-Bacterial Agents). 0 (RNA, Ribosomal, 16S).

Year of Publication

2019

Link to the Ovid Full Text or citation:

[Click here for full text options](https://libaccess.mcmaster.ca/login?url=http://ovidsp.ovid.com/ovidweb.cgi?T=JS&CSC=Y&NEWS=N&PAGE=fulltext&D=medc&AN=30944304)

Link to the External Link Resolver:

[SFX](http://sfx.scholarsportal.info/mcmaster?sid=OVID:medline&id=pmid:30944304&id=doi:10.1038%2Fs41467-019-09252-4&issn=2041-1723&isbn=&volume=10&issue=1&spage=1517&pages=1517&date=2019&title=Nature+communications+&atitle=Perinatal+factors+affect+the+gut+microbiota+up+to+four+years+after+birth.&aulast=Fouhy&pid=<author>Fouhy+F%3BWatkins+C%3BHill+CJ%3BO'Shea+CA%3BNagle+B%3BDempsey+EM%3BO'Toole+PW%3BRoss+RP%3BRyan+CA%3BStanton+C<%2Fauthor><AN>30944304<%2FAN><DT>Journal+Article<%2FDT>)

15.

Twenty-Three Species of Hypobarophilic Bacteria Recovered from Diverse Ecosystems Exhibit Growth under Simulated Martian Conditions at 0.7 kPa.

Schuerger AC; Nicholson WL.

Astrobiology. 16(5):335-47, 2016 May.

[Journal Article. Retracted Publication]

UI: 27135839

UNLABELLED: Bacterial growth at low pressure is a new research area with implications for predicting microbial activity in clouds and the bulk atmosphere on Earth, and for modeling the forward contamination of planetary surfaces like Mars. Here, we describe experiments on the recovery and identification of 23 species of bacterial hypobarophiles (def., growth under hypobaric conditions of approximately 1-2 kPa) in 11 genera capable of growth at 0.7 kPa. Hypobarophilic bacteria, but not archaea or fungi, were recovered from soil and non-soil ecosystems. The highest numbers of hypobarophiles were recovered from Arctic soil, Siberian permafrost, and human saliva. Isolates were identified through 16S rRNA sequencing to belong to the genera Carnobacterium, Exiguobacterium, Leuconostoc, Paenibacillus, and Trichococcus. The highest population of culturable hypobarophilic bacteria (5.1 x 10(4) cfu/g) was recovered from Colour Lake soils from Axel Heiberg Island in the Canadian Arctic. In addition, we extend the number of hypobarophilic species in the genus Serratia to six type-strains that include S. ficaria, S. fonticola, S. grimesii, S. liquefaciens, S. plymuthica, and S. quinivorans. Microbial growth at 0.7 kPa suggests that pressure alone will not be growth-limiting on the martian surface or in Earth's atmosphere up to an altitude of 34 km.

KEY WORDS: Planetary protection-Simulated martian atmosphere-Piezophile-Habitability-Extremophilic microorganisms. Astrobiology 16, 335-347.

Version ID

1

Record Owner

From MEDLINE, a database of the U.S. National Library of Medicine.

Status

PubMed-not-MEDLINE

Authors Full Name

Schuerger, Andrew C; Nicholson, Wayne L.

Institution

Schuerger, Andrew C. 1 Department of Plant Pathology, University of Florida, Gainesville, Florida. Nicholson, Wayne L. 2 Department of Microbiology and Cell Science, University of Florida, Gainesville, Florida.

Comments

Retraction in (RIN)

Year of Publication

2016

Link to the Ovid Full Text or citation:

[Click here for full text options](https://libaccess.mcmaster.ca/login?url=http://ovidsp.ovid.com/ovidweb.cgi?T=JS&CSC=Y&NEWS=N&PAGE=fulltext&D=prem2&AN=27135839)

Link to the External Link Resolver:

[SFX](http://sfx.scholarsportal.info/mcmaster?sid=OVID:medline&id=pmid:27135839&id=doi:10.1089%2Fast.2015.1394&issn=1557-8070&isbn=&volume=16&issue=5&spage=335&pages=335-47&date=2016&title=Astrobiology&atitle=Twenty-Three+Species+of+Hypobarophilic+Bacteria+Recovered+from+Diverse+Ecosystems+Exhibit+Growth+under+Simulated+Martian+Conditions+at+0.7+kPa.&aulast=Schuerger&pid=<author>Schuerger+AC%3BNicholson+WL<%2Fauthor><AN>27135839<%2FAN><DT>Journal+Article<%2FDT>)

16.

Microbial spoilage investigation of thawed common cuttlefish (Sepia officinalis) stored at 2degreeC using next generation sequencing and volatilome analysis.

Parlapani FF; Michailidou S; Anagnostopoulos DA; Sakellariou AK; Pasentsis K; Psomopoulos F; Argiriou A; Haroutounian SA; Boziaris IS.

Food Microbiology. 76:518-525, 2018 Dec.

[Journal Article]

UI: 30166182

Cephalopods are highly appreciated with increasing demand seafood, but are also very perishable and deteriorate fast mainly due to microbiological spoilage. For this reason exploration of bacterial communities through 16S Next Generation Sequencing (NGS) and Volatile Organic Compounds (VOCs) analysis was performed. Furthermore, sensory evaluation, classical microbiological analysis, Total Volatile Base-Nitrogen/TVB-N and Trimethylamine-Nitrogen/TMA-N determination were also carried out. Shelf-life of thawed cuttlefish (Sepia officinalis) stored at 2degreeC determined by sensory evaluation was 4 days. Aerobic Plate Counts (APC) reached the levels of 6.6 log cfu/g. The initial and final population of all spoilage microorganisms enumerated with selective media was under detectable levels with the exception of Pseudomonas. Based on 16S NGS analysis, Psychrobacter were the dominants among others, e.g. Pseudomonas, Shewanella, Comamonas, Carnobacterium, Vagococcus, of the initial microbiota. Psychrobacter was also the dominant microorganisms of the spoiled cuttlefish. TVB-N and TMA-N increased considerably only at the late stages of storage. A plethora of VOCs were produced and some exhibited an increasing profile throughout storage, making them promising molecules as freshness indicators in contrast to TVB-N and TMA-N. The application of next generation sequencing revealed the microbiota that escapes the classic microbiological methodologies, showing that other microorganisms different from those determined on selective culture media might be the main cause of microbiological spoilage.

Copyright © 2018. Published by Elsevier Ltd.

Version ID

1

Record Owner

From MEDLINE, a database of the U.S. National Library of Medicine.

Status

MEDLINE

Authors Full Name

Parlapani, F F; Michailidou, S; Anagnostopoulos, D A; Sakellariou, A K; Pasentsis, K; Psomopoulos, F; Argiriou, A; Haroutounian, S A; Boziaris, I S.

Institution

Parlapani, F F. Lab. Marketing and Technology of Aquatic Products and Foods, Dept. of Ichthyology and Aquatic Environment, School of Agricultural Sciences, University of Thessaly, Fitokou Street, 38446, N. Ionia, Volos, Greece. Michailidou, S. Institute of Applied Biosciences, Centre for Research and Technology Hellas (CERTH), 57001, Thessaloniki, Greece.

Anagnostopoulos, D A. Lab. Marketing and Technology of Aquatic Products and Foods, Dept. of Ichthyology and Aquatic Environment, School of Agricultural Sciences, University of Thessaly, Fitokou Street, 38446, N. Ionia, Volos, Greece.

Sakellariou, A K. Lab. Marketing and Technology of Aquatic Products and Foods, Dept. of Ichthyology and Aquatic Environment, School of Agricultural Sciences, University of Thessaly, Fitokou Street, 38446, N. Ionia, Volos, Greece.

Pasentsis, K. Institute of Applied Biosciences, Centre for Research and Technology Hellas (CERTH), 57001, Thessaloniki, Greece.

Psomopoulos, F. Institute of Applied Biosciences, Centre for Research and Technology Hellas (CERTH), 57001, Thessaloniki, Greece.

Argiriou, A. Institute of Applied Biosciences, Centre for Research and Technology Hellas (CERTH), 57001, Thessaloniki, Greece.

Haroutounian, S A. Department of Animal Science and Aquaculture, Agricultural University of Athens, Iera Odos 75, 118 55, Athens, Greece.

Boziaris, I S. Lab. Marketing and Technology of Aquatic Products and Foods, Dept. of Ichthyology and Aquatic Environment, School of Agricultural Sciences, University of Thessaly, Fitokou Street, 38446, N. Ionia, Volos, Greece. Electronic address: boziaris@uth.gr.

MeSH Heading

Animals. Bacteria/cl [Classification]. Bacteria/ge [Genetics]. *Bacteria/gd [Growth & Development]. *Bacteria/ip [Isolation & Purification]. Cold Temperature. Food Contamination/an [Analysis]. Food Storage. High-Throughput Nucleotide Sequencing. Humans. Microbiota. *Seafood/mi [Microbiology]. *Sepia/mi [Microbiology]. Smell. Taste. *Volatile Organic Compounds/ch [Chemistry]. Volatile Organic Compounds/me [Metabolism].

Keyword Heading

16S rRNA Cephalopods

Cuttlefish

Next generation sequencing (NGS)

Spoilage microbiota

Volatile organic compounds (VOCs).

Registry Number/Name of Substance

0 (Volatile Organic Compounds).

Year of Publication

2018

Link to the Ovid Full Text or citation:

[Click here for full text options](https://libaccess.mcmaster.ca/login?url=http://ovidsp.ovid.com/ovidweb.cgi?T=JS&CSC=Y&NEWS=N&PAGE=fulltext&D=med15&AN=30166182)

Link to the External Link Resolver:

[SFX](http://sfx.scholarsportal.info/mcmaster?sid=OVID:medline&id=pmid:30166182&id=doi:10.1016%2Fj.fm.2018.08.004&issn=0740-0020&isbn=&volume=76&issue=&spage=518&pages=518-525&date=2018&title=Food+Microbiology&atitle=Microbial+spoilage+investigation+of+thawed+common+cuttlefish+(Sepia+officinalis)+stored+at+2degreeC+using+next+generation+sequencing+and+volatilome+analysis.&aulast=Parlapani&pid=<author>Parlapani+FF%3BMichailidou+S%3BAnagnostopoulos+DA%3BSakellariou+AK%3BPasentsis+K%3BPsomopoulos+F%3BArgiriou+A%3BHaroutounian+SA%3BBoziaris+IS<%2Fauthor><AN>30166182<%2FAN><DT>Journal+Article<%2FDT>)

17.

Bacterial DNA detected on pathologically changed heart valves using 16S rRNA gene amplification.

Chalupova M; Skalova A; Hajek T; Geigerova L; Kralova D; Liska P; Hecova H; Molacek J; Hrabak J.

Folia Microbiologica. 63(6):707-711, 2018 Nov.

[Journal Article]

UI: 29786766

Nowadays, dental diseases are one of the most common illnesses in the world. Some of them can lead to translocation of oral bacteria to the bloodstream causing intermittent bacteraemia. Therefore, a potential association between oral infection and cardiovascular diseases has been discussed in recent years as a result of adhesion of oral microbes to the heart valves. The aim of this study was to detect oral bacteria on pathologically changed heart valves not caused by infective endocarditis. In the study, patients with pathologically changed heart valves were involved. Samples of heart valves removed during heart valve replacement surgery were cut into two parts. One aliquot was cultivated aerobically and anaerobically. Bacterial DNA was extracted using Ultra-Deep Microbiome Prep (Molzym GmbH, Bremen, Germany) followed by a 16S rRNA gene PCR amplification using Mastermix 16S Complete kit (Molzym GmbH, Bremen, Germany). Positive PCR products were sequenced and the sequences were analyzed using BLAST database ( <http://www.ncbi.nlm.nih/BLAST> ). During the study period, 41 samples were processed. Bacterial DNA of the following bacteria was detected in 21 samples: Cutibacterium acnes (formerly Propionibacterium acnes) (n = 11; 52.38% of patients with positive bacterial DNA detection), Staphylococcus sp. (n = 9; 42.86%), Streptococcus sp. (n = 1; 4.76%), Streptococcus sanguinis (n = 4; 19.05%), Streptococcus oralis (n = 1; 4.76%), Carnobacterium sp. (n = 1; 4.76%), Bacillus sp. (n = 2; 9.52%), and Bergeyella sp. (n = 1; 4.76%). In nine samples, multiple bacteria were found. Our results showed significant appearance of bacteria on pathologically changed heart valves in patients with no symptoms of infective endocarditis.

Version ID

1

Record Owner

From MEDLINE, a database of the U.S. National Library of Medicine.

Status

MEDLINE

Author NameID

Chalupova, Miroslava; ORCID: <http://orcid.org/0000-0002-7618-3582>

Authors Full Name

Chalupova, Miroslava; Skalova, Anna; Hajek, Tomas; Geigerova, Lenka; Kralova, Dana; Liska, Pavel; Hecova, Hana; Molacek, Jiri; Hrabak, Jaroslav.

Institution

Chalupova, Miroslava. Department of Stomatology, Faculty of Medicine and University Hospital in Pilsen, Charles University, alej Svobody 80, 304 60, Plzen-Lochotin, Czech Republic. chalupova.mirka@gmail.com. Chalupova, Miroslava. Biomedical Center, Faculty of Medicine in Pilsen, Charles University, Plzen, Czech Republic. chalupova.mirka@gmail.com.

Skalova, Anna. Biomedical Center, Faculty of Medicine in Pilsen, Charles University, Plzen, Czech Republic.

Skalova, Anna. Department of Microbiology, Faculty of Medicine and University Hospital in Pilsen, Charles University, Plzen, Czech Republic.

Hajek, Tomas. Department of Cardiac Surgery, University Hospital in Pilsen, Charles University, Plzen, Czech Republic.

Geigerova, Lenka. Department of Microbiology, Faculty of Medicine and University Hospital in Pilsen, Charles University, Plzen, Czech Republic.

Kralova, Dana. Biomedical Center, Faculty of Medicine in Pilsen, Charles University, Plzen, Czech Republic.

Kralova, Dana. Department of Microbiology, Faculty of Medicine and University Hospital in Pilsen, Charles University, Plzen, Czech Republic.

Liska, Pavel. Czech Statistical Office, Prague, Czech Republic.

Hecova, Hana. Department of Stomatology, Faculty of Medicine and University Hospital in Pilsen, Charles University, alej Svobody 80, 304 60, Plzen-Lochotin, Czech Republic.

Molacek, Jiri. Department of Surgery, Faculty of Medicine and University Hospital in Pilsen, Charles University, Plzen, Czech Republic.

Hrabak, Jaroslav. Biomedical Center, Faculty of Medicine in Pilsen, Charles University, Plzen, Czech Republic.

Hrabak, Jaroslav. Department of Microbiology, Faculty of Medicine and University Hospital in Pilsen, Charles University, Plzen, Czech Republic.

MeSH Heading

Aged. Aged, 80 and over. Bacteria/cl [Classification]. Bacteria/ge [Genetics]. *DNA, Bacterial. *Endocarditis, Bacterial/mi [Microbiology]. Endocarditis, Bacterial/mo [Mortality]. Endocarditis, Bacterial/pa [Pathology]. Endocarditis, Bacterial/th [Therapy]. Female. *Gene Amplification. Heart Valve Prosthesis Implantation/mt [Methods]. *Heart Valves/mi [Microbiology]. Heart Valves/pa [Pathology]. Humans. Male. Middle Aged. Polymerase Chain Reaction. *RNA, Ribosomal, 16S. Sequence Analysis, DNA.

Registry Number/Name of Substance

0 (DNA, Bacterial). 0 (RNA, Ribosomal, 16S).

Year of Publication

2018

Link to the Ovid Full Text or citation:

[Click here for full text options](https://libaccess.mcmaster.ca/login?url=http://ovidsp.ovid.com/ovidweb.cgi?T=JS&CSC=Y&NEWS=N&PAGE=fulltext&D=med15&AN=29786766)

Link to the External Link Resolver:

[SFX](http://sfx.scholarsportal.info/mcmaster?sid=OVID:medline&id=pmid:29786766&id=doi:10.1007%2Fs12223-018-0611-6&issn=0015-5632&isbn=&volume=63&issue=6&spage=707&pages=707-711&date=2018&title=Folia+Microbiologica&atitle=Bacterial+DNA+detected+on+pathologically+changed+heart+valves+using+16S+rRNA+gene+amplification.&aulast=Chalupova&pid=<author>Chalupova+M%3BSkalova+A%3BHajek+T%3BGeigerova+L%3BKralova+D%3BLiska+P%3BHecova+H%3BMolacek+J%3BHrabak+J<%2Fauthor><AN>29786766<%2FAN><DT>Journal+Article<%2FDT>)

18.

The influence of probiotic supplementation in broiler chickens on population and carcass contamination with Campylobacter spp. - Field study.

Smialek M; Burchardt S; Koncicki A.

Research in Veterinary Science. 118:312-316, 2018 Jun.

[Journal Article]

UI: 29567598

Campylobacter spp. is a food-borne pathogen occurring all over the world. According to European Food Safety Authority, in Europe, in 2015 the number of recorded and confirmed cases of Campylobacter spp. infections in humans has reached approximately 230,000. Poultry and poultry meat are considered to be the main sources of human infection, which triggers the discussion about the possibility of imposing obligatory control of Campylobacter spp. population at the level of primary poultry production. Recently, the use of probiotics in poultry is considered as a very promising alternative that could reduce infection rate in broiler chickens with Campylobacter spp. Although, there were some approaches made in vivo, up to date, there were no studies that would evaluate those issues under field conditions. A study was carried out in order to determine the feasibility of reducing infection rate in broiler chickens with Campylobacter spp. raised at a commercial farm, by the addition of multispecies probiotic (Lavipan, JHJ, Poland) that composed of Lactococcus lactis, Carnobacterium divergens, Lactobacillus casei, Lactobacillus plantarum and Saccharomyces cerevisae to the feed. Results of our study indicate that probiotic (Lavipan) added to a feed for broiler chickens was capable to reduce the extent of Campylobacter spp. invasion in the gastrointestinal tract of birds and, resultantly, to diminish contamination level in bird environment, which eventually contributed to the improved hygienic parameters of analyzed poultry carcasses. Additionally, this probiotic displayed promising immunomodulatory properties that may improve the effectiveness of the specific prophylaxis program applied in a flock of broiler chickens.

Copyright © 2018 The Authors. Published by Elsevier Ltd.. All rights reserved.

Version ID

1

Record Owner

From MEDLINE, a database of the U.S. National Library of Medicine.

Status

MEDLINE

Authors Full Name

Smialek, Marcin; Burchardt, Szymon; Koncicki, Andrzej.

Institution

Smialek, Marcin. Department of Poultry Diseases, University of Warmia and Mazury, ul. Oczapowskiego 13/13, 10-719 Olsztyn, Poland. Electronic address: marcin.smialek@uwm.edu.pl. Burchardt, Szymon. JHJ Sp. Z.O.O., Nowa Wies 11, 63-308 Gizalki, Poland. Electronic address: szymon.burchardt@jhj.pl.

Koncicki, Andrzej. Department of Poultry Diseases, University of Warmia and Mazury, ul. Oczapowskiego 13/13, 10-719 Olsztyn, Poland. Electronic address: konciki@uwm.edu.pl.

MeSH Heading

Animals. Campylobacter. Campylobacter Infections/ep [Epidemiology]. Campylobacter Infections/pc [Prevention & Control]. Campylobacter Infections/tm [Transmission]. *Campylobacter Infections/ve [Veterinary]. *Chickens. *Food Contamination. Humans. *Poultry Diseases/ep [Epidemiology]. Poultry Diseases/pc [Prevention & Control]. Poultry Diseases/tm [Transmission]. *Probiotics. Zoonoses/mi [Microbiology].

Keyword Heading

Broiler chickens Campylobacter spp.

Humoral immunity

Probiotic supplementation.

Year of Publication

2018

Link to the Ovid Full Text or citation:

[Click here for full text options](https://libaccess.mcmaster.ca/login?url=http://ovidsp.ovid.com/ovidweb.cgi?T=JS&CSC=Y&NEWS=N&PAGE=fulltext&D=med15&AN=29567598)

Link to the External Link Resolver:

[SFX](http://sfx.scholarsportal.info/mcmaster?sid=OVID:medline&id=pmid:29567598&id=doi:10.1016%2Fj.rvsc.2018.03.009&issn=0034-5288&isbn=&volume=118&issue=&spage=312&pages=312-316&date=2018&title=Research+in+Veterinary+Science&atitle=The+influence+of+probiotic+supplementation+in+broiler+chickens+on+population+and+carcass+contamination+with+Campylobacter+spp.+-+Field+study.&aulast=Smialek&pid=<author>Smialek+M%3BBurchardt+S%3BKoncicki+A<%2Fauthor><AN>29567598<%2FAN><DT>Journal+Article<%2FDT>)

19.

Characterization of the spoilage potential of pure and mixed cultures of bacterial species isolated from tropical yellowfin tuna (Thunnus albacares).

Silbande A; Cornet J; Cardinal M; Chevalier F; Rochefort K; Smith-Ravin J; Adenet S; Leroi F.

Journal of Applied Microbiology. 124(2):559-571, 2018 Feb.

[Journal Article]

UI: 29222941

AIM: The spoilage potential of 28 bacterial strains isolated from spoiled raw yellowfin tuna was evaluated.

METHODS AND RESULTS: Bacterial species were inoculated in irradiated tuna matrix. Chemical changes, bacterial growth and sensory quality were monitored during aerobic storage at 8degreeC. Pseudomonas spp., Enterobacter spp. and Escherichia hermanii had no spoiling effect. Brochothrix thermosphacta and Carnobacterium divergens/maltaromaticum developed moderate unpleasant odours. Hafnia paralvei and Serratia spp. released strong off-odours (pyrrolidine, sulphur/cabbage). No bacterial group (except H. paralvei) combined with Pseudomonas spp. deteriorated the sensory quality of tuna. When C. divergens/maltaromaticum was associated with H. paralvei or B. thermosphacta, the odour is close to the naturally contaminated tuna stored on the same conditions. The pH, total volatile basic nitrogen (TVBN) and trimethylamine (TMA) were not correlated with the spoilage.

CONCLUSIONS: The bacterial species had a different impact on the sensory quality of the fish. The bacterial interactions lead to an enhancement or an inhibition of the spoilage potential and the bacterial growth.

SIGNIFICANCE AND IMPACT OF STUDY: The specific spoilage organism (SSO) appears to be an association of lactic acid bacteria (LAB) with Enterobacteriaceae or B. thermosphacta. Pseudomonas, often dominant at the sensory rejection time, is not a good quality indicator.

Copyright © 2017 The Society for Applied Microbiology.

Version ID

1

Record Owner

From MEDLINE, a database of the U.S. National Library of Medicine.

Status

MEDLINE

Authors Full Name

Silbande, A; Cornet, J; Cardinal, M; Chevalier, F; Rochefort, K; Smith-Ravin, J; Adenet, S; Leroi, F.

Institution

Silbande, A. Laboratoire Ecosystemes Microbiens et Molecules Marines pour les Biotechnologies (EM3B), Ifremer, Nantes, France. Silbande, A. Pole Agroalimentaire Regional de Martinique (PARM), Ifremer, Lamentin, Martinique.

Silbande, A. Departement Scientifique Inter facultaire (DSI), EA929 AIHP-GEODE (groupe BIOSPHERES), Universite des Antilles, Schoelcher, Martinique.

Cornet, J. Laboratoire Ecosystemes Microbiens et Molecules Marines pour les Biotechnologies (EM3B), Ifremer, Nantes, France.

Cardinal, M. Laboratoire Ecosystemes Microbiens et Molecules Marines pour les Biotechnologies (EM3B), Ifremer, Nantes, France.

Chevalier, F. Laboratoire Ecosystemes Microbiens et Molecules Marines pour les Biotechnologies (EM3B), Ifremer, Nantes, France.

Rochefort, K. Pole Agroalimentaire Regional de Martinique (PARM), Ifremer, Lamentin, Martinique.

Smith-Ravin, J. Departement Scientifique Inter facultaire (DSI), EA929 AIHP-GEODE (groupe BIOSPHERES), Universite des Antilles, Schoelcher, Martinique.

Adenet, S. Pole Agroalimentaire Regional de Martinique (PARM), Ifremer, Lamentin, Martinique.

Leroi, F. Laboratoire Ecosystemes Microbiens et Molecules Marines pour les Biotechnologies (EM3B), Ifremer, Nantes, France.

MeSH Heading

Animals. Bacteria/cl [Classification]. Bacteria/ge [Genetics]. *Bacteria/ip [Isolation & Purification]. *Fishes/mi [Microbiology]. Food Contamination/an [Analysis]. Food Microbiology. Humans. Odorants/an [Analysis]. Taste. *Tuna/mi [Microbiology].

Keyword Heading

Pseudomonas bacterial interactions

bacterial species

fish

sensory quality

spoilage potential

tropical

tuna.

Year of Publication

2018

Link to the Ovid Full Text or citation:

[Click here for full text options](https://libaccess.mcmaster.ca/login?url=http://ovidsp.ovid.com/ovidweb.cgi?T=JS&CSC=Y&NEWS=N&PAGE=fulltext&D=med15&AN=29222941)

Link to the External Link Resolver:

[SFX](http://sfx.scholarsportal.info/mcmaster?sid=OVID:medline&id=pmid:29222941&id=doi:10.1111%2Fjam.13663&issn=1364-5072&isbn=&volume=124&issue=2&spage=559&pages=559-571&date=2018&title=Journal+of+Applied+Microbiology&atitle=Characterization+of+the+spoilage+potential+of+pure+and+mixed+cultures+of+bacterial+species+isolated+from+tropical+yellowfin+tuna+(Thunnus+albacares).&aulast=Silbande&pid=<author>Silbande+A%3BCornet+J%3BCardinal+M%3BChevalier+F%3BRochefort+K%3BSmith-Ravin+J%3BAdenet+S%3BLeroi+F<%2Fauthor><AN>29222941<%2FAN><DT>Journal+Article<%2FDT>)

20.

Review - Lactic acid bacteria in traditional fermented Asian foods. [Review]

Azam M; Mohsin M; Ijaz H; Tulain UR; Ashraf MA; Fayyaz A; Abadeen Z; Kamran Q.

Pakistan Journal of Pharmaceutical Sciences. 30(5):1803-1814, 2017 Sep.

[Journal Article. Review]

UI: 29084705

Lactic acid bacteria play vital roles in various fermented foods in Asia. This paper reviews many types of the world's lactic acid fermented foods and discusses the beneficial effects of lactic acid fermentation of food. The lactic acid bacteria associated with foods now include species of the genera Carnobacterium, Enterococcus, Lactobacillus, Lactococcus, Leuconostoc, Oenococcus, Pediococcus, Streptococcus, Tetragenococcus, Vagococcus and Weissella. Lactic acid bacteria (LAB) are involved in many fermentation processes of Asian traditional foods, demonstrating their profound effects on improving food quality and food safety. During the past few decades' interest has arisen in the use of the varied antagonistic activities of LAB to extent the shelf-life of protein-rich products such as meats and fish. This review article outlines the main types of LAB fermentation as well as their typical fermented foods such as idli, kishk, sauerkraut, koumiss, Suan-tsai, stinky tofu, Chinese sausage and kefir. The roles of LAB and the reasons for their common presence are also discussed.

Version ID

1

Record Owner

From MEDLINE, a database of the U.S. National Library of Medicine.

Status

MEDLINE

Authors Full Name

Azam, Mariya; Mohsin, Mashkoor; Ijaz, Hira; Tulain, Ume Ruqia; Ashraf, Muhammad Adnan; Fayyaz, Ahad; Abadeen, Zainul; Kamran, Qindeel.

Institution

Azam, Mariya. Institute of Microbiology, University of Agriculture, Faisalabad, Pakistan. Mohsin, Mashkoor. Institute of Microbiology, University of Agriculture, Faisalabad, Pakistan.

Ijaz, Hira. Faculty of Pharmacy, University of Sargodha, Punjab, Pakistan.

Tulain, Ume Ruqia. Faculty of Pharmacy, University of Sargodha, Punjab, Pakistan.

Ashraf, Muhammad Adnan. Faculty of Pharmacy, University of Sargodha, Punjab, Pakistan.

Fayyaz, Ahad. Department of Pathology, University of Agriculture, Faisalabad, Pakistan.

Abadeen, Zainul. Department of Pathology, University of Agriculture, Faisalabad, Pakistan.

Kamran, Qindeel. Institute of Pharmacy, Physiology and Pharmacology, University of Agriculture, Faisalabad, Pakistan.

MeSH Heading

Asia. *Asian Continental Ancestry Group. Consumer Product Safety. Diet/ae [Adverse Effects]. *Diet/eh [Ethnology]. *Fermentation. Fermented Foods and Beverages/ae [Adverse Effects]. *Fermented Foods and Beverages/mi [Microbiology]. *Food Microbiology/mt [Methods]. Food Preservation/mt [Methods]. Food Quality. Food Safety. Humans. *Lactic Acid/me [Metabolism]. *Lactobacillales/me [Metabolism]. Nutritive Value.

Registry Number/Name of Substance

33X04XA5AT (Lactic Acid).

Year of Publication

2017

Link to the Ovid Full Text or citation:

[Click here for full text options](https://libaccess.mcmaster.ca/login?url=http://ovidsp.ovid.com/ovidweb.cgi?T=JS&CSC=Y&NEWS=N&PAGE=fulltext&D=med14&AN=29084705)

Link to the External Link Resolver:

[SFX](http://sfx.scholarsportal.info/mcmaster?sid=OVID:medline&id=pmid:29084705&id=doi:&issn=1011-601X&isbn=&volume=30&issue=5&spage=1803&pages=1803-1814&date=2017&title=Pakistan+Journal+of+Pharmaceutical+Sciences&atitle=Review+-+Lactic+acid+bacteria+in+traditional+fermented+Asian+foods.&aulast=Azam&pid=<author>Azam+M%3BMohsin+M%3BIjaz+H%3BTulain+UR%3BAshraf+MA%3BFayyaz+A%3BAbadeen+Z%3BKamran+Q<%2Fauthor><AN>29084705<%2FAN><DT>Journal+Article<%2FDT>)

21.

Protective Effect of Carnobacterium spp. against Listeria monocytogenes during Host Cell Invasion Using In vitro HT29 Model.

Pilchova T; Pilet MF; Cappelier JM; Pazlarova J; Tresse O.

Frontiers in Cellular & Infection Microbiology. 6:88, 2016.

[Journal Article. Research Support, Non-U.S. Gov't]

UI: 27617232

The pathogenesis of listeriosis results mainly from the ability of Listeria monocytogenes to attach, invade, replicate and survive within various cell types in mammalian tissues. In this work, the effect of two bacteriocin-producing Carnobacterium (C. divergens V41 and C. maltaromaticum V1) and three non-bacteriocinogenic strains: (C. divergens V41C9, C. divergens 2763, and C. maltaromaticum 2762) was investigated on the reduction of L. monocytogenes Scott A plaque-forming during human infection using the HT-29 in vitro model. All Carnobacteria tested resulted in a reduction in the epithelial cell invasion caused by L. monocytogenes Scott A. To understand better the mechanism underlying the level of L. monocytogenes infection inhibition by Carnobacteria, infection assays from various pretreatments of Carnobacteria were assessed. The results revealed the influence of bacteriocin production combined with a passive mechanism of mammalian cell monolayers protection by Carnobacteria. These initial results showing a reduction in L. monocytogenes virulence on epithelial cells by Carnobacteria would be worthwhile analyzing further as a promising probiotic tool for human health.

Version ID

1

Record Owner

From MEDLINE, a database of the U.S. National Library of Medicine.

Status

MEDLINE

Authors Full Name

Pilchova, Tereza; Pilet, Marie-France; Cappelier, Jean-Michel; Pazlarova, Jarmila; Tresse, Odile.

Institution

Pilchova, Tereza. Department of Biochemistry and Microbiology, Faculty of Food and Biochemical Technology, University of Chemistry and TechnologyPrague, Czech Republic; UMR1014 SECALIM, INRA, OnirisNantes, France. Pilet, Marie-France. UMR1014 SECALIM, INRA, Oniris Nantes, France.

Cappelier, Jean-Michel. UMR1014 SECALIM, INRA, Oniris Nantes, France.

Pazlarova, Jarmila. Department of Biochemistry and Microbiology, Faculty of Food and Biochemical Technology, University of Chemistry and Technology Prague, Czech Republic.

Tresse, Odile. UMR1014 SECALIM, INRA, Oniris Nantes, France.

MeSH Heading

*Antibiosis. *Carnobacterium/ph [Physiology]. *Endocytosis. *Epithelial Cells/mi [Microbiology]. HT29 Cells. Humans. *Listeria monocytogenes/py [Pathogenicity].

Keyword Heading

*Carnobacterium divergens *Carnobacterium maltaromaticum

*HT29

*bacteriocin

*foodborne pathogens

*mucus layer.

Year of Publication

2016

Link to the Ovid Full Text or citation:

[Click here for full text options](https://libaccess.mcmaster.ca/login?url=http://ovidsp.ovid.com/ovidweb.cgi?T=JS&CSC=Y&NEWS=N&PAGE=fulltext&D=med13&AN=27617232)

Link to the External Link Resolver:

[SFX](http://sfx.scholarsportal.info/mcmaster?sid=OVID:medline&id=pmid:27617232&id=doi:10.3389%2Ffcimb.2016.00088&issn=2235-2988&isbn=&volume=6&issue=&spage=88&pages=88&date=2016&title=Frontiers+in+Cellular+%26+Infection+Microbiology&atitle=Protective+Effect+of+Carnobacterium+spp.+against+Listeria+monocytogenes+during+Host+Cell+Invasion+Using+In+vitro+HT29+Model.&aulast=Pilchova&pid=<author>Pilchova+T%3BPilet+MF%3BCappelier+JM%3BPazlarova+J%3BTresse+O<%2Fauthor><AN>27617232<%2FAN><DT>Journal+Article<%2FDT>)

22.

Carnobacterium divergens Bacteremia in woman.

Smati M; Palacios C; Cohen Y; Mechai F; Tankovic J; Le Fleche-Mateos A; Picard B; Gonzalez F.

Emerging Infectious Diseases. 21(6):1081-2, 2015 Jun.

[Case Reports. Letter]

UI: 25988484

Version ID

1

Record Owner

From MEDLINE, a database of the U.S. National Library of Medicine.

Status

MEDLINE

Authors Full Name

Smati, Mounira; Palacios, Christia; Cohen, Yves; Mechai, Frederic; Tankovic, Jacques; Le Fleche-Mateos, Anne; Picard, Bertrand; Gonzalez, Frederic.

MeSH Heading

Anti-Bacterial Agents/tu [Therapeutic Use]. *Bacteremia. Bacterial Typing Techniques. Carnobacterium/ge [Genetics]. *Carnobacterium/ip [Isolation & Purification]. Female. *Gram-Positive Bacterial Infections/di [Diagnosis]. Gram-Positive Bacterial Infections/dt [Drug Therapy]. *Gram-Positive Bacterial Infections/mi [Microbiology]. Humans. Middle Aged. Treatment Outcome.

Keyword Heading

Carnobacterium divergens bacteremia

bacteria

bacterial translocation

enteral nutrition

probiotics.

Registry Number/Name of Substance

0 (Anti-Bacterial Agents).

Year of Publication

2015

Link to the Ovid Full Text or citation:

[Click here for full text options](https://libaccess.mcmaster.ca/login?url=http://ovidsp.ovid.com/ovidweb.cgi?T=JS&CSC=Y&NEWS=N&PAGE=fulltext&D=med12&AN=25988484)

Link to the External Link Resolver:

[SFX](http://sfx.scholarsportal.info/mcmaster?sid=OVID:medline&id=pmid:25988484&id=doi:10.3201%2Feid2106.141799&issn=1080-6040&isbn=&volume=21&issue=6&spage=1081&pages=1081-2&date=2015&title=Emerging+Infectious+Diseases&atitle=Carnobacterium+divergens+Bacteremia+in+woman.&aulast=Smati&pid=<author>Smati+M%3BPalacios+C%3BCohen+Y%3BMechai+F%3BTankovic+J%3BLe+Fleche-Mateos+A%3BPicard+B%3BGonzalez+F<%2Fauthor><AN>25988484<%2FAN><DT>Case+Reports<%2FDT>)

23.

Lactic acid bacteria and their controversial role in fresh meat spoilage.

Pothakos V; Devlieghere F; Villani F; Bjorkroth J; Ercolini D.

Meat Science. 109:66-74, 2015 Nov.

[Journal Article]

UI: 25972087

Lactic acid bacteria (LAB) constitute a heterogeneous group that has been widely associated with fresh meat and cooked meat products. They represent a controversial cohort of microbial species that either contribute to spoilage through generation of offensive metabolites and the subsequent organoleptic downgrading of meat or serve as bioprotective agents with strains of certain species causing unperceivable or no alterations. Therefore, significant distinction among biotypes is substantiated by studies determining spoilage potential as a strain-specific trait corroborating the need to revisit the concept of spoilage.

Copyright © 2015 Elsevier Ltd. All rights reserved.

Version ID

1

Record Owner

From MEDLINE, a database of the U.S. National Library of Medicine.

Status

MEDLINE

Authors Full Name

Pothakos, Vasileios; Devlieghere, Frank; Villani, Francesco; Bjorkroth, Johanna; Ercolini, Danilo.

Institution

Pothakos, Vasileios. Laboratory of Food Microbiology and Food Preservation, Department of Food Safety and Food Quality, Member of Food2Know, Faculty of Bioscience Engineering, Ghent University, Ghent, Belgium. Devlieghere, Frank. Laboratory of Food Microbiology and Food Preservation, Department of Food Safety and Food Quality, Member of Food2Know, Faculty of Bioscience Engineering, Ghent University, Ghent, Belgium.

Villani, Francesco. Department of Agricultural Sciences, Division of Microbiology, University of Naples Federico II, Portici, Italy.

Bjorkroth, Johanna. Department of Food Hygiene and Environmental Health, University of Helsinki, Helsinki, Finland.

Ercolini, Danilo. Department of Agricultural Sciences, Division of Microbiology, University of Naples Federico II, Portici, Italy. Electronic address: ercolini@unina.it.

MeSH Heading

Animals. *Carnobacterium. *Food Microbiology. Food Packaging. Food Storage. Humans. Lactic Acid. *Lactobacillus. *Leuconostoc. *Meat/mi [Microbiology]. Meat Products/mi [Microbiology].

Keyword Heading

Carnobacterium Food packaging

Lactic acid bacteria

Leuconostoc gelidum

Meat spoilage.

Registry Number/Name of Substance

33X04XA5AT (Lactic Acid).

Year of Publication

2015

Link to the Ovid Full Text or citation:

[Click here for full text options](https://libaccess.mcmaster.ca/login?url=http://ovidsp.ovid.com/ovidweb.cgi?T=JS&CSC=Y&NEWS=N&PAGE=fulltext&D=med12&AN=25972087)

Link to the External Link Resolver:

[SFX](http://sfx.scholarsportal.info/mcmaster?sid=OVID:medline&id=pmid:25972087&id=doi:10.1016%2Fj.meatsci.2015.04.014&issn=0309-1740&isbn=&volume=109&issue=&spage=66&pages=66-74&date=2015&title=Meat+Science&atitle=Lactic+acid+bacteria+and+their+controversial+role+in+fresh+meat+spoilage.&aulast=Pothakos&pid=<author>Pothakos+V%3BDevlieghere+F%3BVillani+F%3BBjorkroth+J%3BErcolini+D<%2Fauthor><AN>25972087<%2FAN><DT>Journal+Article<%2FDT>)

24.

Microbiological changes, shelf life and identification of initial and spoilage microbiota of sea bream fillets stored under various conditions using 16S rRNA gene analysis.

Parlapani FF; Kormas KA; Boziaris IS.

Journal of the Science of Food & Agriculture. 95(12):2386-94, 2015 Sep.

[Journal Article. Research Support, Non-U.S. Gov't]

UI: 25312872

BACKGROUND: Sea bream fillets are one of the most important value-added products of the seafood market. Fresh seafood spoils mainly owing to bacterial action. In this study an exploration of initial and spoilage microbiota of sea bream fillets stored under air and commercial modified atmosphere packaging (MAP) at 0 and 5 degreeC was conducted by 16S rRNA gene sequence analysis of isolates grown on plates. Sensory evaluation and enumeration of total viable counts and spoilage microorganisms were also conducted to determine shelf life and bacterial growth respectively.

RESULTS: Different temperatures and atmospheres affected growth and synthesis of spoilage microbiota as well as shelf life. Shelf life under air at 0 and 5 degreeC was 14 and 5 days respectively, while under MAP it was 20 and 8 days respectively. Initial microbiota were dominated by Pseudomonas fluorescens, Psychrobacter and Macrococcus caseolyticus. Different temperatures and atmospheres affected the synthesis of spoilage microbiota. At the end of shelf life, different phylotypes of Pseudomonas closely related to Pseudomonas fragi were found to dominate in most cases, while Pseudomonas veronii dominated in fillets under MAP at 0 degreeC. Furthermore, in fillets under MAP at 5 degreeC, new dominant species such as Carnobacterium maltaromaticum, Carnobacterium divergens and Vagococcus fluvialis were revealed.

CONCLUSION: Different temperature and atmospheric conditions affected bacterial growth, shelf life and the synthesis of spoilage microbiota. Molecular identification revealed species and strains of microorganisms that have not been reported before for sea bream fillets stored under various conditions, thus providing valuable information regarding microbiological spoilage.

Copyright © 2014 Society of Chemical Industry.

Version ID

1

Record Owner

From MEDLINE, a database of the U.S. National Library of Medicine.

Status

MEDLINE

Authors Full Name

Parlapani, Foteini F; Kormas, Konstantinos Ar; Boziaris, Ioannis S.

Institution

Parlapani, Foteini F. Department of Ichthyology and Aquatic Environment, School of Agricultural Sciences, University of Thessaly, Fitokou Street, GR-38446 N. Ionia, Volos, Greece. Kormas, Konstantinos Ar. Department of Ichthyology and Aquatic Environment, School of Agricultural Sciences, University of Thessaly, Fitokou Street, GR-38446 N. Ionia, Volos, Greece.

Boziaris, Ioannis S. Department of Ichthyology and Aquatic Environment, School of Agricultural Sciences, University of Thessaly, Fitokou Street, GR-38446 N. Ionia, Volos, Greece.

MeSH Heading

Animals. Bacteria/ge [Genetics]. Bacteria/ip [Isolation & Purification]. Colony Count, Microbial. DNA, Bacterial/ge [Genetics]. *Fish Products/mi [Microbiology]. *Food Microbiology. *Food Preservation. Humans. Phylogeny. RNA, Ribosomal, 16S/ge [Genetics]. *Sea Bream. Temperature.

Keyword Heading

16S rRNA PCR

fish

sea bream

seafood

spoilage microbiota.

Registry Number/Name of Substance

0 (DNA, Bacterial). 0 (RNA, Ribosomal, 16S).

Year of Publication

2015

Link to the Ovid Full Text or citation:

[Click here for full text options](https://libaccess.mcmaster.ca/login?url=http://ovidsp.ovid.com/ovidweb.cgi?T=JS&CSC=Y&NEWS=N&PAGE=fulltext&D=med12&AN=25312872)

Link to the External Link Resolver:

[SFX](http://sfx.scholarsportal.info/mcmaster?sid=OVID:medline&id=pmid:25312872&id=doi:10.1002%2Fjsfa.6957&issn=0022-5142&isbn=&volume=95&issue=12&spage=2386&pages=2386-94&date=2015&title=Journal+of+the+Science+of+Food+%26+Agriculture&atitle=Microbiological+changes%2C+shelf+life+and+identification+of+initial+and+spoilage+microbiota+of+sea+bream+fillets+stored+under+various+conditions+using+16S+rRNA+gene+analysis.&aulast=Parlapani&pid=<author>Parlapani+FF%3BKormas+KA%3BBoziaris+IS<%2Fauthor><AN>25312872<%2FAN><DT>Journal+Article<%2FDT>)

25.

The shelf life of farmed turbot (Scophthalmus maximus).

Roth B; Kramer L; Skuland AV; Lovdal T; Oines S; Foss A; Imsland AK.

Journal of Food Science. 79(8):S1568-74, 2014 Aug.

[Journal Article. Research Support, Non-U.S. Gov't]

UI: 25046980

A total of 18 farmed turbot (Scophthalmus maximus) were slaughtered over 4 successive weeks in November 2012 and stored in polystyrene boxes with ice until analyzed. The fish were stored between 1 and 22 d and presented to a taste panel and further analyzed for quality index method (QIM), microbiological analysis by real-time quantitative PCR (qPCR), taste, pH, color by computer imaging, protein denaturation with differential scanner calorimeter (DSC), texture hardness, and shear force. Results show small, but significant changes in physical and visual attributes such as texture and color. No gaping was observed. Only small changes in texture were observed explained by lack of myosin denaturation. The fillets became more white and yellow during storage, whereas the major changes occurred during the 1st week. A panel evaluating QIM and taste could not distinguish major differences in appearance and taste and over 15 d storage period, but were able to quantify the age by smell. Analysis of microorganisms on the epidermis displayed growth of Carnobacterium maltaromaticum, potentially inhibiting growth of other spoilage bacteria. Fish stored for 22 d were rejected by the taste panel caused by a stale smell and taste, but not bitter or rancid. It is concluded that turbot has a shelf life of at least 16 d.

Copyright © 2014 Institute of Food Technologists R

Version ID

1

Record Owner

From MEDLINE, a database of the U.S. National Library of Medicine.

Status

MEDLINE

Authors Full Name

Roth, Bjorn; Kramer, Lene; Skuland, Aase Vorre; Lovdal, Trond; Oines, Sigurd; Foss, Atle; Imsland, Albert Kjartansson.

Institution

Roth, Bjorn. Nofima Dept. of Processing Technology, P.O. Box 8034, N-4068 Stavanger, Norway.

MeSH Heading

Animals. Calorimetry, Differential Scanning. Carnobacterium/gd [Growth & Development]. *Carnobacterium/ip [Isolation & Purification]. Color. Cooking. DNA, Bacterial/ge [Genetics]. *Flatfishes/mi [Microbiology]. Food Contamination/an [Analysis]. Food Microbiology. *Food Storage. Hardness. Humans. Hydrogen-Ion Concentration. Lactobacillus/gd [Growth & Development]. Lactobacillus/ip [Isolation & Purification]. Linear Models. Muscle, Skeletal/ch [Chemistry]. Photobacterium/gd [Growth & Development]. Photobacterium/ip [Isolation & Purification]. *Seafood/mi [Microbiology]. Sequence Analysis, DNA. Taste.

Keyword Heading

fish proteolysis

sensory analysis

shelf life

texture.

Registry Number/Name of Substance

0 (DNA, Bacterial).

Year of Publication

2014

Link to the Ovid Full Text or citation:

[Click here for full text options](https://libaccess.mcmaster.ca/login?url=http://ovidsp.ovid.com/ovidweb.cgi?T=JS&CSC=Y&NEWS=N&PAGE=fulltext&D=med11&AN=25046980)

Link to the External Link Resolver:

[SFX](http://sfx.scholarsportal.info/mcmaster?sid=OVID:medline&id=pmid:25046980&id=doi:10.1111%2F1750-3841.12541&issn=0022-1147&isbn=&volume=79&issue=8&spage=S1568&pages=S1568-74&date=2014&title=Journal+of+Food+Science&atitle=The+shelf+life+of+farmed+turbot+(Scophthalmus+maximus).&aulast=Roth&pid=<author>Roth+B%3BKramer+L%3BSkuland+AV%3BLovdal+T%3BOines+S%3BFoss+A%3BImsland+AK<%2Fauthor><AN>25046980<%2FAN><DT>Journal+Article<%2FDT>)

26.

Associations between bacterial communities of house dust and infant gut.

Konya T; Koster B; Maughan H; Escobar M; Azad MB; Guttman DS; Sears MR; Becker AB; Brook JR; Takaro TK; Kozyrskyj AL; Scott JA; CHILD Study Investigators.

Environmental Research. 131:25-30, 2014 May.

[Journal Article. Research Support, Non-U.S. Gov't]

UI: 24637181

The human gut is host to a diverse and abundant community of bacteria that influence health and disease susceptibility. This community develops in infancy, and its composition is strongly influenced by environmental factors, notably perinatal anthropogenic exposures such as delivery mode (Cesarean vs. vaginal) and feeding method (breast vs. formula); however, the built environment as a possible source of exposure has not been considered. Here we report on a preliminary investigation of the associations between bacteria in house dust and the nascent fecal microbiota from 20 subjects from the Canadian Healthy Infant Longitudinal Development (CHILD) Study using high-throughput sequence analysis of portions of the 16S rRNA gene. Despite significant differences between the dust and fecal microbiota revealed by Nonmetric Multidimensional Scaling (NMDS) analysis, permutation analysis confirmed that 14 bacterial OTUs representing the classes Actinobacteria (3), Bacilli (3), Clostridia (6) and Gammaproteobacteria (2) co-occurred at a significantly higher frequency in matched dust-stool pairs than in randomly permuted pairs, indicating an association between these dust and stool communities. These associations could indicate a role for the indoor environment in shaping the nascent gut microbiota, but future studies will be needed to confirm that our findings do not solely reflect a reverse pathway. Although pet ownership was strongly associated with the presence of certain genera in the dust for dogs (Agrococcus, Carnobacterium, Exiguobacterium, Herbaspirillum, Leifsonia and Neisseria) and cats (Escherichia), no clear patterns were observed in the NMDS-resolved stool community profiles as a function of pet ownership.

Copyright © 2014 Elsevier Inc. All rights reserved.

Version ID

1

Record Owner

From MEDLINE, a database of the U.S. National Library of Medicine.

Status

MEDLINE

Authors Full Name

Konya, T; Koster, B; Maughan, H; Escobar, M; Azad, M B; Guttman, D S; Sears, M R; Becker, A B; Brook, J R; Takaro, T K; Kozyrskyj, A L; Scott, J A; CHILD Study Investigators.

Institution

Konya, T. Division of Occupational and Environmental Health, Dalla Lana School of Public Health, University of Toronto, Canada. Koster, B. Division of Occupational and Environmental Health, Dalla Lana School of Public Health, University of Toronto, Canada.

Maughan, H. Department of Cell and Systems Biology, University of Toronto, Canada.

Escobar, M. Division of Occupational and Environmental Health, Dalla Lana School of Public Health, University of Toronto, Canada.

Azad, M B. Department of Pediatrics, University of Alberta, Canada.

Guttman, D S. Department of Cell and Systems Biology, University of Toronto, Canada.

Sears, M R. Department of Medicine, McMaster University, Canada.

Becker, A B. University of Manitoba, Canada.

Brook, J R. Division of Occupational and Environmental Health, Dalla Lana School of Public Health, University of Toronto, Canada; Environment Canada, Canada.

Takaro, T K. Faculty of Health Science, Simon Fraser University, Canada.

Kozyrskyj, A L. Department of Pediatrics, University of Alberta, Canada.

Scott, J A. Division of Occupational and Environmental Health, Dalla Lana School of Public Health, University of Toronto, Canada. Electronic address: james.scott@utoronto.ca.

MeSH Heading

Animals. Cats. Dogs. *Dust. *Feces/mi [Microbiology]. Humans. Infant. Longitudinal Studies. *Microbial Consortia. Pets.

Keyword Heading

Built environment Child health

Gut microbiota.

Registry Number/Name of Substance

0 (Dust).

Year of Publication

2014

Link to the Ovid Full Text or citation:

[Click here for full text options](https://libaccess.mcmaster.ca/login?url=http://ovidsp.ovid.com/ovidweb.cgi?T=JS&CSC=Y&NEWS=N&PAGE=fulltext&D=med11&AN=24637181)

Link to the External Link Resolver:

[SFX](http://sfx.scholarsportal.info/mcmaster?sid=OVID:medline&id=pmid:24637181&id=doi:10.1016%2Fj.envres.2014.02.005&issn=0013-9351&isbn=&volume=131&issue=&spage=25&pages=25-30&date=2014&title=Environmental+Research&atitle=Associations+between+bacterial+communities+of+house+dust+and+infant+gut.&aulast=Konya&pid=<author>Konya+T%3BKoster+B%3BMaughan+H%3BEscobar+M%3BAzad+MB%3BGuttman+DS%3BSears+MR%3BBecker+AB%3BBrook+JR%3BTakaro+TK%3BKozyrskyj+AL%3BScott+JA%3BCHILD+Study+Investigators<%2Fauthor><AN>24637181<%2FAN><DT>Journal+Article<%2FDT>)

27.

In vivo study of spoilage bacteria on polyphenoloxidase activity and melanosis of modified atmosphere packaged Pacific white shrimp.

Qian YF; Xie J; Yang SP; Wu WH; Xiong Q; Gao ZL.

Food Chemistry. 155:126-31, 2014 Jul 15.

[Journal Article. Research Support, Non-U.S. Gov't]

UI: 24594164

This study investigated the effect of the three spoilage bacteria (Carnobacterium maltaromaticum, Shewanella putrefaciens and Aeromonas salmonicida) on the development of melanosis by inoculating the bacteria on modified atmosphere packaged shrimp. The three bacteria, which inoculated at about 5 log cfu/g, proliferated to a maximum level of 7.49, 6.86 and 6.89 log cfu/g, respectively at the end of storage. In regards to the effect of bacteria on melanosis, it was found that C. maltaromaticum did not display a significant effect on PPO activity and melanosis, and A. salmonicida showed an inhibitory effect on PPO activity with an activity value of less than 1.5 Units/ml. However, the PPO activity of shrimp inoculated S. putrefaciens was about one time higher than other samples and greater melanosis was displayed in the first 48 h. As melanosis can cause sensory quality loss, the growth of S. putrefaciens should be limited.

Copyright © 2014 Elsevier Ltd. All rights reserved.

Version ID

1

Record Owner

From MEDLINE, a database of the U.S. National Library of Medicine.

Status

MEDLINE

Authors Full Name

Qian, Yun-Fang; Xie, Jing; Yang, Sheng-Ping; Wu, Wen-Hui; Xiong, Qing; Gao, Zhi-Li.

Institution

Qian, Yun-Fang. Shanghai Engineering Research Center of Aquatic Product Processing & Preservation, Shanghai Ocean University, Shanghai 201306, PR China; College of Food Science & Technology, Shanghai Ocean University, Shanghai 201306, PR China. Xie, Jing. Shanghai Engineering Research Center of Aquatic Product Processing & Preservation, Shanghai Ocean University, Shanghai 201306, PR China; College of Food Science & Technology, Shanghai Ocean University, Shanghai 201306, PR China. Electronic address: jxie@shou.edu.cn.

Yang, Sheng-Ping. Shanghai Engineering Research Center of Aquatic Product Processing & Preservation, Shanghai Ocean University, Shanghai 201306, PR China; College of Food Science & Technology, Shanghai Ocean University, Shanghai 201306, PR China.

Wu, Wen-Hui. Shanghai Engineering Research Center of Aquatic Product Processing & Preservation, Shanghai Ocean University, Shanghai 201306, PR China; College of Food Science & Technology, Shanghai Ocean University, Shanghai 201306, PR China.

Xiong, Qing. Shanghai Engineering Research Center of Aquatic Product Processing & Preservation, Shanghai Ocean University, Shanghai 201306, PR China; College of Food Science & Technology, Shanghai Ocean University, Shanghai 201306, PR China.

Gao, Zhi-Li. Shanghai Engineering Research Center of Aquatic Product Processing & Preservation, Shanghai Ocean University, Shanghai 201306, PR China; College of Food Science & Technology, Shanghai Ocean University, Shanghai 201306, PR China.

MeSH Heading

Adult. *Aeromonas salmonicida/gd [Growth & Development]. Animals. *Arthropod Proteins/me [Metabolism]. *Carnobacterium/gd [Growth & Development]. *Catechol Oxidase/me [Metabolism]. Color. Female. Food Packaging. Humans. Male. *Penaeidae/en [Enzymology]. Penaeidae/me [Metabolism]. Penaeidae/mi [Microbiology]. *Pigments, Biological/me [Metabolism]. *Shellfish/an [Analysis]. Shellfish/mi [Microbiology]. *Shewanella putrefaciens/gd [Growth & Development]. Taste.

Keyword Heading

Aeromonas salmonicida Carnobacterium maltaromaticum

Melanosis

Modified atmosphere packaging

Polyphenoloxidase

Shewanella putrefaciens

Shrimp.

Registry Number/Name of Substance

0 (Arthropod Proteins). 0 (Pigments, Biological). EC 1-10-3-1 (Catechol Oxidase).

Year of Publication

2014

Link to the Ovid Full Text or citation:

[Click here for full text options](https://libaccess.mcmaster.ca/login?url=http://ovidsp.ovid.com/ovidweb.cgi?T=JS&CSC=Y&NEWS=N&PAGE=fulltext&D=med11&AN=24594164)

Link to the External Link Resolver:

[SFX](http://sfx.scholarsportal.info/mcmaster?sid=OVID:medline&id=pmid:24594164&id=doi:10.1016%2Fj.foodchem.2014.01.031&issn=0308-8146&isbn=&volume=155&issue=&spage=126&pages=126-31&date=2014&title=Food+Chemistry&atitle=In+vivo+study+of+spoilage+bacteria+on+polyphenoloxidase+activity+and+melanosis+of+modified+atmosphere+packaged+Pacific+white+shrimp.&aulast=Qian&pid=<author>Qian+YF%3BXie+J%3BYang+SP%3BWu+WH%3BXiong+Q%3BGao+ZL<%2Fauthor><AN>24594164<%2FAN><DT>Journal+Article<%2FDT>)

28.

Evaluation of the spoilage potential of bacteria isolated from spoiled cooked whole tropical shrimp (Penaeus vannamei) stored under modified atmosphere packaging.

Mace S; Cardinal M; Jaffres E; Cornet J; Lalanne V; Chevalier F; Serot T; Pilet MF; Dousset X; Joffraud JJ.

Food Microbiology. 40:9-17, 2014 Jun.

[Evaluation Study. Journal Article. Research Support, Non-U.S. Gov't]

UI: 24549192

The spoilage potential of isolates belonging to five bacterial groups/species (Shewanella baltica, Carnobacterium maltaromaticum, Aeromonas salmonicida, Vibrio sp., "other Gamma-Proteobacteria" [containing one strain of Pseudoalteromonas sp. and one strain of Psychrobacter sp.]) isolated from spoiled cooked and whole tropical shrimp stored under modified atmosphere packaging (MAP) was evaluated by inoculation into ionized cooked and peeled tropical shrimp followed by storage for 32 days at 8 degreeC. Microbial growth and sensory changes were monitored during the storage period. The major spoilage bacterial isolate groups were C. maltaromaticum and S. baltica. In order to characterize their spoilage potential further and to study the effect of their interactions, each of these two specific spoilage organisms (SSO) and one mixed-culture, C. maltaromaticum/S. baltica, were tested using a combination of complementary methods: molecular (PCR-TTGE), sensory, chemical, and conventional microbiological analyses. It was concluded that, in the mixed-culture-inoculated samples, both species groups imposed their spoilage characteristics.

Copyright © 2013 Elsevier Ltd. All rights reserved.

Version ID

1

Record Owner

From MEDLINE, a database of the U.S. National Library of Medicine.

Status

MEDLINE

Authors Full Name

Mace, Sabrina; Cardinal, Mireille; Jaffres, Emmanuel; Cornet, Josiane; Lalanne, Valerie; Chevalier, Frederique; Serot, Thierry; Pilet, Marie-France; Dousset, Xavier; Joffraud, Jean-Jacques.

Institution

Mace, Sabrina. LUNAM Universite, ONIRIS, Universite Nantes, UMR1014 Secalim, Nantes F-44307, France; INRA, Nantes F-44307, France; Ifremer, Laboratoire Science et Technologie de la Biomasse Marine, BP 21105, 44311 Nantes Cedex 3, France. Cardinal, Mireille. Ifremer, Laboratoire Science et Technologie de la Biomasse Marine, BP 21105, 44311 Nantes Cedex 3, France.

Jaffres, Emmanuel. LUNAM Universite, ONIRIS, Universite Nantes, UMR1014 Secalim, Nantes F-44307, France; INRA, Nantes F-44307, France.

Cornet, Josiane. Ifremer, Laboratoire Science et Technologie de la Biomasse Marine, BP 21105, 44311 Nantes Cedex 3, France.

Lalanne, Valerie. LUNAM Universite, ONIRIS, Universite Nantes, UMR 6144 GEPEA, Flavor Unit, F-44307, France; CNRS, Nantes F-44307, France.

Chevalier, Frederique. Ifremer, Laboratoire Science et Technologie de la Biomasse Marine, BP 21105, 44311 Nantes Cedex 3, France.

Serot, Thierry. LUNAM Universite, ONIRIS, Universite Nantes, UMR 6144 GEPEA, Flavor Unit, F-44307, France; CNRS, Nantes F-44307, France.

Pilet, Marie-France. LUNAM Universite, ONIRIS, Universite Nantes, UMR1014 Secalim, Nantes F-44307, France; INRA, Nantes F-44307, France.

Dousset, Xavier. LUNAM Universite, ONIRIS, Universite Nantes, UMR1014 Secalim, Nantes F-44307, France; INRA, Nantes F-44307, France.

Joffraud, Jean-Jacques. Ifremer, Laboratoire Science et Technologie de la Biomasse Marine, BP 21105, 44311 Nantes Cedex 3, France. Electronic address: jean.jacques.joffraud@ifremer.fr.

MeSH Heading

Animals. Bacteria/cl [Classification]. Bacteria/ge [Genetics]. Bacteria/gd [Growth & Development]. *Bacteria/ip [Isolation & Purification]. Cooking. *Food Packaging/mt [Methods]. Food Storage. Humans. Penaeidae/ch [Chemistry]. *Penaeidae/mi [Microbiology]. Shellfish/an [Analysis]. *Shellfish/mi [Microbiology]. Taste.

Keyword Heading

Bacterial interaction Cooked tropical shrimp

Specific spoilage organism

Spoilage potential

Volatile compounds.

Year of Publication

2014

Link to the Ovid Full Text or citation:

[Click here for full text options](https://libaccess.mcmaster.ca/login?url=http://ovidsp.ovid.com/ovidweb.cgi?T=JS&CSC=Y&NEWS=N&PAGE=fulltext&D=med11&AN=24549192)

Link to the External Link Resolver:

[SFX](http://sfx.scholarsportal.info/mcmaster?sid=OVID:medline&id=pmid:24549192&id=doi:10.1016%2Fj.fm.2013.11.018&issn=0740-0020&isbn=&volume=40&issue=&spage=9&pages=9-17&date=2014&title=Food+Microbiology&atitle=Evaluation+of+the+spoilage+potential+of+bacteria+isolated+from+spoiled+cooked+whole+tropical+shrimp+(Penaeus+vannamei)+stored+under+modified+atmosphere+packaging.&aulast=Mace&pid=<author>Mace+S%3BCardinal+M%3BJaffres+E%3BCornet+J%3BLalanne+V%3BChevalier+F%3BSerot+T%3BPilet+MF%3BDousset+X%3BJoffraud+JJ<%2Fauthor><AN>24549192<%2FAN><DT>Evaluation+Study<%2FDT>)

29.

Effective survival of immobilized Lactobacillus casei during ripening and heat treatment of probiotic dry-fermented sausages and investigation of the microbial dynamics.

Sidira M; Karapetsas A; Galanis A; Kanellaki M; Kourkoutas Y.

Meat Science. 96(2 Pt A):948-55, 2014 Feb.

[Journal Article. Research Support, Non-U.S. Gov't]

UI: 24211554

The aim was the assessment of immobilized Lactobacillus casei ATCC 393 on wheat in the production of probiotic dry-fermented sausages and the investigation of the microbial dynamics. For comparison, sausages containing either free L. casei ATCC 393 or no starter culture were also prepared. During ripening, the numbers of lactobacilli exceeded 7 log cfu/g, while a drastic decrease was observed in enterobacteria, staphylococci and pseudomonas counts. Microbial diversity was further studied applying a PCR-DGGE protocol. Members of Lactobacillus, Leuconostoc, Lactococcus, Carnobacterium, Brochothrix, Bacillus and Debaryomyces were the main microbial populations detected. Microbiological and strain-specific multiplex PCR analysis confirmed that the levels of L. casei ATCC 393 in the samples after 66 days of ripening were above the minimum concentration for conferring a probiotic effect (>= 6 log cfu/g). However, after heat treatment, this strain was detected at the above levels, only in sausages containing immobilized cells.

Copyright © 2013.

Version ID

1

Record Owner

From MEDLINE, a database of the U.S. National Library of Medicine.

Status

MEDLINE

Authors Full Name

Sidira, Marianthi; Karapetsas, Athanasios; Galanis, Alex; Kanellaki, Maria; Kourkoutas, Yiannis.

Institution

Sidira, Marianthi. Food Biotechnology Group, Section of Analytical Environmental and Applied Chemistry, Department of Chemistry, University of Patras, GR-26500 Patras, Greece.; Applied Microbiology and Molecular Biotechnology Research Group, Department of Molecular Biology & Genetics, Democritus University of Thrace, Alexandroupolis 68100, Greece.

MeSH Heading

Cells, Immobilized. Chemical Phenomena. Colony Count, Microbial. Enterobacteriaceae/ip [Isolation & Purification]. *Fermentation. Food Contamination/pc [Prevention & Control]. *Food Handling/mt [Methods]. Food Microbiology. Hot Temperature. Humans. *Lactobacillus/ip [Isolation & Purification]. Meat Products/an [Analysis]. *Meat Products/mi [Microbiology]. Polymerase Chain Reaction. *Probiotics/me [Metabolism]. Pseudomonas/ip [Isolation & Purification]. Staphylococcus/ip [Isolation & Purification]. Taste.

Keyword Heading

Dry-fermented sausages L. casei ATCC 393

Multiplex PCR

PCR-DGGE

Probiotics.

Year of Publication

2014

Link to the Ovid Full Text or citation:

[Click here for full text options](https://libaccess.mcmaster.ca/login?url=http://ovidsp.ovid.com/ovidweb.cgi?T=JS&CSC=Y&NEWS=N&PAGE=fulltext&D=med11&AN=24211554)

Link to the External Link Resolver:

[SFX](http://sfx.scholarsportal.info/mcmaster?sid=OVID:medline&id=pmid:24211554&id=doi:10.1016%2Fj.meatsci.2013.09.013&issn=0309-1740&isbn=&volume=96&issue=2&spage=948&pages=948-55&date=2014&title=Meat+Science&atitle=Effective+survival+of+immobilized+Lactobacillus+casei+during+ripening+and+heat+treatment+of+probiotic+dry-fermented+sausages+and+investigation+of+the+microbial+dynamics.&aulast=Sidira&pid=<author>Sidira+M%3BKarapetsas+A%3BGalanis+A%3BKanellaki+M%3BKourkoutas+Y<%2Fauthor><AN>24211554<%2FAN><DT>Journal+Article<%2FDT>)

30.

Antimicrobial packaging to retard the growth of spoilage bacteria and to reduce the release of volatile metabolites in meat stored under vacuum at 1degreeC.

Ferrocino I; La Storia A; Torrieri E; Musso SS; Mauriello G; Villani F; Ercolini D.

Journal of Food Protection. 76(1):52-8, 2013 Jan.

[Journal Article. Research Support, Non-U.S. Gov't]

UI: 23317856

A nisin-EDTA solution was used for activation of the internal surface of plastic bags that were used to store beef chops at 1degreeC after vacuum packaging. The aim of the work was to evaluate the effect of the antimicrobial packaging on beef during storage. Volatile compounds and microbial populations were monitored after 0, 9, 20, 36, and 46 days of storage. The active packaging retarded the growth of lactic acid bacteria. Brochothrix thermosphacta was unable to grow for the whole storage time in treated samples, while the levels of Carnobacterium spp. in treated samples were below the detection limit for the first 9 days and reached loads below 5 Log CFU/cm(2) after 46 days. On the other hand, Enterobacteriaceae and Pseudomonas spp. were not affected by the use of the antimicrobial packaging and grew in all of the samples, with final populations of about 4 Log CFU/cm(2). Carnobacterium divergens was identified by PCR-denaturing gradient gel electrophoresis analysis of DNA extracted from beef after 36 days of storage. During beef storage, alcohols, aldehydes, ketones, and carboxylic acids were detected in the headspace of beef samples by solid-phase microextraction-gas chromatography-mass spectrometry analysis. The microbial metabolic activity was affected by the use of the antimicrobial film from the beginning up to 36 days with a maximum in the differences of volatile metabolites in samples analyzed at 20 days. The volatiles were also determined by electronic nose, allowing differentiation based on the time of storage and not on the type of packaging. The active packaging reduces the loads of spoilage microbial populations and the release of metabolites in the headspace of beef with a probable positive impact on meat quality.

Version ID

1

Record Owner

From MEDLINE, a database of the U.S. National Library of Medicine.

Status

MEDLINE

Authors Full Name

Ferrocino, Ilario; La Storia, Antonietta; Torrieri, Elena; Musso, Salvatore Spagna; Mauriello, Gianluigi; Villani, Francesco; Ercolini, Danilo.

Institution

Ferrocino, Ilario. Dipartimento di Scienza degli Alimenti, Universita degli Studi di Napoli Federico II, Via Universita 100, 80055 Portici, Italy.

MeSH Heading

Animals. Anti-Bacterial Agents/pd [Pharmacology]. *Bacteria/gd [Growth & Development]. Bacteria/me [Metabolism]. Carbon Dioxide/an [Analysis]. Cattle. Colony Count, Microbial. *Food Contamination/an [Analysis]. Food Contamination/pc [Prevention & Control]. *Food Handling/mt [Methods]. Food Microbiology. *Food Packaging/mt [Methods]. Humans. *Meat/mi [Microbiology]. Microbial Viability. Nisin/pd [Pharmacology]. Oxygen/an [Analysis]. Temperature. Time Factors. *Vacuum. Volatilization.

Registry Number/Name of Substance

0 (Anti-Bacterial Agents). 1414-45-5 (Nisin). 142M471B3J (Carbon Dioxide). S88TT14065 (Oxygen).

Year of Publication

2013

Link to the Ovid Full Text or citation:

[Click here for full text options](https://libaccess.mcmaster.ca/login?url=http://ovidsp.ovid.com/ovidweb.cgi?T=JS&CSC=Y&NEWS=N&PAGE=fulltext&D=med10&AN=23317856)

Link to the External Link Resolver:

[SFX](http://sfx.scholarsportal.info/mcmaster?sid=OVID:medline&id=pmid:23317856&id=doi:10.4315%2F0362-028X.JFP-12-257&issn=0362-028X&isbn=&volume=76&issue=1&spage=52&pages=52-8&date=2013&title=Journal+of+Food+Protection&atitle=Antimicrobial+packaging+to+retard+the+growth+of+spoilage+bacteria+and+to+reduce+the+release+of+volatile+metabolites+in+meat+stored+under+vacuum+at+1degreeC.&aulast=Ferrocino&pid=<author>Ferrocino+I%3BLa+Storia+A%3BTorrieri+E%3BMusso+SS%3BMauriello+G%3BVillani+F%3BErcolini+D<%2Fauthor><AN>23317856<%2FAN><DT>Journal+Article<%2FDT>)

31.

Evaluation of the spoilage potential of bacteria isolated from spoiled raw salmon (Salmo salar) fillets stored under modified atmosphere packaging.

Mace S; Joffraud JJ; Cardinal M; Malcheva M; Cornet J; Lalanne V; Chevalier F; Serot T; Pilet MF; Dousset X.

International Journal of Food Microbiology. 160(3):227-38, 2013 Jan 01.

[Journal Article. Research Support, Non-U.S. Gov't]

UI: 23290229

The spoilage potential of eight bacterial groups/species (Serratia spp., Hafnia alvei, Brochothrix thermosphacta, Carnobacterium maltaromaticum, Shewanella baltica, Lactococcus piscium, Photobacterium phosphoreum, "other Enterobacteriaceae" [containing one strain of Moellerella sp., Morganella sp. and Pectobacterium sp.]) isolated from spoiled raw salmon fillets stored under modified atmosphere packaging (MAP) was evaluated by inoculation into sterile raw salmon cubes followed by storage for 12days at 8degreeC. Microbial growth and sensory changes were monitored during the storage period. The dominant spoilage bacteria were C. maltaromaticum, H. alvei and P. phosphoreum. In order to further characterize their spoilage potential and to study the effect of their interactions, each of these 3 specific spoilage organisms (SSO) and two mixed-cultures, C. maltaromaticum/H. alvei and C. maltaromaticum/P. phosphoreum were tested in the sterile salmon model system using a combination of complementary methods: molecular (PCR-TTGE), sensory, chemical and conventional microbiological analyses. It was concluded that, in the mixed-culture inoculated samples, the dominant species determined the spoilage characteristics. The volatile fraction of P. phosphoreum inoculated samples was analyzed by solid-phase microextraction (SPME) followed by gas chromatography coupled to mass spectrometry (GC-MS). Among the specific volatile compounds present on P. phosphoreum spoiled inoculated samples, acetic acid was correlated with sensory analysis and can be proposed as a raw salmon spoilage marker.

Copyright © 2012 Elsevier B.V. All rights reserved.

Version ID

1

Record Owner

From MEDLINE, a database of the U.S. National Library of Medicine.

Status

MEDLINE

Authors Full Name

Mace, Sabrina; Joffraud, Jean-Jacques; Cardinal, Mireille; Malcheva, Mariya; Cornet, Josiane; Lalanne, Valerie; Chevalier, Frederique; Serot, Thierry; Pilet, Marie-France; Dousset, Xavier.

Institution

Mace, Sabrina. LUNAM Universite, ONIRIS, Univ Nantes, UMR1014 Secalim, Nantes, France.

MeSH Heading

Animals. Bacteria/gd [Growth & Development]. Bacteria/ip [Isolation & Purification]. Bacterial Load. *Bacterial Physiological Phenomena. *Food Microbiology. Food Packaging/st [Standards]. Humans. Meat/an [Analysis]. *Meat/mi [Microbiology]. Odorants/an [Analysis]. *Salmo salar. Sensation. Time Factors. Volatile Organic Compounds/an [Analysis].

Registry Number/Name of Substance

0 (Volatile Organic Compounds).

Year of Publication

2013

Link to the Ovid Full Text or citation:

[Click here for full text options](https://libaccess.mcmaster.ca/login?url=http://ovidsp.ovid.com/ovidweb.cgi?T=JS&CSC=Y&NEWS=N&PAGE=fulltext&D=med10&AN=23290229)

Link to the External Link Resolver:

[SFX](http://sfx.scholarsportal.info/mcmaster?sid=OVID:medline&id=pmid:23290229&id=doi:10.1016%2Fj.ijfoodmicro.2012.10.013&issn=0168-1605&isbn=&volume=160&issue=3&spage=227&pages=227-38&date=2013&title=International+Journal+of+Food+Microbiology&atitle=Evaluation+of+the+spoilage+potential+of+bacteria+isolated+from+spoiled+raw+salmon+(Salmo+salar)+fillets+stored+under+modified+atmosphere+packaging.&aulast=Mace&pid=<author>Mace+S%3BJoffraud+JJ%3BCardinal+M%3BMalcheva+M%3BCornet+J%3BLalanne+V%3BChevalier+F%3BSerot+T%3BPilet+MF%3BDousset+X<%2Fauthor><AN>23290229<%2FAN><DT>Journal+Article<%2FDT>)

32.

Growth of Carnobacterium spp. from permafrost under low pressure, temperature, and anoxic atmosphere has implications for Earth microbes on Mars.

Nicholson WL; Krivushin K; Gilichinsky D; Schuerger AC.

Proceedings of the National Academy of Sciences of the United States of America. 110(2):666-71, 2013 Jan 08.

[Journal Article. Research Support, U.S. Gov't, Non-P.H.S.]

UI: 23267097

The ability of terrestrial microorganisms to grow in the near-surface environment of Mars is of importance to the search for life and protection of that planet from forward contamination by human and robotic exploration. Because most water on present-day Mars is frozen in the regolith, permafrosts are considered to be terrestrial analogs of the martian subsurface environment. Six bacterial isolates were obtained from a permafrost borehole in northeastern Siberia capable of growth under conditions of low temperature (0 degreeC), low pressure (7 mbar), and a CO(2)-enriched anoxic atmosphere. By 16S ribosomal DNA analysis, all six permafrost isolates were identified as species of the genus Carnobacterium, most closely related to C. inhibens (five isolates) and C. viridans (one isolate). Quantitative growth assays demonstrated that the six permafrost isolates, as well as nine type species of Carnobacterium (C. alterfunditum, C. divergens, C. funditum, C. gallinarum, C. inhibens, C. maltaromaticum, C. mobile, C. pleistocenium, and C. viridans) were all capable of growth under cold, low-pressure, anoxic conditions, thus extending the low-pressure extreme at which life can function.

Version ID

1

Record Owner

From MEDLINE, a database of the U.S. National Library of Medicine.

Status

MEDLINE

Authors Full Name

Nicholson, Wayne L; Krivushin, Kirill; Gilichinsky, David; Schuerger, Andrew C.

Institution

Nicholson, Wayne L. Department of Microbiology and Cell Science, Space Life Sciences Laboratory, University of Florida, Merritt Island, FL 32953, USA. WLN@ufl.edu

MeSH Heading

Anaerobiosis. Atmospheric Pressure. Base Sequence. *Carnobacterium/ge [Genetics]. *Carnobacterium/gd [Growth & Development]. Cluster Analysis. DNA, Ribosomal/ge [Genetics]. Exobiology. *Extraterrestrial Environment. *Mars. Molecular Sequence Data. Phylogeny. Sequence Analysis, DNA. Siberia. *Soil Microbiology. Species Specificity. Temperature.

Registry Number/Name of Substance

0 (DNA, Ribosomal).

Year of Publication

2013

Link to the Ovid Full Text or citation:

[Click here for full text options](https://libaccess.mcmaster.ca/login?url=http://ovidsp.ovid.com/ovidweb.cgi?T=JS&CSC=Y&NEWS=N&PAGE=fulltext&D=med10&AN=23267097)

Link to the External Link Resolver:

[SFX](http://sfx.scholarsportal.info/mcmaster?sid=OVID:medline&id=pmid:23267097&id=doi:10.1073%2Fpnas.1209793110&issn=0027-8424&isbn=&volume=110&issue=2&spage=666&pages=666-71&date=2013&title=Proceedings+of+the+National+Academy+of+Sciences+of+the+United+States+of+America&atitle=Growth+of+Carnobacterium+spp.+from+permafrost+under+low+pressure%2C+temperature%2C+and+anoxic+atmosphere+has+implications+for+Earth+microbes+on+Mars.&aulast=Nicholson&pid=<author>Nicholson+WL%3BKrivushin+K%3BGilichinsky+D%3BSchuerger+AC<%2Fauthor><AN>23267097<%2FAN><DT>Journal+Article<%2FDT>)

33.

Investigation of spoilage in saveloy samples inoculated with four potential spoilage bacteria.

Holm ES; Schafer A; Koch AG; Petersen MA.

Meat Science. 93(3):687-95, 2013 Mar.

[Journal Article. Research Support, Non-U.S. Gov't]

UI: 23261532

Sliced saveloy samples were inoculated with monocultures of four potential spoilage bacteria and studied during a four week storage period. The objective was to investigate the resulting changes in the composition of Volatile Organic Compounds (VOCs) and the sensory quality of the product. Based on the sensory scores and the VOC composition Brochothrix thermosphacta, Chryseomonas luteola and Carnobacterium maltaromaticum were found to have a high spoilage potential in saveloy samples subjected to consumer simulated storage during the fourth week. Inoculation with Leuconostoc carnosum only resulted in a low level of spoilage. The sensory changes in the saveloy samples were modeled based on the VOC composition using Partial Least Squares Regression. The changes in the six sensory descriptors were closely related to the amount of diacetyl, acetoin, 2- and 3-methylbutanol, 2- and 3-methylbutanal and 2-methylpropanol found in the samples. These compounds are therefore potentially important for the shelf-life of sliced saveloy.

Copyright © 2012 Elsevier Ltd. All rights reserved.

Version ID

1

Record Owner

From MEDLINE, a database of the U.S. National Library of Medicine.

Status

MEDLINE

Authors Full Name

Holm, E S; Schafer, A; Koch, A G; Petersen, M A.

Institution

Holm, E S. Department of Food Science, Quality & Technology, Faculty of Science, University of Copenhagen, Rolighedsvej 30, 1958 Frederiksberg C, Denmark. esbenskibstedholm@gmail.com

MeSH Heading

Animals. *Bacteria. *Food Microbiology. Food Storage. Humans. Least-Squares Analysis. *Meat Products/mi [Microbiology]. *Odorants. *Volatile Organic Compounds/me [Metabolism].

Registry Number/Name of Substance

0 (Volatile Organic Compounds).

Year of Publication

2013

Link to the Ovid Full Text or citation:

[Click here for full text options](https://libaccess.mcmaster.ca/login?url=http://ovidsp.ovid.com/ovidweb.cgi?T=JS&CSC=Y&NEWS=N&PAGE=fulltext&D=med10&AN=23261532)

Link to the External Link Resolver:

[SFX](http://sfx.scholarsportal.info/mcmaster?sid=OVID:medline&id=pmid:23261532&id=doi:10.1016%2Fj.meatsci.2012.11.016&issn=0309-1740&isbn=&volume=93&issue=3&spage=687&pages=687-95&date=2013&title=Meat+Science&atitle=Investigation+of+spoilage+in+saveloy+samples+inoculated+with+four+potential+spoilage+bacteria.&aulast=Holm&pid=<author>Holm+ES%3BSchafer+A%3BKoch+AG%3BPetersen+MA<%2Fauthor><AN>23261532<%2FAN><DT>Journal+Article<%2FDT>)

34.

Bacterial diversity and spoilage-related microbiota associated with freshly prepared chicken products under aerobic conditions at 4degreeC.

Liang R; Yu X; Wang R; Luo X; Mao Y; Zhu L; Zhang Y.

Journal of Food Protection. 75(6):1057-62, 2012 Jun.

[Journal Article. Research Support, Non-U.S. Gov't]

UI: 22691472

This study analyzed the bacterial diversity and spoilage-related microbiota associated with freshly prepared chicken products stored aerobically at 4degreeC, using "bone and chicken string," a product popular in the People's Republic of China, as the study subject. Samples collected from three different factories were tray packaged with cling film and stored at 4degreeC. Bacterial diversity and dominant bacteria were analyzed using PCR amplification and denaturing gradient gel electrophoresis. Combined with selective cultivation of the dominant bacteria and correlation analysis, the dominant spoilage microbiota was determined. The results showed that bacterial diversity varied with different manufacturers. Such bacteria as Acinetobacter sp., Carnobacterium sp., Rahnella sp., Pseudomonas sp., Brochothrix sp., and Weissella sp. were detected in freshly prepared chicken products during storage. And Carnobacterium sp., Pseudomonas sp., and Brochothrix sp. bacteria were the common dominant spoilage bacteria groups in most freshly prepared chicken products from different factories. Carnobacterium was, for the first time, shown to be an important contributor to the spoilage-related microflora of freshly prepared chicken products stored aerobically under refrigeration. Our work shows the bacterial diversity and dominant spoilage microbiota of freshly prepared chicken products stored aerobically under refrigeration.

Version ID

1

Record Owner

From MEDLINE, a database of the U.S. National Library of Medicine.

Status

MEDLINE

Authors Full Name

Liang, Rongrong; Yu, Xiaoqiao; Wang, Renhuan; Luo, Xin; Mao, Yanwei; Zhu, Lixian; Zhang, Yimin.

Institution

Liang, Rongrong. Professional Laboratory of Beef Processing and Quality Control, College of Food Science and Engineering, Shandong Agricultural University, Tai'an, 271018, People's Republic of China.

MeSH Heading

Animals. *Bacteria/ip [Isolation & Purification]. Chickens. Cold Temperature. Colony Count, Microbial. *DNA, Bacterial/an [Analysis]. Electrophoresis, Polyacrylamide Gel. *Food Handling/mt [Methods]. Food Microbiology. *Food Packaging/mt [Methods]. Humans. Meat-Packing Industry/st [Standards]. Oxygen. Polymerase Chain Reaction. *Poultry Products/mi [Microbiology].

Registry Number/Name of Substance

0 (DNA, Bacterial). S88TT14065 (Oxygen).

Year of Publication

2012

Link to the Ovid Full Text or citation:

[Click here for full text options](https://libaccess.mcmaster.ca/login?url=http://ovidsp.ovid.com/ovidweb.cgi?T=JS&CSC=Y&NEWS=N&PAGE=fulltext&D=med9&AN=22691472)

Link to the External Link Resolver:

[SFX](http://sfx.scholarsportal.info/mcmaster?sid=OVID:medline&id=pmid:22691472&id=doi:10.4315%2F0362-028X.JFP-11-439&issn=0362-028X&isbn=&volume=75&issue=6&spage=1057&pages=1057-62&date=2012&title=Journal+of+Food+Protection&atitle=Bacterial+diversity+and+spoilage-related+microbiota+associated+with+freshly+prepared+chicken+products+under+aerobic+conditions+at+4degreeC.&aulast=Liang&pid=<author>Liang+R%3BYu+X%3BWang+R%3BLuo+X%3BMao+Y%3BZhu+L%3BZhang+Y<%2Fauthor><AN>22691472<%2FAN><DT>Journal+Article<%2FDT>)

35.

Carnobacterium divergens - a dominating bacterium of pork meat juice.

Rieder G; Krisch L; Fischer H; Kaufmann M; Maringer A; Wessler S.

FEMS Microbiology Letters. 332(2):122-30, 2012 Jul.

[Letter]

UI: 22537055

Nonspoiled food that nevertheless contains bacterial pathogens constitutes a much more serious health problem than spoiled food, as the consumer is not warned beforehand. However, data on the diversity of bacterial species in meat juice are rare. To study the bacterial load of fresh pork from ten different distributors, we applied a combination of the conventional culture-based and molecular methods for detecting and quantifying the microbial spectrum of fresh pork meat juice samples. Altogether, we identified 23 bacterial species of ten different families analyzed by 16S rRNA gene sequencing. The majority of isolates were belonging to the typical spoilage bacterial population of lactic acid bacteria (LAB), Enterococcaceae, and Pseudomonadaceae. Several additional isolates were identified as Staphylococcus spp. and Bacillus spp. originating from human and animal skin and other environmental niches including plants, soil, and water. Carnobacterium divergens, a LAB contributing to the spoilage of raw meat even at refrigeration temperature, was the most frequently isolated species in our study (5/10) with a bacterial load of 10(3) - 10(7) CFU mL(-1). In several of the analyzed pork meat juice samples, two bacterial faecal indicators, Serratia grimesii and Serratia proteamaculans, were identified together with another opportunistic food-borne pathogen, Staphylococcus equorum. Our data reveal a high bacterial load of fresh pork meat supporting the potential health risk of meat juice for the end consumer even under refrigerated conditions.

Copyright © 2012 Federation of European Microbiological Societies. Published by Blackwell Publishing Ltd. All rights reserved.

Version ID

1

Record Owner

From MEDLINE, a database of the U.S. National Library of Medicine.

Status

MEDLINE

Authors Full Name

Rieder, Gabriele; Krisch, Linda; Fischer, Harald; Kaufmann, Maria; Maringer, Adolf; Wessler, Silja.

Institution

Rieder, Gabriele. Division of Microbiology, Department of Molecular Biology, University of Salzburg, Salzburg, Austria. gabriele.rieder@sbg.ac.at

MeSH Heading

Animals. *Bacteria/cl [Classification]. Bacteria/ge [Genetics]. *Bacteria/ip [Isolation & Purification]. Bacterial Load. *Biodiversity. Cluster Analysis. DNA, Bacterial/ch [Chemistry]. DNA, Bacterial/ge [Genetics]. DNA, Ribosomal/ch [Chemistry]. DNA, Ribosomal/ge [Genetics]. *Meat/mi [Microbiology]. Phylogeny. RNA, Ribosomal, 16S/ge [Genetics]. Sequence Analysis, DNA. Swine.

Registry Number/Name of Substance

0 (DNA, Bacterial). 0 (DNA, Ribosomal). 0 (RNA, Ribosomal, 16S).

Year of Publication

2012

Link to the Ovid Full Text or citation:

[Click here for full text options](https://libaccess.mcmaster.ca/login?url=http://ovidsp.ovid.com/ovidweb.cgi?T=JS&CSC=Y&NEWS=N&PAGE=fulltext&D=med9&AN=22537055)

Link to the External Link Resolver:

[SFX](http://sfx.scholarsportal.info/mcmaster?sid=OVID:medline&id=pmid:22537055&id=doi:10.1111%2Fj.1574-6968.2012.02584.x&issn=0378-1097&isbn=&volume=332&issue=2&spage=122&pages=122-30&date=2012&title=FEMS+Microbiology+Letters&atitle=Carnobacterium+divergens+-+a+dominating+bacterium+of+pork+meat+juice.&aulast=Rieder&pid=<author>Rieder+G%3BKrisch+L%3BFischer+H%3BKaufmann+M%3BMaringer+A%3BWessler+S<%2Fauthor><AN>22537055<%2FAN><DT>Letter<%2FDT>)

36.

Phylogenetic analysis of antimicrobial lactic acid bacteria from farmed seabass Dicentrarchus labrax.

Bourouni OC; El Bour M; Calo-Mata P; Mraouna R; Abedellatif B; Barros-Velazquez J.

Canadian Journal of Microbiology. 58(4):463-74, 2012 Apr.

[Journal Article]

UI: 22439634

The use of lactic acid bacteria (LAB) in the prevention or reduction of fish diseases is receiving increasing attention. In the present study, 47 LAB strains were isolated from farmed seabass ( Dicentrarchus labrax ) and were phenotypically and phylogenetically analysed by 16S rDNA and randomly amplified polymorphic DNA - polymerase chain reaction (RAPD-PCR). Their antimicrobial effect was tested in vitro against a wide variety of pathogenic and spoilage bacteria. Most of the strains isolated were enterococci belonging to the following species: Enterococcus faecium (59%), Enterococcus faecalis (21%), Enterococcus sanguinicola (4 strains), Enterococcus mundtii (1 strain), Enterococcus pseudoavium (1 strain), and Lactococcus lactis (1 strain). An Aerococcus viridans strain was also isolated. The survey of their antimicrobial susceptibility showed that all isolates were sensitive to vancomycin and exhibited resistance to between 4 and 10 other antibiotics relevant for therapy in human and animal medicine. Different patterns of resistance were noted for skin and intestines isolates. More than 69% (32 strains) of the isolates inhibited the growth of the majority of pathogenic and spoilage bacteria tested, including Listeria monocytogenes, Staphylococcus aureus, Aeromonas hydrophila, Aeromonas salmonicida, Vibrio anguillarum, and Carnobacterium sp. To our knowledge, this is the first report of bioactive enterococcal species isolated from seabass that could potentially inhibit the undesirable bacteria found in food systems.

Version ID

1

Record Owner

From MEDLINE, a database of the U.S. National Library of Medicine.

Status

MEDLINE

Authors Full Name

Bourouni, Ouissal Chahad; El Bour, Monia; Calo-Mata, Pilar; Mraouna, Radhia; Abedellatif, Boudabous; Barros-Velazquez, Jorge.

Institution

Bourouni, Ouissal Chahad. Institut national des sciences et technologies de la mer, rue 2 Mars 1934, 2025 Salammbo, Tunis, Tunisia.

MeSH Heading

Animals. Anti-Bacterial Agents/pd [Pharmacology]. Aquaculture. *Bacteria/cl [Classification]. Bacteria/ge [Genetics]. Bacteria/ip [Isolation & Purification]. Bacteria/me [Metabolism]. Base Sequence. *Bass/mi [Microbiology]. Enterococcus/cl [Classification]. Enterococcus/ge [Genetics]. Enterococcus/ip [Isolation & Purification]. Enterococcus/ph [Physiology]. Humans. Lactic Acid/me [Metabolism]. Molecular Sequence Data. Phylogeny. Polymerase Chain Reaction. Random Amplified Polymorphic DNA Technique. Seafood/mi [Microbiology]. Vancomycin/pd [Pharmacology]. Vancomycin Resistance/ge [Genetics].

Registry Number/Name of Substance

0 (Anti-Bacterial Agents). 33X04XA5AT (Lactic Acid). 6Q205EH1VU (Vancomycin).

Year of Publication

2012

Link to the Ovid Full Text or citation:

[Click here for full text options](https://libaccess.mcmaster.ca/login?url=http://ovidsp.ovid.com/ovidweb.cgi?T=JS&CSC=Y&NEWS=N&PAGE=fulltext&D=med9&AN=22439634)

Link to the External Link Resolver:

[SFX](http://sfx.scholarsportal.info/mcmaster?sid=OVID:medline&id=pmid:22439634&id=doi:10.1139%2Fw2012-014&issn=0008-4166&isbn=&volume=58&issue=4&spage=463&pages=463-74&date=2012&title=Canadian+Journal+of+Microbiology&atitle=Phylogenetic+analysis+of+antimicrobial+lactic+acid+bacteria+from+farmed+seabass+Dicentrarchus+labrax.&aulast=Bourouni&pid=<author>Bourouni+OC%3BEl+Bour+M%3BCalo-Mata+P%3BMraouna+R%3BAbedellatif+B%3BBarros-Velazquez+J<%2Fauthor><AN>22439634<%2FAN><DT>Journal+Article<%2FDT>)

37.

High pressure inactivation of Escherichia coli, Campylobacter jejuni, and spoilage microbiota on poultry meat.

Liu Y; Betti M; Ganzle MG.

Journal of Food Protection. 75(3):497-503, 2012 Mar.

[Journal Article. Research Support, Non-U.S. Gov't]

UI: 22410223

This study evaluated the high pressure inactivation of Campylobacter jejuni, Escherichia coli, and poultry meat spoilage organisms. All treatments were performed in aseptically prepared minced poultry meat. Treatment of 19 strains of C. jejuni at 300 MPa and 30degreeC revealed a large variation of pressure resistance. The recovery of pressure-induced sublethally injured C. jejuni depended on the availability of iron. The addition of iron content to enumeration media was required for resuscitation of sublethally injured cells. Survival of C. jejuni during storage of refrigerated poultry meat was analyzed in fresh and pressuretreated poultry meat, and in the presence or absence of spoilage microbiota. The presence of spoilage microbiota did not significantly influence the survival of C. jejuni. Pressure treatment at 400 MPa and 40degreeC reduced cell counts of Brochothrix thermosphacta, Carnobacterium divergens, C. jejuni, and Pseudomonas fluorescens to levels below the detection limit. Cell counts of E. coli AW1.7, however, were reduced by only 3.5 log (CFU/g) and remained stable during subsequent refrigerated storage. The resistance to treatment at 600 MPa and 40degreeC of E. coli AW1.7 was compared with Salmonella enterica, Shiga toxin-producing E. coli and nonpathogenic E. coli strains, and Staphylococcus spp. Cell counts of all organisms except E. coli AW 1.7 were reduced by more than 6 log CFU/g. Cell counts of E. coli AW1.7 were reduced by 4.5 log CFU/g only. Moreover, the ability of E. coli AW1.7 to resist pressure was comparable to the pressure-resistant mutant E. coli LMM1030. Our results indicate that preservation of fresh meat requires a combination of high pressure with high temperature (40 to 60degreeC) or other antimicrobial hurdles.

Version ID

1

Record Owner

From MEDLINE, a database of the U.S. National Library of Medicine.

Status

MEDLINE

Authors Full Name

Liu, Yang; Betti, Mirko; Ganzle, Michael G.

Institution

Liu, Yang. Department of Agricultural, Food and Nutritional Sciences, University of Alberta, Edmonton, Alberta T6G 2P5, Canada.

MeSH Heading

Animals. *Campylobacter jejuni/gd [Growth & Development]. Campylobacter jejuni/me [Metabolism]. Colony Count, Microbial. Consumer Product Safety. *Escherichia coli/gd [Growth & Development]. *Food Handling/mt [Methods]. Hot Temperature. Humans. Iron/me [Metabolism]. *Poultry Products/mi [Microbiology]. *Pressure.

Registry Number/Name of Substance

E1UOL152H7 (Iron).

Year of Publication

2012

Link to the Ovid Full Text or citation:

[Click here for full text options](https://libaccess.mcmaster.ca/login?url=http://ovidsp.ovid.com/ovidweb.cgi?T=JS&CSC=Y&NEWS=N&PAGE=fulltext&D=med9&AN=22410223)

Link to the External Link Resolver:

[SFX](http://sfx.scholarsportal.info/mcmaster?sid=OVID:medline&id=pmid:22410223&id=doi:10.4315%2F0362-028X.JFP-11-316&issn=0362-028X&isbn=&volume=75&issue=3&spage=497&pages=497-503&date=2012&title=Journal+of+Food+Protection&atitle=High+pressure+inactivation+of+Escherichia+coli%2C+Campylobacter+jejuni%2C+and+spoilage+microbiota+on+poultry+meat.&aulast=Liu&pid=<author>Liu+Y%3BBetti+M%3BGanzle+MG<%2Fauthor><AN>22410223<%2FAN><DT>Journal+Article<%2FDT>)

38.

The effect of crowding stress on bacterial growth and sensory properties of chilled Atlantic salmon fillets.

Hansen AA; Rodbotten M; Eie T; Lea P; Rudi K; Morkore T.

Journal of Food Science. 77(1):S84-90, 2012 Jan.

[Comparative Study. Journal Article. Research Support, Non-U.S. Gov't]

UI: 22260135

UNLABELLED: Atlantic salmon were subjected to minimal preslaughter crowding stress (Control), short-term crowding for 20 min (SS-group), or long-term crowding for 24 h (LS-group). The fish were filleted prerigor, cut into 270 g pieces, and packaged in modified atmosphere (60% CO2 and 40% N2). Fillet quality analyses were determined during 22 d of storage at 0.3 degreeC. Bacterial growth and unpleasant sensory properties increased earlier in the LS-group. The negative effects of long-term preslaughter stress were more pronounced for raw than cooked samples, and more pronounced for odor than flavor. Sequence analyses of bacterial DNA at the end of storage revealed that 100% of the bacteria were comprised by Photobacterium phosphoreum of the SS- and LS-group, whereas the Control group also contained 21% of Carnobacterium maltaromaticum (lactic acid bacteria, LAB). Counting of LAB, using Man-Rogosa-Sharke agar, similarly showed higher numbers of the Control group after 15 d of storage. A total bacterial count of log 6 CFU/g was observed after 15 d of storage of the LS-group, which was 3 and 7 d earlier compared with the Control and SS-group, respectively. Fillet color, texture, and liquid losses were not negatively affected by preslaughter crowding stress. From the sensory and bacterial analyses, it is concluded that long-term crowding stress accelerates bacterial growth and development of unpleasant sensory properties, hence reduces the shelf life of prerigor modified atmosphere packaged (MAP) salmon.

PRACTICAL APPLICATION: Stressful handling of Atlantic salmon before slaughter resulted in faster reduction of fresh taste and smell, faster bacterial growth, and hence shorter shelf life. The deteriorative effects were more pronounced of raw compared to cooked salmon. Therefore, salmon should be handled carefully in connection with slaughter to avoid impaired welfare and fillet quality, in particularly for fish that is consumed raw, such as sushi.

Copyright © 2011 Institute of Food Technologists R

Version ID

1

Record Owner

From MEDLINE, a database of the U.S. National Library of Medicine.

Status

MEDLINE

Authors Full Name

Hansen, Anlaug Adland; Rodbotten, Marit; Eie, Thomas; Lea, Per; Rudi, Knut; Morkore, Turid.

Institution

Hansen, Anlaug Adland. Nofima AS, Osloveien 1, N-1430 As, Norway. anlaug.hansen@nofima.no

MeSH Heading

Animals. Aquaculture. Carnobacterium/cl [Classification]. Carnobacterium/gd [Growth & Development]. Carnobacterium/ip [Isolation & Purification]. Chemical Phenomena. Colony Count, Microbial. *Crowding. Food Handling. Hot Temperature. Humans. Mechanical Phenomena. Molecular Typing. Odorants. Photobacterium/cl [Classification]. Photobacterium/gd [Growth & Development]. Photobacterium/ip [Isolation & Purification]. Quality Control. *Salmo salar/mi [Microbiology]. *Salmo salar/ph [Physiology]. *Seafood/an [Analysis]. *Seafood/mi [Microbiology]. Sensation. *Stress, Physiological. Taste. Time Factors.

Year of Publication

2012

Link to the Ovid Full Text or citation:

[Click here for full text options](https://libaccess.mcmaster.ca/login?url=http://ovidsp.ovid.com/ovidweb.cgi?T=JS&CSC=Y&NEWS=N&PAGE=fulltext&D=med9&AN=22260135)

Link to the External Link Resolver:

[SFX](http://sfx.scholarsportal.info/mcmaster?sid=OVID:medline&id=pmid:22260135&id=doi:10.1111%2Fj.1750-3841.2011.02513.x&issn=0022-1147&isbn=&volume=77&issue=1&spage=S84&pages=S84-90&date=2012&title=Journal+of+Food+Science&atitle=The+effect+of+crowding+stress+on+bacterial+growth+and+sensory+properties+of+chilled+Atlantic+salmon+fillets.&aulast=Hansen&pid=<author>Hansen+AA%3BRodbotten+M%3BEie+T%3BLea+P%3BRudi+K%3BMorkore+T<%2Fauthor><AN>22260135<%2FAN><DT>Comparative+Study<%2FDT>)

39.

The genome sequence of the lactic acid bacterium, Carnobacterium maltaromaticum ATCC 35586 encodes potential virulence factors.

Leisner JJ; Hansen MA; Larsen MH; Hansen L; Ingmer H; Sorensen SJ.

International Journal of Food Microbiology. 152(3):107-15, 2012 Jan 16.

[Journal Article]

UI: 21704418

The genus Carnobacterium belongs to the lactic acid bacteria and Carnobacterium maltaromaticum is commonly found in modified atmosphere packed and vacuum packed fish and meat products as well as in live fish. This species has been described as a fish pathogenic organism but human clinical isolates have only been obtained at one occasion. To investigate the virulence potential we sequenced the entire genome of strain ATCC 35586, isolated from a diseased salmon. When comparing the translated gene products of ATCC 35586 to those of Gram positive bacterial pathogens and probiotics as well as the related Carnobacterium sp. AT7 we identified a range of putative virulence genes including genes encoding products involved in adhesion to fibronectin and collagen, capsule synthesis, cell wall modification, iron scavenging mechanisms, haemolysis, invasion and resistance to toxic compounds. Of particular interest was the presence of internalin encoding gene homologues to some of those found in Listeria spp. and Lactobacillus plantarum. Furthermore, the ATCC 35586 strain possesses a gene encoding a product similar to the central Listeria monocytogenes transcriptional regulator PrfA, that in this organism controls virulence gene expression by binding to conserved DNA binding sites. Based on the consensus DNA sequence of this binding site, we identified a total of 65 genes in the ATCC 35586 genome that in the upstream region carry a PrfA binding motif. Among these is one of the internalin encoding genes; two genes encoding products involved in capsule biosynthesis as well as various genes encoding products with metabolic functions. In contrast to L. monocytogenes, the ATCC 35586 strain did not encode other PrfA dependent virulence factors such as listeriolysin O, phospholipases A and B, ActA, listeriolysin O, zinc metallo protease and internalins A and B. In conclusion, C. maltaromaticum ATCC 35586 carries putative virulence genes that may explain its reported ability to infect fish. The findings of this study give no reason for concern regarding human health by the presence of this species in food products.

Copyright © 2011 Elsevier B.V. All rights reserved.

Version ID

1

Record Owner

From MEDLINE, a database of the U.S. National Library of Medicine.

Status

MEDLINE

Authors Full Name

Leisner, J J; Hansen, M A; Larsen, M H; Hansen, L; Ingmer, H; Sorensen, S J.

Institution

Leisner, J J. Department of Veterinary Disease Biology, Faculty of Life Sciences, University of Copenhagen, Denmark. jjl@life.ku.dk

MeSH Heading

Animals. *Carnobacterium/ge [Genetics]. Carnobacterium/me [Metabolism]. *Carnobacterium/py [Pathogenicity]. Drug Resistance, Bacterial. Fishes/mi [Microbiology]. Gene Expression Regulation, Bacterial. *Genome, Bacterial. Lactic Acid/me [Metabolism]. Listeria monocytogenes/ge [Genetics]. Meat Products/mi [Microbiology]. Peptide Termination Factors/me [Metabolism]. *Virulence Factors/ge [Genetics]. Virulence Factors/me [Metabolism].

Registry Number/Name of Substance

0 (Peptide Termination Factors). 0 (Virulence Factors). 33X04XA5AT (Lactic Acid).

Year of Publication

2012

Link to the Ovid Full Text or citation:

[Click here for full text options](https://libaccess.mcmaster.ca/login?url=http://ovidsp.ovid.com/ovidweb.cgi?T=JS&CSC=Y&NEWS=N&PAGE=fulltext&D=med9&AN=21704418)

Link to the External Link Resolver:

[SFX](http://sfx.scholarsportal.info/mcmaster?sid=OVID:medline&id=pmid:21704418&id=doi:10.1016%2Fj.ijfoodmicro.2011.05.012&issn=0168-1605&isbn=&volume=152&issue=3&spage=107&pages=107-15&date=2012&title=International+Journal+of+Food+Microbiology&atitle=The+genome+sequence+of+the+lactic+acid+bacterium%2C+Carnobacterium+maltaromaticum+ATCC+35586+encodes+potential+virulence+factors.&aulast=Leisner&pid=<author>Leisner+JJ%3BHansen+MA%3BLarsen+MH%3BHansen+L%3BIngmer+H%3BSorensen+SJ<%2Fauthor><AN>21704418<%2FAN><DT>Journal+Article<%2FDT>)

40.

Tetracycline resistance associated with commensal bacteria from representative ready-to-consume deli and restaurant foods.

Li X; Wang HH.

Journal of Food Protection. 73(10):1841-8, 2010 Oct.

[Journal Article. Research Support, Non-U.S. Gov't]

UI: 21067672

Proper knowledge of antibiotic resistance (AR) dissemination is essential for effective mitigation. This study examined the profiles of tetracycline-resistant (Tetr) commensal bacteria from representative ready-to-consume food samples from salad bars at local grocery stores and restaurants. Out of 900 Tetr isolates examined, 158 (17.6%) carried one or more of tetM, tetL, tetS, and tetK genes by conventional PCR, 28 harbored more than one Tetr determinants. The most prevalent genotype was tetM, which was detected in 70.9% of the AR gene carriers, followed by tetL (31.6%), tetS (13.9%), and tetK (2.5%). Identified AR gene carriers included Enterococcus, Lactococcus, Staphylococcus, Brochothrix, Carnobacterium, Stenotrophomonas, Pseudomonas, and Sphingobacterium, by 16S rRNA gene sequence analysis. AR determinants were successfully transmitted, and led to resistance in Streptococcus mutans via natural gene transformation and Enterococcus faecalis via electroporation, suggesting the functionality and mobility of the AR genes from the food commensal bacteria. In addition, the AR traits in many isolates are quite stable, even in the absence of the selective pressure. The identification of new commensal carriers for representative AR genes revealed the involvement of a broad spectrum of bacteria in the horizontal transmission of AR genes. Meanwhile, the spectrum of the antibiotic-resistant bacteria differed from the spectrum of the total bacteria (by denaturing gradient gel electrophoresis) associated with the food items. Our data revealed a common avenue in AR exposure and will assist in proper risk assessment and the development of comprehensive mitigation strategies to effectively combat AR.

Version ID

1

Record Owner

From MEDLINE, a database of the U.S. National Library of Medicine.

Status

MEDLINE

Authors Full Name

Li, Xiaojing; Wang, Hua H.

Institution

Li, Xiaojing. Department of Food Science, The Ohio State University, 2015 Fyffe Court, Columbus, Ohio 43210, USA.

MeSH Heading

*Anti-Bacterial Agents/pd [Pharmacology]. Bacteria/de [Drug Effects]. *Bacteria/ge [Genetics]. Drug Resistance, Bacterial/ge [Genetics]. *Food Microbiology. Gene Transfer, Horizontal. Humans. *Meat Products/mi [Microbiology]. Microbial Sensitivity Tests. Restaurants. *Tetracycline Resistance/ge [Genetics].

Registry Number/Name of Substance

0 (Anti-Bacterial Agents).

Year of Publication

2010

Link to the Ovid Full Text or citation:

[Click here for full text options](https://libaccess.mcmaster.ca/login?url=http://ovidsp.ovid.com/ovidweb.cgi?T=JS&CSC=Y&NEWS=N&PAGE=fulltext&D=med8&AN=21067672)

Link to the External Link Resolver:

[SFX](http://sfx.scholarsportal.info/mcmaster?sid=OVID:medline&id=pmid:21067672&id=doi:10.4315%2F0362-028x-73.10.1841&issn=0362-028X&isbn=&volume=73&issue=10&spage=1841&pages=1841-8&date=2010&title=Journal+of+Food+Protection&atitle=Tetracycline+resistance+associated+with+commensal+bacteria+from+representative+ready-to-consume+deli+and+restaurant+foods.&aulast=Li&pid=<author>Li+X%3BWang+HH<%2Fauthor><AN>21067672<%2FAN><DT>Journal+Article<%2FDT>)

41.

Interactions between bacterial isolates from modified-atmosphere-packaged artisan-type cooked ham in view of the development of a bioprotective culture.

Vasilopoulos C; De Mey E; Dewulf L; Paelinck H; De Smedt A; Vandendriessche F; De Vuyst L; Leroy F.

Food Microbiology. 27(8):1086-94, 2010 Dec.

[Journal Article. Research Support, Non-U.S. Gov't]

UI: 20832689

Growth and metabolite production of three dominant bacterial isolates (Carnobacterium divergens 3M14, Leuconostoc carnosum 3M42, and Brochothrix thermosphacta RMS6) from modified-atmosphere-packaged (MAP), artisan-type cooked ham were assessed for their interactions in view of the development of a bioprotective culture. During monoculture experiments in cooked ham simulation medium, Leuc. carnosum 3M42 converted the available glucose into lactic acid and ethanol, whereas the two other strains produced additional metabolites such as acetic acid and 3-methyl-1-butanol. When grown in co-culture, Leuc. carnosum 3M42 suppressed the growth and metabolism of B. thermosphacta RMS6. In contrast, a co-culture of the latter bacterium with C. divergens 3M14 led to a variety of spoilage-related metabolic compounds. Subsequently, experiments with a commercial cooked ham product indicated that Leuc. carnosum 3M42 dominated the meat matrix and improved acceptability of the product over time, hence acting as a bioprotective culture for MAP, artisan-type cooked ham.

Copyright © 2010 Elsevier Ltd. All rights reserved.

Version ID

1

Record Owner

From MEDLINE, a database of the U.S. National Library of Medicine.

Status

MEDLINE

Authors Full Name

Vasilopoulos, Charalampos; De Mey, Eveline; Dewulf, Lore; Paelinck, Hubert; De Smedt, Anny; Vandendriessche, Frank; De Vuyst, Luc; Leroy, Frederic.

Institution

Vasilopoulos, Charalampos. Research Group of Industrial Microbiology and Food Biotechnology (IMDO), Faculty of Sciences and Bio-engineering Sciences, Vrije Universiteit Brussel, Brussels, Belgium.

MeSH Heading

Animals. *Brochothrix/gd [Growth & Development]. Brochothrix/ip [Isolation & Purification]. Brochothrix/me [Metabolism]. *Carnobacterium/gd [Growth & Development]. Carnobacterium/ip [Isolation & Purification]. Carnobacterium/me [Metabolism]. Cattle. Fermentation. Food Packaging. Humans. *Leuconostoc/gd [Growth & Development]. Leuconostoc/ip [Isolation & Purification]. Leuconostoc/me [Metabolism]. Meat Products/an [Analysis]. *Meat Products/mi [Microbiology]. Taste.

Year of Publication

2010

Link to the Ovid Full Text or citation:

[Click here for full text options](https://libaccess.mcmaster.ca/login?url=http://ovidsp.ovid.com/ovidweb.cgi?T=JS&CSC=Y&NEWS=N&PAGE=fulltext&D=med8&AN=20832689)

Link to the External Link Resolver:

[SFX](http://sfx.scholarsportal.info/mcmaster?sid=OVID:medline&id=pmid:20832689&id=doi:10.1016%2Fj.fm.2010.07.013&issn=0740-0020&isbn=&volume=27&issue=8&spage=1086&pages=1086-94&date=2010&title=Food+Microbiology&atitle=Interactions+between+bacterial+isolates+from+modified-atmosphere-packaged+artisan-type+cooked+ham+in+view+of+the+development+of+a+bioprotective+culture.&aulast=Vasilopoulos&pid=<author>Vasilopoulos+C%3BDe+Mey+E%3BDewulf+L%3BPaelinck+H%3BDe+Smedt+A%3BVandendriessche+F%3BDe+Vuyst+L%3BLeroy+F<%2Fauthor><AN>20832689<%2FAN><DT>Journal+Article<%2FDT>)

42.

Carnobacterium maltaromaticum: identification, isolation tools, ecology and technological aspects in dairy products. [Review] [91 refs]

Afzal MI; Jacquet T; Delaunay S; Borges F; Milliere JB; Revol-Junelles AM; Cailliez-Grimal C.

Food Microbiology. 27(5):573-9, 2010 Aug.

[Journal Article. Review]

UI: 20510773

Carnobacterium species constitute a genus of Lactic Acid Bacteria (LAB) present in different ecological niches. The aim of this article is to summarize the knowledge about Carnobacterium maltaromaticum species at different microbiological levels such as taxonomy, isolation and identification, ecology, technological aspects and safety in dairy products. Works published during the last decade concerning C. maltaromaticum have shown that this non-starter LAB (NSLAB) could present major interests in dairy product technology. Four reasons can be mentioned: i) it can grow in milk during the ripening period with no competition with starter LAB, ii) this species synthesizes different flavouring compounds e.g., 3-methylbutanal, iii) it can inhibit the growth of foodborne pathogens as Listeria monocytogenes due to its ability to produce bacteriocins, iv) it has never been reported to be involved in human diseases as no cases of human infection have been directly linked to the consumption of dairy products containing this species.

Copyright 2010 Elsevier Ltd. All rights reserved. [References: 91]

Version ID

1

Record Owner

From MEDLINE, a database of the U.S. National Library of Medicine.

Status

MEDLINE

Authors Full Name

Afzal, Muhammad Inam; Jacquet, Thibaut; Delaunay, Stephane; Borges, Frederic; Milliere, Jean-Bernard; Revol-Junelles, Anne-Marie; Cailliez-Grimal, Catherine.

Institution

Afzal, Muhammad Inam. Nancy-Universite, Institut National Polytechnique de Lorraine, Laboratoire d'Ingenierie des Biomolecules, 2, avenue de Foret de Haye, B.P. 172, 54505 Vandoeuvre-les-Nancy, France.

MeSH Heading

Animals. *Bacterial Typing Techniques/mt [Methods]. Carnobacterium/cl [Classification]. Carnobacterium/ge [Genetics]. *Carnobacterium/ip [Isolation & Purification]. Carnobacterium/ph [Physiology]. Consumer Product Safety. *Dairy Products/mi [Microbiology]. Food Preservation. *Food Technology. Humans.

Year of Publication

2010

Link to the Ovid Full Text or citation:

[Click here for full text options](https://libaccess.mcmaster.ca/login?url=http://ovidsp.ovid.com/ovidweb.cgi?T=JS&CSC=Y&NEWS=N&PAGE=fulltext&D=med8&AN=20510773)

Link to the External Link Resolver:

[SFX](http://sfx.scholarsportal.info/mcmaster?sid=OVID:medline&id=pmid:20510773&id=doi:10.1016%2Fj.fm.2010.03.019&issn=0740-0020&isbn=&volume=27&issue=5&spage=573&pages=573-9&date=2010&title=Food+Microbiology&atitle=Carnobacterium+maltaromaticum%3A+identification%2C+isolation+tools%2C+ecology+and+technological+aspects+in+dairy+products.&aulast=Afzal&pid=<author>Afzal+MI%3BJacquet+T%3BDelaunay+S%3BBorges+F%3BMilliere+JB%3BRevol-Junelles+AM%3BCailliez-Grimal+C<%2Fauthor><AN>20510773<%2FAN><DT>Journal+Article<%2FDT>)

43.

Evaluation of microbial dynamics during the ripening of a traditional Taiwanese naturally fermented ham.

Tu RJ; Wu HY; Lock YS; Chen MJ.

Food Microbiology. 27(4):460-7, 2010 Jun.

[Journal Article. Research Support, Non-U.S. Gov't]

UI: 20417394

Isolation and identification of the autochthonous starter from a naturally fermented meat allows control of the fermentation process and promises microbiological safety for this specialty. Thus the purpose of this study was to identify the lactic acid bacteria and coagulase-negative cocci present in a traditional Taiwanese naturally fermented ham (TNFH) and to study the microbial dynamics at different ripening stages; the approach was a combination of conventional microbiological cultivation, polymerase chain reaction-denaturing gradient gel electrophoresis and DNA sequencing. In total, twelve different strains of lactic acid bacteria and three Staphylococcus strains were identified in the TNFH samples, whereas only 5 dominant strains were observed in the TNFH samples when the DGGE as a culture-independent method was applied. The bacterial ecology on the surface of the samples was mainly characterized by the stable presence of Lactobacillus sakei and Staphylococcus saprophyticus; nonetheless Leuconostoc mesenteroides and Carnobacterium divergens were the most abundant bacteria found in the final product. These results are also agreed with the findings of the culture-independent method. In addition, Microbacterium spp., Carnobacterium spp., Enterobacter spp., Brochothrix spp., Enterococcus spp., and Bacillus spp. were also present at the beginning of the ripening, but few bacteria were found at the center of the TNFH samples during the early ripening stages. However, after 30 days of ripening, the microbial ecology at the center of the TNFH samples paralleled that of the surface. Finally, as far as we have been able to determine, our report is the first to investigate the microbiological dynamics in fermented meat products using combination of cultivation, the Harrison disc method, DGGE and DNA sequencing as the culture-dependent method. Our report is also the first to show the presence of Staphylococcus arlettae in a fermented sausage and ham product.

Copyright 2009. Published by Elsevier Ltd.

Version ID

1

Record Owner

From MEDLINE, a database of the U.S. National Library of Medicine.

Status

MEDLINE

Authors Full Name

Tu, Rong-Jen; Wu, Hsiang-Yun; Lock, Ying-Shiang; Chen, Ming-Ju.

Institution

Tu, Rong-Jen. Livestock Research Institute, Council of Agriculture, Executive Yuan, 112 Muchang, HsinHua, Tainan, Taiwan, ROC.

MeSH Heading

Animals. Base Sequence. Colony Count, Microbial. Culture Media. *DNA, Bacterial/an [Analysis]. Electrophoresis, Gel, Pulsed-Field. Fermentation. Food Microbiology. Humans. *Lactobacillaceae/gd [Growth & Development]. Lactobacillaceae/ip [Isolation & Purification]. *Meat Products/mi [Microbiology]. Polymerase Chain Reaction. Population Dynamics. *Staphylococcus/gd [Growth & Development]. Staphylococcus/ip [Isolation & Purification]. Swine. Taiwan.

Registry Number/Name of Substance

0 (Culture Media). 0 (DNA, Bacterial).

Year of Publication

2010

Link to the Ovid Full Text or citation:

[Click here for full text options](https://libaccess.mcmaster.ca/login?url=http://ovidsp.ovid.com/ovidweb.cgi?T=JS&CSC=Y&NEWS=N&PAGE=fulltext&D=med8&AN=20417394)

Link to the External Link Resolver:

[SFX](http://sfx.scholarsportal.info/mcmaster?sid=OVID:medline&id=pmid:20417394&id=doi:10.1016%2Fj.fm.2009.12.011&issn=0740-0020&isbn=&volume=27&issue=4&spage=460&pages=460-7&date=2010&title=Food+Microbiology&atitle=Evaluation+of+microbial+dynamics+during+the+ripening+of+a+traditional+Taiwanese+naturally+fermented+ham.&aulast=Tu&pid=<author>Tu+RJ%3BWu+HY%3BLock+YS%3BChen+MJ<%2Fauthor><AN>20417394<%2FAN><DT>Journal+Article<%2FDT>)

44.

Effects of temperature and pH on the growth of bacteria isolated from blown packs of vacuum-packaged beef.

Yang X; Gill CO; Balamurugan S.

Journal of Food Protection. 72(11):2380-5, 2009 Nov.

[Journal Article. Research Support, Non-U.S. Gov't]

UI: 19903404

Bacteria recovered from the microflora of blown packs of vacuum-packaged beef were identified as Leuconostoc mesenteroides, Lactococcus lactis, Carnobacterium maltaromaticum, and Clostridium estertheticum, with L. mesenteroides predominant. Isolates of these lactic acid bacteria all grew in peptone yeast extract glucose starch broth (PYGSB) at temperatures between -2 and 30 degrees C but generally grew more slowly and over a more restricted temperature range in meat juice medium (MJM). A C. estertheticum isolate and the type strain of C. estertheticum subsp. estertheticum (ATCC 51377) both grew in PYGSB and MJM at similar rates at temperatures between -2 and 17 degrees C and grew at 20 degrees C in MJM but not in PYGSB. Square root models of the variation of the growth rate with temperature indicated that the C. maltaromaticum isolate and the C. estertheticum strains grew at similar rates that were faster than those of the other isolates at temperatures between -2 and 0 degrees C. The L. mesenteroides and L. lactis isolates grew in PYGSB at pH 5.0, but the C. maltaromaticum isolate and both strains of C. estertheticum did not grow in PYGSB at pH <or= 5.3. C. estertheticum stopped growing in MJM buffered at pH 6.5 when glucose was exhausted, although these bacteria then utilized lactate. The findings suggest that, like carnobacteria, C. estertheticum may predominate during the early stages of development of the spoilage microflora of vacuum-packaged beef but that C. estertheticum will likely be inhibited by a falling pH and so may be only a minor part of the spoilage microflora when maximum numbers are attained.

Version ID

1

Record Owner

From MEDLINE, a database of the U.S. National Library of Medicine.

Status

MEDLINE

Authors Full Name

Yang, Xianqin; Gill, Colin O; Balamurugan, Sampathkumar.

Institution

Yang, Xianqin. Agriculture and Agri-Food Canada, Lacombe Research Centre, Lacombe, Alberta, Canada T4L 1W1.

MeSH Heading

Animals. Carnobacterium/gd [Growth & Development]. Cattle. Clostridium/gd [Growth & Development]. Colony Count, Microbial. *Food Contamination/an [Analysis]. Food Handling/mt [Methods]. Food Microbiology. *Food Packaging/mt [Methods]. *Food Preservation/mt [Methods]. *Gram-Positive Bacteria/gd [Growth & Development]. Humans. Hydrogen-Ion Concentration. Kinetics. Lactococcus lactis/gd [Growth & Development]. Leuconostoc/gd [Growth & Development]. *Meat/mi [Microbiology]. Temperature. Vacuum.

Year of Publication

2009

Link to the Ovid Full Text or citation:

[Click here for full text options](https://libaccess.mcmaster.ca/login?url=http://ovidsp.ovid.com/ovidweb.cgi?T=JS&CSC=Y&NEWS=N&PAGE=fulltext&D=med7&AN=19903404)

Link to the External Link Resolver:

[SFX](http://sfx.scholarsportal.info/mcmaster?sid=OVID:medline&id=pmid:19903404&id=doi:10.4315%2F0362-028x-72.11.2380&issn=0362-028X&isbn=&volume=72&issue=11&spage=2380&pages=2380-5&date=2009&title=Journal+of+Food+Protection&atitle=Effects+of+temperature+and+pH+on+the+growth+of+bacteria+isolated+from+blown+packs+of+vacuum-packaged+beef.&aulast=Yang&pid=<author>Yang+X%3BGill+CO%3BBalamurugan+S<%2Fauthor><AN>19903404<%2FAN><DT>Journal+Article<%2FDT>)

45.

A novel packaging method with a dissolving CO(2) headspace combined with organic acids prolongs the shelf life of fresh salmon.

Schirmer BC; Heiberg R; Eie T; Moretro T; Maugesten T; Carlehog M; Langsrud S.

International Journal of Food Microbiology. 133(1-2):154-60, 2009 Jul 31.

[Journal Article. Research Support, Non-U.S. Gov't]

UI: 19523706

The aim of this study was to evaluate a novel packaging method for fresh fish and to determine its effect on the bacterial growth in fresh salmon. Fresh salmon was packed with a small amount of 100% CO(2) (gas/product ratio 0.2/1.0 v/v) and a brine solution containing various combinations of citric acid (3% w/w, pH 5), acetic acid (1% w/w, pH 5) and cinnamaldehyde (200 microg/ml). Total bacterial counts, counts of sulphur reducing bacteria, lactic acid bacteria and Enterobacteriaceae as well as the bacterial composition in the product after storage were determined. The combination of CO(2) and organic acids completely inhibited bacterial growth during 14 days of storage at 4 degrees C both in inoculation experiments and in experiments on salmon with natural background flora. CO(2), acetic acid and citric acid alone each inhibited the growth of total bacterial counts, lactic acid bacteria, sulphur reducing bacteria and Enterobacteriaceae, but effects were enhanced in combinations. The addition of cinnamaldehyde did not influence bacterial growth. Analysis of the bacterial flora of salmon inoculated with different spoilage bacteria showed that Photobacterium phosphoreum and Carnobacterium maltaromaticum remained the dominating species after inoculation while Yersinia aldovae, Aeromonas salmonicida and Shewanella putrefaciens were outcompeted by other species. In addition, lactic acid bacteria from the natural background flora grew to high numbers. Combinations of CO(2) and acetic acid reduced the relative abundance of P. phosphoreum. All CO(2) dissolved in the product, thereby creating a product with the outer appearance of a vacuum package. Further work is needed to determine consumer acceptability of acid concentrations and to implement the packaging method for industrial purposes. However, implication of this packaging method in the industry may lead to a new packaging technology, combining the advantages of vacuum packaging (low space requirement) and modified atmosphere packaging (antimicrobial effect of CO(2)).

Version ID

1

Record Owner

From MEDLINE, a database of the U.S. National Library of Medicine.

Status

MEDLINE

Authors Full Name

Schirmer, Bjorn Christian; Heiberg, Ragnhild; Eie, Thomas; Moretro, Trond; Maugesten, Tove; Carlehog, Mats; Langsrud, Solveig.

Institution

Schirmer, Bjorn Christian. Nofima Mat AS, Osloveien, As, Norway. Bjorn.Schirmer@nofima.no

MeSH Heading

*Acetic Acid. Acrolein/aa [Analogs & Derivatives]. Animals. *Bacteria/gd [Growth & Development]. *Carbon Dioxide. *Citric Acid. Cold Temperature. Colony Count, Microbial. *Food Contamination/pc [Prevention & Control]. *Food Packaging/mt [Methods]. Food Preservation/mt [Methods]. Humans. Refrigeration. *Salmon/mi [Microbiology].

Registry Number/Name of Substance

142M471B3J (Carbon Dioxide). 2968PHW8QP (Citric Acid). 7864XYD3JJ (Acrolein). Q40Q9N063P (Acetic Acid). SR60A3XG0F (cinnamic aldehyde).

Year of Publication

2009

Link to the Ovid Full Text or citation:

[Click here for full text options](https://libaccess.mcmaster.ca/login?url=http://ovidsp.ovid.com/ovidweb.cgi?T=JS&CSC=Y&NEWS=N&PAGE=fulltext&D=med7&AN=19523706)

Link to the External Link Resolver:

[SFX](http://sfx.scholarsportal.info/mcmaster?sid=OVID:medline&id=pmid:19523706&id=doi:10.1016%2Fj.ijfoodmicro.2009.05.015&issn=0168-1605&isbn=&volume=133&issue=1-2&spage=154&pages=154-60&date=2009&title=International+Journal+of+Food+Microbiology&atitle=A+novel+packaging+method+with+a+dissolving+CO(2)+headspace+combined+with+organic+acids+prolongs+the+shelf+life+of+fresh+salmon.&aulast=Schirmer&pid=<author>Schirmer+BC%3BHeiberg+R%3BEie+T%3BMoretro+T%3BMaugesten+T%3BCarlehog+M%3BLangsrud+S<%2Fauthor><AN>19523706<%2FAN><DT>Journal+Article<%2FDT>)

46.

Microbial ecology of Gorgonzola rinds and occurrence of different biotypes of Listeria monocytogenes.

Cocolin L; Nucera D; Alessandria V; Rantsiou K; Dolci P; Grassi MA; Lomonaco S; Civera T.

International Journal of Food Microbiology. 133(1-2):200-5, 2009 Jul 31.

[Journal Article. Research Support, Non-U.S. Gov't]

UI: 19477544

In this study we investigated the microbiota of Gorgonzola rinds and maturing shelf swabs collected in 5 different maturing cellars in the Northwest part of Italy, in association with the detection and characterization of Listeria monocytogenes. Culture-dependent and -independent methods were performed in order to profile the main microbial populations present on the rinds and in the maturing shelves and species-specific PCR and Pulsed Field Gel Electrophoresis (PFGE) were used to identify and type L. monocytogenes isolates. The microflora was predominated by lactic acid bacteria and coagulase negative cocci, while enterococci and yeasts were very variable between the samples. Arthrobacter sp., Carnobacterium sp., Staphylococcus sp. and Brevibacterium linens, as bacteria, and Debaryomyces hansenii, as yeast, were detected by Denaturing Gradient Gel Electrophoresis (DGGE). Cluster analysis of the DGGE profiles clearly highlighted a cellar-specific microflora. L. monocytogenes was isolated in 11.1% of the rinds and 29.4% of the swabs and the molecular characterization of the isolates suggests a route of contamination from the maturing shelves to the rinds. No correlation was found between DGGE profiles and presence or absence of L. monocytogenes.

Version ID

1

Record Owner

From MEDLINE, a database of the U.S. National Library of Medicine.

Status

MEDLINE

Authors Full Name

Cocolin, Luca; Nucera, Daniele; Alessandria, Valentina; Rantsiou, Kalliopi; Dolci, Paola; Grassi, Maria Auxilia; Lomonaco, Sara; Civera, Tiziana.

Institution

Cocolin, Luca. Di.Va.P.R.A., University of Turin, Italy. lucasimone.cocolin@unito.it

MeSH Heading

*Bacteria/ip [Isolation & Purification]. *Cheese/mi [Microbiology]. Electrophoresis. *Food Microbiology. Genetic Variation. Humans. *Listeria monocytogenes/ip [Isolation & Purification]. Polymerase Chain Reaction. *Yeasts/ip [Isolation & Purification].

Year of Publication

2009

Link to the Ovid Full Text or citation:

[Click here for full text options](https://libaccess.mcmaster.ca/login?url=http://ovidsp.ovid.com/ovidweb.cgi?T=JS&CSC=Y&NEWS=N&PAGE=fulltext&D=med7&AN=19477544)

Link to the External Link Resolver:

[SFX](http://sfx.scholarsportal.info/mcmaster?sid=OVID:medline&id=pmid:19477544&id=doi:10.1016%2Fj.ijfoodmicro.2009.05.003&issn=0168-1605&isbn=&volume=133&issue=1-2&spage=200&pages=200-5&date=2009&title=International+Journal+of+Food+Microbiology&atitle=Microbial+ecology+of+Gorgonzola+rinds+and+occurrence+of+different+biotypes+of+Listeria+monocytogenes.&aulast=Cocolin&pid=<author>Cocolin+L%3BNucera+D%3BAlessandria+V%3BRantsiou+K%3BDolci+P%3BGrassi+MA%3BLomonaco+S%3BCivera+T<%2Fauthor><AN>19477544<%2FAN><DT>Journal+Article<%2FDT>)

47.

Psychrotrophic lactic acid bacteria used to improve the safety and quality of vacuum-packaged cooked and peeled tropical shrimp and cold-smoked salmon.

Matamoros S; Leroi F; Cardinal M; Gigout F; Kasbi Chadli F; Cornet J; Prevost H; Pilett MF.

Journal of Food Protection. 72(2):365-74, 2009 Feb.

[Journal Article. Research Support, Non-U.S. Gov't]

UI: 19350982

Previously isolated lactic acid bacteria (LAB) from seafood products have been investigated for their capacity to increase the sensory shelf life of vacuum-packaged shrimp and cold-smoked salmon and to inhibit the growth of three pathogenic bacteria. Two different manufactured batches of cooked, peeled, and vacuum-packaged shrimp were inoculated with seven LAB strains separately at an initial level of 5 log CFU g-t, and the spoilage was estimated by sensory analysis after 7 and 28 days of storage at 8 degrees C. Two Leuconostoc gelidum strains greatly extended the shelf life of both batches, two Lactococcus piscium strains had a moderate effect, two bacteria were spoilers (Lactobacillus fuchuensis and Carnobacterium alterfunditum), and the last one (another Leuconostoc gelidum strain) showed highly variable results depending on the batch considered. The four strains showing the best results (two Leuconostoc gelidum and two Lactococcus piscium strains) were selected for the same experiment in cold-smoked salmon. In this product, Lactococcus piscium strains showed better inhibiting capacities, improving the sensory quality significantly at 14 and 28 days of storage. Finally, the inhibiting capacities of two strains (one Leuconostoc gelidum strain and one Lactococcus piscium strain) were tested against three pathogenic bacteria (Vibrio cholerae, Listeria monocytogenes, and Staphylococcus aureus) by challenge tests in shrimp. LAB and pathogenic bacteria were coinoculated in vacuum-packaged shrimp and enumerated during 5 weeks. Lactococcus piscium strain EU2241 was able to reduce significantly the number of Listeria monocytogenes and S. aureus organisms in the product by 2 log throughout the study for Listeria monocytogenes and up to 4 weeks for S. aureus.

Version ID

1

Record Owner

From MEDLINE, a database of the U.S. National Library of Medicine.

Status

MEDLINE

Authors Full Name

Matamoros, S; Leroi, F; Cardinal, M; Gigout, F; Kasbi Chadli, F; Cornet, J; Prevost, H; Pilett, M F.

Institution

Matamoros, S. UMR INRA 1014 SECALIM ENITIAA, Nantes, France.

MeSH Heading

Animals. Antibiosis. Colony Count, Microbial. Consumer Product Safety. Food Handling/mt [Methods]. Food Microbiology. *Food Packaging/mt [Methods]. Food Preservation/mt [Methods]. Humans. *Lactococcus/ph [Physiology]. *Leuconostoc/ph [Physiology]. Listeria monocytogenes/gd [Growth & Development]. *Penaeidae/mi [Microbiology]. *Salmon/mi [Microbiology]. *Seafood/mi [Microbiology]. Seafood/st [Standards]. *Shellfish/mi [Microbiology]. Shellfish/st [Standards]. Staphylococcus aureus/gd [Growth & Development]. Taste. Temperature. Time Factors. Vacuum. Vibrio cholerae/gd [Growth & Development].

Year of Publication

2009

Link to the Ovid Full Text or citation:

[Click here for full text options](https://libaccess.mcmaster.ca/login?url=http://ovidsp.ovid.com/ovidweb.cgi?T=JS&CSC=Y&NEWS=N&PAGE=fulltext&D=med7&AN=19350982)

Link to the External Link Resolver:

[SFX](http://sfx.scholarsportal.info/mcmaster?sid=OVID:medline&id=pmid:19350982&id=doi:10.4315%2F0362-028x-72.2.365&issn=0362-028X&isbn=&volume=72&issue=2&spage=365&pages=365-74&date=2009&title=Journal+of+Food+Protection&atitle=Psychrotrophic+lactic+acid+bacteria+used+to+improve+the+safety+and+quality+of+vacuum-packaged+cooked+and+peeled+tropical+shrimp+and+cold-smoked+salmon.&aulast=Matamoros&pid=<author>Matamoros+S%3BLeroi+F%3BCardinal+M%3BGigout+F%3BKasbi+Chadli+F%3BCornet+J%3BPrevost+H%3BPilett+MF<%2Fauthor><AN>19350982<%2FAN><DT>Journal+Article<%2FDT>)

48.

Interactions between two carnobacteriocins Cbn BM1 and Cbn B2 from Carnobacterium maltaromaticum CP5 on target bacteria and Caco-2 cells.

Jasniewski J; Cailliez-Grimal C; Chevalot I; Milliere JB; Revol-Junelles AM.

Food & Chemical Toxicology. 47(4):893-7, 2009 Apr.

[Journal Article]

UI: 19271288

Two purified class IIa carnobacteriocins Cbn BM1 and Cbn B2, from Carnobacterium maltaromaticum CP5, were evaluated for antimicrobial activity against pathogenic, spoilage and lactic acid bacteria. Then, the presence of a synergistic mode of action of these two carnobacteriocins on Listeria sp., Enterococcus sp. and Carnobacterium sp. was investigated. A synergistic mode of action between Cbn BM1 and Cbn B2 on sensitive target bacteria was demonstrated using the FIC index method. Combinations of carnobacteriocins enhanced their antibacterial activities and MICs were significantly reduced, between 2- and 15-fold, by the addition of the second bacteriocin. To improve the safety of the bacteriocins as biopreservative agents, the cytotoxicity of the combination of theses two bacteriocins was determined on Caco-2 cell line. However, these two peptides used alone or in combination, at concentration 100-fold higher than those required for antimicrobial activity, were not cytotoxic. This suggests that the two carnobacteriocins produced by C. maltaromaticum CP5 could be potential natural agents for food preservation.

Version ID

1

Record Owner

From MEDLINE, a database of the U.S. National Library of Medicine.

Status

MEDLINE

Authors Full Name

Jasniewski, Jordane; Cailliez-Grimal, Catherine; Chevalot, Isabelle; Milliere, Jean-Bernard; Revol-Junelles, Anne-Marie.

Institution

Jasniewski, Jordane. Laboratoire de Science et Genie Alimentaires, Institut National Polytechnique de Lorraine, Nancy-Universite, Vandoeuvre-les-Nancy, France.

MeSH Heading

*Bacteria/de [Drug Effects]. *Bacteriocins/pd [Pharmacology]. Caco-2 Cells. Drug Synergism. Food Preservatives/pd [Pharmacology]. Humans. Microbial Sensitivity Tests.

Registry Number/Name of Substance

0 (Bacteriocins). 0 (Food Preservatives).

Year of Publication

2009

Link to the Ovid Full Text or citation:

[Click here for full text options](https://libaccess.mcmaster.ca/login?url=http://ovidsp.ovid.com/ovidweb.cgi?T=JS&CSC=Y&NEWS=N&PAGE=fulltext&D=med7&AN=19271288)

Link to the External Link Resolver:

[SFX](http://sfx.scholarsportal.info/mcmaster?sid=OVID:medline&id=pmid:19271288&id=doi:10.1016%2Fj.fct.2009.01.025&issn=0278-6915&isbn=&volume=47&issue=4&spage=893&pages=893-7&date=2009&title=Food+%26+Chemical+Toxicology&atitle=Interactions+between+two+carnobacteriocins+Cbn+BM1+and+Cbn+B2+from+Carnobacterium+maltaromaticum+CP5+on+target+bacteria+and+Caco-2+cells.&aulast=Jasniewski&pid=<author>Jasniewski+J%3BCailliez-Grimal+C%3BChevalot+I%3BMilliere+JB%3BRevol-Junelles+AM<%2Fauthor><AN>19271288<%2FAN><DT>Journal+Article<%2FDT>)

49.

Comparison of culture-dependent and independent techniques for characterisation of the microflora of peroxyacetic acid treated, vacuum-packaged beef.

Brightwell G; Clemens R; Adam K; Urlich S; Boerema J.

Food Microbiology. 26(3):283-8, 2009 May.

[Comparative Study. Journal Article. Research Support, Non-U.S. Gov't]

UI: 19269570

The diversity of microflora associated with peroxyacetic acid (POAA) treated and untreated beef was investigated by 16S rDNA gene cloning, DGGE analysis and conventional bacterial cultivation. Following vacuum packaging, POAA treated and untreated meat samples were stored for up to 18 weeks at -1.5 degrees C. Each culture independent method showed Carnobacterium spp. to predominate on both POAA treated and untreated meat. However, 16S rDNA gene analysis also detected the presence of psychrotolerant Clostridium spp. in the POAA-treated beef. Culture-dependent analysis did not distinguish Carnobacterium spp. from Lactobacilli. Although culture-dependent analysis showed an increase in the ratio of Enterobacteriaceae to lactic acid bacteria from weeks 6-18 in the POAA treated compared with the untreated meat, the numbers of Enterobacteriaceae were significantly less on POAA treated than on untreated meat. The combination of data collected by culture-dependent and independent techniques provided the most robust approach for elucidating the efficacy of chemical sanitization of chilled vacuum-packaged beef. If conventional cultivation is used for monitoring bacterial spoilage of vacuum-packaged chilled meats it is recommended that culture methods specific for Carnobacterium and Clostridium spp. should be included in order to provide a more complete indication of microbial diversity.

Version ID

1

Record Owner

From MEDLINE, a database of the U.S. National Library of Medicine.

Status

MEDLINE

Authors Full Name

Brightwell, Gale; Clemens, Robyn; Adam, Katharine; Urlich, Shelley; Boerema, Jackie.

Institution

Brightwell, Gale. Food Metabolism and Microbiology, AgResearch, Ruakura MIRINZ Centre, Private Bag 3123, Hamilton, Waikato, New Zealand. gale.brightwell@agresearch.co.nz

MeSH Heading

Animals. Cattle. Clostridium/de [Drug Effects]. Clostridium/gd [Growth & Development]. *Colony Count, Microbial/mt [Methods]. DNA, Bacterial/ch [Chemistry]. DNA, Bacterial/ge [Genetics]. DNA, Ribosomal. *Disinfectants/pd [Pharmacology]. Electrophoresis, Polyacrylamide Gel. *Food Contamination/an [Analysis]. Food Contamination/pc [Prevention & Control]. Food Packaging. *Food Preservation/mt [Methods]. Humans. Lactobacillus/de [Drug Effects]. Lactobacillus/gd [Growth & Development]. *Meat Products/mi [Microbiology]. *Peracetic Acid/pd [Pharmacology]. Sequence Analysis, DNA. Temperature. Time Factors. Vacuum.

Registry Number/Name of Substance

0 (DNA, Bacterial). 0 (DNA, Ribosomal). 0 (Disinfectants). I6KPI2E1HD (Peracetic Acid).

Year of Publication

2009

Link to the Ovid Full Text or citation:

[Click here for full text options](https://libaccess.mcmaster.ca/login?url=http://ovidsp.ovid.com/ovidweb.cgi?T=JS&CSC=Y&NEWS=N&PAGE=fulltext&D=med7&AN=19269570)

Link to the External Link Resolver:

[SFX](http://sfx.scholarsportal.info/mcmaster?sid=OVID:medline&id=pmid:19269570&id=doi:10.1016%2Fj.fm.2008.12.010&issn=0740-0020&isbn=&volume=26&issue=3&spage=283&pages=283-8&date=2009&title=Food+Microbiology&atitle=Comparison+of+culture-dependent+and+independent+techniques+for+characterisation+of+the+microflora+of+peroxyacetic+acid+treated%2C+vacuum-packaged+beef.&aulast=Brightwell&pid=<author>Brightwell+G%3BClemens+R%3BAdam+K%3BUrlich+S%3BBoerema+J<%2Fauthor><AN>19269570<%2FAN><DT>Comparative+Study<%2FDT>)

50.

Divercin V41 from gene characterization to food applications: 1998-2008, a decade of solved and unsolved questions. [Review] [29 refs]

Rihakova J; Belguesmia Y; Petit VW; Pilet MF; Prevost H; Dousset X; Drider D.

Letters in Applied Microbiology. 48(1):1-7, 2009 Jan.

[Journal Article. Review]

UI: 19018960

The emergence of an increasing number of antibiotic resistant human clinical bacteria has been a great cause of concern for the last decades. As an example, Staphylococcus aureus isolates in the hospital environment are becoming more and more resistant to antibiotics including vancomycin which is considered as a last line of defence in treatment of Staphylococcus aureus-resistant methicillin. On the other hand, food safety is threatened by development of pathogenic bacteria including Listeria monocytogenes, Campylobacter jejuni, Salmonella enteritidis, Escherichia coli O157:H7 and Staphylococcus aureus. The use of antimicrobial peptides such as glycopeptides, semi-synthetic peptides, bacteriocins including lantibiotics offers a hope to face these clinical and food microbiology concerns. Clinical approval of new chemotherapeutic agents requires a long period of time. Research on bacteriocins has demonstrated potential use to fight against undesired foodborne pathogens but the use industrial use of bacteriocins is limited. To date only lantibiotic nisin and in class IIa bacteriocin Pediocin PA-1 are legally used as food preservative in many countries. The present minireview is focused on divercin V41 (DvnV41), a class IIa bacteriocin naturally produced by Carnobacterium divergens V41. The last decade has been the witness of intensive investigations carried out on this cationic peptide tempting to answer multiple questions covering basic and applied aspects. DvnV41 has shown a wide spectrum of activity either alone or in combination with nisin and/or polymixins (synergistic effect). This outcome indicates that Cb. divergens V41 could potentially be used for safe and efficient prevention of L. monocytogenes growth in cold smoked salmon. [References: 29]

Version ID

1

Record Owner

From MEDLINE, a database of the U.S. National Library of Medicine.

Status

MEDLINE

Authors Full Name

Rihakova, J; Belguesmia, Y; Petit, V W; Pilet, M F; Prevost, H; Dousset, X; Drider, D.

Institution

Rihakova, J. Laboratoire de Microbiologie, ENITIAA, Rue de la Geraudiere, Nantes Cedex, France.

MeSH Heading

Animals. *Anti-Bacterial Agents/pd [Pharmacology]. *Bacteriocins/ge [Genetics]. *Bacteriocins/pd [Pharmacology]. *Food Preservatives/pd [Pharmacology]. Humans. *Listeria monocytogenes/de [Drug Effects]. *Salmon/mi [Microbiology].

Registry Number/Name of Substance

0 (Anti-Bacterial Agents). 0 (Bacteriocins). 0 (Food Preservatives). 0 (divercin V41).

Year of Publication

2009

Link to the Ovid Full Text or citation:

[Click here for full text options](https://libaccess.mcmaster.ca/login?url=http://ovidsp.ovid.com/ovidweb.cgi?T=JS&CSC=Y&NEWS=N&PAGE=fulltext&D=med7&AN=19018960)

Link to the External Link Resolver:

[SFX](http://sfx.scholarsportal.info/mcmaster?sid=OVID:medline&id=pmid:19018960&id=doi:10.1111%2Fj.1472-765X.2008.02490.x&issn=0266-8254&isbn=&volume=48&issue=1&spage=1&pages=1-7&date=2009&title=Letters+in+Applied+Microbiology&atitle=Divercin+V41+from+gene+characterization+to+food+applications%3A+1998-2008%2C+a+decade+of+solved+and+unsolved+questions.&aulast=Rihakova&pid=<author>Rihakova+J%3BBelguesmia+Y%3BPetit+VW%3BPilet+MF%3BPrevost+H%3BDousset+X%3BDrider+D<%2Fauthor><AN>19018960<%2FAN><DT>Journal+Article<%2FDT>)

51.

Microbial changes and growth of Listeria monocytogenes during chilled storage of brined shrimp (Pandalus borealis).

Mejlholm O; Kjeldgaard J; Modberg A; Vest MB; Boknaes N; Koort J; Bjorkroth J; Dalgaard P.

International Journal of Food Microbiology. 124(3):250-9, 2008 Jun 10.

[Journal Article. Research Support, Non-U.S. Gov't]

UI: 18456355

Thirteen storage trials and ten challenge tests were carried out to examine microbial changes, spoilage and the potential growth of Listeria monocytogenes in brined shrimp (Pandalus borealis). Shrimp in brine as well as brined and drained shrimp in modified atmosphere packaging (MAP) were produced and studied. Different recipes were used to study the effect of preserving parameters (organic acids, pH and NaCl) on growth of microorganisms and shelf life at 7-8 degrees C or 12 degrees C. Particularly, brines with different concentrations of (i) benzoic, citric and sorbic acids or (ii) acetic, citric and lactic acids were studied. Furthermore, the effect of adding diacetate to brined shrimp was evaluated. A single batch of cooked and peeled shrimp was used to study both industrially and manually processed brined shrimp with respect to the effect of process hygiene on microbial changes and the shelf life of products. Concentrations of microorganisms on newly produced brined shrimp from an industrial scale processing line were 1.0-2.3 log (CFU g(-1)) higher than comparable concentrations in manually processed samples. This resulted in a substantially shorter shelf life and a more diverse spoilage microflora of the industrially processed brined shrimp. In addition, shelf life of brined shrimp was affected by the types and concentrations of organic acids and by the storage temperature as expected. The effect of MAP was less pronounced. Eighty-two isolates from the spoilage microflora of brined shrimp were identified and they included 53 lactic acid bacteria, 6 coagulase negative Staphylococcus spp., 18 Pseudomonas fluorescens and 5 yeast isolates. After storage at 7 degrees C, P. fluorescens, Enterococcus-like isolates, E. malodoratus, Carnobacterium maltaromaticum, coagulase negative Staphylococcus spp. and Lactobacillus sakei constituted the dominating microflora of shrimp in brines that contained benzoic, citric and sorbic acids as preservatives. L. sakei dominated the spoilage microflora of brined and drained MAP shrimp, and of brined shrimp preserved using acetic, citric and lactic acids, irrespective of packaging conditions. Shrimp in brine with benzoic, citric and sorbic acids prevented growth of L. monocytogenes during more than 40 days at 7 degrees C when the preserving parameters resembled those of commercial products. However, small changes in the preserving parameters and, particularly, reduced concentrations of benzoic acid led to growth of L. monocytogenes in brined shrimp. The present study provides significant new information on microbial changes, shelf life and growth of L. monocytogenes in brined shrimp. This information can facilitate development of new and safe brined shrimp products.

Version ID

1

Record Owner

From MEDLINE, a database of the U.S. National Library of Medicine.

Status

MEDLINE

Authors Full Name

Mejlholm, Ole; Kjeldgaard, Jette; Modberg, Anne; Vest, Mette Bohn; Boknaes, Niels; Koort, Joanna; Bjorkroth, Johanna; Dalgaard, Paw.

Institution

Mejlholm, Ole. Department of Seafood Research, DTU Aqua, Technical University of Denmark, Lyngby, Denmark. ome@difres.dk

MeSH Heading

Animals. Colony Count, Microbial. Consumer Product Safety. *Food Contamination/an [Analysis]. Food Handling/mt [Methods]. Food Microbiology. *Food Packaging/mt [Methods]. *Food Preservation/mt [Methods]. Humans. Hydrogen-Ion Concentration. Hygiene. *Listeria monocytogenes/gd [Growth & Development]. *Pandalidae/mi [Microbiology]. Refrigeration. *Shellfish/mi [Microbiology]. Time Factors. Vacuum.

Year of Publication

2008

Link to the Ovid Full Text or citation:

[Click here for full text options](https://libaccess.mcmaster.ca/login?url=http://ovidsp.ovid.com/ovidweb.cgi?T=JS&CSC=Y&NEWS=N&PAGE=fulltext&D=med7&AN=18456355)

Link to the External Link Resolver:

[SFX](http://sfx.scholarsportal.info/mcmaster?sid=OVID:medline&id=pmid:18456355&id=doi:10.1016%2Fj.ijfoodmicro.2008.03.022&issn=0168-1605&isbn=&volume=124&issue=3&spage=250&pages=250-9&date=2008&title=International+Journal+of+Food+Microbiology&atitle=Microbial+changes+and+growth+of+Listeria+monocytogenes+during+chilled+storage+of+brined+shrimp+(Pandalus+borealis).&aulast=Mejlholm&pid=<author>Mejlholm+O%3BKjeldgaard+J%3BModberg+A%3BVest+MB%3BBoknaes+N%3BKoort+J%3BBjorkroth+J%3BDalgaard+P<%2Fauthor><AN>18456355<%2FAN><DT>Journal+Article<%2FDT>)

52.

Genetic and biochemical characterization of CAD-1, a chromosomally encoded new class A penicillinase from Carnobacterium divergens.

Meziane-Cherif D; Decre D; Hoiby EA; Courvalin P; Perichon B.

Antimicrobial Agents & Chemotherapy. 52(2):551-6, 2008 Feb.

[Journal Article. Research Support, Non-U.S. Gov't]

UI: 18070972

Carnobacterium divergens clinical isolates BM4489 and BM4490 were resistant to penicillins but remained susceptible to combinations of amoxicillin-clavulanic acid and piperacillin-tazobactam. Cloning and sequencing of the responsible determinant from BM4489 revealed a coding sequence of 912 bp encoding a class A beta-lactamase named CAD-1. The bla(CAD-1) gene was assigned to a chromosomal location in the two strains that had distinct pulsed-field gel electrophoresis patterns. CAD-1 shared 53% and 42% identity with beta-lactamases from Bacillus cereus and Staphylococcus aureus, respectively. Alignment of CAD-1 with other class A beta-lactamases indicated the presence of 25 out of the 26 isofunctional amino acids in class A beta-lactamases. Escherichia coli harboring bla(CAD-1) exhibited resistance to penams (benzylpenicillin and amoxicillin) and remained susceptible to amoxicillin in combination with clavulanic acid. Mature CAD-1 consisted of a 34.4-kDa polypeptide. Kinetic analysis indicated that CAD-1 exhibited a narrow substrate profile, hydrolyzing benzylpenicillin, ampicillin, and piperacillin with catalytic efficiencies of 6,600, 3,200, and 2,900 mM(-1) s(-1), respectively. The enzyme did not interact with oxyiminocephalosporins, imipenem, or aztreonam. CAD-1 was inhibited by tazobactam (50% inhibitory concentration [IC(50)] = 0.27 microM), clavulanic acid (IC(50) = 4.7 microM), and sulbactam (IC(50) = 43.5 microM). The bla(CAD-1) gene is likely to have been acquired by BM4489 and BM4490 as part of a mobile genetic element, since it was not found in the susceptible type strain CIP 101029 and was adjacent to a gene for a resolvase.

Version ID

1

Record Owner

From MEDLINE, a database of the U.S. National Library of Medicine.

Status

MEDLINE

Authors Full Name

Meziane-Cherif, Djalal; Decre, Dominique; Hoiby, E Arne; Courvalin, Patrice; Perichon, Bruno.

Institution

Meziane-Cherif, Djalal. Unite des Agents Antibacteriens, Institut Pasteur, 25 Rue du Docteur Roux, 75724 Paris Cedex 15, France.

MeSH Heading

Amino Acid Sequence. *Chromosomes, Bacterial/ge [Genetics]. Cloning, Molecular. Electrophoresis, Gel, Pulsed-Field. Gram-Positive Bacteria/de [Drug Effects]. *Gram-Positive Bacteria/en [Enzymology]. Gram-Positive Bacteria/ge [Genetics]. Gram-Positive Bacteria/ip [Isolation & Purification]. *Gram-Positive Bacterial Infections/mi [Microbiology]. Humans. Infant, Newborn. Kinetics. Microbial Sensitivity Tests. Molecular Sequence Data. Penicillin Resistance. Penicillinase/ch [Chemistry]. Penicillinase/ge [Genetics]. Penicillinase/me [Metabolism]. *Penicillinase. Sequence Alignment. Sequence Analysis, DNA. Substrate Specificity. beta-Lactams/pd [Pharmacology].

Registry Number/Name of Substance

0 (beta-Lactams). EC 3-5-2 (Penicillinase).

Year of Publication

2008

Link to the Ovid Full Text or citation:

[Click here for full text options](https://libaccess.mcmaster.ca/login?url=http://ovidsp.ovid.com/ovidweb.cgi?T=JS&CSC=Y&NEWS=N&PAGE=fulltext&D=med7&AN=18070972)

Link to the External Link Resolver:

[SFX](http://sfx.scholarsportal.info/mcmaster?sid=OVID:medline&id=pmid:18070972&id=doi:10.1128%2FAAC.01145-07&issn=0066-4804&isbn=&volume=52&issue=2&spage=551&pages=551-6&date=2008&title=Antimicrobial+Agents+%26+Chemotherapy&atitle=Genetic+and+biochemical+characterization+of+CAD-1%2C+a+chromosomally+encoded+new+class+A+penicillinase+from+Carnobacterium+divergens.&aulast=Meziane-Cherif&pid=<author>Meziane-Cherif+D%3BDecre+D%3BHoiby+EA%3BCourvalin+P%3BPerichon+B<%2Fauthor><AN>18070972<%2FAN><DT>Journal+Article<%2FDT>)

53.

Quality changes during refrigerated storage of MA-packaged pre-rigor fillets of farmed Atlantic cod (Gadus morhua L.) using traditional MAP, CO2 emitter, and vacuum.

Hansen AA; Morkore T; Rudi K; Olsen E; Eie T.

Journal of Food Science. 72(9):M423-30, 2007 Nov.

[Evaluation Study. Journal Article. Research Support, Non-U.S. Gov't]

UI: 18034737

Quality changes during 3 wk of refrigerated storage (1.3 degrees C) were studied on pre-rigor filleted farmed Atlantic cod packed in modified atmosphere (MAP, 60% CO2 and 40% O2) or vacuum. The packages of MAP contained either a CO2 emitter and low gas volume to product volume (g/p ratio) of 1.3, or a 3.9 g/p ratio and no emitter. The CO2 level remained stable or increased in the packages with CO2 emitter, whereas the CO2 level in the packages with no CO2 emitter decreased to 40% after 4 d of refrigerated storage. High levels of oxygen in the gas mixture prevented formation of trimethyl amine (TMA) during storage of the MA-packed fish, whereas the TMA content increased significantly after 10-d storage in vacuum. MA-packed samples had the highest values of 1-penten-3-ol. Sensory scores of sour, sulfur, and pungent odors were significantly higher for vacuum-packed cod compared to the 2 MA-packaging methods measured 14 d after slaughtering. No differences in sensory scores were observed between the 2 methods of MAP, and shelf life of these samples seemed to be 14 to 21 d. Cod samples packaged in vacuum packages had higher pH values compared to ordinary MAP and packages containing a CO2 emitter. Bacterial growth was inhibited by MAP and resulted at the end of the storage period in dominance of Carnobacterium and some Photobacterium. In MA packages with high O2 levels the Photobacterium was inhibited. It is concluded that CO2 emitters are well suited for reduction of transport volume for MA-packaged farmed cod.

Version ID

1

Record Owner

From MEDLINE, a database of the U.S. National Library of Medicine.

Status

MEDLINE

Authors Full Name

Hansen, A A; Morkore, T; Rudi, K; Olsen, E; Eie, T.

Institution

Hansen, A A. MATFORSK AS, Norwegian Food Research Inst., N-1430 As, Norway. anlaug.adland@matforsk.no

MeSH Heading

Analysis of Variance. Animals. *Carbon Dioxide/me [Metabolism]. Colony Count, Microbial. *Food Handling/mt [Methods]. Food Packaging/is [Instrumentation]. *Food Packaging/mt [Methods]. *Gadus morhua. Humans. Hydrogen-Ion Concentration. Methylamines/me [Metabolism]. Odorants. *Oxygen/me [Metabolism]. Photobacterium/gd [Growth & Development]. Quality Control. *Refrigeration. Smell/ph [Physiology]. Time Factors. Vacuum.

Registry Number/Name of Substance

0 (Methylamines). 142M471B3J (Carbon Dioxide). LHH7G8O305 (trimethylamine). S88TT14065 (Oxygen).

Year of Publication

2007

Link to the Ovid Full Text or citation:

[Click here for full text options](https://libaccess.mcmaster.ca/login?url=http://ovidsp.ovid.com/ovidweb.cgi?T=JS&CSC=Y&NEWS=N&PAGE=fulltext&D=med6&AN=18034737)

Link to the External Link Resolver:

[SFX](http://sfx.scholarsportal.info/mcmaster?sid=OVID:medline&id=pmid:18034737&id=doi:10.1111%2Fj.1750-3841.2007.00561.x&issn=0022-1147&isbn=&volume=72&issue=9&spage=M423&pages=M423-30&date=2007&title=Journal+of+Food+Science&atitle=Quality+changes+during+refrigerated+storage+of+MA-packaged+pre-rigor+fillets+of+farmed+Atlantic+cod+(Gadus+morhua+L.)+using+traditional+MAP%2C+CO2+emitter%2C+and+vacuum.&aulast=Hansen&pid=<author>Hansen+AA%3BMorkore+T%3BRudi+K%3BOlsen+E%3BEie+T<%2Fauthor><AN>18034737<%2FAN><DT>Evaluation+Study<%2FDT>)

54.

Spoilage of value-added, high-oxygen modified-atmosphere packaged raw beef steaks by Leuconostoc gasicomitatum and Leuconostoc gelidum.

Vihavainen EJ; Bjorkroth KJ.

International Journal of Food Microbiology. 119(3):340-5, 2007 Nov 01.

[Journal Article. Research Support, Non-U.S. Gov't]

UI: 17913272

Moisture-enhancing and marinating of meats are commonly used by the meat industry to add value to raw, retail products. Recently in Finland, certain value-added beef steak products have proven to be unusually susceptible to microbial spoilage leading to untoward quality deteriorations during producer-defined shelf-life. This study was conducted to evaluate the role of lactic acid bacteria (LAB) in the premature spoilage of value-added beef packaged under high-oxygen modified atmospheres. Spoilage was characterised by green discolouration and a buttery off-odour. The predominant LAB in eight packages of spoiled, marinated or moisture-enhanced beef steaks were identified by reference to a 16 and 23S rRNA gene restriction fragment length polymorphism pattern (ribotype) database. Leuconostoc gasicomitatum, Leuconostoc gelidum, Lactobacillus algidus, Lactobacillus sakei and Carnobacterium divergens were found to predominate in the LAB populations at numbers above 10(8) CFU/g. Inoculation of moisture-enhanced steaks with LAB strains and strain mixtures originating from the spoiled products demonstrated the spoilage potential of L. gasicomitatum and L. gelidum isolates. These two species produced green surface discolouration and buttery off-odours similar to these found in the spoiled, commercial products.

Version ID

1

Record Owner

From MEDLINE, a database of the U.S. National Library of Medicine.

Status

MEDLINE

Authors Full Name

Vihavainen, Elina J; Bjorkroth, K Johanna.

Institution

Vihavainen, Elina J. Department of Food and Environmental Hygiene, Faculty of Veterinary Medicine, FIN-00014, University of Helsinki, Finland. elina.vihavainen@helsinki.fi

MeSH Heading

Animals. Cattle. Colony Count, Microbial. *Food Contamination/an [Analysis]. Food Microbiology. *Food Packaging/mt [Methods]. Humans. Leuconostoc/cl [Classification]. Leuconostoc/ge [Genetics]. *Leuconostoc/gd [Growth & Development]. Leuconostoc/ip [Isolation & Purification]. *Meat/mi [Microbiology]. Odorants/an [Analysis]. *Oxygen/me [Metabolism]. Phylogeny. Polymorphism, Restriction Fragment Length. RNA, Ribosomal, 16S/ge [Genetics]. RNA, Ribosomal, 23S/ge [Genetics]. Ribotyping.

Registry Number/Name of Substance

0 (RNA, Ribosomal, 16S). 0 (RNA, Ribosomal, 23S). S88TT14065 (Oxygen).

Year of Publication

2007

Link to the Ovid Full Text or citation:

[Click here for full text options](https://libaccess.mcmaster.ca/login?url=http://ovidsp.ovid.com/ovidweb.cgi?T=JS&CSC=Y&NEWS=N&PAGE=fulltext&D=med6&AN=17913272)

Link to the External Link Resolver:

[SFX](http://sfx.scholarsportal.info/mcmaster?sid=OVID:medline&id=pmid:17913272&id=doi:10.1016%2Fj.ijfoodmicro.2007.08.029&issn=0168-1605&isbn=&volume=119&issue=3&spage=340&pages=340-5&date=2007&title=International+Journal+of+Food+Microbiology&atitle=Spoilage+of+value-added%2C+high-oxygen+modified-atmosphere+packaged+raw+beef+steaks+by+Leuconostoc+gasicomitatum+and+Leuconostoc+gelidum.&aulast=Vihavainen&pid=<author>Vihavainen+EJ%3BBjorkroth+KJ<%2Fauthor><AN>17913272<%2FAN><DT>Journal+Article<%2FDT>)

55.

Carnobacterium: positive and negative effects in the environment and in foods. [Review] [222 refs]

Leisner JJ; Laursen BG; Prevost H; Drider D; Dalgaard P.

FEMS Microbiology Reviews. 31(5):592-613, 2007 Sep.

[Journal Article. Review]

UI: 17696886

The genus Carnobacterium contains nine species, but only C. divergens and C. maltaromaticum are frequently isolated from natural environments and foods. They are tolerant to freezing/thawing and high pressure and able to grow at low temperatures, anaerobically and with increased CO(2) concentrations. They metabolize arginine and various carbohydrates, including chitin, and this may improve their survival in the environment. Carnobacterium divergens and C. maltaromaticum have been extensively studied as protective cultures in order to inhibit growth of Listeria monocytogenes in fish and meat products. Several carnobacterial bacteriocins are known, and parameters that affect their production have been described. Currently, however, no isolates are commercially applied as protective cultures. Carnobacteria can spoil chilled foods, but spoilage activity shows intraspecies and interspecies variation. The responsible spoilage metabolites are not well characterized, but branched alcohols and aldehydes play a partial role. Their production of tyramine in foods is critical for susceptible individuals, but carnobacteria are not otherwise human pathogens. Carnobacterium maltaromaticum can be a fish pathogen, although carnobacteria are also suggested as probiotic cultures for use in aquaculture. Representative genome sequences are not yet available, but would be valuable to answer questions associated with fundamental and applied aspects of this important genus. [References: 222]

Version ID

1

Record Owner

From MEDLINE, a database of the U.S. National Library of Medicine.

Status

MEDLINE

Authors Full Name

Leisner, Jorgen J; Laursen, Birgit Groth; Prevost, Herve; Drider, Djamel; Dalgaard, Paw.

Institution

Leisner, Jorgen J. Department of Veterinary Pathobiology, Faculty of Life Sciences, University of Copenhagen, Gronnegardsvej 15, DK-1870 Frederiksberg C., Denmark. jjl@life.ku.dk

MeSH Heading

Animals. *Environmental Microbiology. *Food Microbiology. Gram-Positive Bacteria/ch [Chemistry]. Gram-Positive Bacteria/ip [Isolation & Purification]. *Gram-Positive Bacteria/py [Pathogenicity]. Humans.

Year of Publication

2007

Link to the Ovid Full Text or citation:

[Click here for full text options](https://libaccess.mcmaster.ca/login?url=http://ovidsp.ovid.com/ovidweb.cgi?T=JS&CSC=Y&NEWS=N&PAGE=fulltext&D=med6&AN=17696886)

Link to the External Link Resolver:

[SFX](http://sfx.scholarsportal.info/mcmaster?sid=OVID:medline&id=pmid:17696886&id=doi:10.1111%2Fj.1574-6976.2007.00080.x&issn=0168-6445&isbn=&volume=31&issue=5&spage=592&pages=592-613&date=2007&title=FEMS+Microbiology+Reviews&atitle=Carnobacterium%3A+positive+and+negative+effects+in+the+environment+and+in+foods.&aulast=Leisner&pid=<author>Leisner+JJ%3BLaursen+BG%3BPrevost+H%3BDrider+D%3BDalgaard+P<%2Fauthor><AN>17696886<%2FAN><DT>Journal+Article<%2FDT>)

56.

Limitations in the use of Drosophila melanogaster as a model host for gram-positive bacterial infection.

Jensen RL; Pedersen KS; Loeschcke V; Ingmer H; Leisner JJ.

Letters in Applied Microbiology. 44(2):218-23, 2007 Feb.

[Journal Article. Research Support, Non-U.S. Gov't]

UI: 17257264

AIMS: To examine sensitivities of various Drosophila melanogaster strains towards human pathogenic and nonpathogenic gram-positive bacteria.

METHODS AND RESULTS: The D. melanogaster Oregon R strain was infected by injecting the thorax with a needle containing Escherichia coli (negative control), Listeria monocytogenes, Staphylococcus aureus (both food-borne pathogens), Listeria innocua, Bacillus subtilis, Carnobacterium maltaromaticum, Lactobacillus plantarum or Pediococcus acidilactici (all nonpathogenic bacteria). Listeria monocytogenes and S. aureus killed the host rapidly compared with the negative control. Infection with L. innocua, B. subtilis or C. maltaromaticum also resulted in a high fly mortality, whereas Lact. plantarum and P. acidilactici resulted in a slightly increased mortality. Four additional D. melanogaster lines, three of which had been selected for heat, cold and desiccation resistance respectively, were subjected to infection by L. monocytogenes, S. aureus and E. coli. Mortality rates were comparable with that of the Oregon R strain.

CONCLUSIONS: Use of the injection method shows the limitation of D. melanogaster as a model host for gram-positive bacteria as opportunistic infection by nonpathogenic gram-positive bacteria results in partial or high mortality. In addition, lines of fruit flies resistant to various stress exposures did not show an increased resistance to infection by gram-positive pathogens under the conditions tested.

SIGNIFICANCE AND IMPACT OF THE STUDY: This study demonstrates the inadequacy of D. melanogaster infected by the injection method in order to distinguish between virulent and nonvirulent gram-positive bacteria.

Version ID

1

Record Owner

From MEDLINE, a database of the U.S. National Library of Medicine.

Status

MEDLINE

Authors Full Name

Jensen, R L; Pedersen, K S; Loeschcke, V; Ingmer, H; Leisner, J J.

Institution

Jensen, R L. Department of Veterinary Pathobiology, Royal Veterinary and Agricultural University, Copenhagen, Denmark.

MeSH Heading

Animals. *Disease Models, Animal. *Drosophila melanogaster/mi [Microbiology]. *Gram-Positive Bacterial Infections/ve [Veterinary].

Year of Publication

2007

Link to the Ovid Full Text or citation:

[Click here for full text options](https://libaccess.mcmaster.ca/login?url=http://ovidsp.ovid.com/ovidweb.cgi?T=JS&CSC=Y&NEWS=N&PAGE=fulltext&D=med6&AN=17257264)

Link to the External Link Resolver:

[SFX](http://sfx.scholarsportal.info/mcmaster?sid=OVID:medline&id=pmid:17257264&id=doi:10.1111%2Fj.1472-765X.2006.02040.x&issn=0266-8254&isbn=&volume=44&issue=2&spage=218&pages=218-23&date=2007&title=Letters+in+Applied+Microbiology&atitle=Limitations+in+the+use+of+Drosophila+melanogaster+as+a+model+host+for+gram-positive+bacterial+infection.&aulast=Jensen&pid=<author>Jensen+RL%3BPedersen+KS%3BLoeschcke+V%3BIngmer+H%3BLeisner+JJ<%2Fauthor><AN>17257264<%2FAN><DT>Journal+Article<%2FDT>)

57.

Lactic acid bacteria associated with vacuum-packed cooked meat product spoilage: population analysis by rDNA-based methods.

Chenoll E; Macian MC; Elizaquivel P; Aznar R.

Journal of Applied Microbiology. 102(2):498-508, 2007 Feb.

[Journal Article. Research Support, Non-U.S. Gov't]

UI: 17241356

AIM: To determine the lactic acid bacteria (LAB) implicated in bloating spoilage of vacuum-packed and refrigerated meat products.

METHODS AND RESULTS: A total of 18 samples corresponding to four types of meat products, with and without spoilage symptoms, were studied. In all, 387 colonies growing on de Man, Rogosa and Sharpe, yeast glucose lactose peptone and trypticase soy yeast extract plates were identified by internal spacer region (ISR), ISR-restriction fragment length polymorphism and rapid amplified ribosomal DNA restriction analysis profiles as Lactobacillus (37%), Leuconostoc (43%), Carnobacterium (11%), Enterococcus (4%) and Lactococcus (2%). Leuconostoc mesenteroides dominated the microbial population of spoiled products and was always present at the moment bloating occurred. Lactobacillus sakei, Lactobacillus plantarum and Lactobacillus curvatus were found in decreasing order of abundance. The analysis of two meat products, 'morcilla' and 'fiambre de magro adobado' obtained from production lines revealed a common succession pattern in LAB populations in both products and showed that Leuc. mesenteroides became the main species during storage, despite being below the detection level of culture methods after packing.

CONCLUSIONS: Our results pointed to Leuc. mesenteroides as the main species responsible for bloating spoilage in vacuum-packed meat products.

SIGNIFICANCE AND IMPACT OF THE STUDY: Prevention of bloating spoilage in vacuum-packed cooked meat products requires the sensitive detection of Leuc. mesenteroides (i.e. by PCR).

Version ID

1

Record Owner

From MEDLINE, a database of the U.S. National Library of Medicine.

Status

MEDLINE

Authors Full Name

Chenoll, E; Macian, M C; Elizaquivel, P; Aznar, R.

Institution

Chenoll, E. Departamento de Microbiologia, University of Valencia, Valencia, Spain.

MeSH Heading

Animals. Cooking. *DNA, Bacterial/an [Analysis]. Electronic Data Processing. *Food Microbiology. Food Packaging. *Food Preservation/mt [Methods]. Humans. *Lactobacillus/ge [Genetics]. *Leuconostoc/ge [Genetics]. *Meat Products/mi [Microbiology]. Poultry Products/mi [Microbiology]. Ribotyping. Swine. Turkeys. Vacuum.

Registry Number/Name of Substance

0 (DNA, Bacterial).

Year of Publication

2007

Link to the Ovid Full Text or citation:

[Click here for full text options](https://libaccess.mcmaster.ca/login?url=http://ovidsp.ovid.com/ovidweb.cgi?T=JS&CSC=Y&NEWS=N&PAGE=fulltext&D=med6&AN=17241356)

Link to the External Link Resolver:

[SFX](http://sfx.scholarsportal.info/mcmaster?sid=OVID:medline&id=pmid:17241356&id=doi:10.1111%2Fj.1365-2672.2006.03081.x&issn=1364-5072&isbn=&volume=102&issue=2&spage=498&pages=498-508&date=2007&title=Journal+of+Applied+Microbiology&atitle=Lactic+acid+bacteria+associated+with+vacuum-packed+cooked+meat+product+spoilage%3A+population+analysis+by+rDNA-based+methods.&aulast=Chenoll&pid=<author>Chenoll+E%3BMacian+MC%3BElizaquivel+P%3BAznar+R<%2Fauthor><AN>17241356<%2FAN><DT>Journal+Article<%2FDT>)

58.

Inhibition of Listeria innocua growth by antimicrobial-producing lactic acid cultures in vacuum-packed cold-smoked salmon.

Vescovo M; Scolari G; Zacconi C.

Food Microbiology. 23(7):689-93, 2006 Oct.

[Journal Article]

UI: 16943070

The biopreservative potential of three antimicrobial-producing lactic acid bacteria strains was evaluated on cold-smoked salmon. Lactobacillus casei, Lactobacillus plantarum and Carnobacterium piscicola were added singly or in association to cold-smoked salmon, artificially contaminated with Listeria innocua and stored under vacuum for 30 days at 4 degrees C. All the lactic cultures were able to inhibit Listeria innocua growth, showing a bacteriostatic or bactericidal effect, without affecting negatively the sensory quality of the product. Lactobacillus casei was bacteriostatic when inoculated at 6 log cfu/g, but bactericidal at 8 log cfu/g, reducing Listeria innocua of 3.3 log cfu/g in comparison with the test at the end of storage. Lactobacillus plantarum and C. piscicola strains, inoculated singly at 6 log cfu/g reduced Listeria innocua counts of 2.8 and 2.7 log cfu/g, respectively, compared with the test. The association Lactobacillus casei-Lactobacillus plantarum was the most effective among the treatments with 6 log cfu/g inoculum, as Listeria innocua counts decreased of 3.2 log cfu/g compared with the test. The treatment with Lactobacillus casei-C. piscicola association was less effective than C. piscicola alone.

Version ID

1

Record Owner

From MEDLINE, a database of the U.S. National Library of Medicine.

Status

MEDLINE

Authors Full Name

Vescovo, Marisa; Scolari, Gianluigi; Zacconi, Carla.

Institution

Vescovo, Marisa. Istituto di Microbiologia, Universita Cattolica del Sacro Cuore, Via Emilia Parmense, 84, 29100 Piacenza, Italy. marisa.vescovo@unicatt.it

MeSH Heading

Animals. Antibiosis. Colony Count, Microbial. *Food Contamination/an [Analysis]. *Food Packaging/mt [Methods]. *Food Preservation/mt [Methods]. Humans. Lactic Acid/bi [Biosynthesis]. Lactic Acid/pd [Pharmacology]. Lactobacillus/me [Metabolism]. *Lactobacillus/ph [Physiology]. Lactobacillus casei/me [Metabolism]. Lactobacillus casei/ph [Physiology]. Lactobacillus plantarum/me [Metabolism]. Lactobacillus plantarum/ph [Physiology]. Listeria/de [Drug Effects]. *Listeria/gd [Growth & Development]. *Salmon/mi [Microbiology]. *Seafood/mi [Microbiology]. Temperature. Time Factors. Vacuum.

Registry Number/Name of Substance

33X04XA5AT (Lactic Acid).

Year of Publication

2006

Link to the Ovid Full Text or citation:

[Click here for full text options](https://libaccess.mcmaster.ca/login?url=http://ovidsp.ovid.com/ovidweb.cgi?T=JS&CSC=Y&NEWS=N&PAGE=fulltext&D=med6&AN=16943070)

Link to the External Link Resolver:

[SFX](http://sfx.scholarsportal.info/mcmaster?sid=OVID:medline&id=pmid:16943070&id=doi:10.1016%2Fj.fm.2005.12.002&issn=0740-0020&isbn=&volume=23&issue=7&spage=689&pages=689-93&date=2006&title=Food+Microbiology&atitle=Inhibition+of+Listeria+innocua+growth+by+antimicrobial-producing+lactic+acid+cultures+in+vacuum-packed+cold-smoked+salmon.&aulast=Vescovo&pid=<author>Vescovo+M%3BScolari+G%3BZacconi+C<%2Fauthor><AN>16943070<%2FAN><DT>Journal+Article<%2FDT>)

59.

Eggshell factors influencing eggshell penetration and whole egg contamination by different bacteria, including Salmonella enteritidis.

De Reu K; Grijspeerdt K; Messens W; Heyndrickx M; Uyttendaele M; Debevere J; Herman L.

International Journal of Food Microbiology. 112(3):253-60, 2006 Dec 01.

[Journal Article. Research Support, Non-U.S. Gov't]

UI: 16822571

Trans-shell infection routes and whole egg contamination of 7 selected bacterial strains; Staphylococcus warneri, Acinetobacter baumannii, Alcaligenes sp., Serratia marcescens, Carnobacterium sp., Pseudomonas sp. and Salmonella enteritidis, recovered from egg contents, were studied. The first objective was to correlate bacterial eggshell penetration with various eggshell characteristics and bacterial strains. An agar approach was used to assess the eggshell penetration. The second objective was to assess the contamination of whole eggs with the bacterial strains; whole intact eggs were used in this case. The intact shells of agar-filled and whole eggs were inoculated with 10(3) -10(4) cfu of the selected strains. During 3 weeks storage at 20 degrees C and 60% relative humidity, the bacterial eggshell penetration was regularly monitored. The whole egg contamination was only analyzed after 3 weeks. The eggshell characteristics such as area eggshell, shell thickness and number of pores did not influence the bacterial eggshell penetration. For each individual bacterial strain the mean cuticle deposition was lower for penetrated compared to non-penetrated eggshells. For the individual strain Carnobacterium sp. and for the global results of all strains this difference was statistical significantly. The whole egg contamination was not influenced by neither the area of the eggshell nor the porosity of the eggshell. The results of the agar approach indicate that the Gram-negative, motile and non-clustering bacteria penetrated the eggshell most frequently; Pseudomonas sp. (60%) and Alcaligenes sp. (58%) were primary invaders followed by S. enteritidis (43%). All selected strains were able to penetrate; penetration was observed most frequently after ca. 4-5 days. Particularly S. enteritidis was a primary invader of whole eggs: the membranes and/or the content of 32% of the whole eggs was contaminated. The remaining bacterial eggshell contamination with the selected strain was determined after 3 weeks storage. Penetrated eggshells and contaminated whole eggs showed a significantly higher bacterial contamination on the eggshell compared to non-penetrated eggshells and non-contaminated whole eggs respectively (global results of all strains). The influence of hen age on bacterial eggshell penetration and egg content contamination was not significant. While the agar approach is suitable to study the influence of the eggshell characteristics on the bacterial eggshell penetration, the intact egg approach gives an estimation of the penetration of the shell followed by the probability of survival and migration in whole eggs.

Version ID

1

Record Owner

From MEDLINE, a database of the U.S. National Library of Medicine.

Status

MEDLINE

Authors Full Name

De Reu, K; Grijspeerdt, K; Messens, W; Heyndrickx, M; Uyttendaele, M; Debevere, J; Herman, L.

Institution

De Reu, K. Department of Animal Product Quality and Transformation Technology, Agricultural Research Centre-Ghent, Ministry of the Flemish Community, Brusselsesteenweg 370, 9090 Melle, Belgium. K.Dereu@clo.fgov.be

MeSH Heading

Animals. Colony Count, Microbial. Consumer Product Safety. Egg Shell/ch [Chemistry]. *Egg Shell/mi [Microbiology]. *Food Contamination/an [Analysis]. Food Handling/mt [Methods]. *Food Microbiology. *Food Preservation/mt [Methods]. Humans. Humidity. Permeability. *Salmonella enteritidis/gd [Growth & Development]. Salmonella enteritidis/py [Pathogenicity]. Salmonella enteritidis/ph [Physiology]. Temperature. Time Factors.

Year of Publication

2006

Link to the Ovid Full Text or citation:

[Click here for full text options](https://libaccess.mcmaster.ca/login?url=http://ovidsp.ovid.com/ovidweb.cgi?T=JS&CSC=Y&NEWS=N&PAGE=fulltext&D=med6&AN=16822571)

Link to the External Link Resolver:

[SFX](http://sfx.scholarsportal.info/mcmaster?sid=OVID:medline&id=pmid:16822571&id=doi:10.1016%2Fj.ijfoodmicro.2006.04.011&issn=0168-1605&isbn=&volume=112&issue=3&spage=253&pages=253-60&date=2006&title=International+Journal+of+Food+Microbiology&atitle=Eggshell+factors+influencing+eggshell+penetration+and+whole+egg+contamination+by+different+bacteria%2C+including+Salmonella+enteritidis.&aulast=De+Reu&pid=<author>De+Reu+K%3BGrijspeerdt+K%3BMessens+W%3BHeyndrickx+M%3BUyttendaele+M%3BDebevere+J%3BHerman+L<%2Fauthor><AN>16822571<%2FAN><DT>Journal+Article<%2FDT>)

60.

Enterococcus species dominating in fresh modified-atmosphere-packaged, marinated broiler legs are overgrown by Carnobacterium and Lactobacillus species during storage at 6 degrees C.

Bjorkroth J; Ristiniemi M; Vandamme P; Korkeala H.

International Journal of Food Microbiology. 97(3):267-76, 2005 Jan 01.

[Journal Article]

UI: 15582737

In order to show which of the initial lactic acid bacteria (LAB) contaminants are also causing spoilage of a modified-atmosphere-packaged (MAP), marinated broiler leg product at 6 degrees C, LAB were enumerated and identified on the 2nd and 17th days following manufacture. A total of 8 fresh and 13 spoiled packages were studied for LAB levels. In addition, aerobic mesophilic bacteria and Enterobacteriaceae were determined. The average CFU/g values in the 8 fresh packages were 1.3 x 10(3), 9.8 x 10(3) and 2.6 x 10(2) on de Man Rogosa Sharpe agar (MRS), Plate Count Agar (PCA) and Violet Red Bile Glucose agar (VRBG), respectively. The commercial shelf life for the product had been set as 12 days, and all packages analyzed on the 17th day were deemed unfit for human consumption by sensory analysis. The corresponding CFU/g averages in the spoiled product were 1.4 x 10(9), 1.1 x 10(9) and 3.9 x 10(7) on MRS, PCA and VRBG agar, respectively. For characterization of LAB population, 104 colonies originating from the fresh packages and 144 colonies from the spoiled packages were randomly picked, cultured pure and identified to species level using a 16 and 23S rDNA HindIII RFLP (ribotyping) database. The results showed that enterococci (35.7% of the LAB population) were dominating in the fresh product, whereas carnobacteria (59.7%) dominated among the spoilage LAB. Enterococcus faecalis, Carnobacterium piscicola and Carnobacterium divergens were the main species detected. In general, when the initial LAB population is compared to the spoilage LAB, a shift from homofermentative cocci towards carnobacteria, Lactobacillus sakei/curvatus and heterofermentative rods is seen in this marinated product.

Version ID

1

Record Owner

From MEDLINE, a database of the U.S. National Library of Medicine.

Status

MEDLINE

Authors Full Name

Bjorkroth, Johanna; Ristiniemi, Minna; Vandamme, Peter; Korkeala, Hannu.

Institution

Bjorkroth, Johanna. Department of Food and Environmental Hygiene, Faculty of Veterinary Medicine, University of Helsinki, P.O. Box 57, FIN-00014 Helsinki, Finland. johanna.bjorkroth@helsinki.fi

MeSH Heading

Animals. Chickens. Colony Count, Microbial. Enterococcus/cl [Classification]. *Enterococcus/ip [Isolation & Purification]. *Food Contamination/an [Analysis]. *Food Handling/mt [Methods]. Food Microbiology. Food Packaging. Food Preservation/mt [Methods]. Lactobacillus/cl [Classification]. *Lactobacillus/ip [Isolation & Purification]. Leuconostoc/cl [Classification]. Leuconostoc/ip [Isolation & Purification]. *Meat/mi [Microbiology]. Phylogeny. Ribotyping. Temperature. Time Factors.

Year of Publication

2005

Link to the Ovid Full Text or citation:

[Click here for full text options](https://libaccess.mcmaster.ca/login?url=http://ovidsp.ovid.com/ovidweb.cgi?T=JS&CSC=Y&NEWS=N&PAGE=fulltext&D=med6&AN=15582737)

Link to the External Link Resolver:

[SFX](http://sfx.scholarsportal.info/mcmaster?sid=OVID:medline&id=pmid:15582737&id=doi:10.1016%2Fj.ijfoodmicro.2004.04.011&issn=0168-1605&isbn=&volume=97&issue=3&spage=267&pages=267-76&date=2005&title=International+Journal+of+Food+Microbiology&atitle=Enterococcus+species+dominating+in+fresh+modified-atmosphere-packaged%2C+marinated+broiler+legs+are+overgrown+by+Carnobacterium+and+Lactobacillus+species+during+storage+at+6+degrees+C.&aulast=Bjorkroth&pid=<author>Bjorkroth+J%3BRistiniemi+M%3BVandamme+P%3BKorkeala+H<%2Fauthor><AN>15582737<%2FAN><DT>Journal+Article<%2FDT>)

61.

NMR solution structure of ImB2, a protein conferring immunity to antimicrobial activity of the type IIa bacteriocin, carnobacteriocin B2.

Sprules T; Kawulka KE; Vederas JC.

Biochemistry. 43(37):11740-9, 2004 Sep 21.

[Journal Article. Research Support, Non-U.S. Gov't]

UI: 15362858

Bacteriocins produced by lactic acid bacteria are potent antimicrobial compounds which are active against closely related bacteria. Producer strains are protected against the effects of their cognate bacteriocins by immunity proteins that are located on the same genetic locus and are coexpressed with the gene encoding the bacteriocin. Several structures are available for class IIa bacteriocins; however, to date, no structures are available for the corresponding immunity proteins. We report here the NMR solution structure of the 111-amino acid immunity protein for carnobacteriocin B2 (ImB2). ImB2 folds into a globular domain in aqueous solution which contains an antiparallel four-helix bundle. Extensive packing by hydrophobic side chains in adjacent helices forms the core of the protein. The C-terminus, containing a fifth helix and an extended strand, is held against the four-helix bundle by hydrophobic interactions with helices 3 and 4. Most of the charged and polar residues in the protein face the solvent. Helix 3 is well-defined to residue 55, and a stretch of nascent helix followed by an unstructured loop joins it to helix 4. No interaction is observed between ImB2 and either carnobacteriocin B2 (CbnB2) or its precursor. Protection from the action of CbnB2 is only observed when ImB2 is expressed within the cell. The loop between helices 3 and 4, and a hydrophobic pocket which it partially masks, may be important for interaction with membrane receptors responsible for sensitivity to class IIa bacteriocins.

Version ID

1

Record Owner

From MEDLINE, a database of the U.S. National Library of Medicine.

Status

MEDLINE

Authors Full Name

Sprules, Tara; Kawulka, Karen E; Vederas, John C.

Institution

Sprules, Tara. Department of Chemistry, University of Alberta, Edmonton, Alberta, Canada T6G 2G2.

MeSH Heading

Amino Acid Sequence. *Bacterial Proteins/ch [Chemistry]. Bacterial Proteins/ge [Genetics]. *Bacterial Proteins/im [Immunology]. Bacteriocins/ge [Genetics]. *Bacteriocins/im [Immunology]. Humans. Models, Molecular. Molecular Sequence Data. Nuclear Magnetic Resonance, Biomolecular. Protein Structure, Secondary. *Protein Structure, Tertiary. Recombinant Fusion Proteins/ge [Genetics]. Recombinant Fusion Proteins/me [Metabolism]. Sequence Alignment.

Registry Number/Name of Substance

0 (Bacterial Proteins). 0 (Bacteriocins). 0 (Recombinant Fusion Proteins). 155982-38-0 (bacteriocin B2 protein, Carnobacterium piscicola).

Year of Publication

2004

Link to the Ovid Full Text or citation:

[Click here for full text options](https://libaccess.mcmaster.ca/login?url=http://ovidsp.ovid.com/ovidweb.cgi?T=JS&CSC=Y&NEWS=N&PAGE=fulltext&D=med5&AN=15362858)

Link to the External Link Resolver:

[SFX](http://sfx.scholarsportal.info/mcmaster?sid=OVID:medline&id=pmid:15362858&id=doi:10.1021%2Fbi048854%2B&issn=0006-2960&isbn=&volume=43&issue=37&spage=11740&pages=11740-9&date=2004&title=Biochemistry&atitle=NMR+solution+structure+of+ImB2%2C+a+protein+conferring+immunity+to+antimicrobial+activity+of+the+type+IIa+bacteriocin%2C+carnobacteriocin+B2.&aulast=Sprules&pid=<author>Sprules+T%3BKawulka+KE%3BVederas+JC<%2Fauthor><AN>15362858<%2FAN><DT>Journal+Article<%2FDT>)

62.

Enhancement of nisin production by Lactococcus lactis in periodically re-alkalized cultures.

Guerra NP; Castro LP.

Biotechnology & Applied Biochemistry. 38(Pt 2):157-67, 2003 Oct.

[Evaluation Study. Journal Article. Research Support, Non-U.S. Gov't]

UI: 12793859

Synthesis of nisin as well as biomass production by Lactococcus lactis subsp. lactis CECT (Coleccion Espanola de Cultivos Tipo) 539 on both hydrolysed mussel-processing waste and whey medium were followed in three fixed volume fed-batch fermentations, with re-alkalization cycles. The two cultures on mussel-processing waste (MPW) were fed with a 240 g/l concentrated glucose and with a concentrated MPW (about 100 g of glucose/l). The culture on whey was fed with a mixture of concentrated whey (48 g of total sugars/l) and a 400 g/l concentrated lactose. The three cultures were mainly characterized with higher nisin titres [49.7, 109.6 and 124.7 bacteriocin activity units (AU)/ml respectively] compared with the batch process on de Man, Rogosa and Sharpe [(1960) J. Appl. Bacteriol. 23, 130-135] medium (49.6 AU/ml), MPW (9.5 AU/ml) and whey (22.5 AU/ml) [1 AU/ml is the amount of antibacterial compound needed to obtain 50% growth inhibition (LD50) compared with control tubes]. In the three fed-batch cultures a shift from homolactic to mixed-acid fermentation was observed, and other products (acetic acid, butane-2,3-diol or ethanol) in addition to lactic acid were detectable in the medium. However, their contributions to the total antibacterial activity of the post-incubates (the cell-free culture supernatant obtained at the end of the fermentation process) of L. lactis CECT 539 against Carnobacterium piscicola CECT 4020 were very low.

Version ID

1

Record Owner

From MEDLINE, a database of the U.S. National Library of Medicine.

Status

MEDLINE

Authors Full Name

Guerra, Nelson Perez; Castro, Lorenzo Pastrana.

Institution

Guerra, Nelson Perez. Departamento de Bioquimica, Xenetica e Inmunoloxia, Facultade de Ciencias de Ourense, Universidade de Vigo, As Lagoas, 32004 Ourense, Spain.

MeSH Heading

Animals. Bacteriocins/pd [Pharmacology]. Biotechnology/mt [Methods]. Bivalvia/me [Metabolism]. Culture Media/ch [Chemistry]. Fermentation. Glucose/me [Metabolism]. Humans. Hydrogen-Ion Concentration. Industrial Waste. Lactococcus lactis/gd [Growth & Development]. *Lactococcus lactis/me [Metabolism]. Male. Milk Proteins. *Nisin/bi [Biosynthesis]. Time Factors. Whey Proteins.

Registry Number/Name of Substance

0 (Bacteriocins). 0 (Culture Media). 0 (Industrial Waste). 0 (Milk Proteins). 0 (Whey Proteins). 1414-45-5 (Nisin). IY9XDZ35W2 (Glucose).

Year of Publication

2003

Link to the Ovid Full Text or citation:

[Click here for full text options](https://libaccess.mcmaster.ca/login?url=http://ovidsp.ovid.com/ovidweb.cgi?T=JS&CSC=Y&NEWS=N&PAGE=fulltext&D=med5&AN=12793859)

Link to the External Link Resolver:

[SFX](http://sfx.scholarsportal.info/mcmaster?sid=OVID:medline&id=pmid:12793859&id=doi:10.1042%2FBA20030059&issn=0885-4513&isbn=&volume=38&issue=2&spage=157&pages=157-67&date=2003&title=Biotechnology+%26+Applied+Biochemistry&atitle=Enhancement+of+nisin+production+by+Lactococcus+lactis+in+periodically+re-alkalized+cultures.&aulast=Guerra&pid=<author>Guerra+NP%3BCastro+LP<%2Fauthor><AN>12793859<%2FAN><DT>Evaluation+Study<%2FDT>)

63.

Effects of a bacteriocin-like inhibitory substance from Carnobacterium piscicola against human and salmon isolates of Listeria monocytogenes.

Schobitz R; Suazo V; Costa M; Ciampi L.

International Journal of Food Microbiology. 84(2):237-44, 2003 Jul 25.

[Journal Article. Research Support, Non-U.S. Gov't]

UI: 12781946

The aim of this study was to characterize the antagonism of a bacteriocin-like inhibitory substance (BLIS) produced by Carnobacterium piscicola L103 against Listeria monocytogenes strains isolated from salmon and human samples. The inhibitory effect of the BLIS was evaluated in Tryptic soy agar (TSA) during different growth phases of L. monocytogenes at 5 degrees C, using the well diffusion method. Also, the type of inhibition, either bacteriostatic or bactericidal of the BLIS in Tryptic soy broth (TSB), was studied and the development of resistant cells investigated. Results showed an antagonistic effect of the BLIS on all the strains of L. monocytogenes. Four selected strains presented a higher sensitivity to the BLIS in the exponential growth phase and were more resistant in the stationary phase. In TSB, the inhibitory substance showed a partially bactericidal effect on L. monocytogenes. After inactivation of the BLIS with a protease, however, a regrowth of L. monocytogenes was found. The isolate most affected by the action of the BLIS was one of salmon origin. From the 86 isolated colonies that grew in the presence of the BLIS, 93% showed total resistance and 7% partial resistance, which was maintained through five consecutive culture cycles in the absence of the BLIS.

Version ID

1

Record Owner

From MEDLINE, a database of the U.S. National Library of Medicine.

Status

MEDLINE

Authors Full Name

Schobitz, R; Suazo, V; Costa, M; Ciampi, L.

Institution

Schobitz, R. Facultad de Ciencias Agrarias, Instituto de Ciencia y Tecnologia de los Alimentos, Universidad Austral de Chile, Chile. rschobit@uach.cl

MeSH Heading

Animals. Bacteriocins/bi [Biosynthesis]. *Bacteriocins/pd [Pharmacology]. Consumer Product Safety. Food Microbiology. Humans. Lactobacillaceae/gd [Growth & Development]. *Lactobacillaceae/ph [Physiology]. Listeria monocytogenes/de [Drug Effects]. *Listeria monocytogenes/gd [Growth & Development]. *Salmon/mi [Microbiology].

Registry Number/Name of Substance

0 (Bacteriocins).

Year of Publication

2003

Link to the Ovid Full Text or citation:

[Click here for full text options](https://libaccess.mcmaster.ca/login?url=http://ovidsp.ovid.com/ovidweb.cgi?T=JS&CSC=Y&NEWS=N&PAGE=fulltext&D=med5&AN=12781946)

Link to the External Link Resolver:

[SFX](http://sfx.scholarsportal.info/mcmaster?sid=OVID:medline&id=pmid:12781946&id=doi:10.1016%2Fs0168-1605(02)00406-3&issn=0168-1605&isbn=&volume=84&issue=2&spage=237&pages=237-44&date=2003&title=International+Journal+of+Food+Microbiology&atitle=Effects+of+a+bacteriocin-like+inhibitory+substance+from+Carnobacterium+piscicola+against+human+and+salmon+isolates+of+Listeria+monocytogenes.&aulast=Schobitz&pid=<author>Schobitz+R%3BSuazo+V%3BCosta+M%3BCiampi+L<%2Fauthor><AN>12781946<%2FAN><DT>Journal+Article<%2FDT>)

64.

The lactic acid bacteria: a literature survey. [Review] [296 refs]

Carr FJ; Chill D; Maida N.

Critical Reviews in Microbiology. 28(4):281-370, 2002.

[Journal Article. Review]

UI: 12546196

The purpose of this review article on the lactic acid bacteria grew from an early curiosity and a desire to convey and impart the broad scope of literary information on their functions as starter cultures, in the manufacture of fermentation products such as dairy products and alcoholic beverages, as well as their contribution to better health. This review article is an attempt to empower the reader and to circumvent the difficult task in acquiring and elucidating a large body of information. The intent is to familiarize the reader with the various lactic species, their habitat or source, associated food, physiological characteristics, colonial morphology, biochemical characteristics, culture media (enrichment, nonselective, and selective), classic description, and taxonomy. This review provides information on Lactobacillus, Lactococcus, Leuconostoc, Pediococcus, Carnobacterium, and Enterococcus. Trends are presented, such as the use of nisin to extend food shelf-life and the current research premise that Probiotic strains may alter the intestinal flora and thus prevent intestinal wall penetration by pathogens. [References: 296]

Version ID

1

Record Owner

From MEDLINE, a database of the U.S. National Library of Medicine.

Status

MEDLINE

Authors Full Name

Carr, Frank J; Chill, Don; Maida, Nino.

Institution

Carr, Frank J. Clinical Laboratory Consulting, 107 Ootsima Way, Loudon, TN 37774, USA.

MeSH Heading

Culture Media. *Food Microbiology. Humans. Lactobacillus/cl [Classification]. Lactobacillus/ph [Physiology]. *Lactobacillus. *Probiotics. Streptococcaceae/cl [Classification]. Streptococcaceae/ph [Physiology]. *Streptococcaceae.

Registry Number/Name of Substance

0 (Culture Media).

Year of Publication

2002

Link to the Ovid Full Text or citation:

[Click here for full text options](https://libaccess.mcmaster.ca/login?url=http://ovidsp.ovid.com/ovidweb.cgi?T=JS&CSC=Y&NEWS=N&PAGE=fulltext&D=med4&AN=12546196)

Link to the External Link Resolver:

[SFX](http://sfx.scholarsportal.info/mcmaster?sid=OVID:medline&id=pmid:12546196&id=doi:10.1080%2F1040-840291046759&issn=1040-841X&isbn=&volume=28&issue=4&spage=281&pages=281-370&date=2002&title=Critical+Reviews+in+Microbiology&atitle=The+lactic+acid+bacteria%3A+a+literature+survey.&aulast=Carr&pid=<author>Carr+FJ%3BChill+D%3BMaida+N<%2Fauthor><AN>12546196<%2FAN><DT>Journal+Article<%2FDT>)

65.

Isolation of Carnobacterium piscicola from human pus--case report.

Chmelar D; Matusek A; Korger J; Durnova E; Steffen M; Chmelarova E.

Folia Microbiologica. 47(4):455-7, 2002.

[Case Reports. Journal Article]

UI: 12422528

Carnobacterium piscicola was first described in 1984. These bacteria are often isolated from fish afflicted with bacterial infections. To date, there has been no reported isolation of this bacterium from human specimens. We report here the isolation of C. piscicola from the pus following traumatic amputation of the right hand in the wrist of a 35-year-old man. The traumatic amputation occurred with an industrial water sawmill. The identity of the human strain was determined biochemically, by 16S rDNA sequence similarity and by fatty-acid methyl-ester profile from bacterial cell.

Version ID

1

Record Owner

From MEDLINE, a database of the U.S. National Library of Medicine.

Status

MEDLINE

Authors Full Name

Chmelar, D; Matusek, A; Korger, J; Durnova, E; Steffen, M; Chmelarova, E.

Institution

Chmelar, D. Department of Bacteriology, Regional Institute of Hygiene, Ostrava, Czechia. chmelar@ha-vel.cz

MeSH Heading

Adult. Amputation, Traumatic/mi [Microbiology]. Bacterial Infections/mi [Microbiology]. DNA, Bacterial/ge [Genetics]. Drug Resistance, Bacterial. Fatty Acids/an [Analysis]. Humans. Lactobacillaceae/ch [Chemistry]. Lactobacillaceae/de [Drug Effects]. Lactobacillaceae/ge [Genetics]. *Lactobacillaceae/ip [Isolation & Purification]. Male. *Suppuration/mi [Microbiology]. Wrist.

Registry Number/Name of Substance

0 (DNA, Bacterial). 0 (Fatty Acids).

Year of Publication

2002

Link to the Ovid Full Text or citation:

[Click here for full text options](https://libaccess.mcmaster.ca/login?url=http://ovidsp.ovid.com/ovidweb.cgi?T=JS&CSC=Y&NEWS=N&PAGE=fulltext&D=med4&AN=12422528)

Link to the External Link Resolver:

[SFX](http://sfx.scholarsportal.info/mcmaster?sid=OVID:medline&id=pmid:12422528&id=doi:10.1007%2Fbf02818708&issn=0015-5632&isbn=&volume=47&issue=4&spage=455&pages=455-7&date=2002&title=Folia+Microbiologica&atitle=Isolation+of+Carnobacterium+piscicola+from+human+pus--case+report.&aulast=Chmelar&pid=<author>Chmelar+D%3BMatusek+A%3BKorger+J%3BDurnova+E%3BSteffen+M%3BChmelarova+E<%2Fauthor><AN>12422528<%2FAN><DT>Case+Reports<%2FDT>)

66.

[A Carnobacterium-like organism isolated form a patient with multiple bacterial synergistic gangrene]. [Chinese]

Xu J; Yang H; Wu J; Lai X; Liu B.

Wei Sheng Wu Hsueh Pao - Acta Microbiologica Sinica. 40(1):21-5, 2000 Feb.

[English Abstract. Journal Article. Research Support, Non-U.S. Gov't]

UI: 12548873

An atypical lactic-acid producing gram positive rod Y6 strain was studied in this report, which was isolated form clinic sample of a patient with multiple bacterial synergistic gangrene, and could not be identified by routine method. A 1.5 kb of 16S rDNA of Y6 strain was synthesized and sequenced. Comparative 16S rDNA sequence analyses revealed that stain Y6 is most closely related to the genus of Carnobacterium. The overall similarity value between Y6 strain and Carnobacterium species are 93% to 97%. The signature nucleotides in 16S rDNA primary sequence of strain Y6 and that of genus of Carnobacterium were identical. The biological features of Y6 strain are very similar to that of Carnobacterium, such as lactic acid as main end product of in PYG broth, no gas produced form fermentation of glucose, catalase negative, no motility. Data suggested that Y6 strain is very similar to the genus of Carnobacterium, of which no strain has been isolated form clinical sample so far. Based on the results obtained, we names Y6 strain as Carnobacterium-like organism.

Version ID

1

Record Owner

From MEDLINE, a database of the U.S. National Library of Medicine.

Status

MEDLINE

Authors Full Name

Xu, J; Yang, H; Wu, J; Lai, X; Liu, B.

Institution

Xu, J. Laboratory of Molecular Medical Bacteriology, Ministry of Health, Institute of Epidemiology and Microbiology, Chinese Academy for Preventive Medicine, Beijing 102206.

MeSH Heading

Base Sequence. DNA, Bacterial/ge [Genetics]. DNA, Ribosomal. *Gangrene/mi [Microbiology]. *Gram-Positive Rods/cl [Classification]. Gram-Positive Rods/ge [Genetics]. Gram-Positive Rods/ip [Isolation & Purification]. Humans. Molecular Sequence Data. RNA, Bacterial/ge [Genetics].

Registry Number/Name of Substance

0 (DNA, Bacterial). 0 (DNA, Ribosomal). 0 (RNA, Bacterial).

Year of Publication

2000

Link to the Ovid Full Text or citation:

[Click here for full text options](https://libaccess.mcmaster.ca/login?url=http://ovidsp.ovid.com/ovidweb.cgi?T=JS&CSC=Y&NEWS=N&PAGE=fulltext&D=med4&AN=12548873)

Link to the External Link Resolver:

[SFX](http://sfx.scholarsportal.info/mcmaster?sid=OVID:medline&id=pmid:12548873&id=doi:&issn=0001-6209&isbn=&volume=40&issue=1&spage=21&pages=21-5&date=2000&title=Wei+Sheng+Wu+Hsueh+Pao+-+Acta+Microbiologica+Sinica&atitle=%5BA+Carnobacterium-like+organism+isolated+form+a+patient+with+multiple+bacterial+synergistic+gangrene%5D.&aulast=Xu&pid=<author>Xu+J%3BYang+H%3BWu+J%3BLai+X%3BLiu+B<%2Fauthor><AN>12548873<%2FAN><DT>English+Abstract<%2FDT>)
