# Supplementary material for: Carnobacterium inhibens isolated in blood culture of an immunocompromised, metastatic cancer patient: a case report and literature review
Source: BMC Infect Dis. 2021 May 1;21:403. doi: 10.1186/s12879-021-06095-7 (PMC8088058; doi:10.1186/s12879-021-06095-7)
Supplement: Supplementary file 2 — Additional file 2. Database: Embase <1974 to 2020 December 29> – Search Strategy. Compilation of search strategy, search key terms, and full list of journal article titles and abstracts from initial literature search of EMBASE database (inception to December 2020); list was used for screening of relevant articles for subsequent literature review (Table 1). [file 12879_2021_6095_MOESM2_ESM.doc]

Database: Embase <1974 to 2020 December 29>

Search Strategy:

--------------------------------------------------------------------------------

1 exp Carnobacterium/ or carnobacterium.mp. (649)

2 human.mp. or exp human/ (22732023)

3 1 and 2 (98)

***************************

1.

Nested structure of intraspecific competition network in Carnobacterium maltaromaticum.

Ramia N.E., Mangavel C., Gaiani C., Muller-Gueudin A., Taha S., Revol-Junelles A.-M., Borges F.

Scientific reports. 10 (1) (pp 7335), 2020. Date of Publication: 30 Apr 2020.

AN: 631681010

While competition targeting food-borne pathogens is being widely documented, few studies have focused on competition among non-pathogenic food bacteria. Carnobacterium maltaromaticum is a genetically diverse lactic acid bacterium known for comprising several bacteriocinogenic strains with bioprotective potentialities against the food-borne pathogen Listeria monocytogenes. The aim of our study is to examine the network properties of competition among a collection of 73 strains of C. maltaromaticum and to characterize their individual interaction potential. The performed high-throughput competition assays, investigating 5 329 pairwise interactions, showed that intraspecific competition was major in C. maltaromaticum with approximately 56% of the sender strains antagonizing at least one receiver strain. A high diversity of inhibitory and sensitivity spectra was identified along with a majority of narrow inhibitory as well as sensitivity spectra. Through network analysis approach, we determined the highly nested architecture of C. maltaromaticum competition network, thus showing that competition in this species is determined by both the spectrum width of the inhibitory activity of sender strains and the spectrum width of the sensitivity of receiver strains. This study provides knowledge of the competition network in C. maltaromaticum that could be used in rational assembly of compatible microbial strains for the design of mixed starter cultures.

PMID

32355239 [<http://www.ncbi.nlm.nih.gov/pubmed/?term=32355239>]

Author NameID

Mangavel, Cecile; ORCID: <http://orcid.org/0000-0002-4582-8675> Gaiani, Claire; ORCID: <http://orcid.org/0000-0003-0434-8453>

Institution

(Ramia, Mangavel, Gaiani, Revol-Junelles, Borges) LIBio, Universite de Lorraine, Nancy F-54000, France (Ramia, Taha) Laboratoire de Biotechnologies Appliquees, EDST, Universite Libanaise, Tripoli, Lebanon

(Muller-Gueudin) CNRS, Inria, IECL, Universite de Lorraine, Nancy F-54000, France

Publisher

NLM (Medline)

Emtree Heading

animal; *antibiosis; binding competition; Carnobacterium; fish; fish product; *food contamination; *food control; human; Listeria monocytogenes; meat; metabolism; *microbial sensitivity test; microbiology; *physiology; species difference; bacteriocin; lactic acid.

Drug Index Terms

bacteriocin; lactic acid.

Other Index Terms

animal; *antibiosis; binding competition; Carnobacterium; fish; fish product; *food contamination; *food control; human; Listeria monocytogenes; meat; metabolism; *microbial sensitivity test; microbiology; *physiology; species difference.

Link to the Ovid Full Text or citation:

[Click here for full text options](https://libaccess.mcmaster.ca/login?url=http://ovidsp.ovid.com/ovidweb.cgi?T=JS&CSC=Y&NEWS=N&PAGE=fulltext&D=emexc&AN=631681010)

Link to the External Link Resolver:

[SFX](http://sfx.scholarsportal.info/mcmaster?sid=OVID:embase&id=pmid:32355239&id=doi:10.1038%2Fs41598-020-63844-5&issn=2045-2322&isbn=&volume=10&issue=1&spage=7335&pages=7335&date=2020&title=Scientific+reports&atitle=Nested+structure+of+intraspecific+competition+network+in+Carnobacterium+maltaromaticum&aulast=Ramia&pid=<author>Ramia+N.E.%3BMangavel+C.%3BGaiani+C.%3BMuller-Gueudin+A.%3BTaha+S.%3BRevol-Junelles+A.-M.%3BBorges+F.<%2Fauthor><AN>631681010<%2FAN><DT>Article<%2FDT>)

2.

In the age of synthetic biology, will antimicrobial peptides be the next generation of antibiotics?.

Jaumaux F., de Cadinanos L.P.G., Gabant P.

Antibiotics. 9 (8) (pp 1-13), 2020. Article Number: 484. Date of Publication: August 2020.

AN: 2004877538

Antibiotics have changed human health and revolutionised medical practice since the Second World War. Today, the use of antibiotics is increasingly limited by the rise of antimicrobial-resistant strains. Additionally, broad-spectrum antibiotic activity is not adapted to maintaining a balanced microbiome essential for human health. Targeted antimicrobials could overcome these two drawbacks. Although the rational design of targeted antimicrobial molecules presents a formidable challenge, in nature, targeted genetically encoded killing molecules are used by microbes in their natural ecosystems. The use of a synthetic biology approach allows the harnessing of these natural functions. In this commentary article we illustrate the potential of applying synthetic biology towards bacteriocins to design a new generation of antimicrobials.

Copyright © 2020 by the authors. Licensee MDPI, Basel, Switzerland.

Institution

(Jaumaux, de Cadinanos, Gabant) Syngulon, Rue du Bois Saint-Jean 15/1, Seraing 4102, Belgium

Publisher

MDPI AG

Emtree Heading

antibiotic resistance; antimicrobial activity; article; bacterial infection; bacteriophage; Carnobacterium maltaromaticum; Clostridioides difficile; CRISPR Cas system; diagnostic test; disease predisposition; drug design; dysbiosis; fecal microbiota transplantation; genetic engineering; host pathogen interaction; human; intestine flora; lactic acid bacterium; Lactococcus lactis; microbial community; microbiome; phage therapy; priority journal; Staphylococcus aureus; Staphylococcus epidermidis; *synthetic biology; transgenic organism; *antibiotic agent; bacteriocin; meticillin; nisin; penicillin derivative; *polypeptide antibiotic agent; probiotic agent; unclassified drug; vancomycin; carnocyclin A.

Candidate Terms

carnocyclin A [drug term].

Drug Index Terms

*antibiotic agent; bacteriocin; meticillin; nisin; penicillin derivative; *polypeptide antibiotic agent; probiotic agent; unclassified drug; vancomycin.

Other Index Terms

antibiotic resistance; antimicrobial activity; Article; bacterial infection; bacteriophage; Carnobacterium maltaromaticum; Clostridioides difficile; CRISPR Cas system; diagnostic test; disease predisposition; drug design; dysbiosis; fecal microbiota transplantation; genetic engineering; host pathogen interaction; human; intestine flora; lactic acid bacterium; Lactococcus lactis; microbial community; microbiome; phage therapy; priority journal; Staphylococcus aureus; Staphylococcus epidermidis; *synthetic biology; transgenic organism.

Link to the Ovid Full Text or citation:

[Click here for full text options](https://libaccess.mcmaster.ca/login?url=http://ovidsp.ovid.com/ovidweb.cgi?T=JS&CSC=Y&NEWS=N&PAGE=fulltext&D=emexc&AN=2004877538)

Link to the External Link Resolver:

[SFX](http://sfx.scholarsportal.info/mcmaster?sid=OVID:embase&id=pmid:&id=doi:10.3390%2Fantibiotics9080484&issn=2079-6382&isbn=&volume=9&issue=8&spage=1&pages=1-13&date=2020&title=Antibiotics&atitle=In+the+age+of+synthetic+biology%2C+will+antimicrobial+peptides+be+the+next+generation+of+antibiotics%3F&aulast=Jaumaux&pid=<author>Jaumaux+F.%3Bde+Cadinanos+L.P.G.%3BGabant+P.<%2Fauthor><AN>2004877538<%2FAN><DT>Article<%2FDT>)

3.

Antimicrobial activity of tissue and associated bacteria from benthic sea anemone Stichodactyla haddoni against microbial pathogens.

Prakash Williams G., Babu S., Ravikumar S., Kathiresan K., Arul Prathap S., Chinnapparaj S., Marian M.P., Liakath Alikhan S.

Journal of Environmental Biology. 28 (4) (pp 789-793), 2007. Date of Publication: October 2007.

AN: 47605734

Associated bacteria from Stichodactyla haddoni are found maximum in tentacle tissues than the body tissue. There are eight associated bacterial species viz., Alcaligenes sp, Corynebacterium sp, Aeromonas sp, Sporosarcina sp, Renibacterium sp, Carnobacterium sp1, Carnobacterium sp2 and Salinococcus sp were recorded. The culture extracts from the associated bacterial species showed sensitivity against human bacterial and fungal pathogens. However, the hexane tissue extract of sea anemone showed maximum sensitivity (24 mm dia.) against the fish bacterial pathogen Aeromonas hydrophila than the other chosen pathogens. Comparatively, the tissue extracts showed promising antimicrobial sensitivity than the cell free extracts of associated bacteria, and hence, the tissue samples from the sea anemone Stichodactyla haddoni is recommended for further exploration of novel antimicrobial drugs than the associated bacteria. © Triveni Enterprises.

PMID

18405113 [<http://www.ncbi.nlm.nih.gov/pubmed/?term=18405113>]

Institution

(Prakash Williams) Department of Microbial Technology, Malankara Catholic College, Mariagiri, Kaliakkavilai-629 153, India (Ravikumar) Department of Oceanography and Coastal Area Studies, Alagappa University, Thondi - 623 409, India

(Babu, Arul Prathap, Chinnapparaj, Marian) Division of Marine Microbiology and Medicine, Centre for Marine Science and Technology, Manonmaniam Sundaranar University, Rajakkamangalam-629 502, India

(Kathiresan) CAS in Marine Biology, Annamalai University, Portonovo-608 502, India

(Liakath Alikhan) Department of Chemistry, Dr. Zakir Hussain College, Ilayangudi-623 702, India

Publisher

Triveni Enterprises

Emtree Heading

Aeromonas; Aeromonas hydrophila; antibiotic sensitivity; antimicrobial activity; article; bacterial strain; *bacterium; bacterium culture; *benthos; Carnobacterium; comparative study; Corynebacterium; fish; fungus; human; microorganism; nonhuman; pathogenesis; Renibacterium; sample; *sea anemone; species; Sporosarcina; antiinfective agent; cell extract; hexane; tissue extract; alcaligene; *Stichodactyla haddoni.

Candidate Terms

alcaligene [other term]; *stichodactyla haddoni [other term].

Drug Index Terms

antiinfective agent; cell extract; hexane; tissue extract.

Other Index Terms

Aeromonas; Aeromonas hydrophila; antibiotic sensitivity; antimicrobial activity; article; bacterial strain; *bacterium; bacterium culture; *benthos; Carnobacterium; comparative study; Corynebacterium; fish; fungus; human; microorganism; nonhuman; pathogenesis; Renibacterium; sample; *sea anemone; species; Sporosarcina.

Link to the Ovid Full Text or citation:

[Click here for full text options](https://libaccess.mcmaster.ca/login?url=http://ovidsp.ovid.com/ovidweb.cgi?T=JS&CSC=Y&NEWS=N&PAGE=fulltext&D=emexc&AN=47605734)

Link to the External Link Resolver:

[SFX](http://sfx.scholarsportal.info/mcmaster?sid=OVID:embase&id=pmid:18405113&id=doi:&issn=0254-8704&isbn=&volume=28&issue=4&spage=789&pages=789-793&date=2007&title=Journal+of+Environmental+Biology&atitle=Antimicrobial+activity+of+tissue+and+associated+bacteria+from+benthic+sea+anemone+Stichodactyla+haddoni+against+microbial+pathogens&aulast=Prakash+Williams&pid=<author>Prakash+Williams+G.%3BBabu+S.%3BRavikumar+S.%3BKathiresan+K.%3BArul+Prathap+S.%3BChinnapparaj+S.%3BMarian+M.P.%3BLiakath+Alikhan+S.<%2Fauthor><AN>47605734<%2FAN><DT>Article<%2FDT>)

4.

Review - Lactic acid bacteria in traditional fermented Asian foods.

Azam M., Mohsin M., Ijaz H., Tulain U.R., Ashraf M.A., Fayyaz A., Abadeen Z., Kamran Q.

Pakistan journal of pharmaceutical sciences. 30 (5) (pp 1803-1814), 2017. Date of Publication: 01 Sep 2017.

AN: 627330443

Lactic acid bacteria play vital roles in various fermented foods in Asia. This paper reviews many types of the world's lactic acid fermented foods and discusses the beneficial effects of lactic acid fermentation of food. The lactic acid bacteria associated with foods now include species of the genera Carnobacterium, Enterococcus, Lactobacillus, Lactococcus, Leuconostoc, Oenococcus, Pediococcus, Streptococcus, Tetragenococcus, Vagococcus and Weissella. Lactic acid bacteria (LAB) are involved in many fermentation processes of Asian traditional foods, demonstrating their profound effects on improving food quality and food safety. During the past few decades' interest has arisen in the use of the varied antagonistic activities of LAB to extent the shelf-life of protein-rich products such as meats and fish. This review article outlines the main types of LAB fermentation as well as their typical fermented foods such as idli, kishk, sauerkraut, koumiss, Suan-tsai, stinky tofu, Chinese sausage and kefir. The roles of LAB and the reasons for their common presence are also discussed.

Institution

(Azam, Mohsin) Institute of Microbiology, University of Agriculture, Faisalabad, Pakistan (Ijaz, Tulain, Ashraf) Faculty of Pharmacy, University of Sargodha, Punjab, Pakistan

(Fayyaz, Abadeen) Department of Pathology, University of Agriculture, Faisalabad, Pakistan

(Kamran) Institute of Pharmacy, Physiology and Pharmacology, University of Agriculture, Faisalabad, Pakistan

Publisher

NLM (Medline)

Emtree Heading

Asia; *Asian continental ancestry group; diet; *ethnology; *fermentation; fermented product; food control; food preservation; food quality; food safety; human; Lactobacillales; *metabolism; *microbiology; nutritional value; *procedures; product safety; lactic acid.

Drug Index Terms

lactic acid.

Other Index Terms

Asia; *Asian continental ancestry group; diet; *ethnology; *fermentation; fermented product; food control; food preservation; food quality; food safety; human; Lactobacillales; *metabolism; *microbiology; nutritional value; *procedures; product safety.

Link to the Ovid Full Text or citation:

[Click here for full text options](https://libaccess.mcmaster.ca/login?url=http://ovidsp.ovid.com/ovidweb.cgi?T=JS&CSC=Y&NEWS=N&PAGE=fulltext&D=emexc&AN=627330443)

Link to the External Link Resolver:

[SFX](http://sfx.scholarsportal.info/mcmaster?sid=OVID:embase&id=pmid:&id=doi:&issn=1011-601X&isbn=&volume=30&issue=5&spage=1803&pages=1803-1814&date=2017&title=Pakistan+journal+of+pharmaceutical+sciences&atitle=Review+-+Lactic+acid+bacteria+in+traditional+fermented+Asian+foods&aulast=Azam&pid=<author>Azam+M.%3BMohsin+M.%3BIjaz+H.%3BTulain+U.R.%3BAshraf+M.A.%3BFayyaz+A.%3BAbadeen+Z.%3BKamran+Q.<%2Fauthor><AN>627330443<%2FAN><DT>Review<%2FDT>)

5.

Effective survival of immobilized Lactobacillus casei during ripening and heat treatment of probiotic dry-fermented sausages and investigation of the microbial dynamics.

Sidira M., Karapetsas A., Galanis A., Kanellaki M., Kourkoutas Y.

Meat science. 96 (2 Pt A) (pp 948-955), 2014. Date of Publication: Feb 2014.

AN: 563078390

The aim was the assessment of immobilized Lactobacillus casei ATCC 393 on wheat in the production of probiotic dry-fermented sausages and the investigation of the microbial dynamics. For comparison, sausages containing either free L. casei ATCC 393 or no starter culture were also prepared. During ripening, the numbers of lactobacilli exceeded 7 log cfu/g, while a drastic decrease was observed in enterobacteria, staphylococci and pseudomonas counts. Microbial diversity was further studied applying a PCR-DGGE protocol. Members of Lactobacillus, Leuconostoc, Lactococcus, Carnobacterium, Brochothrix, Bacillus and Debaryomyces were the main microbial populations detected. Microbiological and strain-specific multiplex PCR analysis confirmed that the levels of L. casei ATCC 393 in the samples after 66 days of ripening were above the minimum concentration for conferring a probiotic effect (>= 6 log cfu/g). However, after heat treatment, this strain was detected at the above levels, only in sausages containing immobilized cells. © 2013.

Institution

(Sidira) Food Biotechnology Group, Section of Analytical Environmental and Applied Chemistry, Department of Chemistry, University of Patras, GR-26500 Patras, Greece.; Applied Microbiology and Molecular Biotechnology Research Group, Department of Molecular Biology & Genetics, Democritus University of Thrace, Alexandroupolis 68100, Greece.

Emtree Heading

article; bacterial count; Enterobacteriaceae; *fermentation; food contamination/pc [Prevention]; food control; *food handling; heat; human; immobilized cell; isolation and purification; *Lactobacillus; *meat/an [Drug Analysis]; metabolism; methodology; microbiology; multiplex polymerase chain reaction; physical chemistry; polymerase chain reaction; Pseudomonas; Staphylococcus; taste; *probiotic agent; Dry-fermented sausages; L. casei ATCC 393; PCR-DGGE.

Candidate Terms

Dry-fermented sausages [other term]; L. casei ATCC 393 [other term]; PCR-DGGE [other term].

Drug Index Terms

*probiotic agent.

Other Index Terms

article; bacterial count; Enterobacteriaceae; *fermentation; food contamination / prevention; food control; *food handling; heat; human; immobilized cell; isolation and purification; *Lactobacillus; *meat / *drug analysis; metabolism; methodology; microbiology; multiplex polymerase chain reaction; physical chemistry; polymerase chain reaction; Pseudomonas; Staphylococcus; taste.

Link to the Ovid Full Text or citation:

[Click here for full text options](https://libaccess.mcmaster.ca/login?url=http://ovidsp.ovid.com/ovidweb.cgi?T=JS&CSC=Y&NEWS=N&PAGE=fulltext&D=emexc&AN=563078390)

Link to the External Link Resolver:

[SFX](http://sfx.scholarsportal.info/mcmaster?sid=OVID:embase&id=pmid:&id=doi:&issn=1873-4138&isbn=&volume=96&issue=2+Pt+A&spage=948&pages=948-955&date=2014&title=Meat+science&atitle=Effective+survival+of+immobilized+Lactobacillus+casei+during+ripening+and+heat+treatment+of+probiotic+dry-fermented+sausages+and+investigation+of+the+microbial+dynamics&aulast=Sidira&pid=<author>Sidira+M.%3BKarapetsas+A.%3BGalanis+A.%3BKanellaki+M.%3BKourkoutas+Y.<%2Fauthor><AN>563078390<%2FAN><DT>Article<%2FDT>)

6.

Antibiotic Resistance Genes and Bacterial Communities of Farmed Rainbow Trout Fillets (Oncorhynchus mykiss).

Helsens N., Calvez S., Prevost H., Bouju-Albert A., Maillet A., Rossero A., Hurtaud-Pessel D., Zagorec M., Magras C.

Frontiers in Microbiology. 11 (no pagination), 2020. Article Number: 590902. Date of Publication: 03 Dec 2020.

AN: 633680367

The rise of antibiotic resistance is not only a challenge for human and animal health treatments, but is also posing the risk of spreading among bacterial populations in foodstuffs. Farmed fish-related foodstuffs, the food of animal origin most consumed worldwide, are suspected to be a reservoir of antibiotic resistance genes and resistant bacterial hazards. However, scant research has been devoted to the possible sources of diversity in fresh fillet bacterial ecosystems (farm environment including rivers and practices, and factory environment). In this study bacterial communities and the antibiotic resistance genes of fresh rainbow trout fillet were described using amplicon sequencing of the V3-V4 region of the 16S rRNA gene and high-throughput qPCR assay. The antibiotic residues were quantified using liquid chromatography/mass spectrometry methods. A total of 56 fillets (composed of muscle and skin tissue) from fish raised on two farms on the same river were collected and processed under either factory or laboratory sterile filleting conditions. We observed a core-bacterial community profile on the fresh rainbow trout fillets, but the processing conditions of the fillets has a great influence on their mean bacterial load (3.38 +/- 1.01 log CFU/g vs 2.29 +/- 0.72 log CFU/g) and on the inter-individual diversity of the bacterial community. The bacterial communities were dominated by Gamma- and Alpha-proteobacteria, Bacteroidetes, Firmicutes, and Actinobacteria. The most prevalent genera were Pseudomonas, Escherichia-Shigella, Chryseobacterium, and Carnobacterium. Of the 73 antibiotic residues searched, only oxytetracycline residues were detected in 13/56 fillets, all below the European Union maximum residue limit (6.40-40.20 mug/kg). Of the 248 antibiotic resistance genes searched, 11 were found to be present in at least 20% of the fish population (tetracycline resistance genes tetM and tetV, beta-lactam resistance genes blaDHA and blaACC, macrolide resistance gene mphA, vancomycin resistance genes vanTG and vanWG and multidrug-resistance genes mdtE, mexF, vgaB and msrA) at relatively low abundances calculated proportionally to the 16S rRNA gene.

© Copyright © 2020 Helsens, Calvez, Prevost, Bouju-Albert, Maillet, Rossero, Hurtaud-Pessel, Zagorec and Magras.

Institution

(Helsens, Prevost, Bouju-Albert, Maillet, Rossero, Zagorec, Magras) INRAE, Oniris, SECALIM, Nantes, France (Helsens, Calvez) INRAE, Oniris, BIOEPAR, Nantes, France

(Hurtaud-Pessel) ANSES, Laboratoire de Fougeres, Unite Analyse des Residus et Contaminants, Fougeres, France

Publisher

Frontiers Media S.A.

Emtree Heading

Actinobacteria; agricultural land; Alphaproteobacteria; amplicon; article; bacterial load; *beta-lactam resistance; Carnobacterium; Chryseobacterium; controlled study; Escherichia; European Union; *fillet (fish); human; liquid chromatography-mass spectrometry; macrolide resistance; *microbial community; multidrug resistance; muscle tissue; nonhuman; *Oncorhynchus mykiss; Pseudomonas; quantitative analysis; river; Shigella; skin; tetracycline resistance; vancomycin resistance; endogenous compound; *oxytetracycline; RNA 16S.

Drug Index Terms

endogenous compound [m]; *oxytetracycline [m]; RNA 16S [m].

Other Index Terms

Actinobacteria [m]; agricultural land [m]; Alphaproteobacteria [m]; amplicon [m]; article [m]; bacterial load [m]; *beta-lactam resistance [m]; Carnobacterium [m]; Chryseobacterium [m]; controlled study [m]; Escherichia [m]; European Union [m]; *fillet (fish) [m]; human [m]; liquid chromatography-mass spectrometry [m]; macrolide resistance [m]; *microbial community [m]; multidrug resistance [m]; muscle tissue [m]; nonhuman [m]; *Oncorhynchus mykiss [m]; Pseudomonas [m]; quantitative analysis [m]; river [m]; Shigella [m]; skin [m]; tetracycline resistance [m]; vancomycin resistance [m].

Link to the Ovid Full Text or citation:

[Click here for full text options](https://libaccess.mcmaster.ca/login?url=http://ovidsp.ovid.com/ovidweb.cgi?T=JS&CSC=Y&NEWS=N&PAGE=fulltext&D=emexc&AN=633680367)

Link to the External Link Resolver:

[SFX](http://sfx.scholarsportal.info/mcmaster?sid=OVID:embase&id=pmid:&id=doi:10.3389%2Ffmicb.2020.590902&issn=1664-302X&isbn=&volume=11&issue=&spage=&pages=&date=2020&title=Frontiers+in+Microbiology&atitle=Antibiotic+Resistance+Genes+and+Bacterial+Communities+of+Farmed+Rainbow+Trout+Fillets+(Oncorhynchus+mykiss)&aulast=Helsens&pid=<author>Helsens+N.%3BCalvez+S.%3BPrevost+H.%3BBouju-Albert+A.%3BMaillet+A.%3BRossero+A.%3BHurtaud-Pessel+D.%3BZagorec+M.%3BMagras+C.<%2Fauthor><AN>633680367<%2FAN><DT>Article<%2FDT>)

7.

Divergicin M35-Chitosan Film: Development and Characterization.

Benabbou R., Subirade M., Desbiens M., Fliss I.

Probiotics and Antimicrobial Proteins. 12 (4) (pp 1562-1570), 2020. Date of Publication: 01 Dec 2020.

AN: 2004992011

Chitosan films loaded with bacteriocin were examined by FTIR spectroscopy, tested for color, puncture strength, water vapor permeability, and as antimicrobials of Listeria innocua HPB13. Divergicin M35, a bacteriocin produced by Carnobacterium divergens, was incorporated into films made with chitosan of molecular mass 2 kDa, 20 kDa, or 100 kDa and de-acetylated either 87% or 95%. Only 100 kDa chitosan yielded films that could be peeled and handled easily. The higher degree of de-acetylation increased the total color factor (DELTAE) of bacteriocin-loaded films, their permeability, and puncture strength. Incorporation of divergicin M35 into the films increased amide I peak intensity but otherwise did not induce significant structural change. The FTIR spectra of divergicin M35 shed from the films did not differ from those of the original free bacteriocin, except in overall peak intensity. The release of active divergicin M35 from the film was faster into the buffer than into tryptic soy broth and peaked at 10-12 h in both cases. Chitosan 95% de-acetylated and loaded with divergicin M35 was the most active, producing a six-log drop in Listeria innocua HPB13 viable count within 24 h. These results suggest that the biocompatible and biodegradable films developed here have the potential for application as antimicrobials of Listeria spp. in foods, especially ready-to-eat, minimally processed products.

Copyright © 2020, Springer Science+Business Media, LLC, part of Springer Nature.

PMID

32430585 [<http://www.ncbi.nlm.nih.gov/pubmed/?term=32430585>]

Institution

(Benabbou, Subirade, Fliss) Department of Food Science, Faculty of Agriculture and Food Sciences, Institute of Nutrition and Functional Foods, Laval University, Quebec City, QC, Canada (Benabbou) Laboratory Engineering Research-OSIL Team Optimization of Industrial and Logistics Systems, University Hassan II, Casablanca, Morocco

(Desbiens) Centre Technologique des Produits aquatiques, Ministere de l'Agriculture des Pecheries et de l'Alimentation, Gaspe, QC, Canada

Publisher

Springer

Emtree Heading

article; Carnobacterium; case report; clinical article; convenience food; deacetylation; Fourier transform infrared spectroscopy; human tissue; Listeria innocua; molecular weight; nonhuman; *physical chemistry; puncture; viable cell count; water vapor; amide; bacteriocin; buffer; *chitosan.

Drug Index Terms

amide [m]; bacteriocin [m]; buffer [m]; *chitosan [m].

Other Index Terms

article [m]; Carnobacterium [m]; case report [m]; clinical article [m]; convenience food [m]; deacetylation [m]; Fourier transform infrared spectroscopy [m]; human tissue [m]; Listeria innocua [m]; molecular weight [m]; nonhuman [m]; *physical chemistry [m]; puncture [m]; viable cell count [m]; water vapor [m].

Link to the Ovid Full Text or citation:

[Click here for full text options](https://libaccess.mcmaster.ca/login?url=http://ovidsp.ovid.com/ovidweb.cgi?T=JS&CSC=Y&NEWS=N&PAGE=fulltext&D=emexc&AN=2004992011)

Link to the External Link Resolver:

[SFX](http://sfx.scholarsportal.info/mcmaster?sid=OVID:embase&id=pmid:32430585&id=doi:10.1007%2Fs12602-020-09660-9&issn=1867-1306&isbn=&volume=12&issue=4&spage=1562&pages=1562-1570&date=2020&title=Probiotics+and+Antimicrobial+Proteins&atitle=Divergicin+M35-Chitosan+Film%3A+Development+and+Characterization&aulast=Benabbou&pid=<author>Benabbou+R.%3BSubirade+M.%3BDesbiens+M.%3BFliss+I.<%2Fauthor><AN>2004992011<%2FAN><DT>Article<%2FDT>)

8.

Use of Probiotics in Commercially Important Finfish Aquaculture.

Jamal M.T., Sumon Md.A.A., Pugazhendi A., Harbi M.A., Hussain M.A., Haque M.F.

International Journal of Probiotics and Prebiotics. 15 (1) (pp 7-21), 2020. Date of Publication: 2020.

AN: 2007349773

Finfish aquaculture is one of the quickly developing food industries in the world. But, the major drawbacks of this industry are the frequent outbreak of infectious diseases due to elevated stress in an intensive culture system. Antibiotics are extenAsively used to combat these diseases. Prophylactic administrations of antibiotics in aquaculture lead to the emergence and spread of antibiotic-resistant pathogens which indirectly cause a risk to health of human. Therefore, hard rules and regulations have been established in various countries to minimize or ban the application of antibiotics in finfish aquaculture. Recently probiotics have garnered significant attraction as an alternative measure for disease prevention in aquaculture. Probiotics increase health status, disease resistance, growth performance and feed utilization through improving hosts microbial balance. This review presents the summary and discussion of the results of the effects of probiotic administration in the culture of commercially important finfish. Besides, the current study attempts to explore the gap in present scientific information as well as suggests concerns that worth further research.

Copyright © 2020 New Century Health Publishers. All rights reserved.

Institution

(Jamal, Sumon, Pugazhendi, Harbi) Department of Marine Biology, Faculty of Marine Sciences, King Abdulaziz University, P.O Box 80207, Jeddah, Saudi Arabia (Pugazhendi) Center of Excellence in Environmental Studies, Faculty of Fisheries, King Abdulaziz University, Jeddah 21589, Saudi Arabia

(Pugazhendi) Fisheries Technology and Quality Control, Faculty of Fisheries, Sylhet Agricultural University, Sylhet, Bangladesh

(Hussain) Department of Zoology, Faculty of Biological Sciences, University of Rajshahi, Rajshahi 6205, Bangladesh

(Haque) Department of Zoology, University of Rajshahi, Rajshai 6205, Bangladesh

Publisher

New Century Health Publishers (E-mail: info@nutraceuticalresearch.org)

Emtree Heading

*aquaculture; Bacillus amyloliquefaciens; Bacillus coagulans; Bacillus subtilis; body growth; Brochothrix thermosphacta; Carnobacterium; carp; disease resistance; Enterobacter; Enterobacter cloacae; Enterococcus casseliflavus; Enterococcus faecium; *fish; Flavobacterium; grass carp; immunity; Lactobacillus; Lactobacillus delbrueckii; Lactobacillus plantarum; Lactobacillus rhamnosus; nonhuman; Oncorhynchus mykiss; Pediococcus acidilactici; Pseudomonas aeruginosa; Pseudomonas fluorescens; review; Rhodobacter sphaeroides; Saccharomyces cerevisiae; Salmo salar; survival rate; Tetraselmis; Tilapia; Vibrio alginolyticus; *probiotic agent.

Drug Index Terms

*probiotic agent.

Other Index Terms

*aquaculture; Bacillus amyloliquefaciens; Bacillus coagulans; Bacillus subtilis; body growth; Brochothrix thermosphacta; Carnobacterium; carp; disease resistance; Enterobacter; Enterobacter cloacae; Enterococcus casseliflavus; Enterococcus faecium; *fish; Flavobacterium; grass carp; immunity; Lactobacillus; Lactobacillus delbrueckii; Lactobacillus plantarum; Lactobacillus rhamnosus; nonhuman; Oncorhynchus mykiss; Pediococcus acidilactici; Pseudomonas aeruginosa; Pseudomonas fluorescens; Review; Rhodobacter sphaeroides; Saccharomyces cerevisiae; Salmo salar; survival rate; Tetraselmis; Tilapia; Vibrio alginolyticus.

Link to the Ovid Full Text or citation:

[Click here for full text options](https://libaccess.mcmaster.ca/login?url=http://ovidsp.ovid.com/ovidweb.cgi?T=JS&CSC=Y&NEWS=N&PAGE=fulltext&D=emexb&AN=2007349773)

Link to the External Link Resolver:

[SFX](http://sfx.scholarsportal.info/mcmaster?sid=OVID:embase&id=pmid:&id=doi:10.37290%2FIJPP2641-7197.157-21&issn=1555-1431&isbn=&volume=15&issue=1&spage=7&pages=7-21&date=2020&title=International+Journal+of+Probiotics+and+Prebiotics&atitle=Use+of+Probiotics+in+Commercially+Important+Finfish+Aquaculture&aulast=Jamal&pid=<author>Jamal+M.T.%3BSumon+Md.A.A.%3BPugazhendi+A.%3BHarbi+M.A.%3BHussain+M.A.%3BHaque+M.F.<%2Fauthor><AN>2007349773<%2FAN><DT>Review<%2FDT>)

9.

Shelf-life and microbial community dynamics of super-chilled beef imported from Australia to China.

Chen X., Zhang Y., Yang X., Hopkins D.L., Zhu L., Dong P., Liang R., Luo X.

Food research international (Ottawa, Ont.). 120 (pp 784-792), 2019. Date of Publication: 01 Jun 2019.

AN: 627482303

The aim of this study was to investigate the shelf-life and microbial community dynamics of super-chilled vacuum-packaged beef striploins imported from Australia to China after approximately five weeks of shipping time and an additional 15weeks of storage at -1degreeC+/-0.5degreeC. Data analysis using a mixed model (REML) with time as the fixed effect and portion as a random effect, showed that the only beef quality trait that changed during storage was total volatile basic nitrogen (TVBN; P<.05), which reached the threshold of 15mg/100g between 15 and 20weeks (including 5weeks of transport). The total viable count (TVC) accounted for 78% of the variance in TVBN, when storage time was included in the model. Sensory scores decreased as storage time extended (P<.05), but were still acceptable at 20weeks. After 9weeks, Carnobacterium spp. and Lactobacillus spp. dominated alternately and then Lactobacillus became the most prevalent bacteria. An operational taxominc unit based hierarchical cluster analysis using the unweighted pair-group method with arithmetic means was performed and it was shown that the bacterial communities tended to be consistent as storage time extended. Overall indications are that beef which is safe can be imported into China from Australia and aged for extended periods.

Copyright © 2018 Elsevier Ltd. All rights reserved.

PMID

31000298 [<http://www.ncbi.nlm.nih.gov/pubmed/?term=31000298>]

Institution

(Chen, Zhang, Yang, Zhu, Dong, Liang) Lab of Beef Processing and Quality Control, College of Food Science and Engineering, Shandong Agricultural University, Tai'an, Shandong 271018, China (Hopkins) Lab of Beef Processing and Quality Control, College of Food Science and Engineering, Shandong Agricultural University, Tai'an, Shandong 271018, PR China; NSW Department of Primary Industries, Centre for Red Meat and Sheep Development, PO Box 129, Cowra, NSW 2794, Australia

(Luo) Lab of Beef Processing and Quality Control, College of Food Science and Engineering, Shandong Agricultural University, Tai'an, Shandong 271018, PR China; Jiangsu Synergetic Innovation Center of Meat Production and Processing Quality and Safety Control, Nanjing, Jiangsu 210000, PR China

Publisher

NLM (Medline)

Emtree Heading

Australia; China; color; food control; food preservation; *food quality; food storage; human; meat industry; *microflora; oxidation reduction reaction; pH; *procedures; red meat; lipid.

Drug Index Terms

lipid.

Other Index Terms

Australia; China; color; food control; food preservation; *food quality; food storage; human; meat industry; *microflora; oxidation reduction reaction; pH; *procedures; red meat.

Link to the Ovid Full Text or citation:

[Click here for full text options](https://libaccess.mcmaster.ca/login?url=http://ovidsp.ovid.com/ovidweb.cgi?T=JS&CSC=Y&NEWS=N&PAGE=fulltext&D=emexb&AN=627482303)

Link to the External Link Resolver:

[SFX](http://sfx.scholarsportal.info/mcmaster?sid=OVID:embase&id=pmid:31000298&id=doi:10.1016%2Fj.foodres.2018.11.039&issn=1873-7145&isbn=&volume=120&issue=&spage=784&pages=784-792&date=2019&title=Food+research+international+(Ottawa%2C+Ont.)&atitle=Shelf-life+and+microbial+community+dynamics+of+super-chilled+beef+imported+from+Australia+to+China&aulast=Chen&pid=<author>Chen+X.%3BZhang+Y.%3BYang+X.%3BHopkins+D.L.%3BZhu+L.%3BDong+P.%3BLiang+R.%3BLuo+X.<%2Fauthor><AN>627482303<%2FAN><DT>Article<%2FDT>)

10.

Metagenomics reveals a core macrolide resistome related to microbiota in chronic respiratory disease.

Aogain M.M., Lau K.J.X., Cai Z., Narayana J.K., Purbojati R.W., Drautz-Moses D.I., Gaultier N.E., Jaggi T.K., Tiew P.Y., Ong T.H., Koh M.S., Hou A.L.Y., Abisheganaden J.A., Tsaneva-Atanasova K., Schuster S.C., Chotirmall S.H.

American Journal of Respiratory and Critical Care Medicine. 202 (3) (pp 433-447), 2020. Date of Publication: 01 Aug 2020.

AN: 2007550122

Rationale: Long-term antibiotic use for managing chronic respiratory disease is increasing; however, the role of the airway resistome and its relationship to host microbiomes remains unknown.

Objective(s): To evaluate airway resistomes and relate them to host and environmental microbiomes using ultradeep metagenomic shotgun sequencing.

Method(s): Airway specimens from 85 individuals with and without chronic respiratory disease (severe asthma, chronic obstructive pulmonary disease, and bronchiectasis) were subjected to metagenomic sequencing to an average depth exceeding 20 million reads. Respiratory and device-associated microbiomes were evaluated on the basis of taxonomical classification and functional annotation including the Comprehensive Antibiotic Resistance Database to determine airway resistomes. Co-occurrence networks of gene-microbe association were constructed to determine potential microbial sources of the airway resistome. Paired patient-inhaler metagenomes were compared (n = 31) to assess for the presence of airway-environment overlap in microbiomes and/or resistomes.

Measurements and Main Results: Airway metagenomes exhibit taxonomic and metabolic diversity and distinct antimicrobial resistance patterns. A "core"airway resistome dominated by macrolide but with high prevalence of beta-lactam, fluoroquinolone, and tetracycline resistance genes exists and is independent of disease status or antibiotic exposure. Streptococcus and Actinomyces are key potential microbial reservoirs of macrolide resistance including the ermX, ermF, and msrD genes. Significant patient-inhaler overlap in airway microbiomes and their resistomes is identified where the latter may be a proxy for airway microbiome assessment in chronic respiratory disease.

Conclusion(s): Metagenomic analysis of the airway reveals a core macrolide resistome harbored by the host microbiome.

Copyright © 2020 by the American Thoracic Society.

PMID

32320621 [<http://www.ncbi.nlm.nih.gov/pubmed/?term=32320621>]

Author NameID

Aogain, Micheal Mac; ORCID: <http://orcid.org/0000-0002-1726-7700> Chotirmall, Sanjay H.; ORCID: <http://orcid.org/0000-0003-0417-7607>

Tiew, Pei Yee; ORCID: <http://orcid.org/0000-0002-6346-2072>

Institution

(Aogain, Narayana, Jaggi, Tiew, Chotirmall) Lee Kong Chian School of Medicine, Nanyang Technological University, 11 Mandalay Road, Singapore 308232, Singapore (Lau, Cai, Purbojati, Drautz-Moses, Gaultier, Schuster) Singapore Centre for Environmental Life Sciences Engineering, Nanyang Technological University, Singapore, Singapore

(Tiew, Ong, Koh) Department of Respiratory and Critical Care Medicine, Singapore General Hospital, Singapore, Singapore

(Hou, Abisheganaden) Department of Respiratory and Critical Care Medicine, Tan Tock Seng Hospital, Singapore, Singapore

(Tsaneva-Atanasova) Department of Mathematics, College of Engineering, Mathematics and Physical Sciences, University of Exeter, Exeter, United Kingdom

Publisher

American Thoracic Society (E-mail: malexander@thoracic.org)

Emtree Heading

Actinobacteria; Actinomyces; adult; aged; *antibiotic resistome; article; *asthma; bacterial gene; beta-lactam resistance; *bronchiectasis; Carnobacterium maltaromaticum; chronic obstructive lung disease; *chronic respiratory tract disease/dr [Drug Resistance]; clinical article; disease severity; ecology; female; Firmicutes; fluoroquinolone resistance; Fusobacteria; Haemophilus; human; *macrolide resistance; male; metagenome; *metagenomics; microbial diversity; microflora; nonhuman; Prevotella; Prevotella intermedia; priority journal; Pseudomonas; Pseudomonas aeruginosa; Rothia mucilaginosa; *shotgun sequencing; sputum analysis; Streptococcus; tetracycline resistance; Treponema; *macrolide; inhaler; airway flora.

Candidate Terms

airway flora [other term].

Device Index Terms

inhaler.

Drug Index Terms

*macrolide.

Other Index Terms

Actinobacteria; Actinomyces; adult; aged; *antibiotic resistome; Article; *asthma; bacterial gene; beta-lactam resistance; *bronchiectasis; Carnobacterium maltaromaticum; chronic obstructive lung disease; *chronic respiratory tract disease / *drug resistance; clinical article; disease severity; ecology; female; Firmicutes; fluoroquinolone resistance; Fusobacteria; Haemophilus; human; *macrolide resistance; male; metagenome; *metagenomics; microbial diversity; microflora; nonhuman; Prevotella; Prevotella intermedia; priority journal; Pseudomonas; Pseudomonas aeruginosa; Rothia mucilaginosa; *shotgun sequencing; sputum analysis; Streptococcus; tetracycline resistance; Treponema.

Link to the Ovid Full Text or citation:

[Click here for full text options](https://libaccess.mcmaster.ca/login?url=http://ovidsp.ovid.com/ovidweb.cgi?T=JS&CSC=Y&NEWS=N&PAGE=fulltext&D=emexb&AN=2007550122)

Link to the External Link Resolver:

[SFX](http://sfx.scholarsportal.info/mcmaster?sid=OVID:embase&id=pmid:32320621&id=doi:10.1164%2Frccm.201911-2202OC&issn=1073-449X&isbn=&volume=202&issue=3&spage=433&pages=433-447&date=2020&title=American+Journal+of+Respiratory+and+Critical+Care+Medicine&atitle=Metagenomics+reveals+a+core+macrolide+resistome+related+to+microbiota+in+chronic+respiratory+disease&aulast=Aogain&pid=<author>Aogain+M.M.%3BLau+K.J.X.%3BCai+Z.%3BNarayana+J.K.%3BPurbojati+R.W.%3BDrautz-Moses+D.I.%3BGaultier+N.E.%3BJaggi+T.K.%3BTiew+P.Y.%3BOng+T.H.%3BKoh+M.S.%3BHou+A.L.Y.%3BAbisheganaden+J.A.%3BTsaneva-Atanasova+K.%3BSchuster+S.C.%3BChotirmall+S.H.<%2Fauthor><AN>2007550122<%2FAN><DT>Article<%2FDT>)

11.

Identification of symbiotic bacteria in the midgut of the medically important mosquito, Culiseta longiareolata (Diptera: Culicidae).

Ghahvechi Khaligh F., Vahedi M., Chavshin A.R.

BMC research notes. 13 (1) (pp 378), 2020. Date of Publication: 10 Aug 2020.

AN: 632584180

OBJECTIVE: The potential use of symbiotic bacteria for the control of mosquito-borne diseases has attracted the attention of scientists over the past few years. Culiseta longiareolata is among the medically important mosquitoes that transmit a wide range of vector-borne diseases worldwide. However, no extensive studies have been done on the identification of its symbiotic bacteria. Given the role of this species in the transmission of some important diseases and its widespread presence in different parts of the world, including northwestern parts and the West Azerbaijan Province in Iran, a knowledge about the symbiotic bacteria of this species may provide a valuable tool for the biological control of this mosquito. Accordingly, the present study was conducted to isolate and identify the cultivable isolates bacterial symbionts of Culiseta longiareolata using 16S rRNA fragment analysis.

RESULT(S): The midguts of 42 specimens of Cs. longiareolata were dissected, and the bacteria were cultured on agar plates. After the purification of the bacterial colonies, 16srRNA region amplification and gene sequence analysis were performed, and the sequences were confirmed by biochemical methods. In the present study, 21 isolates belonging to the genera Acinetobacter, Aerococcus, Aeromonas, Bacillus, Carnobacterium, Klebsiella, Morganella, Pseudomonas, Shewanella and Staphylococcus were identified.

PMID

32778137 [<http://www.ncbi.nlm.nih.gov/pubmed/?term=32778137>]

Author NameID

Chavshin, Ali Reza; ORCID: <http://orcid.org/0000-0002-2359-8610>

Institution

(Ghahvechi Khaligh, Vahedi, Chavshin) Department of Medical Entomology and Vector Control, School of Public Health, Urmia University of Medical Sciences, Iran, Islamic Republic of (Chavshin) Social Determinants of Health Research Center, Urmia University of Medical Sciences, Iran, Islamic Republic of

Publisher

NLM (Medline)

Emtree Heading

Acinetobacter; Aerococcus; Aeromonas; article; Azerbaijan; Bacillus; bacterium colony; bacterium culture; biological pest control; Carnobacterium; controlled study; gene amplification; gene sequence; human cell; Iran; Klebsiella; *midgut; Morganella; *mosquito; nonhuman; Pseudomonas; sequence analysis; Shewanella; Staphylococcus; *symbiont; endogenous compound; *RNA 16S.

Drug Index Terms

endogenous compound [m]; *RNA 16S [m].

Other Index Terms

Acinetobacter [m]; Aerococcus [m]; Aeromonas [m]; article [m]; Azerbaijan [m]; Bacillus [m]; bacterium colony [m]; bacterium culture [m]; biological pest control [m]; Carnobacterium [m]; controlled study [m]; gene amplification [m]; gene sequence [m]; human cell [m]; Iran [m]; Klebsiella [m]; *midgut [m]; Morganella [m]; *mosquito [m]; nonhuman [m]; Pseudomonas [m]; sequence analysis [m]; Shewanella [m]; Staphylococcus [m]; *symbiont [m].

Link to the Ovid Full Text or citation:

[Click here for full text options](https://libaccess.mcmaster.ca/login?url=http://ovidsp.ovid.com/ovidweb.cgi?T=JS&CSC=Y&NEWS=N&PAGE=fulltext&D=emexb&AN=632584180)

Link to the External Link Resolver:

[SFX](http://sfx.scholarsportal.info/mcmaster?sid=OVID:embase&id=pmid:32778137&id=doi:10.1186%2Fs13104-020-05220-0&issn=1756-0500&isbn=&volume=13&issue=1&spage=378&pages=378&date=2020&title=BMC+research+notes&atitle=Identification+of+symbiotic+bacteria+in+the+midgut+of+the+medically+important+mosquito%2C+Culiseta+longiareolata+(Diptera%3A+Culicidae)&aulast=Ghahvechi+Khaligh&pid=<author>Ghahvechi+Khaligh+F.%3BVahedi+M.%3BChavshin+A.R.<%2Fauthor><AN>632584180<%2FAN><DT>Article<%2FDT>)

12.

Signatures of mucosal microbiome in oral squamous cell carcinoma identified using a random forest model.

Zhou J., Wang L., Yuan R., Yu X., Chen Z., Yang F., Sun G., Dong Q.

Cancer Management and Research. 12 (pp 5353-5363), 2020. Date of Publication: 2020.

AN: 2004653848

Objective: The aim of this study was to explore the signatures of oral microbiome associated with OSCC using a random forest (RF) model.

Patients and Methods: A total of 24 patients with OSCC were enrolled in the study. The oral microbiome was assessed in cancerous lesions and matched paracancerous tissues from each patient using 16S rRNA gene sequencing. Signatures of mucosal microbiome in OSCC were identified using a RF model.

Result(s): Significant differences were found between OSCC lesions and matched paracancerous tissues with respect to the microbial profile and composition. Linear discriminant analysis effect size analyses (LEfSe) identified 15 bacteria genera associated with cancerous lesions. Fusobacterium, Treponema, Streptococcus, Peptostreptococcus, Carnobacterium, Tannerella, Parvimonas and Filifactor were enriched. A classifier based on RF model identified a microbial signature comprising 12 bacteria, which was capable of distinguishing cancerous lesions and paracancerous tissues (AUC = 0.82). The network of the oral microbiome in cancerous lesions appeared to be simplified and fragmented. Functional analyses of oral microbiome showed altered functions in amino acid metabolism and increased capacity of glucose utilization in OSCC.

Conclusion(s): The identified microbial signatures may potentially be used as a biomarker for predicting OSCC or for clinical assessment of oral cancer risk.

Copyright © 2020 Zhou et al.

Institution

(Zhou, Yuan, Chen, Yang) Department of Stomatology, Qingdao Municipal Hospital, Qingdao University, Qingdao, Shandong 266071, China (Wang, Yu, Dong) Central Laboratories and Department of Gastroenterology, Qingdao Municipal Hospital, Qingdao University, Qingdao, Shandong 266071, China

(Sun) Clinical Laboratory, The Affiliated Hospital, Qingdao University, Qingdao, Shandong 266011, China

Publisher

Dove Medical Press Ltd (PO Box 300-008, Albany, 44 Corinthian Drive, Albany,Auckland 0752, New Zealand. E-mail: angela@dovepress.com)

Emtree Heading

adult; aged; alcohol consumption; amino acid metabolism; article; cancer staging; carcinogenesis; Carnobacterium; clinical article; cohort analysis; female; Fusobacterium; gene; gene sequence; glucose utilization; human; human tissue; male; middle aged; *mouth flora; *mouth mucosa; *mouth squamous cell carcinoma/et [Etiology]; Parvimonas; Peptostreptococcus; random forest; smoking; Streptococcus; Tannerella; Treponema; very elderly; biological marker/ec [Endogenous Compound]; glucose/ec [Endogenous Compound]; RNA 16S/ec [Endogenous Compound]; 16S rRNA gene.

Candidate Terms

16S rRNA gene [other term].

Drug Index Terms

biological marker / endogenous compound; glucose / endogenous compound; RNA 16S / endogenous compound.

Other Index Terms

adult; aged; alcohol consumption; amino acid metabolism; Article; cancer staging; carcinogenesis; Carnobacterium; clinical article; cohort analysis; female; Fusobacterium; gene; gene sequence; glucose utilization; human; human tissue; male; middle aged; *mouth flora; *mouth mucosa; *mouth squamous cell carcinoma / *etiology; Parvimonas; Peptostreptococcus; random forest; smoking; Streptococcus; Tannerella; Treponema; very elderly.

Link to the Ovid Full Text or citation:

[Click here for full text options](https://libaccess.mcmaster.ca/login?url=http://ovidsp.ovid.com/ovidweb.cgi?T=JS&CSC=Y&NEWS=N&PAGE=fulltext&D=emexb&AN=2004653848)

Link to the External Link Resolver:

[SFX](http://sfx.scholarsportal.info/mcmaster?sid=OVID:embase&id=pmid:&id=doi:10.2147%2FCMAR.S251021&issn=1179-1322&isbn=&volume=12&issue=&spage=5353&pages=5353-5363&date=2020&title=Cancer+Management+and+Research&atitle=Signatures+of+mucosal+microbiome+in+oral+squamous+cell+carcinoma+identified+using+a+random+forest+model&aulast=Zhou&pid=<author>Zhou+J.%3BWang+L.%3BYuan+R.%3BYu+X.%3BChen+Z.%3BYang+F.%3BSun+G.%3BDong+Q.<%2Fauthor><AN>2004653848<%2FAN><DT>Article<%2FDT>)

13.

High-altitude living shapes the skin microbiome in humans and pigs.

Zeng B., Zhao J., Guo W., Zhang S., Hua Y., Tang J., Kong F., Yang X., Fu L., Liao K., Yu X., Chen G., Jin L., Shuai S., Yang J., Si X., Ning R., Mishra S., Li Y.

Frontiers in Microbiology. 8 (OCT) (pp 1929), 2017. Article Number: 1929. Date of Publication: 06 Oct 2017.

AN: 618614696

While the skin microbiome has been shown to play important roles in health and disease in several species, the effects of altitude on the skin microbiome and how high-altitude skin microbiomes may be associated with health and disease states remains largely unknown. Using 16S rRNA marker gene sequencing, we characterized the skin microbiomes of people from two racial groups (the Tibetans and the Hans) and of three local pig breeds (Tibetan pig, Rongchang pig, and Qingyu pig) at high and low altitudes. The skin microbial communities of low-altitude pigs and humans were distinct from those of high-altitude pigs and humans, with five bacterial taxa (Arthrobacter, Paenibacillus, Carnobacterium, and two unclassified genera in families Cellulomonadaceae and Xanthomonadaceae) consistently enriched in both pigs and humans at high altitude. Alpha diversity was also significantly lower in skin samples collected from individuals living at high altitude compared to individuals at low altitude. Several of the taxa unique to high-altitude humans and pigs are known extremophiles adapted to harsh environments such as those found at high altitude. Altogether our data reveal that altitude has a significant effect on the skin microbiome of pigs and humans.

Copyright © 2017 Zeng, Zhao, Guo, Zhang, Hua, Tang, Kong, Yang, Fu, Liao, Yu, Chen, Jin, Shuai, Yang, Si, Ning, Mishra and Li.

Institution

(Zeng, Guo, Zhang, Hua, Tang, Kong, Jin, Shuai, Yang, Si, Ning, Mishra, Li) Farm Animal Genetic Resources Exploration and Innovation Key Laboratory of Sichuan Province, Sichuan Agricultural University, Chengdu, China (Zhao) Division of Agriculture, Department of Animal Science, University of Arkansas, Fayetteville, AR, United States

(Yang, Yu, Chen) Animal Husbandry and Technology Bureau of Daocheng County, Daocheng, China

(Fu) Chongqing Academy of Animal Sciences, Chongqing, China

(Liao) Pasturage Station of Tongjiang Agriculture Bureau, Bazhong, China

Publisher

Frontiers Media S.A. (E-mail: info@frontiersin.org)

Emtree Heading

Actinobacteria; *altitude; Arthrobacter; article; Carnobacterium; Cellulomonadaceae; controlled study; Corynebacterium; Firmicutes; gene sequence; Han Chinese; high throughput sequencing; human; Lactococcus; *microbiome; next generation sequencing; nonhuman; normal human; Paenibacillus; phylogenetic tree; pig; polymerase chain reaction; Proteobacteria; Tibetan (people); Xanthomonadaceae; bacterial DNA.

Drug Index Terms

bacterial DNA.

Other Index Terms

Actinobacteria; *altitude; Arthrobacter; Article; Carnobacterium; Cellulomonadaceae; controlled study; Corynebacterium; Firmicutes; gene sequence; Han Chinese; high throughput sequencing; human; Lactococcus; *microbiome; next generation sequencing; nonhuman; normal human; Paenibacillus; phylogenetic tree; pig; polymerase chain reaction; Proteobacteria; Tibetan (people); Xanthomonadaceae.

Link to the Ovid Full Text or citation:

[Click here for full text options](https://libaccess.mcmaster.ca/login?url=http://ovidsp.ovid.com/ovidweb.cgi?T=JS&CSC=Y&NEWS=N&PAGE=fulltext&D=emexb&AN=618614696)

Link to the External Link Resolver:

[SFX](http://sfx.scholarsportal.info/mcmaster?sid=OVID:embase&id=pmid:&id=doi:10.3389%2Ffmicb.2017.01929&issn=1664-302X&isbn=&volume=8&issue=OCT&spage=1929&pages=1929&date=2017&title=Frontiers+in+Microbiology&atitle=High-altitude+living+shapes+the+skin+microbiome+in+humans+and+pigs&aulast=Zeng&pid=<author>Zeng+B.%3BZhao+J.%3BGuo+W.%3BZhang+S.%3BHua+Y.%3BTang+J.%3BKong+F.%3BYang+X.%3BFu+L.%3BLiao+K.%3BYu+X.%3BChen+G.%3BJin+L.%3BShuai+S.%3BYang+J.%3BSi+X.%3BNing+R.%3BMishra+S.%3BLi+Y.<%2Fauthor><AN>618614696<%2FAN><DT>Article<%2FDT>)

14.

The microbiome of an active meat curing brine.

Woods D.F., Kozak I.M., Flynn S., O'Gara F.

Frontiers in Microbiology. 10 (JAN) (no pagination), 2019. Article Number: 3346. Date of Publication: 2019.

AN: 627188504

Traditional food products are important to our culture and heritage, and to the continued success of the food industry. Many of the production processes associated with these products have not been subjected to an in-depth microbial compositional analysis. The traditional process of curing meat, both preserves a natural protein source, as well as increasing its organoleptic qualities. One of the most important salting processes is known as Wiltshire curing. The Wiltshire process involves injecting pork with a curing solution and immersing the meat into microbial-rich brine which promotes the development of the distinct organoleptic characteristics. The important microbial component of Wiltshire brine has not been extensively characterized. We analyzed the key microbial component of Wiltshire brine by performing microbiome analysis using Next Generation Sequencing (NGS) technologies. This analysis identified the genera, Marinilactibacillus, Carnobacterium, Leuconostoc, and Vibrio as the core microflora present in Wiltshire curing brine. The important food industrial applications of these bacteria were also assessed. The bacterial diversity of the brine was investigated, and the community composition of the brine was demonstrated to change over time. New knowledge on the characterization of key microbiota associated with a productive Wiltshire brine is an important development linked to promoting enhanced quality and safety of meat processing in the food industry.

Copyright © 2019 Woods, Kozak, Flynn and O'Gara.

Institution

(Woods, Kozak, Flynn, O'Gara) Biomerit Research Centre, School of Microbiology, University College Cork, Cork, Ireland (O'Gara) Telethon Kids Institute, Subiaco, WA, Australia

(O'Gara) Human Microbiome Programme, School of Pharmacy and Biomedical Sciences, Curtin University, Perth, WA, Australia

Publisher

Frontiers Media S.A. (E-mail: info@frontiersin.org)

Emtree Heading

article; Carnobacterium; human; Leuconostoc; meat industry; *microbiome; *next generation sequencing; nonhuman; pork; Vibrio.

Other Index Terms

article; Carnobacterium; human; Leuconostoc; meat industry; *microbiome; *next generation sequencing; nonhuman; pork; Vibrio.

Link to the Ovid Full Text or citation:

[Click here for full text options](https://libaccess.mcmaster.ca/login?url=http://ovidsp.ovid.com/ovidweb.cgi?T=JS&CSC=Y&NEWS=N&PAGE=fulltext&D=emexb&AN=627188504)

Link to the External Link Resolver:

[SFX](http://sfx.scholarsportal.info/mcmaster?sid=OVID:embase&id=pmid:&id=doi:10.3389%2Ffmicb.2018.03346&issn=1664-302X&isbn=&volume=10&issue=JAN&spage=&pages=&date=2019&title=Frontiers+in+Microbiology&atitle=The+microbiome+of+an+active+meat+curing+brine&aulast=Woods&pid=<author>Woods+D.F.%3BKozak+I.M.%3BFlynn+S.%3BO'Gara+F.<%2Fauthor><AN>627188504<%2FAN><DT>Article<%2FDT>)

15.

Anti-tumor activities of probiotics in cervical cancer.

Jahanshahi M., Maleki Dana P., Badehnoosh B., Asemi Z., Hallajzadeh J., Mansournia M.A., Yousefi B., Moazzami B., Chaichian S.

Journal of Ovarian Research. 13 (1) (no pagination), 2020. Article Number: 68. Date of Publication: 11 Jun 2020.

AN: 632077859

Cervical cancer is considered as an important malignancy among women worldwide. Currently-used treatments of cervical cancer are reported to be cytotoxic for patients. Moreover, these therapies have shown some side effects which can negatively affect the lives of women suffering from this cancer. Therefore, there is need for anti-tumor agents that are less toxic than common therapeutic drugs. Besides, applying agents for preventing or reducing the side effects of cervical cancer therapies can be effective in improving the life quality of cervical cancer patients. Studies have shown that probiotics have several effects on biological processes. One of the most prominent aspects in which probiotics play a role is in the field of cancer. There are multiple studies which have focused on the functions of probiotics in diagnosis, prevention, or treatment of cancer. Besides their direct anti-tumor activities, probiotics can be used as an additional agent for enhancing or modulating other diagnostic and therapeutic methods. Herein, the effects of probiotics on cervical cancer cells are discussed, which may be useful in the prevention and treatment of this cancer. We review the studies concerned with the roles of probiotics in modulating and reducing the gastrointestinal adverse effects caused by cervical cancer therapies. Furthermore, we cover the investigations focusing on the combination of probiotics with other drugs for diagnosis or treatment of cervical cancer.

Copyright © 2020 The Author(s).

PMID

32527332 [<http://www.ncbi.nlm.nih.gov/pubmed/?term=32527332>]

Author NameID

Hallajzadeh, Jamal; ORCID: <http://orcid.org/0000-0003-0696-9229>

Institution

(Jahanshahi) Clinical Research Development Center (CRDC), Sayad Shirazi Hospital, Golestan University of Medical Sciences, Gorgan, Iran, Islamic Republic of (Maleki Dana, Asemi) Research Center for Biochemistry and Nutrition in Metabolic Diseases, Institute for Basic Sciences, Kashan University of Medical Sciences, Kashan, Iran, Islamic Republic of

(Badehnoosh) Department of Gynecology and Obstetrics, School of Medicine, Alborz University of Medical Sciences, Karaj, Iran, Islamic Republic of

(Hallajzadeh) Department of Biochemistry and Nutrition, Research Center for Evidence-Based Health Management, Maragheh University of Medical Sciences, Maragheh, Iran, Islamic Republic of

(Mansournia) Department of Epidemiology and Biostatistics, School of Public Health, Tehran University of Medical Sciences, Tehran, Iran, Islamic Republic of

(Yousefi) Stem Cell Research Center, Tabriz University of Medical Sciences, Tabriz, Iran, Islamic Republic of

(Yousefi) Department of Biochemistry, Faculty of Medicine, Tabriz University of Medical Sciences, Tabriz, Iran, Islamic Republic of

(Moazzami, Chaichian) Pars Advanced and Minimally Invasive Medical Manners Research Center, Pars Hospital, Iran University of Medical Sciences, Tehran, Iran, Islamic Republic of

Publisher

BioMed Central Ltd. (E-mail: info@biomedcentral.com)

Emtree Heading

adaptive immunity; adjuvant therapy; *antineoplastic activity; antiproliferative activity; bacteremia/si [Side Effect]; Bifidobacterium; cancer patient; cancer prevention; cancer radiotherapy; cancer screening; Carnobacterium; cholangitis/si [Side Effect]; colposcopy; constipation/si [Side Effect]; diagnostic value; diarrhea/co [Complication]; diarrhea/dt [Drug Therapy]; drug potentiation; drug safety; endocarditis/si [Side Effect]; Enterococcus; Escherichia coli; fertility preservation; flatulence/si [Side Effect]; genetic engineering; gynecologic surgery; hiccup/si [Side Effect]; human; immunomodulation; immunostimulation; infection/si [Side Effect]; innate immunity; Lactobacillus; Lactobacillus crispatus; Lactobacillus gasseri; Lactobacillus jensenii; Lactobacillus rhamnosus; Lactococcus; Leuconostoc; liver abscess/si [Side Effect]; nausea/si [Side Effect]; Oenococcus; positron emission tomography-computed tomography; priority journal; protein expression; quality of life; rash/si [Side Effect]; review; Saccharomyces; sepsis/si [Side Effect]; Streptococcus; Tetragenococcus; *uterine cervix cancer/di [Diagnosis]; *uterine cervix cancer/dt [Drug Therapy]; *uterine cervix cancer/pc [Prevention]; *uterine cervix cancer/rt [Radiotherapy]; *uterine cervix cancer/su [Surgery]; uterine cervix cytology; antineoplastic agent/dt [Drug Therapy]; bevacizumab/dt [Drug Therapy]; cisplatin/cb [Drug Combination]; cisplatin/it [Drug Interaction]; cisplatin/dt [Drug Therapy]; fluorodeoxyglucose; immunoglobulin enhancer binding protein/ec [Endogenous Compound]; interleukin 10/ec [Endogenous Compound]; interleukin 12/ec [Endogenous Compound]; interleukin 1beta/ec [Endogenous Compound]; interleukin 6/ec [Endogenous Compound]; microRNA/ec [Endogenous Compound]; *probiotic agent/ae [Adverse Drug Reaction]; *probiotic agent/cb [Drug Combination]; *probiotic agent/it [Drug Interaction]; *probiotic agent/dt [Drug Therapy]; *probiotic agent/po [Oral Drug Administration]; *probiotic agent/pd [Pharmacology]; STAT3 protein/ec [Endogenous Compound]; vasculotropin/ec [Endogenous Compound]; Wart virus vaccine/dt [Drug Therapy].

Drug Index Terms

antineoplastic agent / drug therapy; bevacizumab / drug therapy; cisplatin / drug combination / drug interaction / drug therapy; fluorodeoxyglucose; immunoglobulin enhancer binding protein / endogenous compound; interleukin 10 / endogenous compound; interleukin 12 / endogenous compound; interleukin 1beta / endogenous compound; interleukin 6 / endogenous compound; microRNA / endogenous compound; *probiotic agent / *adverse drug reaction / *drug combination / *drug interaction / *drug therapy / *oral drug administration / *pharmacology; STAT3 protein / endogenous compound; vasculotropin / endogenous compound; Wart virus vaccine / drug therapy.

Other Index Terms

adaptive immunity; adjuvant therapy; *antineoplastic activity; antiproliferative activity; bacteremia / side effect; Bifidobacterium; cancer patient; cancer prevention; cancer radiotherapy; cancer screening; Carnobacterium; cholangitis / side effect; colposcopy; constipation / side effect; diagnostic value; diarrhea / complication / drug therapy; drug potentiation; drug safety; endocarditis / side effect; Enterococcus; Escherichia coli; fertility preservation; flatulence / side effect; genetic engineering; gynecologic surgery; hiccup / side effect; human; immunomodulation; immunostimulation; infection / side effect; innate immunity; Lactobacillus; Lactobacillus crispatus; Lactobacillus gasseri; Lactobacillus jensenii; Lactobacillus rhamnosus; Lactococcus; Leuconostoc; liver abscess / side effect; nausea / side effect; Oenococcus; positron emission tomography-computed tomography; priority journal; protein expression; quality of life; rash / side effect; Review; Saccharomyces; sepsis / side effect; Streptococcus; Tetragenococcus; *uterine cervix cancer / *diagnosis / *drug therapy / *prevention / *radiotherapy / *surgery; uterine cervix cytology.

Link to the Ovid Full Text or citation:

[Click here for full text options](https://libaccess.mcmaster.ca/login?url=http://ovidsp.ovid.com/ovidweb.cgi?T=JS&CSC=Y&NEWS=N&PAGE=fulltext&D=emexb&AN=632077859)

Link to the External Link Resolver:

[SFX](http://sfx.scholarsportal.info/mcmaster?sid=OVID:embase&id=pmid:32527332&id=doi:10.1186%2Fs13048-020-00668-x&issn=1757-2215&isbn=&volume=13&issue=1&spage=&pages=&date=2020&title=Journal+of+Ovarian+Research&atitle=Anti-tumor+activities+of+probiotics+in+cervical+cancer&aulast=Jahanshahi&pid=<author>Jahanshahi+M.%3BMaleki+Dana+P.%3BBadehnoosh+B.%3BAsemi+Z.%3BHallajzadeh+J.%3BMansournia+M.A.%3BYousefi+B.%3BMoazzami+B.%3BChaichian+S.<%2Fauthor><AN>632077859<%2FAN><DT>Review<%2FDT>)

16.

Functional characterization of the alanine-serine-cysteine exchanger of Carnobacterium sp AT7.

Bartoccioni P., Fort J., Zorzano A., Errasti-Murugarren E., Palacin M.

The Journal of general physiology. 151 (4) (pp 505-517), 2019. Date of Publication: 01 Apr 2019.

AN: 627172837

Many key cell processes require prior cell uptake of amino acids from the environment, which is facilitated by cell membrane amino acid transporters such as those of the L-type amino acid transporter (LAT) subfamily. Alterations in LAT subfamily amino acid transport are associated with several human diseases, including cancer, aminoacidurias, and neurodegenerative conditions. Therefore, from the perspective of human health, there is considerable interest in obtaining structural information about these transporter proteins. We recently solved the crystal structure of the first LAT transporter, the bacterial alanine-serine-cysteine exchanger of Carnobacterium sp AT7 (BasC). Here, we provide a complete functional characterization of detergent-purified, liposome-reconstituted BasC transporter to allow the extension of the structural insights into mechanistic understanding. BasC is a sodium- and proton-independent small neutral amino acid exchanger whose substrate and inhibitor selectivity are almost identical to those previously described for the human LAT subfamily member Asc-1. Additionally, we show that, like its human counterparts, this transporter has apparent affinity asymmetry for the intra- and extracellular substrate binding sites-a key feature in the physiological role played by these proteins. BasC is an excellent paradigm of human LAT transporters and will contribute to our understanding of the molecular mechanisms underlying substrate recognition and translocation at both sides of the plasma membrane.

Copyright © 2019 Bartoccioni et al.

PMID

30696726 [<http://www.ncbi.nlm.nih.gov/pubmed/?term=30696726>]

Author NameID

Errasti-Murugarren, Ekaitz; ORCID: <http://orcid.org/0000-0002-6054-7900> Fort, Joana; ORCID: <http://orcid.org/0000-0003-0399-2116>

Institution

(Bartoccioni, Fort, Zorzano, Errasti-Murugarren, Palacin) Institute for Research in Biomedicine, Barcelona Institute of Science and Technology, Barcelona, Spain (Bartoccioni, Fort, Palacin) Centro de Investigacion Biomedica en Red de Enfermedades Raras, Barcelona, Spain

(Fort, Zorzano, Palacin) Department of Biochemistry and Molecular Biomedicine, Faculty of Biology, University of Barcelona, Barcelona, Spain

(Zorzano) Centro de Investigacion Biomedica en Red de Diabetes y Enfermedades Metabolicas Asociadas, Barcelona, Spain

Publisher

NLM (Medline)

Emtree Heading

Carnobacterium; *chemistry; gene expression regulation; *metabolism; molecular cloning; amino acid transporter; bacterial protein.

Drug Index Terms

amino acid transporter; bacterial protein.

Other Index Terms

Carnobacterium; *chemistry; gene expression regulation; *metabolism; molecular cloning.

Link to the Ovid Full Text or citation:

[Click here for full text options](https://libaccess.mcmaster.ca/login?url=http://ovidsp.ovid.com/ovidweb.cgi?T=JS&CSC=Y&NEWS=N&PAGE=fulltext&D=emexb&AN=627172837)

Link to the External Link Resolver:

[SFX](http://sfx.scholarsportal.info/mcmaster?sid=OVID:embase&id=pmid:30696726&id=doi:10.1085%2Fjgp.201812195&issn=1540-7748&isbn=&volume=151&issue=4&spage=505&pages=505-517&date=2019&title=The+Journal+of+general+physiology&atitle=Functional+characterization+of+the+alanine-serine-cysteine+exchanger+of+Carnobacterium+sp+AT7&aulast=Bartoccioni&pid=<author>Bartoccioni+P.%3BFort+J.%3BZorzano+A.%3BErrasti-Murugarren+E.%3BPalacin+M.<%2Fauthor><AN>627172837<%2FAN><DT>Article<%2FDT>)

17.

Microbiome and Functional Analysis of a Traditional Food Process: Isolation of a Novel Species (Vibrio hibernica) With Industrial Potential.

Woods D.F., Kozak I.M., O'Gara F.

Frontiers in Microbiology. 11 (no pagination), 2020. Article Number: 647. Date of Publication: 09 Apr 2020.

AN: 631537925

Traditional food preservation processes are vital for the food industry. They not only preserve a high-quality protein and nutrient source but can also provide important value-added organoleptic properties. The Wiltshire process is a traditional food curing method applied to meat, and special recognition is given to the maintenance of a live rich microflora within the curing brine. We have previously analyzed a curing brine from this traditional meat process and characterized a unique microbial core signature. The characteristic microbial community is actively maintained and includes the genera, Marinilactibacillus, Carnobacterium, Leuconostoc, and Vibrio. The bacteria present are vital for Wiltshire curing compliance. However, the exact function of this microflora is largely unknown. A microbiome profiling of three curing brines was conducted and investigated for functional traits by the robust bioinformatic tool, Tax4Fun. The key objective was to uncover putative metabolic functions associated with the live brine and to identify changes over time. The functional bioinformatic analysis revealed metabolic enrichments over time, with many of the pathways identified as being involved in organoleptic development. The core bacteria present in the brine are Lactic Acid Bacteria (LAB), with the exception of the Vibrio genus. LAB are known for their positive contribution to food processing, however, little work has been conducted on the use of Vibrio species for beneficial processes. The Vibrio genome was sequenced by Illumina MiSeq technologies and annotated in RAST. A phylogenetic reconstruction was completed using both the 16S rRNA gene and housekeeping genes, gapA, ftsZ, mreB, topA, gyrB, pyrH, recA, and rpoA. The isolated Vibrio species was defined as a unique novel species, named Vibrio hibernica strain B1.19. Metabolic profiling revealed that the bacterium has a unique substrate scope in comparison to other closely related Vibrio species tested. The possible function and industrial potential of the strain was investigated using carbohydrate metabolizing profiling under food processing relevant conditions. Vibrio hibernica is capable of metabolizing a unique carbohydrate profile at low temperatures. This characteristic provides new application options for use in the industrial food sector, as well as highlighting the key role of this bacterium in the Wiltshire curing process.

© Copyright © 2020 Woods, Kozak and O'Gara.

Institution

(Woods, Kozak, O'Gara) BIOMERIT Research Centre, School of Microbiology, University College Cork, Cork, Ireland (O'Gara) Human Microbiome Programme, School of Biomedical Sciences, Curtin Health Innovation Research Institute, Curtin University, Perth, WA, Australia

(O'Gara) Telethon Kids Institute, Perth Children's Hospital, Perth, WA, Australia

Publisher

Frontiers Media S.A. (E-mail: info@frontiersin.org)

Emtree Heading

amino acid metabolism; article; bioinformatics; carbohydrate metabolism; *food industry; food preservation; *food processing; functional genomics; gene; human; lactic acid bacterium; low temperature; metabolic fingerprinting; microbial community; *microbiome; morphotype; nonhuman; organoleptic property; phenotype; phylogeny; RecA gene; sequence alignment; sequence analysis; *Vibrio; MreB gene; topA gene; *Vibrio hibernica.

Candidate Terms

mreB gene [other term]; topA gene [other term]; *Vibrio hibernica [other term].

Other Index Terms

amino acid metabolism; Article; bioinformatics; carbohydrate metabolism; *food industry; food preservation; *food processing; functional genomics; gene; human; lactic acid bacterium; low temperature; metabolic fingerprinting; microbial community; *microbiome; morphotype; nonhuman; organoleptic property; phenotype; phylogeny; RecA gene; sequence alignment; sequence analysis; *Vibrio.

Link to the Ovid Full Text or citation:

[Click here for full text options](https://libaccess.mcmaster.ca/login?url=http://ovidsp.ovid.com/ovidweb.cgi?T=JS&CSC=Y&NEWS=N&PAGE=fulltext&D=emexb&AN=631537925)

Link to the External Link Resolver:

[SFX](http://sfx.scholarsportal.info/mcmaster?sid=OVID:embase&id=pmid:&id=doi:10.3389%2Ffmicb.2020.00647&issn=1664-302X&isbn=&volume=11&issue=&spage=&pages=&date=2020&title=Frontiers+in+Microbiology&atitle=Microbiome+and+Functional+Analysis+of+a+Traditional+Food+Process%3A+Isolation+of+a+Novel+Species+(Vibrio+hibernica)+With+Industrial+Potential&aulast=Woods&pid=<author>Woods+D.F.%3BKozak+I.M.%3BO'Gara+F.<%2Fauthor><AN>631537925<%2FAN><DT>Article<%2FDT>)

18.

Influence of pathogen contamination on beef microbiota under different storage temperatures.

Choi H., Hwang B.K., Kim B.-S., Choi S.H.

Food research international (Ottawa, Ont.). 132 (pp 109118), 2020. Date of Publication: 01 Jun 2020.

AN: 631634790

Outbreaks of food poisoning due to the consumption of contaminated beef from fast-food chains are becoming more frequent. Pathogen contamination in beef influences its spoilage as well as the development of foodborne illness. Thus, the influence of pathogen contamination on beef microbiota should be analyzed to evaluate food safety. We analyzed the influence of pathogen contamination on the shift in microbiota and the interactions between the pathogen and indigenous microbes in beef stored under different conditions. Sixty beef samples were stored at 25 degreeC and 4 degreeC for 24 h, and the shifts in microbiota were analyzed using the MiSeq system. The influence of pathogen contamination on microbiota was analyzed by artificial contamination experiments with Escherichia coli FORC_044, which was isolated from the stool of a food poisoning patient in Korea. The bacterial amounts and the proportion of Escherichia were higher when the beef was stored at 25 degreeC. Artificially contaminated Escherichia positively correlated with the indigenous microbes such as Pseudomonas, Brochothrix, Staphylococcus, Rahnella, and Rhizobium as determined by co-occurrence network analyses. Carnobacterium, a potential spoilage microbe, was negatively correlated with other microbes. The predicted functions of altered microbiota showed that the pathways related to the process of spoilage including biosynthesis of acetic acid and lactic acid increased over time. The shift in pathways was more pronounced in contaminated beef stored at 25 degreeC. Carnobacterium, Lactobacillus, and Escherichia were the main genera contributing to the shift in the relative abundance of functional genes involved in the various spoilage pathways. Our results indicated that pathogen contamination could influence beef microbiota and mediate spoilage. This study extends our understanding of the beef microbiota and provides insights into the role of pathogen and storage conditions in meat spoilage.

Copyright © 2020 Elsevier Ltd. All rights reserved.

PMID

32331694 [<http://www.ncbi.nlm.nih.gov/pubmed/?term=32331694>]

Institution

(Choi, Hwang, Choi) Department of Agricultural Biotechnology, Center for Food Safety and Toxicology, Seoul National University, Seoul 08826, South Korea (Kim) Department of Life Science, Hallym University, Chuncheon, South Korea

Publisher

NLM (Medline)

Emtree Heading

adult; article; Brochothrix; Carnobacterium; *contamination; controlled study; Escherichia coli; feces; female; food poisoning; *food safety; genetic analyzer; human; *infectious agent; Korea; Lactobacillus; major clinical study; male; *microbial interaction; nonhuman; Pseudomonas; Rahnella; Rhizobium; Staphylococcus; *storage temperature; acetic acid; lactic acid.

Drug Index Terms

acetic acid [m]; lactic acid [m].

Other Index Terms

adult [m]; article [m]; Brochothrix [m]; Carnobacterium [m]; *contamination [m]; controlled study [m]; Escherichia coli [m]; feces [m]; female [m]; food poisoning [m]; *food safety [m]; genetic analyzer [m]; human [m]; *infectious agent [m]; Korea [m]; Lactobacillus [m]; major clinical study [m]; male [m]; *microbial interaction [m]; nonhuman [m]; Pseudomonas [m]; Rahnella [m]; Rhizobium [m]; Staphylococcus [m]; *storage temperature [m].

Link to the Ovid Full Text or citation:

[Click here for full text options](https://libaccess.mcmaster.ca/login?url=http://ovidsp.ovid.com/ovidweb.cgi?T=JS&CSC=Y&NEWS=N&PAGE=fulltext&D=emexb&AN=631634790)

Link to the External Link Resolver:

[SFX](http://sfx.scholarsportal.info/mcmaster?sid=OVID:embase&id=pmid:32331694&id=doi:10.1016%2Fj.foodres.2020.109118&issn=1873-7145&isbn=&volume=132&issue=&spage=109118&pages=109118&date=2020&title=Food+research+international+(Ottawa%2C+Ont.)&atitle=Influence+of+pathogen+contamination+on+beef+microbiota+under+different+storage+temperatures&aulast=Choi&pid=<author>Choi+H.%3BHwang+B.K.%3BKim+B.-S.%3BChoi+S.H.<%2Fauthor><AN>631634790<%2FAN><DT>Article<%2FDT>)

19.

Gastric mucosal microbiota in a Mongolian population with gastric cancer and precursor conditions.

Gantuya B., El Serag H.B., Matsumoto T., Ajami N.J., Uchida T., Oyuntsetseg K., Bolor D., Yamaoka Y.

Alimentary Pharmacology and Therapeutics. 51 (8) (pp 770-780), 2020. Date of Publication: 01 Apr 2020.

AN: 2004481769

Background: Incidence and mortality of gastric cancer (GC) are high in Mongolia despite Helicobacter pylori in the Mongolian population being less virulent.

Aim(s): To evaluate gastric bacterial microbiota profiles in patients with GC and its precursor histological conditions.

Method(s): We conducted a case-control study among 48 GC and 120 noncancer patients (20 normal gastric mucosa [control], 20 gastritis, 40 with atrophy and 40 intestinal metaplasia [IM]). We performed 16S rRNA gene amplicon sequencing and compared taxonomic and functional prediction profiles based on the diagnosis group and H pylori infection status.

Result(s): The highest overall bacterial alpha diversity metrics were observed in the control group, followed by the IM and cancer groups. The gastritis and atrophy groups had the least diversity. Lactobacilli and Enterococci were the dominant genus in several cancer patients especially in the absence of H pylori. In addition, Carnobacterium, Glutamicibacter, Paeniglutamicibacter, Fusobacterium and Parvimonas were associated with GC regardless of H pylori infection. Firmicutes were decreased in the gastritis and atrophy groups and increased in the IM and cancer groups. The functional metabolic activity of the Embden-Meyerhof-Parnas pathway and the utilization of sugar, were significantly increased in cancer group compared with the noncancer group.

Conclusion(s): Microbial factors other than H pylori may play a role in Mongolian GC. We identified novel associations between GC and the genera Enterococcus, Lactobacillus, Carnobacterium, Glutamicibacter, Paeniglutamicibacter, Fusobacterium, and Parvimonas.

Copyright © 2020 John Wiley & Sons Ltd

PMID

32133670 [<http://www.ncbi.nlm.nih.gov/pubmed/?term=32133670>]

Author NameID

Gantuya, Boldbaatar; ORCID: <http://orcid.org/0000-0003-4293-0532> El Serag, Hashem B.; ORCID: <http://orcid.org/0000-0001-5964-7579>

Yamaoka, Yoshio; ORCID: <http://orcid.org/0000-0002-1222-5819>

Institution

(Gantuya, Oyuntsetseg) Department of Gastroenterology, Mongolian National University of Medical Sciences, Ulaanbaatar, Mongolia (Gantuya, Oyuntsetseg) Endoscopy Unit, Mongolia-Japan Teaching Hospital, Mongolian National University of Medical Sciences, Ulaanbaatar, Mongolia

(El Serag, Yamaoka) Department of Medicine, Gastroenterology and Hepatology Section, Baylor College of Medicine, Houston, TX, United States

(Matsumoto, Yamaoka) Department of Environmental and Preventive Medicine, Oita University of Medicine, Yufu, Japan

(Ajami) Department of Molecular Virology and Microbiology, Baylor College of Medicine, Houston, TX, United States

(Uchida) Department of Molecular Pathology, Oita University of Medicine, Yufu, Japan

(Bolor) Department of Endoscopy, Mongolian National Cancer Center Hospital, Ulaanbaatar, Mongolia

Publisher

Blackwell Publishing Ltd

Emtree Heading

adult; amplicon; article; bacterium; bacterium identification; cancer incidence; Carnobacterium; case control study; controlled study; cross-sectional study; Enterococcus; environmental factor; female; Firmicutes; Fusobacterium; gastritis; gender; genotype; Helicobacter infection; human; human tissue; *intestine flora; intestine metaplasia; Lactobacillus; major clinical study; male; microbial diversity; middle aged; Mongolian (people); nonhuman; Parvimonas; population research; prediction; priority journal; prospective study; RNA gene; RNA sequencing; stomach atrophy; *stomach cancer; stomach mucosa; tumor biopsy; RNA 16S/ec [Endogenous Compound]; tumor marker/ec [Endogenous Compound]; Glutamicibacter; Paeniglutamicibacter.

Candidate Terms

Glutamicibacter [other term]; Paeniglutamicibacter [other term].

Drug Index Terms

RNA 16S / endogenous compound; tumor marker / endogenous compound.

Other Index Terms

adult; amplicon; Article; bacterium; bacterium identification; cancer incidence; Carnobacterium; case control study; controlled study; cross-sectional study; Enterococcus; environmental factor; female; Firmicutes; Fusobacterium; gastritis; gender; genotype; Helicobacter infection; human; human tissue; *intestine flora; intestine metaplasia; Lactobacillus; major clinical study; male; microbial diversity; middle aged; Mongolian (people); nonhuman; Parvimonas; population research; prediction; priority journal; prospective study; RNA gene; RNA sequencing; stomach atrophy; *stomach cancer; stomach mucosa; tumor biopsy.

Link to the Ovid Full Text or citation:

[Click here for full text options](https://libaccess.mcmaster.ca/login?url=http://ovidsp.ovid.com/ovidweb.cgi?T=JS&CSC=Y&NEWS=N&PAGE=fulltext&D=emexb&AN=2004481769)

Link to the External Link Resolver:

[SFX](http://sfx.scholarsportal.info/mcmaster?sid=OVID:embase&id=pmid:32133670&id=doi:10.1111%2Fapt.15675&issn=0269-2813&isbn=&volume=51&issue=8&spage=770&pages=770-780&date=2020&title=Alimentary+Pharmacology+and+Therapeutics&atitle=Gastric+mucosal+microbiota+in+a+Mongolian+population+with+gastric+cancer+and+precursor+conditions&aulast=Gantuya&pid=<author>Gantuya+B.%3BEl+Serag+H.B.%3BMatsumoto+T.%3BAjami+N.J.%3BUchida+T.%3BOyuntsetseg+K.%3BBolor+D.%3BYamaoka+Y.<%2Fauthor><AN>2004481769<%2FAN><DT>Article<%2FDT>)

20.

Changes in the microbial communities of air- and water-chilled yellow-feathered broilers during storage at 2 degreeC.

Wang H., Qin X., Li X., Wang X., Gao H., Zhang C.

Food microbiology. 87 (pp 103390), 2020. Date of Publication: 01 May 2020.

AN: 630652138

Carcass chilling is a critical step in broiler processing. Understanding the effect of chilling on the bacterial communities of broilers is important, as these communities may be largely responsible for the spoilage process. This study examined the effect of chilling systems (air chilling [AC] and water chilling [WC]) and subsequent aerobic storage on the microbiota of yellow-feathered broiler carcasses using a high-throughput sequencing technique targeting the V3-V4 region of the 16S RNA gene. Evidence of the clear differences in the microbiota structures between AC and WC carcasses was illustrated by principle coordinates and heat map clustered analyses. The distinctions between the AC and WC carcass bacterial communities were more pronounced during the later storage stages. The major genera on the spoiled AC carcasses were Pseudomonas, Psychrobacter and Shewanella, whereas the major genera on the spoiled WC carcasses were Psychrobacter, Pseudomonas and Carnobacterium. These data suggest that the chilling method has a marked effect on the microbiota composition of yellow-feathered broilers along the entire storage period. The chilling method was also of great importance for surface color. However, there was no significant difference in the sensorial shelf-life of chicken when comparing the chilling methods.

Copyright © 2019. Published by Elsevier Ltd.

PMID

31948631 [<http://www.ncbi.nlm.nih.gov/pubmed/?term=31948631>]

Institution

(Wang, Qin, Li, Zhang) Institute of Food Science and Technology, Chinese Academy of Agricultural Sciences, China (Wang) Xinjiang Pagelang Food Co., Ltd, Xinjiang Province, China

(Gao) Taikun Group Co., Ltd, Xinjiang Province, China

Publisher

NLM (Medline)

Emtree Heading

animal; bacterial count; bacterium; chemistry; chicken; classification; cold; food storage; genetics; human; isolation and purification; meat; microbiology; *microflora; taste; water.

Drug Index Terms

water.

Other Index Terms

animal; bacterial count; bacterium; chemistry; chicken; classification; cold; food storage; genetics; human; isolation and purification; meat; microbiology; *microflora; taste.

Link to the Ovid Full Text or citation:

[Click here for full text options](https://libaccess.mcmaster.ca/login?url=http://ovidsp.ovid.com/ovidweb.cgi?T=JS&CSC=Y&NEWS=N&PAGE=fulltext&D=emexb&AN=630652138)

Link to the External Link Resolver:

[SFX](http://sfx.scholarsportal.info/mcmaster?sid=OVID:embase&id=pmid:31948631&id=doi:10.1016%2Fj.fm.2019.103390&issn=1095-9998&isbn=&volume=87&issue=&spage=103390&pages=103390&date=2020&title=Food+microbiology&atitle=Changes+in+the+microbial+communities+of+air-+and+water-chilled+yellow-feathered+broilers+during+storage+at+2+degreeC&aulast=Wang&pid=<author>Wang+H.%3BQin+X.%3BLi+X.%3BWang+X.%3BGao+H.%3BZhang+C.<%2Fauthor><AN>630652138<%2FAN><DT>Article<%2FDT>)

21.

Changes in intestinal microflora in digestive tract diseases during pregnancy.

Jin M., Li D., Ji R., Liu W., Xu X., Li Y.

Archives of Gynecology and Obstetrics. 301 (1) (pp 243-249), 2020. Date of Publication: 01 Jan 2020.

AN: 2003777316

Purpose: This study aimed to investigate the gut microbiome composition in pregnant women with digestive diseases to analyze the relationships between the microflora changes and digestive diseases during pregnancy.

Method(s): Fecal samples obtained from 71 pregnant women [six acute fatty liver (AF group), 21 constipation (C group), 24 excessive vomiting (V group) and 20 normal pregnancy (CP group)] and 26 non-pregnant (NP group) women were subjected to 16 s rRNA sequencing. Differential analysis of intestinal flora at the genera level was performed.

Result(s): The relative abundance of Coprobacillus, Acinetobacter, Enterococcus, Weissella and Lysinibacillus was increased in the digestive diseases (AF, C and V) groups compared with CP group, whereas that of five common genera, including Terrisporobacter, Dysgonomonas, Adlercreutzia, Fusicatenibacter and Blautia, was decreased in digestive diseases groups. Additionally, in digestive diseases (AF, C and V) groups, the abundance of 13 common genera, such as Carnobacterium, Coprobacillus and Psychrobacter, was higher than NP group, whereas that of 27 common genera, such as Blautia and Terrisporobacter, was lower than NP group. About 69 genera were differentially abundant between AF and C groups; two genera (Aerococcus and Senegalimassilia) were identified between AF and V groups; moreover, total 63 genera were obtained between C and V groups.

Conclusion(s): Our data revealed that the abundance of Acinetobacter, Enterococci, Paenibacillus, Blautia and Collinsella might be associated with the digestive diseases during pregnancy. These findings further supported the idea that targeting the gut microbiota could be a new prevention or therapeutic approach for improving digestive diseases during pregnancy.

Copyright © 2019, The Author(s).

PMID

31776707 [<http://www.ncbi.nlm.nih.gov/pubmed/?term=31776707>]

Author NameID

Li, Yanqing; ORCID: <http://orcid.org/0000-0003-0575-0399>

Institution

(Jin) Department of Anesthesiology, Qilu Hospital, Shandong University, Jinan, Shandong 250012, China (Li) Stem Cell and Regenerative Medicine Center of Shandong University, Jinan, Shandong 250012, China

(Ji) Department of Gastroenterology, Qilu Hospital, Shandong University, Jinan, Shandong 250012, China

(Liu, Xu) Department of Obstetrics and Gynecology, Center for Reproductive Medicine, Qilu Hospital, Shandong University, Jinan, Shandong 250012, China

(Li) Department of Gastroenterology, Laboratory of Translational Gastroenterology, Qilu Hospital, Shandong University, Jizhong Building, 107 Wen hua Xi Road, Lixia District, Jinan, Shandong 250012, China

Publisher

Springer

Emtree Heading

Acinetobacter; Aerococcus; article; Blautia; Carnobacterium; constipation/et [Etiology]; controlled study; disease association; drug targeting; Enterococcus; fatty liver/et [Etiology]; feces analysis; female; *gastrointestinal disease/et [Etiology]; Gram positive bacterium; human; hyperemesis/et [Etiology]; *intestine flora; Lysinibacillus; major clinical study; microbial community; *pregnancy; pregnant woman; Psychrobacter; RNA sequencing; Weissella; RNA 16S/ec [Endogenous Compound]; Adlercreutzia; Coprobacillus; Dysgonomonas; Fusicatenibacter; Senegalimassilia; Terrisporobacter.

Candidate Terms

Adlercreutzia [other term]; Coprobacillus [other term]; Dysgonomonas [other term]; Fusicatenibacter [other term]; Senegalimassilia [other term]; Terrisporobacter [other term].

Drug Index Terms

RNA 16S / endogenous compound.

Other Index Terms

Acinetobacter; Aerococcus; Article; Blautia; Carnobacterium; constipation / etiology; controlled study; disease association; drug targeting; Enterococcus; fatty liver / etiology; feces analysis; female; *gastrointestinal disease / *etiology; Gram positive bacterium; human; hyperemesis / etiology; *intestine flora; Lysinibacillus; major clinical study; microbial community; *pregnancy; pregnant woman; Psychrobacter; RNA sequencing; Weissella.

Link to the Ovid Full Text or citation:

[Click here for full text options](https://libaccess.mcmaster.ca/login?url=http://ovidsp.ovid.com/ovidweb.cgi?T=JS&CSC=Y&NEWS=N&PAGE=fulltext&D=emexb&AN=2003777316)

Link to the External Link Resolver:

[SFX](http://sfx.scholarsportal.info/mcmaster?sid=OVID:embase&id=pmid:31776707&id=doi:10.1007%2Fs00404-019-05336-0&issn=0932-0067&isbn=&volume=301&issue=1&spage=243&pages=243-249&date=2020&title=Archives+of+Gynecology+and+Obstetrics&atitle=Changes+in+intestinal+microflora+in+digestive+tract+diseases+during+pregnancy&aulast=Jin&pid=<author>Jin+M.%3BLi+D.%3BJi+R.%3BLiu+W.%3BXu+X.%3BLi+Y.<%2Fauthor><AN>2003777316<%2FAN><DT>Article<%2FDT>)

22.

Human milk as a potential source for isolation of probiotic lactic acid bacteria: A mini review.

Mustakim M., Sinawat S., Salleh S.N., Purwati E., Alias R., Syed Mohamad S.A., Mat Issa Z.

Food Research. 4 (2) (pp 274-285), 2020. Date of Publication: 2020.

AN: 2003608943

Probiotics are living microorganism that can be employed as a new approach to promote human health. These organisms are found to have attractive means for health due to their probiotic properties particularly in generating antimicrobial activity. Lactobacillus spp. is one of the main genera commonly used for probiotic purpose. Human milk is a potential source of Lactobacillus spp. and one of the criteria that found beneficial is that it is of human origin, which could be more reliable sources to be used in human. Lack of studies on isolation of probiotic bacteria from human milk was reported and some probiotic properties show a variation between strains from different regions and population. Therefore, it is important to carry out the isolation of Lactobacillus spp. from a large number of species in the genera to facilitate the finding of the most competent strain to be incorporated as probiotic agent. Moreover, to ensure suitability and compatibility for human use, the probiotic agent originated from human milk should not be an exception. In addition, certain probiotics show a great correlation with prebiotic existed in human milk to boost their function and may suggest an added value to be a suitable candidate as probiotic. This review provides an overview of studies related to human milk as the promising sources for isolation of probiotic microorganisms.

Copyright © 2019 The Authors. Published by Rynnye Lyan Resources.

Institution

(Mustakim, Sinawat, Salleh) Centre of Medical Laboratory Technology, Faculty of Health Sciences, Universiti Teknologi MARA (UiTM), Selangor Branch, Puncak Alam Campus, Bandar Puncak Alam, Selangor 42300, Malaysia (Purwati) Faculty of Animal Science, Andalas University, West Sumatra 25163, Indonesia

(Alias) Institute of Bio-IT Selangor, Universiti Selangor, Jalan Zirkon 7A, Seksyen 7, Shah Alam, Selangor 40000, Malaysia

(Syed Mohamad) Centre of Biology, Faculty of Applied Science, Universiti Teknologi MARA (UiTM), Selangor Branch, Shah Alam Campus, Shah Alam, Selangor 40000, Malaysia

(Mat Issa) Department of Foodservice Management, Faculty of Hotel and Tourism Management, Universiti Teknologi MARA (UiTM), Selangor Branch, Puncak Alam Campus, Selangor 42300, Malaysia

Publisher

Rynnye Lyan Resources

Emtree Heading

antibiotic resistance; antimicrobial activity; bacterium contamination; bacterium identification; *bacterium isolation; Bifidobacterium; breast feeding; *breast milk; Carnobacterium; diarrhea; Enterococcus; Escherichia coli; human; immune system; intestine flora; *lactic acid bacterium; Lactobacillus; Lactobacillus acidophilus; Lactobacillus plantarum; Lactococcus; Leuconostoc; microbial community; nonhuman; Pediococcus; short survey; Streptococcus; virus infection; Weissella; antibiotic agent; antiinfective agent; *probiotic agent.

Drug Index Terms

antibiotic agent; antiinfective agent; *probiotic agent.

Other Index Terms

antibiotic resistance; antimicrobial activity; bacterium contamination; bacterium identification; *bacterium isolation; Bifidobacterium; breast feeding; *breast milk; Carnobacterium; diarrhea; Enterococcus; Escherichia coli; human; immune system; intestine flora; *lactic acid bacterium; Lactobacillus; Lactobacillus acidophilus; Lactobacillus plantarum; Lactococcus; Leuconostoc; microbial community; nonhuman; Pediococcus; Short Survey; Streptococcus; virus infection; Weissella.

Link to the Ovid Full Text or citation:

[Click here for full text options](https://libaccess.mcmaster.ca/login?url=http://ovidsp.ovid.com/ovidweb.cgi?T=JS&CSC=Y&NEWS=N&PAGE=fulltext&D=emexa&AN=2003608943)

Link to the External Link Resolver:

[SFX](http://sfx.scholarsportal.info/mcmaster?sid=OVID:embase&id=pmid:&id=doi:10.26656%2Ffr.2017.4%25282%2529.307&issn=2550-2166&isbn=&volume=4&issue=2&spage=274&pages=274-285&date=2020&title=Food+Research&atitle=Human+milk+as+a+potential+source+for+isolation+of+probiotic+lactic+acid+bacteria%3A+A+mini+review&aulast=Mustakim&pid=<author>Mustakim+M.%3BSinawat+S.%3BSalleh+S.N.%3BPurwati+E.%3BAlias+R.%3BSyed+Mohamad+S.A.%3BMat+Issa+Z.<%2Fauthor><AN>2003608943<%2FAN><DT>Short+Survey<%2FDT>)

23.

Identification of bacteria associated with periapical abscesses of primary teeth by sequence analysis of 16S rDNA clone libraries.

Zhang W., Chen Y., Shi Q., Hou B., Yang Q.

Microbial Pathogenesis. 141 (no pagination), 2020. Article Number: 103954. Date of Publication: April 2020.

AN: 2004694322

Objective: This study aims to detect the predominant bacteria in acute periapical abscesses of primary teeth using culture-independent molecular methods based on 16S ribosomal DNA cloning.

Method(s): Purulent material was collected from nine children diagnosed with abscesses of endodontic origin. DNA was extracted and the 16S rRNA gene amplified with universal primer pairs 27F and 1492R. Amplified genes were cloned, sequenced by Applied Biosystems, and identified by comparison with known 16S rRNA gene sequences.

Result(s): Bacterial DNA was present in all nine purulence samples. A total of 681 clones were classified into 8 phyla, 78 genera, and 125 species/phylotypes. The phyla were Firmicutes, Proteobacteria, Fusobacteria, Bacteroidetes, Actinobacteria, Tenericutes, Deinococcus-Thermus, and Spirochaetes. The most dominant genera were Streptococcus (13.3%), Fusobacterium (11.8%), Parvimonas (7.8%), Prevotella (6.7%), Sphingomonas (5.8%), and Hafnia (5.2%). Fusobacterium nucleatum (11.5%), Parvimonas micra (7.8%), Streptococcus intermedius (6.6%), Sphingomonas echinoides (5.3%), Hafnia alvei (5.2%), and Citrobacter freundii (4.9%) were the most common species/phylotypes. Among these species/phylotypes, F.nucleatum was the most prevalent (7/9). C. freundii, Carnobacterium maltaromaticum, and H. alvei were seldom detected species in acute periapical abscesses but had relatively high abundance and prevalence.

Conclusion(s): Acute periapical abscesses are polymicrobial with certain prevalent bacteria, especially anaerobic bacterium. The most predominant and prevalent bacteria of acute periapical abscesses in children was F. nucleatum.

Copyright © 2019

PMID

31891793 [<http://www.ncbi.nlm.nih.gov/pubmed/?term=31891793>]

Institution

(Zhang, Chen, Yang) Beijing Institute for Dental Research, Capital Medical University, School of Stomatology, Beijing, China (Shi) Department of Pediatric Dentistry, Capital Medical University, School of Stomatology, Beijing, China

(Hou) Department of Endodontics, Capital Medical University, School of Stomatology, Beijing, China

Publisher

Academic Press

Emtree Heading

Actinobacteria; anaerobic bacterium; article; *bacterium identification; Bacteroidetes; Carnobacterium maltaromaticum; child; Citrobacter freundii; clinical article; deciduous tooth; Deinococcus; disease association; DNA extraction; female; Fusobacterium nucleatum; gene amplification; Hafnia alvei; human; male; *molecular cloning; Mollicutes; nonhuman; nucleotide sequence; Parvimonas micra; population abundance; prevalence; Prevotella; priority journal; Proteobacteria; RNA gene; RNA sequence; *Sanger sequencing; Sphingomonas; spirochete; Streptococcus intermedius; Thermus; tissue culture; *tooth abscess; tooth injury; bacterial DNA/ec [Endogenous Compound]; *DNA 16S/ec [Endogenous Compound]; primer RNA/ec [Endogenous Compound]; Sphingomonas echinoides.

Candidate Terms

Sphingomonas echinoides [other term].

Drug Index Terms

bacterial DNA / endogenous compound; *DNA 16S / *endogenous compound; primer RNA / endogenous compound.

Other Index Terms

Actinobacteria; anaerobic bacterium; Article; *bacterium identification; Bacteroidetes; Carnobacterium maltaromaticum; child; Citrobacter freundii; clinical article; deciduous tooth; Deinococcus; disease association; DNA extraction; female; Fusobacterium nucleatum; gene amplification; Hafnia alvei; human; male; *molecular cloning; Mollicutes; nonhuman; nucleotide sequence; Parvimonas micra; population abundance; prevalence; Prevotella; priority journal; Proteobacteria; RNA gene; RNA sequence; *Sanger sequencing; Sphingomonas; spirochete; Streptococcus intermedius; Thermus; tissue culture; *tooth abscess; tooth injury.

Link to the Ovid Full Text or citation:

[Click here for full text options](https://libaccess.mcmaster.ca/login?url=http://ovidsp.ovid.com/ovidweb.cgi?T=JS&CSC=Y&NEWS=N&PAGE=fulltext&D=emexa&AN=2004694322)

Link to the External Link Resolver:

[SFX](http://sfx.scholarsportal.info/mcmaster?sid=OVID:embase&id=pmid:31891793&id=doi:10.1016%2Fj.micpath.2019.103954&issn=0882-4010&isbn=&volume=141&issue=&spage=&pages=&date=2020&title=Microbial+Pathogenesis&atitle=Identification+of+bacteria+associated+with+periapical+abscesses+of+primary+teeth+by+sequence+analysis+of+16S+rDNA+clone+libraries&aulast=Zhang&pid=<author>Zhang+W.%3BChen+Y.%3BShi+Q.%3BHou+B.%3BYang+Q.<%2Fauthor><AN>2004694322<%2FAN><DT>Article<%2FDT>)

24.

Antimicrobials for food and feed; a bacteriocin perspective.

O'Connor P.M., Kuniyoshi T.M., Oliveira R.P., Hill C., Ross R.P., Cotter P.D.

Current Opinion in Biotechnology. 61 (pp 160-167), 2020. Date of Publication: February 2020.

AN: 2004656419

Bacteriocins are natural antimicrobials that have been consumed via fermented foods for millennia and have been the focus of renewed efforts to identify novel bacteriocins, and their producing microorganisms, for use as food biopreservatives and other applications. Bioengineering bacteriocins or combining bacteriocins with multiple modes of action (hurdle approach) can enhance their preservative effect and reduces the incidence of antimicrobial resistance. In addition to their role as food biopreservatives, bacteriocins are gaining credibility as health modulators, due to their ability to regulate the gut microbiota, which is strongly associated with human wellbeing. Indeed the strengthening link between the gut microbiota and obesity make bacteriocins ideal alternatives to Animal Growth Promoters (AGP) in animal feed also. Here we review recent advances in bacteriocin research that will contribute to the development of functional foods and feeds as a consequence of roles in food biopreservation and human/animal health.

Copyright © 2020 Elsevier Ltd

PMID

31968296 [<http://www.ncbi.nlm.nih.gov/pubmed/?term=31968296>]

Author NameID

O'Connor, Paula M; ORCID: <http://orcid.org/0000-0001-6462-2077>

Institution

(O'Connor, Kuniyoshi, Cotter) Teagasc Food Research Centre, Moorepark, Fermoy, Co. Cork, Ireland (O'Connor, Hill, Ross, Cotter) APC Microbiome Ireland, University College Cork, Ireland

(Kuniyoshi, Oliveira) Biochemical and Pharmaceutical Technology Department, Faculty of Pharmaceutical Sciences, University of Sao Paulo, Av. Lineu Prestes 580, Sao Paulo 05508-900, Brazil

(Hill, Ross) School of Microbiology, University College Cork, Ireland

Publisher

Elsevier Ltd

Emtree Heading

*animal food; antibiotic resistance; bacterial count; bacterial growth; bacterial strain; bacterium culture; bioengineering; Carnobacterium maltaromaticum; cell membrane; disease predisposition; Enterococcus; enzyme degradation; fermentation; *food biotechnology; food intake; *food preservation; *food processing; food safety; horizontal gene transfer; human; innate immunity; Lactobacillus curvatus; Lactobacillus plantarum; Lactococcus lactis; membrane permeability; molecular docking; nonhuman; oxidation; Pediococcus acidilactici; priority journal; protein function; review; shelf life; transcriptomics; bacterial protein; *bacteriocin; kanamycin; nisin; pediocin; unclassified drug; vancomycin; Enterococcus mundtii; nisin resistance protein.

Candidate Terms

Enterococcus mundtii [other term]; nisin resistance protein [drug term].

Drug Index Terms

bacterial protein; *bacteriocin; kanamycin; nisin; pediocin; unclassified drug; vancomycin.

Other Index Terms

*animal food; antibiotic resistance; bacterial count; bacterial growth; bacterial strain; bacterium culture; bioengineering; Carnobacterium maltaromaticum; cell membrane; disease predisposition; Enterococcus; enzyme degradation; fermentation; *food biotechnology; food intake; *food preservation; *food processing; food safety; horizontal gene transfer; human; innate immunity; Lactobacillus curvatus; Lactobacillus plantarum; Lactococcus lactis; membrane permeability; molecular docking; nonhuman; oxidation; Pediococcus acidilactici; priority journal; protein function; Review; shelf life; transcriptomics.

Link to the Ovid Full Text or citation:

[Click here for full text options](https://libaccess.mcmaster.ca/login?url=http://ovidsp.ovid.com/ovidweb.cgi?T=JS&CSC=Y&NEWS=N&PAGE=fulltext&D=emexa&AN=2004656419)

Link to the External Link Resolver:

[SFX](http://sfx.scholarsportal.info/mcmaster?sid=OVID:embase&id=pmid:31968296&id=doi:10.1016%2Fj.copbio.2019.12.023&issn=0958-1669&isbn=&volume=61&issue=&spage=160&pages=160-167&date=2020&title=Current+Opinion+in+Biotechnology&atitle=Antimicrobials+for+food+and+feed%3B+a+bacteriocin+perspective&aulast=O'Connor&pid=<author>O'Connor+P.M.%3BKuniyoshi+T.M.%3BOliveira+R.P.%3BHill+C.%3BRoss+R.P.%3BCotter+P.D.<%2Fauthor><AN>2004656419<%2FAN><DT>Review<%2FDT>)

25.

Understanding the association between the human gut, oral and skin microbiome and the Ayurvedic concept of prakriti.

Chaudhari D., Dhotre D., Agarwal D., Gondhali A., Nagarkar A., Lad V., Patil U., Juvekar S., Sinkar V., Shouche Y.

Journal of Biosciences. 44 (5) (no pagination), 2019. Article Number: 112. Date of Publication: 01 Dec 2019.

AN: 2003192509

Ayurveda is one of the ancient systems of medicine which is widely practised as a personalized scientific approach towards the general wellness. Ayurvedic prakriti is broadly defined as the phenotypes which are determined on the basis of physical, psychological and physiological traits irrespective of their social, ethnic, dietary and geographical stature. Prakriti is the constitution of a person, which comprises vata, pitta, and kapha and is a key determinant of how one individual is different from the other. Human microbiome is considered the 'latest discovered' human organ and microbiome research reiterates the fundamental principles of Ayurveda for creating a healthy gut environment by maintaining the individual-specific microbiome. Hence, it is important to understand the association of human microbiome with the Ayurvedic prakriti of an individual. Here, we provide a comprehensive analysis of human microbiome from the gut, oral and skin samples of healthy individuals (n=18) by 16S rRNA gene-based metagenomics using standard QIIME pipeline. In the three different prakriti samples differential abundance of Bacteroides, Desulfovibrio, Parabacteroides, Slackia, and Succinivibrio was observed in the gut microbiome. Analysis also revealed prakriti-specific presence of Mogibacterium, Propionibacterium, Pyramidobacter, Rhodococcus in the kapha prakriti individuals Planomicrobium, Hyphomicrobium, Novosphingobium in the pitta prakriti individuals and Carnobacterium, Robiginitalea, Cetobacterium, Psychrobacter in the vata prakriti individuals. Similarly, the oral and skin microbiome also revealed presence of prakriti-specific differential abundance of diverse bacterial genera. Prakriti-specific presence of bacterial taxa was recorded and only 42% microbiome in the oral samples and 52% microbiome in the skin samples were shared. Bacteria known for preventing gut inflammation by digesting the resistant starch were abundant in the pitta prakriti individuals, who are more prone to develop gut-inflammation-related disorders. In summary, human gut, oral and skin microbiome showed presence or high abundance of few bacterial taxa across three prakriti types, suggesting their specific physiological importance.

Copyright © 2019, Indian Academy of Sciences.

PMID

31719221 [<http://www.ncbi.nlm.nih.gov/pubmed/?term=31719221>]

Institution

(Chaudhari, Dhotre, Sinkar, Shouche) National Centre for Microbial Resource, National Centre for Cell Science, Central Tower, Sai Trinity Building Garware Circle, Sutarwadi, Pashan, Pune, India (Agarwal, Gondhali, Nagarkar, Lad, Juvekar) Vadu Rural Health Program, KEM Hospital Research Centre, Pune, India

(Chaudhari, Patil) R. C. Patel ASC College, Shirpur, Dhule, India

(Patil) Department of Microbiology, Government Institute of Science, Aurangabad 431 004, India

Publisher

Springer

Emtree Heading

article; *Ayurveda; bacterial gene; bacterium; Bacteroides; Carnobacterium; comprehension; Desulfovibrio; human; Hyphomicrobium; *intestine flora; metagenomics; *mouth flora; nonhuman; Parabacteroides; *population abundance; Propionibacterium; Psychrobacter; Rhodococcus; RNA sequence; *skin flora; *species difference; taxon; RNA 16S/ec [Endogenous Compound]; Cetobacterium; Mogibacterium; Novosphingobium; planomicrobium; Pyramidobacter; Robiginitalea; Slackia; Succinivibrio.

Candidate Terms

Cetobacterium [other term]; Mogibacterium [other term]; Novosphingobium [other term]; Planomicrobium [other term]; Pyramidobacter [other term]; Robiginitalea [other term]; Slackia [other term]; Succinivibrio [other term].

Drug Index Terms

RNA 16S / endogenous compound.

Other Index Terms

Article; *Ayurveda; bacterial gene; bacterium; Bacteroides; Carnobacterium; comprehension; Desulfovibrio; human; Hyphomicrobium; *intestine flora; metagenomics; *mouth flora; nonhuman; Parabacteroides; *population abundance; Propionibacterium; Psychrobacter; Rhodococcus; RNA sequence; *skin flora; *species difference; taxon.

Link to the Ovid Full Text or citation:

[Click here for full text options](https://libaccess.mcmaster.ca/login?url=http://ovidsp.ovid.com/ovidweb.cgi?T=JS&CSC=Y&NEWS=N&PAGE=fulltext&D=emexa&AN=2003192509)

Link to the External Link Resolver:

[SFX](http://sfx.scholarsportal.info/mcmaster?sid=OVID:embase&id=pmid:31719221&id=doi:10.1007%2Fs12038-019-9939-6&issn=0250-5991&isbn=&volume=44&issue=5&spage=&pages=&date=2019&title=Journal+of+Biosciences&atitle=Understanding+the+association+between+the+human+gut%2C+oral+and+skin+microbiome+and+the+Ayurvedic+concept+of+prakriti&aulast=Chaudhari&pid=<author>Chaudhari+D.%3BDhotre+D.%3BAgarwal+D.%3BGondhali+A.%3BNagarkar+A.%3BLad+V.%3BPatil+U.%3BJuvekar+S.%3BSinkar+V.%3BShouche+Y.<%2Fauthor><AN>2003192509<%2FAN><DT>Article<%2FDT>)

26.

The skin microbiome of vertebrates.

Ross A.A., Rodrigues Hoffmann A., Neufeld J.D.

Microbiome. 7 (1) (no pagination), 2019. Article Number: 79. Date of Publication: 23 May 2019.

AN: 627856401

The skin constitutes the primary physical barrier between vertebrates and their external environment. Characterization of skin microorganisms is essential for understanding how a host evolves in association with its microbial symbionts, modeling immune system development, diagnosing illnesses, and exploring the origins of potential zoonoses that affect humans. Although many studies have characterized the human microbiome with culture-independent techniques, far less is known about the skin microbiome of other mammals, amphibians, birds, fish, and reptiles. The aim of this review is to summarize studies that have leveraged high-Throughput sequencing to better understand the skin microorganisms that associate with members of classes within the subphylum Vertebrata. Specifically, links will be explored between the skin microbiome and vertebrate characteristics, including geographic location, biological sex, animal interactions, diet, captivity, maternal transfer, and disease. Recent literature on parallel patterns between host evolutionary history and their skin microbial communities, or phylosymbiosis, will also be analyzed. These factors must be considered when designing future microbiome studies to ensure that the conclusions drawn from basic research translate into useful applications, such as probiotics and successful conservation strategies for endangered and threatened animals.

Copyright © 2019 The Author(s).

PMID

31122279 [<http://www.ncbi.nlm.nih.gov/pubmed/?term=31122279>]

Author NameID

Neufeld, Josh D.; ORCID: <http://orcid.org/0000-0002-8722-8571>

Institution

(Ross, Neufeld) University of Waterloo, 200 University Avenue West, Waterloo, ON N2L 3G1, Canada (Ross) Ontario Veterinary College, University of Guelph, 419 Gordon St, Guelph, ON N1G 2W1, Canada

(Rodrigues Hoffmann) Department of Veterinary Pathobiology, College of Veterinary Medicine and Biomedical Sciences, Texas AandM University, 660 Raymond Stotzer Pkwy, College Station, TX, United States

Publisher

BioMed Central Ltd. (E-mail: info@biomedcentral.com)

Emtree Heading

Actinobacteria; Alternaria; Amphibia; apocrine gland; Arthrobacter; Bacteroidetes; bird; breathing; Carnobacterium; Cellulomonadaceae; Chloroflexi; Clostridia; Corynebacterium; Dichelobacter nodosus; dysbiosis; fish; Fusobacteria; high throughput sequencing; mammal; microbial community; microbial diversity; *microbiome; moisture; nonhuman; osmoregulation; Paenibacillus; pH; priority journal; Proteobacteria; Pseudomonas; Psychrobacter; reptile; review; skin infection; symbiont; taxonomy; vertebrate; Xanthomonadaceae; zoonosis; probiotic agent; RNA 16S/ec [Endogenous Compound].

Drug Index Terms

probiotic agent; RNA 16S / endogenous compound.

Other Index Terms

Actinobacteria; Alternaria; Amphibia; apocrine gland; Arthrobacter; Bacteroidetes; bird; breathing; Carnobacterium; Cellulomonadaceae; Chloroflexi; Clostridia; Corynebacterium; Dichelobacter nodosus; dysbiosis; fish; Fusobacteria; high throughput sequencing; mammal; microbial community; microbial diversity; *microbiome; moisture; nonhuman; osmoregulation; Paenibacillus; pH; priority journal; Proteobacteria; Pseudomonas; Psychrobacter; reptile; Review; skin infection; symbiont; taxonomy; vertebrate; Xanthomonadaceae; zoonosis.

Link to the Ovid Full Text or citation:

[Click here for full text options](https://libaccess.mcmaster.ca/login?url=http://ovidsp.ovid.com/ovidweb.cgi?T=JS&CSC=Y&NEWS=N&PAGE=fulltext&D=emexa&AN=627856401)

Link to the External Link Resolver:

[SFX](http://sfx.scholarsportal.info/mcmaster?sid=OVID:embase&id=pmid:31122279&id=doi:10.1186%2Fs40168-019-0694-6&issn=2049-2618&isbn=&volume=7&issue=1&spage=&pages=&date=2019&title=Microbiome&atitle=The+skin+microbiome+of+vertebrates&aulast=Ross&pid=<author>Ross+A.A.%3BRodrigues+Hoffmann+A.%3BNeufeld+J.D.<%2Fauthor><AN>627856401<%2FAN><DT>Review<%2FDT>)

27.

Perinatal factors affect the gut microbiota up to four years after birth.

Fouhy F., Watkins C., Hill C.J., O'Shea C.-A., Nagle B., Dempsey E.M., O'Toole P.W., Ross R.P., Ryan C.A., Stanton C.

Nature Communications. 10 (1) (no pagination), 2019. Article Number: 1517. Date of Publication: 01 Dec 2019.

AN: 627037538

Perinatal factors impact gut microbiota development in early life, however, little is known on the effects of these factors on microbes in later life. Here we sequence DNA from faecal samples of children over the first four years and reveal a perpetual evolution of the gut microbiota during this period. The significant impact of gestational age at birth and delivery mode on gut microbiota progression is evident in the first four years of life, while no measurable effects of antibiotics are found in the first year. Microbiota profiles are also characteristic in children dependant on gestational age and maturity. Full term delivery is characterised by Bacteroides (year one), Parabacteroides (year two) and Christensenellaceae (year four). Preterm delivery is characterised by Lactobacillus (year one), Streptococcus (year two) and Carnobacterium (year four). This study reveals that the gut retains distinct microbial profiles of perinatal factors up to four years of age.

Copyright © 2019, The Author(s).

PMID

30944304 [<http://www.ncbi.nlm.nih.gov/pubmed/?term=30944304>]

Author NameID

Fouhy, Fiona; ORCID: <http://orcid.org/0000-0002-8285-7694>

Institution

(Fouhy, Watkins, Hill, O'Toole, Ross, Ryan, Stanton) APC Microbiome Ireland, Cork T12 YT20, Ireland (Fouhy, Watkins, Nagle, Stanton) Teagasc Food Research Centre, Moorepark, Fermoy, Co, Cork P61 C996, Ireland

(O'Shea, Dempsey, Ryan) Department of Neonatology, Cork University Maternity Hospital, Cork T12 YE02, Ireland

(Dempsey) INFANT Centre, University College Cork, Cork T12 YT20, Ireland

(O'Toole, Ross) School of Microbiology, University College Cork, Cork T12 YT20, Ireland

Publisher

Nature Publishing Group (Houndmills, Basingstoke, Hampshire RG21 6XS, United Kingdom)

Emtree Heading

article; Bacteroidaceae; Bacteroidetes; bioinformatics; breast milk; Carnobacterium; child; DNA extraction; feces microflora; female; gestational age; human; infant; *intestine flora; Lactobacillus; major clinical study; male; microbial community; microbial diversity; microbiome; newborn; Parabacteroides; *perinatal care; prematurity; prenatal diagnosis; redundancy analysis; Streptococcus; vaginal delivery; RNA 16S.

Drug Index Terms

RNA 16S.

Other Index Terms

Article; Bacteroidaceae; Bacteroidetes; bioinformatics; breast milk; Carnobacterium; child; DNA extraction; feces microflora; female; gestational age; human; infant; *intestine flora; Lactobacillus; major clinical study; male; microbial community; microbial diversity; microbiome; newborn; Parabacteroides; *perinatal care; prematurity; prenatal diagnosis; redundancy analysis; Streptococcus; vaginal delivery.

Link to the Ovid Full Text or citation:

[Click here for full text options](https://libaccess.mcmaster.ca/login?url=http://ovidsp.ovid.com/ovidweb.cgi?T=JS&CSC=Y&NEWS=N&PAGE=fulltext&D=emexa&AN=627037538)

Link to the External Link Resolver:

[SFX](http://sfx.scholarsportal.info/mcmaster?sid=OVID:embase&id=pmid:30944304&id=doi:10.1038%2Fs41467-019-09252-4&issn=2041-1723&isbn=&volume=10&issue=1&spage=&pages=&date=2019&title=Nature+Communications&atitle=Perinatal+factors+affect+the+gut+microbiota+up+to+four+years+after+birth&aulast=Fouhy&pid=<author>Fouhy+F.%3BWatkins+C.%3BHill+C.J.%3BO'Shea+C.-A.%3BNagle+B.%3BDempsey+E.M.%3BO'Toole+P.W.%3BRoss+R.P.%3BRyan+C.A.%3BStanton+C.<%2Fauthor><AN>627037538<%2FAN><DT>Article<%2FDT>)

28.

The microbiome of prostate fluid is associated with prostate cancer.

Ma X., Chi C., Fan L., Dong B., Shao X., Xie S., Li M., Xue W.

Frontiers in Microbiology. 10 (JULY) (no pagination), 2019. Article Number: 1664. Date of Publication: 2019.

AN: 628631458

Objectives: To explore the microbiome of the prostatic fluid in high prostate-specific antigen (PSA) patients.

Patients and Methods: The microbiome profiles of prostatic fluid samples from 32 prostate cancer (PCa) patients and 27 non-PCa people were assessed. Microbiome analysis was assessed by massive 16S ribosomal RNA gene sequencing.

Result(s): Compared with the NCA group, the microbial diversity was lower in the CA group. There were no specific microbial species in the CA group or NCA group. However, many species, such as those in the genera Alkaliphilus, Enterobacter, Lactococcus, Cronobacter, Carnobacterium, and Streptococcus, showed a significant difference between the CA group and NCA group.

Conclusion(s): The prostate contains reduced bacteria, suggesting a possible pathophysiological correlation between the composition of the microbiome and PCa. Meanwhile, this study uncovered that the microbiome may be beneficial in maintaining the stability of the microenvironment of the prostate and provides interesting perspectives for the identification of novel biomarkers in high-PSA patients.

Copyright © 2019 Ma, Chi, Fan, Dong, Shao, Xie, Li and Xue. This is an open-access article distributed under the terms of the Creative Commons Attribution License (CC BY). The use, distribution or reproduction in other forums is permitted, provided the original author(s) and the copyright owner(s) are credited and that the original publication in this journal is cited, in accordance with accepted academic practice. No use, distribution or reproduction is permitted which does not comply with these terms.

Institution

(Ma, Chi, Fan, Dong, Shao, Xue) Department of Urology, Renji Hospital, School of Medicine, Shanghai Jiao Tong University, Shanghai, China (Ma, Li) Department of Clinical Laboratory, Renji Hospital, School of Medicine, Shanghai Jiao Tong University, Shanghai, China

(Xie) Department of Ultrasound in Medicine, Renji Hospital, School of Medicine, Shanghai Jiao Tong University, Shanghai, China

Publisher

Frontiers Media S.A. (E-mail: info@frontiersin.org)

Emtree Heading

aged; article; Bacillus; Carnobacterium; clinical article; Clostridiaceae; controlled study; Cronobacter; diagnostic accuracy; DNA extraction; Enterobacter; gene sequence; Gleason score; human tissue; International Prostate Symptom Score; Lactococcus; male; microanalysis; microbial diversity; *microbiome; pathophysiology; polymerase chain reaction; prostate biopsy; *prostate cancer; *prostate fluid; species composition; Streptococcus; tumor microenvironment; bacterial DNA/ec [Endogenous Compound]; prostate specific antigen/ec [Endogenous Compound]; RNA 16S/ec [Endogenous Compound]; DNA purification kit; genetic analyzer; polymerase chain reaction system; spectrophotometer; Alkaliphilus; Geneamp 9700; NanoDrop 2000; QuantiFluor.

Candidate Terms

Alkaliphilus [other term]; GeneAmp 9700 [device term]; Nanodrop 2000 [device term]; QuantiFluor [device term].

Device Index Terms

DNA purification kit; genetic analyzer; polymerase chain reaction system; spectrophotometer.

Drug Index Terms

bacterial DNA / endogenous compound; prostate specific antigen / endogenous compound; RNA 16S / endogenous compound.

Other Index Terms

aged; Article; Bacillus; Carnobacterium; clinical article; Clostridiaceae; controlled study; Cronobacter; diagnostic accuracy; DNA extraction; Enterobacter; gene sequence; Gleason score; human tissue; International Prostate Symptom Score; Lactococcus; male; microanalysis; microbial diversity; *microbiome; pathophysiology; polymerase chain reaction; prostate biopsy; *prostate cancer; *prostate fluid; species composition; Streptococcus; tumor microenvironment.

Link to the Ovid Full Text or citation:

[Click here for full text options](https://libaccess.mcmaster.ca/login?url=http://ovidsp.ovid.com/ovidweb.cgi?T=JS&CSC=Y&NEWS=N&PAGE=fulltext&D=emexa&AN=628631458)

Link to the External Link Resolver:

[SFX](http://sfx.scholarsportal.info/mcmaster?sid=OVID:embase&id=pmid:&id=doi:10.3389%2Ffmicb.2019.01664&issn=1664-302X&isbn=&volume=10&issue=JULY&spage=&pages=&date=2019&title=Frontiers+in+Microbiology&atitle=The+microbiome+of+prostate+fluid+is+associated+with+prostate+cancer&aulast=Ma&pid=<author>Ma+X.%3BChi+C.%3BFan+L.%3BDong+B.%3BShao+X.%3BXie+S.%3BLi+M.%3BXue+W.<%2Fauthor><AN>628631458<%2FAN><DT>Article<%2FDT>)

29.

Antibiotic-producing beneficial bacteria in the gut of the burying beetle nicrophorus vespilloides.

Heise P., Liu Y., Degenkolb T., Vogel H., Schaberle T.F., Vilcinskas A.

Frontiers in Microbiology. 10 (MAY) (no pagination), 2019. Article Number: 1178. Date of Publication: 2019.

AN: 628416923

The increasing prevalence of antibiotic-resistant human pathogens is a growing public concern and there is intense pressure to identify new antibacterial compounds that can be developed into antibiotics with novel mode of action. Evolutionary theory predicts that insects that have evolved to occupy sophisticated ecological niches by feeding and reproducing on carcasses will depend on their gut microbiome to prevent colonization by invading pathogens taken up with the diet. This inspired our hypothesis that the complex interactions between the core microbiome and the more flexible microbial communities dependent on the environment may promote the outsourcing of antibiotic synthesis to beneficial microbes. We tested this hypothesis by cultivating and characterizing bacteria isolated from the gut of the burying beetle Nicrophorus vespilloides, which feeds and reproduces on small vertebrate carcasses buried in the soil to avoid competitors such as fly maggots. The extracts of isolated bacteria were screened for activity against human pathogens such as Escherichia coli, Pseudomonas aeruginosa, Staphylococcus aureus, and Candida albicans. More than 400 strains were isolated, among which the crude extract of Serratia marcescens 2MH3-2 displayed promising activity against Staphylococcus aureus. Bioactivity-guided fractionation enabled purification of the primary antimicrobial compound of the extract. By LC-MS and NMR experiments, it was identified as serrawettin W2 (C38H61N5O9), the antibacterial and nematostatic activity of which was corroborated in our study. We postulate that this antibiotic could contribute to the control of both bacteria and phoretic nematodes in the gut, which compete for food when transferred to the carcass. Our study shows that the gut microbiome of N. vespilloides is a promising resource for the screening of antibiotic-producing bacteria.

Copyright © 2007 - 2019 Frontiers Media S.A. All Rights Reserved.

Institution

(Heise, Schaberle, Vilcinskas) Department of Bioresources, Fraunhofer Institute for Molecular Biology and Applied Ecology, Giessen, Germany (Liu, Degenkolb, Schaberle, Vilcinskas) Institute for Insect Biotechnology, Justus Liebig University Giessen, Giessen, Germany

(Vogel) Department of Entomology, Max Planck Institute for Chemical Ecology, Jena, Germany

(Schaberle) German Center for Infection Research (DZIF), Partner Site Giessen-Marburg-Langen, Giessen, Germany

Publisher

Frontiers Media S.A. (E-mail: info@frontiersin.org)

Emtree Heading

antibacterial activity; antimicrobial activity; article; bacterial colonization; bacterium culture; bacterium identification; bacterium isolation; *beetle; *beneficial organism; biological activity; Candida albicans; Carnobacterium; controlled study; drug identification; *drug manufacture; drug screening; drug synthesis; ED50; electrospray; Enterococcus; Escherichia coli; fluorescence correlation spectroscopy; Gammaproteobacteria; genetic analysis; growth inhibition; heteronuclear single quantum coherence; high performance liquid chromatography; *intestine flora; Lactococcus; liquid chromatography-mass spectrometry; Listeria monocytogenes; microbial community; minimum inhibitory concentration; mouse; Mus musculus; nonhuman; nuclear magnetic resonance imaging; polymerase chain reaction; protein purification; Pseudomonas; Pseudomonas aeruginosa; Serratia marcescens; Staphylococcus aureus; Stenotrophomonas; time of flight mass spectrometry; Xanthomonadales; ampicillin; antibiotic agent; antiinfective agent; carbenicillin; DNA 16S/ec [Endogenous Compound]; ivermectin; kanamycin; RNA 16S/ec [Endogenous Compound]; unclassified drug; insect rearing; Nicrophorus vespilloides; serrawettin W2/an [Drug Analysis]; serrawettin W2/dv [Drug Development].

Candidate Terms

insect rearing [other term]; Nicrophorus vespilloides [other term]; serrawettin W2 / drug analysis / drug development [drug term].

Drug Index Terms

ampicillin; antibiotic agent; antiinfective agent; carbenicillin; DNA 16S / endogenous compound; ivermectin; kanamycin; RNA 16S / endogenous compound; unclassified drug.

Other Index Terms

antibacterial activity; antimicrobial activity; Article; bacterial colonization; bacterium culture; bacterium identification; bacterium isolation; *beetle; *beneficial organism; biological activity; Candida albicans; Carnobacterium; controlled study; drug identification; *drug manufacture; drug screening; drug synthesis; ED50; electrospray; Enterococcus; Escherichia coli; fluorescence correlation spectroscopy; Gammaproteobacteria; genetic analysis; growth inhibition; heteronuclear single quantum coherence; high performance liquid chromatography; *intestine flora; Lactococcus; liquid chromatography-mass spectrometry; Listeria monocytogenes; microbial community; minimum inhibitory concentration; mouse; Mus musculus; nonhuman; nuclear magnetic resonance imaging; polymerase chain reaction; protein purification; Pseudomonas; Pseudomonas aeruginosa; Serratia marcescens; Staphylococcus aureus; Stenotrophomonas; time of flight mass spectrometry; Xanthomonadales.

Link to the Ovid Full Text or citation:

[Click here for full text options](https://libaccess.mcmaster.ca/login?url=http://ovidsp.ovid.com/ovidweb.cgi?T=JS&CSC=Y&NEWS=N&PAGE=fulltext&D=emexa&AN=628416923)

Link to the External Link Resolver:

[SFX](http://sfx.scholarsportal.info/mcmaster?sid=OVID:embase&id=pmid:&id=doi:10.3389%2Ffmicb.2019.01178&issn=1664-302X&isbn=&volume=10&issue=MAY&spage=&pages=&date=2019&title=Frontiers+in+Microbiology&atitle=Antibiotic-producing+beneficial+bacteria+in+the+gut+of+the+burying+beetle+nicrophorus+vespilloides&aulast=Heise&pid=<author>Heise+P.%3BLiu+Y.%3BDegenkolb+T.%3BVogel+H.%3BSchaberle+T.F.%3BVilcinskas+A.<%2Fauthor><AN>628416923<%2FAN><DT>Article<%2FDT>)

30.

The influence of probiotic supplementation in broiler chickens on population and carcass contamination with Campylobacter spp. - Field study.

Smialek M., Burchardt S., Koncicki A.

Research in Veterinary Science. 118 (pp 312-316), 2018. Date of Publication: June 2018.

AN: 2000559012

Campylobacter spp. is a food-borne pathogen occurring all over the world. According to European Food Safety Authority, in Europe, in 2015 the number of recorded and confirmed cases of Campylobacter spp. infections in humans has reached approximately 230,000. Poultry and poultry meat are considered to be the main sources of human infection, which triggers the discussion about the possibility of imposing obligatory control of Campylobacter spp. population at the level of primary poultry production. Recently, the use of probiotics in poultry is considered as a very promising alternative that could reduce infection rate in broiler chickens with Campylobacter spp. Although, there were some approaches made in vivo, up to date, there were no studies that would evaluate those issues under field conditions. A study was carried out in order to determine the feasibility of reducing infection rate in broiler chickens with Campylobacter spp. raised at a commercial farm, by the addition of multispecies probiotic (Lavipan, JHJ, Poland) that composed of Lactococcus lactis, Carnobacterium divergens, Lactobacillus casei, Lactobacillus plantarum and Saccharomyces cerevisae to the feed. Results of our study indicate that probiotic (Lavipan) added to a feed for broiler chickens was capable to reduce the extent of Campylobacter spp. invasion in the gastrointestinal tract of birds and, resultantly, to diminish contamination level in bird environment, which eventually contributed to the improved hygienic parameters of analyzed poultry carcasses. Additionally, this probiotic displayed promising immunomodulatory properties that may improve the effectiveness of the specific prophylaxis program applied in a flock of broiler chickens.

Copyright © 2018 The Authors

PMID

29567598 [<http://www.ncbi.nlm.nih.gov/pubmed/?term=29567598>]

Institution

(Smialek, Koncicki) Department of Poultry Diseases, University of Warmia and Mazury, ul. Oczapowskiego 13/13, Olsztyn 10-719, Poland (Burchardt) JHJ Sp. Z.O.O., Nowa Wies 11, Gizalki 63-308, Poland

Publisher

Elsevier B.V.

Emtree Heading

animal food; article; *broiler; *Campylobacter; carcass; Carnobacterium; chicken; *diet supplementation; feasibility study; gastrointestinal tract; immunomodulation; infection rate; Lactobacillus casei; Lactobacillus plantarum; Lactococcus lactis; microbial population dynamics; nonhuman; risk reduction; Saccharomyces cerevisiae; species composition; species invasion; *probiotic agent/pd [Pharmacology]; Carnobacterium divergens; lavipan.

Candidate Terms

Carnobacterium divergens [other term]; lavipan [drug term].

Drug Index Terms

*probiotic agent / *pharmacology.

Other Index Terms

animal food; Article; *broiler; *Campylobacter; carcass; Carnobacterium; chicken; *diet supplementation; feasibility study; gastrointestinal tract; immunomodulation; infection rate; Lactobacillus casei; Lactobacillus plantarum; Lactococcus lactis; microbial population dynamics; nonhuman; risk reduction; Saccharomyces cerevisiae; species composition; species invasion.

Drug Trade Names and Manufacturers

JHJ [Poland], lavipan: JHJ [Poland]

Link to the Ovid Full Text or citation:

[Click here for full text options](https://libaccess.mcmaster.ca/login?url=http://ovidsp.ovid.com/ovidweb.cgi?T=JS&CSC=Y&NEWS=N&PAGE=fulltext&D=emed19&AN=2000559012)

Link to the External Link Resolver:

[SFX](http://sfx.scholarsportal.info/mcmaster?sid=OVID:embase&id=pmid:29567598&id=doi:10.1016%2Fj.rvsc.2018.03.009&issn=0034-5288&isbn=&volume=118&issue=&spage=312&pages=312-316&date=2018&title=Research+in+Veterinary+Science&atitle=The+influence+of+probiotic+supplementation+in+broiler+chickens+on+population+and+carcass+contamination+with+Campylobacter+spp.+-+Field+study&aulast=Smialek&pid=<author>Smialek+M.%3BBurchardt+S.%3BKoncicki+A.<%2Fauthor><AN>2000559012<%2FAN><DT>Article<%2FDT>)

31.

Patients with acne vulgaris have a distinct gut microbiota in comparison with healthy controls.

Deng Y., Wang H., Zhou J., Mou Y., Wang G., Xiong X.

Acta Dermato-Venereologica. 98 (8) (pp 783-790), 2018. Date of Publication: 2018.

AN: 624552077

Acne vulgaris has been postulated to have a gastrointestinal mechanism; however, little is known about gut microbiota dysfunction in this condition. The aim of this cross-sectional study was to investigate whether the gut microbiota is altered in acne. Faecal bacterial diversity was analysed in 43 patients with acne and 43 controls, using hypervariable tag sequencing of the V3-V4 region of the 16S rDNA gene. Distinct differences were found in microbial diversity between patients with acne and controls (Shannon diversity index (p = 0.009) and Simpson diversity index (p = 0.01)). At the phylum level, the abundance of Firmicutes was lower in the patient group, but that of Bacteroidiain was higher. The most significantly depleted taxa in acne were Clostridia, Clostridiales, Lachnospiraceae and Ruminococcaceae genera, which are potentially beneficial. In conclusion, patients with acne vulgaris have gut microbial dysbiosis; further study is needed to understand its role in the pathogenesis of acne.

Copyright © 2018 Acta Dermato-Venereologica.

PMID

29756631 [<http://www.ncbi.nlm.nih.gov/pubmed/?term=29756631>]

Institution

(Deng, Wang, Xiong) Department of Dermatology & STD, The Affiliated Hospital of Southwest Medical University, Luzhou, Sichuan, China (Wang) Department of Infectious Disease, Peking University First Hospital, Beijing, China

(Zhou) Biomedical Research Institute, Shenzhen Peking University, The Hong Kong University of Science and Technology Medical Center, Shenzhen, Guangdong Province, China

(Mou) Department of Dermatology & STD, Affiliated Hospital of North Sichuan Medical College, Nanchong, Sichuan, China

Publisher

Medical Journals/Acta D-V (St: Johannesgatan 22A, Uppsala SE-753 12, Sweden)

Emtree Heading

*acne vulgaris/et [Etiology]; adult; Aerococcaceae; Aerococcus; article; Bacillales; Bacillus; bacterial structures; Bacteroidales; Bacteroidetes; Bilophila; Blautia; carbohydrate synthesis; Carnobacteriaceae; Carnobacterium; clinical article; Clostridia; Clostridiaceae; Clostridiales; controlled study; dairy product; Desulfovibrionaceae; Desulfovibrionales; disease duration; disease severity; female; Firmicutes; Granulicatella; human; *intestine flora; Lachnospiraceae; lipogenesis; male; microbial diversity; Paenibacillaceae; phylum; Planococcaceae; priority journal; Ruminococcaceae; bacterial DNA/ec [Endogenous Compound]; DNA 16S/ec [Endogenous Compound]; glycan/ec [Endogenous Compound]; glycosphingolipid/ec [Endogenous Compound].

Drug Index Terms

bacterial DNA / endogenous compound; DNA 16S / endogenous compound; glycan / endogenous compound; glycosphingolipid / endogenous compound.

Other Index Terms

*acne vulgaris / *etiology; adult; Aerococcaceae; Aerococcus; Article; Bacillales; Bacillus; bacterial structures; Bacteroidales; Bacteroidetes; Bilophila; Blautia; carbohydrate synthesis; Carnobacteriaceae; Carnobacterium; clinical article; Clostridia; Clostridiaceae; Clostridiales; controlled study; dairy product; Desulfovibrionaceae; Desulfovibrionales; disease duration; disease severity; female; Firmicutes; Granulicatella; human; *intestine flora; Lachnospiraceae; lipogenesis; male; microbial diversity; Paenibacillaceae; phylum; Planococcaceae; priority journal; Ruminococcaceae.

Link to the Ovid Full Text or citation:

[Click here for full text options](https://libaccess.mcmaster.ca/login?url=http://ovidsp.ovid.com/ovidweb.cgi?T=JS&CSC=Y&NEWS=N&PAGE=fulltext&D=emed19&AN=624552077)

Link to the External Link Resolver:

[SFX](http://sfx.scholarsportal.info/mcmaster?sid=OVID:embase&id=pmid:29756631&id=doi:10.2340%2F00015555-2968&issn=0001-5555&isbn=&volume=98&issue=8&spage=783&pages=783-790&date=2018&title=Acta+Dermato-Venereologica&atitle=Patients+with+acne+vulgaris+have+a+distinct+gut+microbiota+in+comparison+with+healthy+controls&aulast=Deng&pid=<author>Deng+Y.%3BWang+H.%3BZhou+J.%3BMou+Y.%3BWang+G.%3BXiong+X.<%2Fauthor><AN>624552077<%2FAN><DT>Article<%2FDT>)

32.

The action of probiotic microorganisms on chemical contaminants in milk.

Wochner K.F., Becker-Algeri T.A., Colla E., Badiale-Furlong E., Drunkler D.A.

Critical Reviews in Microbiology. 44 (1) (pp 112-123), 2018. Date of Publication: 02 Jan 2018.

AN: 620405996

Consumption of milk and its derivatives is an important food habit in the diet of all age groups. However, there has been increasing concern about physical, biological (pathogenic or spoilage microorganisms) and chemical (metals, pesticides and mycotoxins) contaminants in milk and dairy products. Considering the high levels of consumption, it is important that milk and dairy products are free from toxic compounds. Microbial degradation as a strategy for chemical decontamination is an emerging biotechnological approach that is considered a safe and inexpensive practice. The decontaminant activity of probiotic microorganisms is associated with fermentation, antibiosis and the ability of the microbial cell wall to bind to the contaminant. Exploiting the potential of microorganisms for chemical decontamination will further leverage its application in the food industry.

Copyright © 2017 Informa UK Limited, trading as Taylor & Francis Group.

PMID

28537817 [<http://www.ncbi.nlm.nih.gov/pubmed/?term=28537817>]

Institution

(Wochner, Colla, Drunkler) Post Graduate Program in Food Technology (PPGTA), Federal Technological University of Parana (UTFPR), Medianeira, Brazil (Becker-Algeri, Badiale-Furlong) Post Graduate Program in Engineering and Science of Food, Department of Food Chemistry, Federal University of Rio Grande (FURG), Rio Grande, Brazil

Publisher

Taylor and Francis Ltd (E-mail: healthcare.enquiries@informa.com)

Emtree Heading

Aerococcus; bacterial viability; Bifidobacterium; Bifidobacterium animalis; Bifidobacterium bifidum; Bifidobacterium breve; Bifidobacterium longum; Bifidobacterium longum subsp. infantis; Carnobacterium; *dairy product; *decontamination; Enterococcus; *food contamination; food industry; human; Lactobacillus; Lactobacillus fermentum; Lactobacillus plantarum; Lactobacillus rhamnosus; Lactococcus; Leuconostoc; microbial degradation; *milk; nonhuman; Oenococcus; Pediococcus; priority journal; Propionibacterium freudenreichii; review; Saccharomyces boulardii; Streptococcus; Tetragenococcus; Weissella; aflatoxin; heavy metal; mycotoxin; pesticide; *probiotic agent; bifidobacterium thermophilum.

Candidate Terms

Bifidobacterium thermophilum [other term].

Drug Index Terms

aflatoxin; heavy metal; mycotoxin; pesticide; *probiotic agent.

Other Index Terms

Aerococcus; bacterial viability; Bifidobacterium; Bifidobacterium animalis; Bifidobacterium bifidum; Bifidobacterium breve; Bifidobacterium longum; Bifidobacterium longum subsp. infantis; Carnobacterium; *dairy product; *decontamination; Enterococcus; *food contamination; food industry; human; Lactobacillus; Lactobacillus fermentum; Lactobacillus plantarum; Lactobacillus rhamnosus; Lactococcus; Leuconostoc; microbial degradation; *milk; nonhuman; Oenococcus; Pediococcus; priority journal; Propionibacterium freudenreichii; Review; Saccharomyces boulardii; Streptococcus; Tetragenococcus; Weissella.

Link to the Ovid Full Text or citation:

[Click here for full text options](https://libaccess.mcmaster.ca/login?url=http://ovidsp.ovid.com/ovidweb.cgi?T=JS&CSC=Y&NEWS=N&PAGE=fulltext&D=emed19&AN=620405996)

Link to the External Link Resolver:

[SFX](http://sfx.scholarsportal.info/mcmaster?sid=OVID:embase&id=pmid:28537817&id=doi:10.1080%2F1040841X.2017.1329275&issn=1040-841X&isbn=&volume=44&issue=1&spage=112&pages=112-123&date=2018&title=Critical+Reviews+in+Microbiology&atitle=The+action+of+probiotic+microorganisms+on+chemical+contaminants+in+milk&aulast=Wochner&pid=<author>Wochner+K.F.%3BBecker-Algeri+T.A.%3BColla+E.%3BBadiale-Furlong+E.%3BDrunkler+D.A.<%2Fauthor><AN>620405996<%2FAN><DT>Review<%2FDT>)

33.

Lactic acid bacteria in finfish-An update.

Ringo E., Hoseinifar S.H., Ghosh K., Doan H.V., Beck B.R., Song S.K.

Frontiers in Microbiology. 9 (AUG) (no pagination), 2018. Article Number: 1818. Date of Publication: 10 Aug 2018.

AN: 623390073

A complex and dynamic community of microorganisms, play important roles within the fish gastrointestinal (GI) tract. Of the bacteria colonizing the GI tract, are lactic acid bacteria (LAB) generally considered as favorable microorganism due to their abilities to stimulating host GI development, digestive function, mucosal tolerance, stimulating immune response, and improved disease resistance. In early finfish studies, were culture-dependent methods used to enumerate bacterial population levels within the GI tract. However, due to limitations by using culture methods, culture-independent techniques have been used during the last decade. These investigations have revealed the presence of Lactobacillus, Lactococcus, Leuconostoc, Enterococcus, Streptococcus, Carnobacterium, Weissella, and Pediococcus as indigenous species. Numerous strains of LAB isolated from finfish are able to produce antibacterial substances toward different potential fish pathogenic bacteria as well as human pathogens. LAB are revealed be the most promising bacterial genera as probiotic in aquaculture. During the decade numerous investigations are performed on evaluation of probiotic properties of different genus and species of LAB. Except limited contradictory reports, most of administered strains displayed beneficial effects on both, growth-and reproductive performance, immune responses and disease resistance of finfish. This eventually led to industrial scale up and introduction LAB-based commercial probiotics. Pathogenic LAB belonging to the genera Streptococcus, Enterococcus, Lactobacillus, Carnobacterium, and Lactococcus have been detected from ascites, kidney, liver, heart, and spleen of several finfish species. These pathogenic bacteria will be addressed in present review which includes their impacts on finfish aquaculture, possible routes for treatment. Finfish share many common structures and functions of the immune system with warm-blooded animals, although apparent differences exist. This similarity in the immune system may result in many shared LAB effects between finfish and land animals. LAB-fed fish show an increase in innate immune activities leading to disease resistances: neutrophil activity, lysozyme secretion, phagocytosis, and production of pro-inflammatory cytokines (IL-1beta, IL-6, IL-8, and TNF-alpha). However, some LAB strains preferentially induces IL-10 instead, a potent anti-inflammatory cytokine. These results indicate that LAB may vary in their immunological effects depending on the species and hosts. So far, the immunological studies using LAB have been focused on their effects on innate immunity. However, these studies need to be further extended by investigating their involvement in the modulation of adaptive immunity. The present review paper focuses on recent findings in the field of isolation and detection of LAB, their administration as probiotic in aquaculture and their interaction with fish immune responses. Furthermore, the mode of action of probiotics on finfish are discussed.

Copyright © 2018 Ringo, Hoseinifar, Ghosh, Doan, Beck and Song.

Institution

(Ringo) Faculty of Bioscience, Fisheries and Economics, Norwegian College of Fishery Science, UiT The Arctic University of Norway, Tromso, Norway (Hoseinifar) Department of Fisheries, Faculty of Fisheries and Environmental Sciences, Gorgan University of Agricultural Sciences and Natural Resources, Gorgan, Iran, Islamic Republic of

(Ghosh) Aquaculture Laboratory, Department of Zoology, The University of Burdwan, Bardhaman, India

(Doan) Department of Animal and Aquatic Sciences, Faculty of Agriculture, Chiang Mai University, Chiang Mai, Thailand

(Beck, Song) School of Life Science, Handong University, Pohang, South Korea

Publisher

Frontiers Media S.A. (E-mail: info@frontiersin.org)

Emtree Heading

adaptive immunity; antibacterial activity; aquaculture; bacterial strain; Bifidobacterium; Carnobacterium; down regulation; Enterococcus; gastrointestinal tract; immune response; innate immunity; *lactic acid bacterium; Lactobacillus acidophilus; Lactobacillus casei; Lactobacillus delbrueckii; Lactobacillus paracasei; Lactobacillus plantarum; Lactobacillus rhamnosus; Lactococcus; Leuconostoc; microbial community; nonhuman; Pediococcus; Pediococcus acidilactici; Pediococcus pentosaceus; review; Streptococcus; upregulation; Weissella; bacteriocin; probiotic agent; Vagococcus.

Candidate Terms

Vagococcus [other term].

Drug Index Terms

bacteriocin; probiotic agent.

Other Index Terms

adaptive immunity; antibacterial activity; aquaculture; bacterial strain; Bifidobacterium; Carnobacterium; down regulation; Enterococcus; gastrointestinal tract; immune response; innate immunity; *lactic acid bacterium; Lactobacillus acidophilus; Lactobacillus casei; Lactobacillus delbrueckii; Lactobacillus paracasei; Lactobacillus plantarum; Lactobacillus rhamnosus; Lactococcus; Leuconostoc; microbial community; nonhuman; Pediococcus; Pediococcus acidilactici; Pediococcus pentosaceus; Review; Streptococcus; upregulation; Weissella.

Link to the Ovid Full Text or citation:

[Click here for full text options](https://libaccess.mcmaster.ca/login?url=http://ovidsp.ovid.com/ovidweb.cgi?T=JS&CSC=Y&NEWS=N&PAGE=fulltext&D=emed19&AN=623390073)

Link to the External Link Resolver:

[SFX](http://sfx.scholarsportal.info/mcmaster?sid=OVID:embase&id=pmid:&id=doi:10.3389%2Ffmicb.2018.01818&issn=1664-302X&isbn=&volume=9&issue=AUG&spage=&pages=&date=2018&title=Frontiers+in+Microbiology&atitle=Lactic+acid+bacteria+in+finfish-An+update&aulast=Ringo&pid=<author>Ringo+E.%3BHoseinifar+S.H.%3BGhosh+K.%3BDoan+H.V.%3BBeck+B.R.%3BSong+S.K.<%2Fauthor><AN>623390073<%2FAN><DT>Review<%2FDT>)

34.

The medical functions of probiotics and their role in clinical nutrition.

Mancuskova T., Medvedova A., Ozbolt M., Valik L.

Current Nutrition and Food Science. 14 (1) (pp 3-10), 2018. Date of Publication: 2018.

AN: 620683174

Background: Lactic acid bacteria (LAB) have been attracting attention of food microbiologists for more than a hundred years. An attention of researchers and society is paid to their ability to influent human health, to preserve foods and to extend their shelf life. Currently, LAB group includes a large number of different bacterial genera: Lactobacillus, Lactococcus, Streptococcus, Pediococcus, Leuconostoc, Enterococcus, Oenococcus, Aerococcus, Carnobacterium, Vagococcus, Weisella and Tetragenococcus. Some LAB species can provide a positive effect on consumer's health due to changes in milk composition leading to a better digestibility of some milk components. Positive effect of LAB is also linked with restoring the intestinal balance in favour of beneficial microorganisms. These LAB strains are known as probiotics. Probiotics, which are used in foods and nutrition supplements frequently, are isolated especially from gastrointestinal tract of humans and mammals, the vaginas of healthy women, breast and cow milk or fruits and vegetables. Every strain included in the list of probiotic bacteria must necessarily have a proven positive effect on human health. Other important features of probiotics are that they must be precisely identified and classified, cannot be pathogenic, must be able to survive in the digestive tract and must be stable during food processing. The effect of probiotic bacteria on the host health is based on their direct antagonism to pathogens and potential pathogens, as well as on the indirect expulsion of harmful microorganisms from the host. The adherence to the binding sites of mucous membranes, translocation blocking, competing for nutrients, the production of antimicrobial metabolites and the stimulation of the host immune system are applied here. The usage of probiotics for direct treatment of any disease is rare. Usually they are applied as an adjuvant therapy, as an eliminator/reducer of medication side effects, for the prevention of diseases and for long-term support of host immunity. Disorders treated by probiotic bacteria include lactose intolerance, diarrhoea and constipation, infections, obesity and diabetes mellitus, allergic and autoimmune diseases, respiration disorders, oncologic diseases, neurological and psychological disorders. On the other hand, some trials found out that probiotics had no effect on gut bacteria compared to inactive placebo.

Conclusion(s): There is a growing interest in probiotics and defining the proper use of these agents. Although probiotics seemed to have beneficial effects in many clinical trials, the efficiency of probiotics is strain and dose-dependent. Also, there are numerous clinical and methodological differences between trials (e.g., strain used, dose, administration) that make it difficult to draw a conclusion about the efficacy. Additional research in the form of well designed, randomized, double-blind, placebo-controlled trials is needed. Although probiotics appear to be safe in general, caution should be used when administered to specific subgroups of patients such as the immunocom-promised, the elderly, and children.

Copyright © 2018 Bentham Science Publishers.

Institution

(Mancuskova, Medvedova, Valik) Department of Nutrition and Food Quality Assessment, Faculty of Chemical and Food Technology, Slovak University of Technology in Bratislava, Bratislava, Slovakia (Ozbolt) Department of Biotechnology, University of Rijeka, Rijeka, Croatia

Publisher

Bentham Science Publishers B.V. (P.O. Box 294, Bussum 1400 AG, Netherlands)

Emtree Heading

allergy/dt [Drug Therapy]; autoimmune disease/dt [Drug Therapy]; bacterial translocation; breathing disorder/dt [Drug Therapy]; constipation/dt [Drug Therapy]; diabetes mellitus/dt [Drug Therapy]; diarrhea/dt [Drug Therapy]; drug mechanism; human; immunomodulation; infection/dt [Drug Therapy]; lactic acid bacterium; lactose intolerance/dt [Drug Therapy]; malignant neoplasm/dt [Drug Therapy]; mental disease/dt [Drug Therapy]; neurologic disease/dt [Drug Therapy]; nonhuman; *nutrition; obesity/dt [Drug Therapy]; priority journal; review; antiinfective agent; *probiotic agent/dt [Drug Therapy]; *probiotic agent/pd [Pharmacology].

Drug Index Terms

antiinfective agent; *probiotic agent / *drug therapy / *pharmacology.

Other Index Terms

allergy / drug therapy; autoimmune disease / drug therapy; bacterial translocation; breathing disorder / drug therapy; constipation / drug therapy; diabetes mellitus / drug therapy; diarrhea / drug therapy; drug mechanism; human; immunomodulation; infection / drug therapy; lactic acid bacterium; lactose intolerance / drug therapy; malignant neoplasm / drug therapy; mental disease / drug therapy; neurologic disease / drug therapy; nonhuman; *nutrition; obesity / drug therapy; priority journal; Review.

Link to the Ovid Full Text or citation:

[Click here for full text options](https://libaccess.mcmaster.ca/login?url=http://ovidsp.ovid.com/ovidweb.cgi?T=JS&CSC=Y&NEWS=N&PAGE=fulltext&D=emed19&AN=620683174)

Link to the External Link Resolver:

[SFX](http://sfx.scholarsportal.info/mcmaster?sid=OVID:embase&id=pmid:&id=doi:10.2174%2F1573401313666170405152905&issn=1573-4013&isbn=&volume=14&issue=1&spage=3&pages=3-10&date=2018&title=Current+Nutrition+and+Food+Science&atitle=The+medical+functions+of+probiotics+and+their+role+in+clinical+nutrition&aulast=Mancuskova&pid=<author>Mancuskova+T.%3BMedvedova+A.%3BOzbolt+M.%3BValik+L.<%2Fauthor><AN>620683174<%2FAN><DT>Review<%2FDT>)

35.

The fecal bacterial microbiota of bats; Slovenia.

Vengust M., Knapic T., Weese J.S.

PLoS ONE. 13 (5) (no pagination), 2018. Article Number: e0196728. Date of Publication: May 2018.

AN: 622249934

Methods Fecal samples were collected from 92 bats in Slovenia, consisting of 12 different species, and the bacterial microbiota was assessed via next generation sequencing of the 16S rRNA gene V4 region. Results Sequences were assigned to 28 different phyla, but only Proteobacteria, Firmicutes, Bacteroidetes and Actinobacteria accounted for 1% of sequences. One phylum (Proteobacteria), one class (Gammaproteobacteria), three orders (Pseudomonadales, Lactobacillales, Bacil-lales), four families (Enterobacteriaceae, Pseudomonadaceae, Staphylococcaceae, Carno-bacteriaceae), and five genera (Pseudomonas, Staphylococcus, Carnobacterium, an unclassified Enterobacteriaceae, Acinetobacter) accounted for 50% of sequences. There were no significant differences in the relative abundances of any phyla between bat species, but various differences were noted at lower taxonomic levels, such as Enterobacteriaceae (P = 0.007, most abundant in M. blythii), Pseudomonadaceae (P = 0.007, most abundant in Rhinolophus hipposideros) and Chlamydiaceae (P = 0.04, most abundant in Myotis myotis). There were significant differences in richness between species in both adults and juveniles/ subadults, but there was no impact of sex on any alpha diversity index. When only adults are considered, there were significant differences in community membership between M. blythii and M. emarginatus (P = 0.011), and M. blythii and R. hipposideros (P = 0.004). There were also significant differences in community structure between M. blythii and M. emarginatus (P = 0.025), and M. blythii and R. hipposideros (P = 0.026). When adults of the four main species were compared, 14 OTUs were identified as differentially abundant using LEfSe. Only one difference was identified when comparing R. hipposideros adults and juvenile/subadults, with Klebsiella over-represented in the younger bats. Conclusions Bats have a complex and diverse microbiota with a high relative abundance of Proteobacteria. The relevance of this difference is unclear and requires further study. Differences in the microbiota were observed between bat species, perhaps reflecting different diets and environmental exposures.

Copyright © 2018 Vengust et al. This is an open access article distributed under the terms of the Creative Commons Attribution License, which permits unrestricted use, distribution, and reproduction in any medium, provided the original author and source are credited.

PMID

29791473 [<http://www.ncbi.nlm.nih.gov/pubmed/?term=29791473>]

Institution

(Vengust) Veterinary Faculty, University of Ljubljana, Ljubljana, Slovenia (Knapic) Slovenian Museum of Natural History, Ljubljana, Slovenia

(Weese) Dept of Pathobiology, Ontario Veterinary College, University of Guelph, Guelph, ON, Canada

Publisher

Public Library of Science (E-mail: plos@plos.org)

Emtree Heading

Acinetobacter; Actinobacteria; adult; article; *bacterial flora; Bacteroidetes; Carnobacterium; Chlamydiaceae; community structure; diet; environmental exposure; human; juvenile; Klebsiella; Myotis myotis; nonhuman; phylum; Pseudomonas; *Slovenia; Staphylococcus; subadult.

Other Index Terms

Acinetobacter; Actinobacteria; adult; article; *bacterial flora; Bacteroidetes; Carnobacterium; Chlamydiaceae; community structure; diet; environmental exposure; human; juvenile; Klebsiella; Myotis myotis; nonhuman; phylum; Pseudomonas; *Slovenia; Staphylococcus; subadult.

Link to the Ovid Full Text or citation:

[Click here for full text options](https://libaccess.mcmaster.ca/login?url=http://ovidsp.ovid.com/ovidweb.cgi?T=JS&CSC=Y&NEWS=N&PAGE=fulltext&D=emed19&AN=622249934)

Link to the External Link Resolver:

[SFX](http://sfx.scholarsportal.info/mcmaster?sid=OVID:embase&id=pmid:29791473&id=doi:10.1371%2Fjournal.pone.0196728&issn=1932-6203&isbn=&volume=13&issue=5&spage=&pages=&date=2018&title=PLoS+ONE&atitle=The+fecal+bacterial+microbiota+of+bats%3B+Slovenia&aulast=Vengust&pid=<author>Vengust+M.%3BKnapic+T.%3BWeese+J.S.<%2Fauthor><AN>622249934<%2FAN><DT>Article<%2FDT>)

36.

Microbial diversity in two traditional bacterial douchi from Gansu province in northwest China using Illumina sequencing.

Zhang W., Luo Q., Zhu Y., Ma J., Cao L., Yang M., Wen P., Zhang Z., He X.

PLoS ONE. 13 (3) (no pagination), 2018. Article Number: e0194876. Date of Publication: March 2018.

AN: 621359412

Douchi has been consumed as a flavoring ingredient for centuries. During production of douchi, numerous microorganisms play important roles in the hydrolysis and conversion of proteins and starch, which are related to the quality and flavor of the end product. Therefore, in the present study, the microbial diversity in two types of home-made traditional bacterial douchi from Gansu province in northwest China was studied by high-throughput sequencing, and a corresponding analysis of the bacterial and fungal communities were conducted. The results showed that geography may have impacted the fungal diversity and the bacterial and fungal species richness in the samples. The results also showed that the microbial community was significantly different in samples of different origin and the difference in the microbial community at the genus level was greater than at phylum level. Two dominant bacterial genera (Bacillus and Ignatzschineria) were common to the two samples, both of which had a relative abundance of more than 1%. Four bacterial genera (Staphylococcus, Aerococcus, Geobacillus, and Jeotgalicoccus) were dominant only in the sample from Qingyang, while another four (Carnobacterium, Proteus, Aneurinibacillus, and Enterococcus) were dominant only in the sample from Longnan. Two dominant fungal genera (Pichia and Candida) were shared by the two samples. Additionally, two genera (Rhodosporidium and Yarrowia) were dominant only in samples from Longnan. The functional genes of the bacteria present in samples indicated that a significant difference was observed in the bacterial community between samples of different origin. We also found that microbial interactions between bacterial and fungal communities in the samples were very complex. This study provides previously unknown information regarding the impact of the environment on microbial communities in douchi and lays a foundation for further investigations into food ecology in bacterial douchi.

Copyright © 2018 Zhang et al. This is an open access article distributed under the terms of the Creative Commons Attribution License, which permits unrestricted use, distribution, and reproduction in any medium, provided the original author and source are credited.

PMID

29570735 [<http://www.ncbi.nlm.nih.gov/pubmed/?term=29570735>]

Institution

(Zhang, Luo, Zhu, Ma, Cao, Wen, Zhang, He) College of Food Science and Engineering, Gansu Agricultural University, Lanzhou, China (Yang) College of Science, Gansu Agricultural University, Lanzhou, China

Publisher

Public Library of Science (E-mail: plos@plos.org)

Emtree Heading

Aerococcus; article; Bacillus; Candida; Carnobacterium; case report; *China; clinical article; ecology; Enterococcus; fungal community; genus; Geobacillus; geography; high throughput sequencing; human; microbial community; *microbial diversity; nonhuman; organismal interaction; phylum; Pichia; Proteus; Rhodosporidium; species richness; Staphylococcus; Yarrowia.

Other Index Terms

Aerococcus; article; Bacillus; Candida; Carnobacterium; case report; *China; clinical article; ecology; Enterococcus; fungal community; genus; Geobacillus; geography; high throughput sequencing; human; microbial community; *microbial diversity; nonhuman; organismal interaction; phylum; Pichia; Proteus; Rhodosporidium; species richness; Staphylococcus; Yarrowia.

Link to the Ovid Full Text or citation:

[Click here for full text options](https://libaccess.mcmaster.ca/login?url=http://ovidsp.ovid.com/ovidweb.cgi?T=JS&CSC=Y&NEWS=N&PAGE=fulltext&D=emed19&AN=621359412)

Link to the External Link Resolver:

[SFX](http://sfx.scholarsportal.info/mcmaster?sid=OVID:embase&id=pmid:29570735&id=doi:10.1371%2Fjournal.pone.0194876&issn=1932-6203&isbn=&volume=13&issue=3&spage=&pages=&date=2018&title=PLoS+ONE&atitle=Microbial+diversity+in+two+traditional+bacterial+douchi+from+Gansu+province+in+northwest+China+using+Illumina+sequencing&aulast=Zhang&pid=<author>Zhang+W.%3BLuo+Q.%3BZhu+Y.%3BMa+J.%3BCao+L.%3BYang+M.%3BWen+P.%3BZhang+Z.%3BHe+X.<%2Fauthor><AN>621359412<%2FAN><DT>Article<%2FDT>)

37.

Microbial spoilage investigation of thawed common cuttlefish (Sepia officinalis) stored at 2degreeC using next generation sequencing and volatilome analysis.

Parlapani F.F., Michailidou S., Anagnostopoulos D.A., Sakellariou A.K., Pasentsis K., Psomopoulos F., Argiriou A., Haroutounian S.A., Boziaris I.S.

Food microbiology. 76 (pp 518-525), 2018. Date of Publication: 01 Dec 2018.

AN: 624805358

Cephalopods are highly appreciated with increasing demand seafood, but are also very perishable and deteriorate fast mainly due to microbiological spoilage. For this reason exploration of bacterial communities through 16S Next Generation Sequencing (NGS) and Volatile Organic Compounds (VOCs) analysis was performed. Furthermore, sensory evaluation, classical microbiological analysis, Total Volatile Base-Nitrogen/TVB-N and Trimethylamine-Nitrogen/TMA-N determination were also carried out. Shelf-life of thawed cuttlefish (Sepia officinalis) stored at 2degreeC determined by sensory evaluation was 4 days. Aerobic Plate Counts (APC) reached the levels of 6.6 log cfu/g. The initial and final population of all spoilage microorganisms enumerated with selective media was under detectable levels with the exception of Pseudomonas. Based on 16S NGS analysis, Psychrobacter were the dominants among others, e.g. Pseudomonas, Shewanella, Comamonas, Carnobacterium, Vagococcus, of the initial microbiota. Psychrobacter was also the dominant microorganisms of the spoiled cuttlefish. TVB-N and TMA-N increased considerably only at the late stages of storage. A plethora of VOCs were produced and some exhibited an increasing profile throughout storage, making them promising molecules as freshness indicators in contrast to TVB-N and TMA-N. The application of next generation sequencing revealed the microbiota that escapes the classic microbiological methodologies, showing that other microorganisms different from those determined on selective culture media might be the main cause of microbiological spoilage.

Copyright © 2018. Published by Elsevier Ltd.

PMID

30166182 [<http://www.ncbi.nlm.nih.gov/pubmed/?term=30166182>]

Institution

(Parlapani, Anagnostopoulos, Sakellariou, Boziaris) Dept. of Ichthyology and Aquatic Environment, School of Agricultural Sciences, University of Thessaly, Lab. Marketing and Technology of Aquatic Products and FoodsFitokou StreetN. Ionia, Volos 38446, Greece (Michailidou, Pasentsis, Psomopoulos, Argiriou) Institute of Applied Biosciences, Centre for Research and Technology Hellas (CERTH), Thessaloniki 57001, Greece

(Haroutounian) Department of Animal Science and Aquaculture, Agricultural University of Athens, Iera Odos 75, Athens 118 55, Greece

Emtree Heading

analysis; animal; bacterium; *chemistry; classification; cold; cuttlefish; food contamination; food storage; genetics; *growth, development and aging; high throughput sequencing; human; *isolation and purification; metabolism; *microbiology; microflora; odor; sea food; taste; volatile organic compound.

Drug Index Terms

volatile organic compound.

Other Index Terms

analysis; animal; bacterium; *chemistry; classification; cold; cuttlefish; food contamination; food storage; genetics; *growth, development and aging; high throughput sequencing; human; *isolation and purification; metabolism; *microbiology; microflora; odor; sea food; taste.

Link to the Ovid Full Text or citation:

[Click here for full text options](https://libaccess.mcmaster.ca/login?url=http://ovidsp.ovid.com/ovidweb.cgi?T=JS&CSC=Y&NEWS=N&PAGE=fulltext&D=emed19&AN=624805358)

Link to the External Link Resolver:

[SFX](http://sfx.scholarsportal.info/mcmaster?sid=OVID:embase&id=pmid:30166182&id=doi:10.1016%2Fj.fm.2018.08.004&issn=1095-9998&isbn=&volume=76&issue=&spage=518&pages=518-525&date=2018&title=Food+microbiology&atitle=Microbial+spoilage+investigation+of+thawed+common+cuttlefish+(Sepia+officinalis)+stored+at+2degreeC+using+next+generation+sequencing+and+volatilome+analysis&aulast=Parlapani&pid=<author>Parlapani+F.F.%3BMichailidou+S.%3BAnagnostopoulos+D.A.%3BSakellariou+A.K.%3BPasentsis+K.%3BPsomopoulos+F.%3BArgiriou+A.%3BHaroutounian+S.A.%3BBoziaris+I.S.<%2Fauthor><AN>624805358<%2FAN><DT>Article<%2FDT>)

38.

Bacterial DNA detected on pathologically changed heart valves using 16S rRNA gene amplification.

Chalupova M., Skalova A., Hajek T., Geigerova L., Kralova D., Liska P., Hecova H., Molacek J., Hrabak J.

Folia microbiologica. 63 (6) (pp 707-711), 2018. Date of Publication: 01 Nov 2018.

AN: 624688796

Nowadays, dental diseases are one of the most common illnesses in the world. Some of them can lead to translocation of oral bacteria to the bloodstream causing intermittent bacteraemia. Therefore, a potential association between oral infection and cardiovascular diseases has been discussed in recent years as a result of adhesion of oral microbes to the heart valves. The aim of this study was to detect oral bacteria on pathologically changed heart valves not caused by infective endocarditis. In the study, patients with pathologically changed heart valves were involved. Samples of heart valves removed during heart valve replacement surgery were cut into two parts. One aliquot was cultivated aerobically and anaerobically. Bacterial DNA was extracted using Ultra-Deep Microbiome Prep (Molzym GmbH, Bremen, Germany) followed by a 16S rRNA gene PCR amplification using Mastermix 16S Complete kit (Molzym GmbH, Bremen, Germany). Positive PCR products were sequenced and the sequences were analyzed using BLAST database ( <http://www.ncbi.nlm.nih/BLAST> ). During the study period, 41 samples were processed. Bacterial DNA of the following bacteria was detected in 21 samples: Cutibacterium acnes (formerly Propionibacterium acnes) (n=11; 52.38% of patients with positive bacterial DNA detection), Staphylococcus sp. (n=9; 42.86%), Streptococcus sp. (n=1; 4.76%), Streptococcus sanguinis (n=4; 19.05%), Streptococcus oralis (n=1; 4.76%), Carnobacterium sp. (n=1; 4.76%), Bacillus sp. (n=2; 9.52%), and Bergeyella sp. (n=1; 4.76%). In nine samples, multiple bacteria were found. Our results showed significant appearance of bacteria on pathologically changed heart valves in patients with no symptoms of infective endocarditis.

PMID

29786766 [<http://www.ncbi.nlm.nih.gov/pubmed/?term=29786766>]

Author NameID

Chalupova, Miroslava; ORCID: <http://orcid.org/0000-0002-7618-3582>

Institution

(Chalupova, Hecova) Department of Stomatology, Faculty of Medicine and University Hospital in Pilsen, Charles University, alej Svobody 80 304 60, Czechia (Chalupova, Skalova, Kralova, Hrabak) Biomedical Center, Faculty of Medicine in Pilsen, Charles University, Plzen, Czechia

(Skalova, Geigerova, Kralova, Hrabak) Department of Microbiology, Faculty of Medicine and University Hospital in Pilsen, Charles University, Plzen, Czechia

(Hajek) Department of Cardiac Surgery, University Hospital in Pilsen, Charles University, Plzen, Czechia

(Liska) Czech Statistical Office, Prague, Czechia

(Molacek) Department of Surgery, Faculty of Medicine and University Hospital in Pilsen, Charles University, Plzen, Czechia

Publisher

NLM (Medline)

Emtree Heading

aged; bacterial endocarditis/th [Therapy]; bacterium; classification; DNA sequence; female; *gene amplification; genetics; heart valve; heart valve replacement; human; male; *microbiology; middle aged; mortality; pathology; polymerase chain reaction; procedures; very elderly; *bacterial DNA; *RNA 16S.

Drug Index Terms

*bacterial DNA; *RNA 16S.

Other Index Terms

aged; bacterial endocarditis / therapy; bacterium; classification; DNA sequence; female; *gene amplification; genetics; heart valve; heart valve replacement; human; male; *microbiology; middle aged; mortality; pathology; polymerase chain reaction; procedures; very elderly.

Link to the Ovid Full Text or citation:

[Click here for full text options](https://libaccess.mcmaster.ca/login?url=http://ovidsp.ovid.com/ovidweb.cgi?T=JS&CSC=Y&NEWS=N&PAGE=fulltext&D=emed19&AN=624688796)

Link to the External Link Resolver:

[SFX](http://sfx.scholarsportal.info/mcmaster?sid=OVID:embase&id=pmid:29786766&id=doi:10.1007%2Fs12223-018-0611-6&issn=1874-9356&isbn=&volume=63&issue=6&spage=707&pages=707-711&date=2018&title=Folia+microbiologica&atitle=Bacterial+DNA+detected+on+pathologically+changed+heart+valves+using+16S+rRNA+gene+amplification&aulast=Chalupova&pid=<author>Chalupova+M.%3BSkalova+A.%3BHajek+T.%3BGeigerova+L.%3BKralova+D.%3BLiska+P.%3BHecova+H.%3BMolacek+J.%3BHrabak+J.<%2Fauthor><AN>624688796<%2FAN><DT>Article<%2FDT>)

39.

Draft genome sequences of nine new Carnobacterium maltaromaticum strains isolated from diseased sharks.

Martinez-Steele L., Lowe C.G., Okihiro M.S., Berlemont R.

Genome Announcements. 6 (18) (no pagination), 2018. Article Number: e00354-18. Date of Publication: 01 May 2018.

AN: 622002927

Here, we report the draft genome sequences of 9 strains of Carnobacterium maltaromaticum (SK_LD1 to SK_LD3 and SK_AV1 to SK_AV6), a member of the Carnobacteriaceae family (phylum Firmicutes). These strains were isolated from the brain and the inner ear of three diseased thresher sharks and two diseased salmon sharks. The genome assembly resulted in an average of 3,306,205.9 +/- 29,143.9 bp and 3,085 +/- 32.67 coding DNA sequences (CDS).

Copyright © 2018 Martinez-Steele et al.

Institution

(Martinez-Steele, Lowe, Berlemont) Department of Biological Sciences, California State University, Long Beach, CA, United States (Okihiro) California Department of Fish and Wildlife, San Diego, CA, United States

Publisher

American Society for Microbiology (E-mail: Journals@asmusa.org)

Emtree Heading

article; brain; *Carnobacterium maltaromaticum; clinical article; DNA sequence; *genome; human; inner ear; nonhuman; phylum; *shark.

Other Index Terms

article; brain; *Carnobacterium maltaromaticum; clinical article; DNA sequence; *genome; human; inner ear; nonhuman; phylum; *shark.

Link to the Ovid Full Text or citation:

[Click here for full text options](https://libaccess.mcmaster.ca/login?url=http://ovidsp.ovid.com/ovidweb.cgi?T=JS&CSC=Y&NEWS=N&PAGE=fulltext&D=emed19&AN=622002927)

Link to the External Link Resolver:

[SFX](http://sfx.scholarsportal.info/mcmaster?sid=OVID:embase&id=pmid:&id=doi:10.1128%2FgenomeA.00354-18&issn=2169-8287&isbn=&volume=6&issue=18&spage=&pages=&date=2018&title=Genome+Announcements&atitle=Draft+genome+sequences+of+nine+new+Carnobacterium+maltaromaticum+strains+isolated+from+diseased+sharks&aulast=Martinez-Steele&pid=<author>Martinez-Steele+L.%3BLowe+C.G.%3BOkihiro+M.S.%3BBerlemont+R.<%2Fauthor><AN>622002927<%2FAN><DT>Article<%2FDT>)

40.

Multi-Cohort Analysis of Colorectal Cancer Metagenome Identified Altered Bacteria Across Populations and Universal Bacterial Markers.

Dai Z., Coker O.O., Nakatsu G., Wu W.K., Zhao L., Chen Z., Chan F.K., Kristiansen K., Sung J.J., Wong S.H., Yu J.

Gastroenterology. Conference: DDW 2018 - Digestive Disease Week. United States. 154 (6 Supplement 1) (pp S-1047-S-1048), 2018. Date of Publication: May 2018.

AN: 2002433174

Background & Aims Alterations of the gut microbiota are associated with colorectal cancer (CRC) in different populations and several bacterial species were identified to contribute to tumorigenesis. The potential use of gut microbes as non-invasive markers for early diagnosis of CRC has also been explored. However, the highly dynamic nature of the gut microbiome coupled with technical heterogeneity result in inconsistent results across different studies. In this regard, we performed a meta-analysis on metagenomic datasets from multiple cohorts of CRC fecal samples. Our study aimed to unravel alterations in the gut microbiota that are universal across diverse populations. Methods We analyzed a total of 255 CRC and 271 control samples from Chinese, Austrian, American and German-French cohorts that were sequenced on the same sequencing platform with comparable sequencing depth. We processed the raw shotgun metagenome sequencing data sets with Kraken to obtain the bacteria composition on species level. The meta-analysis was then performed using a non-parametric approach, RankSum, to detect bacterial abundance shift associated with CRC. We used SparCC to infer the correlations among bacteria species and HUMAnN2 to analyze the functional gene groups and metabolic pathways involved. Results Seven bacteria species (Bacteroides fragilis, Fusobacterium nucleatum, Porphyromonas asaccharolytica, Parvimonas micra, Prevotella intermedia, Alistipes finegoldii and Thermanaerovibrio acidaminovorans) were identified to be significantly enriched in CRC across different patient populations (p < 0.01), indicating that they might be useful universal biomarkers for CRC. These seven enriched bacterial markers classified CRC from control with an area under the receiver-operating characteristics curve (AUC) of 0.82. We also identified 62 CRC-depleted bacteria species including five probiotics, Clostridium butyricum, Streptococcus salivarius, S. thermophiles, Carnobacterium maltaromaticum and Lactobacillus gallinarum, which may play protective roles against colorectal carcinogenesis. Abundance correlation analysis demonstrated that CRC-enriched and -depleted bacteria species respectively formed their own mutualistic networks, in which the latter was disjointed in CRC. The CRC-enriched bacteria were found to be correlated with lipopolysaccharide and energy biosynthetic pathways. Conclusion Our study identified a combination of bacterial species markers that is robustly enriched in CRC across populations, indicating their potential use as universal, non-invasive diagnostic markers in CRC. We also elucidated the ecological networks associated with CRC and functional capacities of CRC-associated microbiota.

Copyright © 2018 AGA Institute

Publisher

W.B. Saunders

Emtree Heading

adult; Alistipes; area under the curve; Bacteroides fragilis; biosynthesis; cancer patient; Carnobacterium maltaromaticum; Clostridium butyricum; *cohort analysis; colon carcinogenesis; *colorectal cancer; controlled study; correlation analysis; diagnosis; feces; female; functional status; Fusobacterium nucleatum; human; human tissue; intestine flora; male; meta analysis; *metagenome; metagenomics; nonhuman; Parvimonas micra; Porphyromonas asaccharolytica; Prevotella intermedia; receiver operating characteristic; Streptococcus salivarius; thermophile; biological marker; lipopolysaccharide; probiotic agent; conference abstract.

Candidate Terms

conference abstract [other term].

Drug Index Terms

biological marker; lipopolysaccharide; probiotic agent.

Other Index Terms

adult; Alistipes; area under the curve; Bacteroides fragilis; biosynthesis; cancer patient; Carnobacterium maltaromaticum; Clostridium butyricum; *cohort analysis; colon carcinogenesis; *colorectal cancer; controlled study; correlation analysis; diagnosis; feces; female; functional status; Fusobacterium nucleatum; human; human tissue; intestine flora; male; meta analysis; *metagenome; metagenomics; nonhuman; Parvimonas micra; Porphyromonas asaccharolytica; Prevotella intermedia; receiver operating characteristic; Streptococcus salivarius; thermophile.

Link to the Ovid Full Text or citation:

[Click here for full text options](https://libaccess.mcmaster.ca/login?url=http://ovidsp.ovid.com/ovidweb.cgi?T=JS&CSC=Y&NEWS=N&PAGE=fulltext&D=emed19&AN=2002433174)

Link to the External Link Resolver:

[SFX](http://sfx.scholarsportal.info/mcmaster?sid=OVID:embase&id=pmid:&id=doi:10.1016%2FS0016-5085%252818%252933511-X&issn=0016-5085&isbn=&volume=154&issue=6+Supplement+1&spage=S&pages=S-1047-S-1048&date=2018&title=Gastroenterology&atitle=Multi-Cohort+Analysis+of+Colorectal+Cancer+Metagenome+Identified+Altered+Bacteria+Across+Populations+and+Universal+Bacterial+Markers&aulast=Dai&pid=<author>Dai+Z.%3BCoker+O.O.%3BNakatsu+G.%3BWu+W.K.%3BZhao+L.%3BChen+Z.%3BChan+F.K.%3BKristiansen+K.%3BSung+J.J.%3BWong+S.H.%3BYu+J.<%2Fauthor><AN>2002433174<%2FAN><DT>Conference+Abstract<%2FDT>)

41.

Lactic acid bacteria in traditional fermented Asian foods.

Azam M., Mohsin M., Ijaz H., Tulain U.R., Ashraf M.A., Fayyaz A., Ul Abadeen Z., Kamran Q.

Pakistan Journal of Pharmaceutical Sciences. 30 (5) (pp 1803-1814), 2017. Date of Publication: September 2017.

AN: 617762199

Lactic acid bacteria play vital roles in various fermented foods in Asia. This paper reviews many types of the world's lactic acid fermented foods and discusses the beneficial effects of lactic acid fermentation of food. The lactic acid bacteria associated with foods now include species of the genera Carnobacterium, Enterococcus, Lactobacillus, Lactococcus, Leuconostoc, Oenococcus, Pediococcus, Streptococcus, Tetragenococcus, Vagococcus and Weissella. Lactic acid bacteria (LAB) are involved in many fermentation processes of Asian traditional foods, demonstrating their profound effects on improving food quality and food safety. During the past few decades' interest has arisen in the use of the varied antagonistic activities of LAB to extent the shelf-life of protein-rich products such as meats and fish. This review article outlines the main types of LAB fermentation as well as their typical fermented foods such as idli, kishk, sauerkraut, koumiss, Suan-tsai, stinky tofu, Chinese sausage and kefir. The roles of LAB and the reasons for their common presence are also discussed.

PMID

29084705 [<http://www.ncbi.nlm.nih.gov/pubmed/?term=29084705>]

Institution

(Azam, Mohsin, Ashraf) Institute of Microbiology, University of Agriculture, Faisalabad, Pakistan (Ijaz, Tulain) Faculty of Pharmacy, University of Sargodha, Punjab, Pakistan

(Fayyaz, Ul Abadeen) Department of Pathology, University of Agriculture, Faisalabad, Pakistan

(Kamran) Institute of Pharmacy, Physiology and Pharmacology, University of Agriculture, Faisalabad, Pakistan

Publisher

Pakistan Journal of Pharmaceutical Sciences

Emtree Heading

biotechnology; Carnobacterium; dietary intake; Enterococcus; fermentation; *fermented product; food quality; food safety; human; *koumiss; *lactic acid bacterium; Lactobacillus; Lactococcus; Leuconostoc; nonhuman; Oenococcus; Pediococcus; review; shelf life; Streptococcus; Tetragenococcus; Weissella; *idli; *kishk; *sauerkraut; *stinky tofu; *Suan tsai; Vagococcus.

Candidate Terms

*idli [other term]; *kishk [other term]; *sauerkraut [other term]; *stinky tofu [other term]; *Suan tsai [other term]; Vagococcus [other term].

Other Index Terms

biotechnology; Carnobacterium; dietary intake; Enterococcus; fermentation; *fermented product; food quality; food safety; human; *koumiss; *lactic acid bacterium; Lactobacillus; Lactococcus; Leuconostoc; nonhuman; Oenococcus; Pediococcus; Review; shelf life; Streptococcus; Tetragenococcus; Weissella.

Link to the Ovid Full Text or citation:

[Click here for full text options](https://libaccess.mcmaster.ca/login?url=http://ovidsp.ovid.com/ovidweb.cgi?T=JS&CSC=Y&NEWS=N&PAGE=fulltext&D=emed18&AN=617762199)

Link to the External Link Resolver:

[SFX](http://sfx.scholarsportal.info/mcmaster?sid=OVID:embase&id=pmid:29084705&id=doi:&issn=1011-601X&isbn=&volume=30&issue=5&spage=1803&pages=1803-1814&date=2017&title=Pakistan+Journal+of+Pharmaceutical+Sciences&atitle=Lactic+acid+bacteria+in+traditional+fermented+Asian+foods&aulast=Azam&pid=<author>Azam+M.%3BMohsin+M.%3BIjaz+H.%3BTulain+U.R.%3BAshraf+M.A.%3BFayyaz+A.%3BUl+Abadeen+Z.%3BKamran+Q.<%2Fauthor><AN>617762199<%2FAN><DT>Review<%2FDT>)

42.

Influence of potassium-competitive acid blocker on the gut microbiome of Helicobacter pylori -negative healthy individuals.

Otsuka T., Sugimoto M., Inoue R., Ohno M., Ban H., Nishida A., Inatomi O., Takahashi S., Naito Y., Andoh A.

Gut. 66 (9) (pp 1723-1725), 2017. Date of Publication: 01 Sep 2017.

AN: 613865660

PMID

27965281 [<http://www.ncbi.nlm.nih.gov/pubmed/?term=27965281>]

Institution

(Otsuka, Ohno, Nishida, Inatomi, Andoh) Department of Medicine, Shiga University of Medical Science, Seta Tsukinowa, Otsu 520-2192, Japan (Sugimoto, Ban) Division of Gastrointestinal Endoscopy, Shiga University of Medical Science, Otsu, Japan

(Inoue) Laboratory of Animal Science, Department of Agriculture and Life Science, Kyoto Prefectural University, Kyoto, Japan

(Takahashi) TechnoSuruga Laboratory, Shizuoka, Japan

(Naito) Department of Molecular Gastroenterology and Hepatology, Kyoto Prefectural University of Medicine, Kyoto, Japan

Publisher

BMJ Publishing Group (E-mail: subscriptions@bmjgroup.com)

Emtree Heading

Actinomyces; article; Bacillus; Bacteroides; Bifidobacterium; Blautia; Carnobacterium; Collinsella; community structure; controlled study; Eggerthella; feces analysis; genus; Granulicatella; *Helicobacter pylori; human; *intestine flora; microbial diversity; normal human; Parabacteroides; Prevotella; priority journal; RNA gene; Rothia; Streptococcus; *lansoprazole/cm [Drug Comparison]; RNA 16S/ec [Endogenous Compound]; *vonoprazan/cm [Drug Comparison]; Coprococcus; Holdemania; Oribacterium.

Candidate Terms

Coprococcus [other term]; Holdemania [other term]; Oribacterium [other term].

Drug Index Terms

*lansoprazole / *drug comparison; RNA 16S / endogenous compound; *vonoprazan / *drug comparison.

Other Index Terms

Actinomyces; Article; Bacillus; Bacteroides; Bifidobacterium; Blautia; Carnobacterium; Collinsella; community structure; controlled study; Eggerthella; feces analysis; genus; Granulicatella; *Helicobacter pylori; human; *intestine flora; microbial diversity; normal human; Parabacteroides; Prevotella; priority journal; RNA gene; Rothia; Streptococcus.

Link to the Ovid Full Text or citation:

[Click here for full text options](https://libaccess.mcmaster.ca/login?url=http://ovidsp.ovid.com/ovidweb.cgi?T=JS&CSC=Y&NEWS=N&PAGE=fulltext&D=emed18&AN=613865660)

Link to the External Link Resolver:

[SFX](http://sfx.scholarsportal.info/mcmaster?sid=OVID:embase&id=pmid:27965281&id=doi:10.1136%2Fgutjnl-2016-313312&issn=0017-5749&isbn=&volume=66&issue=9&spage=1723&pages=1723-1725&date=2017&title=Gut&atitle=Influence+of+potassium-competitive+acid+blocker+on+the+gut+microbiome+of+Helicobacter+pylori+-negative+healthy+individuals&aulast=Otsuka&pid=<author>Otsuka+T.%3BSugimoto+M.%3BInoue+R.%3BOhno+M.%3BBan+H.%3BNishida+A.%3BInatomi+O.%3BTakahashi+S.%3BNaito+Y.%3BAndoh+A.<%2Fauthor><AN>613865660<%2FAN><DT>Article<%2FDT>)

43.

Midgut bacteria in deltamethrin-resistant, deltamethrin-susceptible, and field-caught populations of Plutella xylostella, and phenomics of the predominant midgut bacterium Enterococcus mundtii.

Li W., Jin D., Shi C., Li F.

Scientific reports. 7 (1) (pp 1947), 2017. Date of Publication: 16 May 2017.

AN: 624889862

Gut bacteria play a significant role in host insect. This study evaluated detail difference of midgut bacteria in deltamethrin-resistant, deltamethrin-susceptible and field-caught populations of diamondback moth, and studied phenomics of the predominant midgut bacterium Enterococcus mundtii. Cultivable bacteria revealed that E. mundtii and Carnobacterium maltaromaticum dominated the bacterial populations from deltamethrin-resistant and deltamethrin-susceptible larval midguts, whereas E. mundtii was predominant in field-caught population. Illumina sequencing analysis indicated that 97% of the midgut bacteria were from the phyla Firmicutes, Proteobacteria and Cyanobacteria. Both resistant and susceptible populations had more Enterococcus and Carnobacterium. Enterococcus, Carnobacterium, Bacillus, and Pseudomonas were predominant in the field-caught population. A phenomics analysis revealed that E. mundtii was able to metabolize 25.26% of the tested carbon sources, 100% of the nitrogen sources, 100% of the phosphorus sources and 97.14% of the sulfur sources, had a wide range of osmolytes and pH conditions, and showed active deaminase activity but no decarboxylase activity. This is the first report regarding different populations of DBM midgut bacteria analyzed using both high-throughput DNA sequencing and cultivation methods, and also first report concerning the phenomics of E. mundtii. The phenomics of E. mundtii provide a basis for the future study of gut bacteria functions.

PMID

28512315 [<http://www.ncbi.nlm.nih.gov/pubmed/?term=28512315>]

Institution

(Li, Jin) Institute of Entomology, Guizhou University, Guiyang 550025, China (Li, Li) Guizhou Institute of Plant Protection, Guizhou Academy of Agricultural Sciences, Guiyang 550006, China

(Shi) College of Agriculture, Yangtze University, Jingzhou 434025, China

Publisher

NLM (Medline)

Emtree Heading

article; carbon source; Carnobacterium maltaromaticum; controlled study; cyanobacterium; DNA sequence; drug resistance; *Enterococcus; enzyme activity; Firmicutes; human; intestine flora; *midgut; nonhuman; *Plutella xylostella; Pseudomonas; susceptible population; carboxylyase; deaminase; *deltamethrin; endogenous compound; nitrogen; phosphorus; sulfur.

Drug Index Terms

carboxylyase [m]; deaminase [m]; *deltamethrin [m]; endogenous compound [m]; nitrogen [m]; phosphorus [m]; sulfur [m].

Other Index Terms

article [m]; carbon source [m]; Carnobacterium maltaromaticum [m]; controlled study [m]; cyanobacterium [m]; DNA sequence [m]; drug resistance [m]; *Enterococcus [m]; enzyme activity [m]; Firmicutes [m]; human [m]; intestine flora [m]; *midgut [m]; nonhuman [m]; *Plutella xylostella [m]; Pseudomonas [m]; susceptible population [m].

Link to the Ovid Full Text or citation:

[Click here for full text options](https://libaccess.mcmaster.ca/login?url=http://ovidsp.ovid.com/ovidweb.cgi?T=JS&CSC=Y&NEWS=N&PAGE=fulltext&D=emed18&AN=624889862)

Link to the External Link Resolver:

[SFX](http://sfx.scholarsportal.info/mcmaster?sid=OVID:embase&id=pmid:28512315&id=doi:10.1038%2Fs41598-017-02138-9&issn=2045-2322&isbn=&volume=7&issue=1&spage=1947&pages=1947&date=2017&title=Scientific+reports&atitle=Midgut+bacteria+in+deltamethrin-resistant%2C+deltamethrin-susceptible%2C+and+field-caught+populations+of+Plutella+xylostella%2C+and+phenomics+of+the+predominant+midgut+bacterium+Enterococcus+mundtii&aulast=Li&pid=<author>Li+W.%3BJin+D.%3BShi+C.%3BLi+F.<%2Fauthor><AN>624889862<%2FAN><DT>Article<%2FDT>)

44.

Alterations of gut microbiome in rheumatoid arthritis.

Wu X., Liu J., Xiao L., Lu A., Zhang G.

Osteoarthritis and Cartilage. Conference: 2017 Osteoarthritis Research Society International, OARSI World Congress. United States. 25 (Supplement 1) (pp S287-S288), 2017. Date of Publication: April 2017.

AN: 620927151

Purpose: Rheumatoid arthritis (RA) is a chronic inflammatory joint disease, which can cause cartilage and bone damage as well as disability. Recent studies have indicated that the pathogenesis of RA requires the interaction between genetic and environmental factors, such as gut microbiome. However, current evidence for the alterations of gut microbiome in RA is still limited. Thus, our study aimed to analyze the fecal samples from a cohort patient with RA and to identify the associated alterations in gut microbiome when compared to healthy controls.

Method(s): Fecal samples were obtained from treatment-naIve RA patients (n=50) and healthy controls (n=50). High-quality 16S rDNA V3-V4 sequences were extracted from fecal samples and further sequenced on a 454 Genome Sequencer platform for bioinformatics analysis, resulting in operational taxonomic units (OTUs) related to gut microbiota and phylogenetic tree. Clinical parameters of RA were correlated with specific microbial taxa by Correlation analysis.

Result(s): A total of 121227 OTUs was clustered at 97 % sequence similarity and assigned taxonomic lineages by comparison with the Ribosomal Database Project database. The OTUs were classified into 35 phylums, 73 classes, 143 orders, 280 families, and 1165 genus. Intriguingly, our data revealed a lower alpha diversity, including richness, evenness and Shannon Index, as well as a lower Firmicutes / Bacteroidetes ratio in the gut microbiome of RA patients when compared with the healthy controls (Figure 1a, b). Moreover, at genus level, our taxonomic analysis identified 288 differentially abundant taxa (P< 0.05) (Figure 2a), among which 6 taxa, including Bacteroides, Parabacteroides, Paraprevotella, Porphyromonadaceae, Phascolarctobacterium and Carnobacterium, were significantly enriched in the gut microbiome of RA patients (Figure 2b, c). Conversely, a series of butyrate-producing taxa such as Faecalibacterium, Roseburia, Subdoligranulum, Ruminococcus and Pseudobutyrivibrio were depleted in RA patients but enriched in healthy controls (Figure 2b, c). Furthermore, we found a negative correlation (P<0.05) between the abundance of butyrate-producing Roseburia and the clinical parameters of disease status and activity such as erythrocyte sedimentation rate and the blood level of rheumatoid factors (IgM) (Figure 3).

Conclusion(s): In the present study, we demonstrated that the composition of the gut microbiome in RA patients differed from that in healthy individuals. Our results revealed a decreased microbial diversity accompanied by altered microbial abundance in the gut microbiome of RA patients, which could be characterized as 'dysbiosis' and associated with the disease status and activity. The identified alterations in the gut microbiome might provide an updated overview for postulating molecular understandings and therapeutic strategy in RA. (Figure Presented) .

Institution

(Wu, Liu, Lu, Zhang) Inst. for Advancing Translational Med. in Bone and Joint Diseases, Hong Kong, Hong Kong (Xiao) Inst. of Arthritis Res., Shanghai Academy of Chinese Med. Sci., Shanghai, China

Publisher

W.B. Saunders Ltd

Emtree Heading

adult; Bacteroides; bioinformatics; Carnobacterium; clinical article; cohort analysis; controlled study; correlation analysis; disease course; erythrocyte sedimentation rate; Faecalibacterium; female; genus; human; human tissue; *intestine flora; male; microbial diversity; nonhuman; ordo; Parabacteroides; phylogenetic tree; phylum; protein blood level; *rheumatoid arthritis; Ruminococcus; butyric acid; DNA 16S; endogenous compound; immunoglobulin M; rheumatoid factor; conference abstract.

Candidate Terms

conference abstract [other term].

Drug Index Terms

butyric acid; DNA 16S; endogenous compound; immunoglobulin M; rheumatoid factor.

Other Index Terms

adult; Bacteroides; bioinformatics; Carnobacterium; clinical article; cohort analysis; controlled study; correlation analysis; disease course; erythrocyte sedimentation rate; Faecalibacterium; female; genus; human; human tissue; *intestine flora; male; microbial diversity; nonhuman; ordo; Parabacteroides; phylogenetic tree; phylum; protein blood level; *rheumatoid arthritis; Ruminococcus.

Link to the Ovid Full Text or citation:

[Click here for full text options](https://libaccess.mcmaster.ca/login?url=http://ovidsp.ovid.com/ovidweb.cgi?T=JS&CSC=Y&NEWS=N&PAGE=fulltext&D=emed18&AN=620927151)

Link to the External Link Resolver:

[SFX](http://sfx.scholarsportal.info/mcmaster?sid=OVID:embase&id=pmid:&id=doi:&issn=1522-9653&isbn=&volume=25&issue=Supplement+1&spage=S287&pages=S287-S288&date=2017&title=Osteoarthritis+and+Cartilage&atitle=Alterations+of+gut+microbiome+in+rheumatoid+arthritis&aulast=Wu&pid=<author>Wu+X.%3BLiu+J.%3BXiao+L.%3BLu+A.%3BZhang+G.<%2Fauthor><AN>620927151<%2FAN><DT>Conference+Abstract<%2FDT>)

45.

Alterations of gut microbiome in rheumatoid arthritis.

Wu X.H., Liu J., Lu A.P., Zhang G.

Osteoporosis International. Conference: World Congress on Osteoporosis, Osteoarthritis and Musculoskeletal Diseases, WCO-IOF-ESCEO 2017. Italy. 28 (Supplement 1) (pp S517-S518), 2017. Date of Publication: 2017.

AN: 617635203

Objectives: Rheumatoid arthritis (RA) is a chronic inflammatory joint disease, which can cause cartilage and bone damage as well as disability. Recent studies have indicated that the pathogenesis of RA requires the interaction between genetic and environmental factors, such as gut microbiome. However, current evidence for the alterations of gut microbiome in RA is still limited. Thus, our study aimed to analyze the fecal samples from a cohort patient with RA and to identify the asso-ciated alterations in gut microbiome when compared to healthy controls.

Material(s) and Method(s): Fecal samples were obtained from treatment-naive RA patients (n=50) and healthy controls (n=50). High-quality 16S rDNA V3-V4 sequences were extracted from fecal samples and further sequenced on a 454 Genome Sequencer platform for bioinformatics analysis, resulting in operational taxo-nomic units (OTUs) related to gut microbiota and phy-logenetic tree. Clinical parameters of RA were correlated with specific microbial taxa by Correlation analysis.

Result(s): A total of 121227 OTUs was clustered at 97% sequence similarity and assigned taxonomic lineages by comparison with the Ribosomal Database Project database. The OTUs were classified into 35 phylums, 73 classes, 143 orders, 280 families, and 1165 genus. Intriguingly, our data revealed a lower alpha diversity, including richness, evenness and Shannon Index, as well as a lower Firmicutes/Bacteroidetes ratio in the gut microbiome of RA patients when compared with the healthy controls. Moreover, at genus level, our taxonomic analysis identified 288 differentially abundant taxa (P<0. 05), among which 6 taxa, including Bacteroides, Parabacteroides, Paraprevotella, Porphyromonadaceae, Phascolarctobacterium and Carnobacterium, were significantly enriched in the gut microbiome of RA patients. Conversely, a series of butyrate-producing taxa such as Faecalibacterium, Roseburia, Subdoligranulum, Ruminococcus and Pseudobutyrivibrio were depleted in RA patients but enriched in healthy controls. Furthermore, we found a negative correlation (P<0. 05) between the abundance of butyrate-producing Roseburia and the clinical parameters of disease status and activity such as erythrocyte sedimentation rate and the blood level of rheumatoid factors (IgM) (Figure 1).

Conclusion(s): In the present study, we demonstrated that the composition of the gut microbiome in RA patients differed from that in healthy individuals. Our results revealed a decreased microbial diversity accompanied by altered microbial abundance in the gut microbiome of RA patients, which could be characterized as 'dysbiosis' and associated with the disease status and activity. The identified alterations in the gut microbiome might provide an updated overview for postulating molecular understandings and therapeutic strategy in RA.

Institution

(Wu, Liu, Lu, Zhang) Hong Kong Baptist University, Hong Kong, Hong Kong

Publisher

Springer London

Emtree Heading

Bacteroides; bioinformatics; blood level; Carnobacterium; clinical article; controlled study; correlation analysis; data base; disease course; erythrocyte sedimentation rate; Faecalibacterium; family; female; genus; human; human tissue; *intestine flora; male; microbial diversity; *nonhuman; normal human; Parabacteroides; phylum; *rheumatoid arthritis; Ruminococcus; butyric acid; DNA 16S; endogenous compound; immunoglobulin M; rheumatoid factor.

Drug Index Terms

butyric acid; DNA 16S; endogenous compound; immunoglobulin M; rheumatoid factor.

Other Index Terms

Bacteroides; bioinformatics; blood level; Carnobacterium; clinical article; controlled study; correlation analysis; data base; disease course; erythrocyte sedimentation rate; Faecalibacterium; family; female; genus; human; human tissue; *intestine flora; male; microbial diversity; *nonhuman; normal human; Parabacteroides; phylum; *rheumatoid arthritis; Ruminococcus.

Link to the Ovid Full Text or citation:

[Click here for full text options](https://libaccess.mcmaster.ca/login?url=http://ovidsp.ovid.com/ovidweb.cgi?T=JS&CSC=Y&NEWS=N&PAGE=fulltext&D=emed18&AN=617635203)

Link to the External Link Resolver:

[SFX](http://sfx.scholarsportal.info/mcmaster?sid=OVID:embase&id=pmid:&id=doi:10.1007%2Fs00198-017-3950-2&issn=1433-2965&isbn=&volume=28&issue=Supplement+1&spage=S517&pages=S517-S518&date=2017&title=Osteoporosis+International&atitle=Alterations+of+gut+microbiome+in+rheumatoid+arthritis&aulast=Wu&pid=<author>Wu+X.H.%3BLiu+J.%3BLu+A.P.%3BZhang+G.<%2Fauthor><AN>617635203<%2FAN><DT>Conference+Abstract<%2FDT>)

46.

Protective effect of Carnobacterium spp. against Listeria monocytogenes during host cell invasion using in vitro HT29 model.

Pilchova T., Pilet M.-F., Cappelier J.-M., Pazlarova J., Tresse O.

Frontiers in Cellular and Infection Microbiology. 6 (AUG) (no pagination), 2016. Article Number: 88. Date of Publication: 26 Aug 2016.

AN: 612505399

The pathogenesis of listeriosis results mainly from the ability of Listeria monocytogenes to attach, invade, replicate and survive within various cell types in mammalian tissues. In this work, the effect of two bacteriocin-producing Carnobacterium (C. divergens V41 and C. maltaromaticum V1) and three non-bacteriocinogenic strains: (C. divergens V41C9, C. divergens 2763, and C. maltaromaticum 2762) was investigated on the reduction of L. monocytogenes Scott A plaque-forming during human infection using the HT-29 in vitro model. All Carnobacteria tested resulted in a reduction in the epithelial cell invasion caused by L. monocytogenes Scott A. To understand better the mechanism underlying the level of L. monocytogenes infection inhibition by Carnobacteria, infection assays from various pretreatments of Carnobacteria were assessed. The results revealed the influence of bacteriocin production combined with a passive mechanism of mammalian cell monolayers protection by Carnobacteria. These initial results showing a reduction in L. monocytogenes virulence on epithelial cells by Carnobacteria would be worthwhile analyzing further as a promising probiotic tool for human health.

Copyright © 2016 Pilchova, Pilet, Cappelier, Pazlarova and Tresse.

PMID

27617232 [<http://www.ncbi.nlm.nih.gov/pubmed/?term=27617232>]

Institution

(Pilchova, Pazlarova) Department of Biochemistry and Microbiology, Faculty of Food and Biochemical Technology, University of Chemistry and Technology, Prague, Czechia (Pilchova, Pilet, Cappelier, Tresse) UMR1014 SECALIM, INRA, Oniris, Nantes, France

Publisher

Frontiers Media S.A. (E-mail: info@frontiersin.org)

Emtree Heading

antimicrobial activity; article; bacterial viability; bacterial virulence; *Carnobacterium; cell adhesion; *cell invasion; *cell protection; colony forming unit; controlled study; HT 29 cell line; human; human cell; *Listeria monocytogenes; nonhuman; plaque forming cell assay; bacteriocin/ec [Endogenous Compound]; gentamicin.

Drug Index Terms

bacteriocin / endogenous compound; gentamicin.

Other Index Terms

antimicrobial activity; Article; bacterial viability; bacterial virulence; *Carnobacterium; cell adhesion; *cell invasion; *cell protection; colony forming unit; controlled study; HT 29 cell line; human; human cell; *Listeria monocytogenes; nonhuman; plaque forming cell assay.

Link to the Ovid Full Text or citation:

[Click here for full text options](https://libaccess.mcmaster.ca/login?url=http://ovidsp.ovid.com/ovidweb.cgi?T=JS&CSC=Y&NEWS=N&PAGE=fulltext&D=emed17&AN=612505399)

Link to the External Link Resolver:

[SFX](http://sfx.scholarsportal.info/mcmaster?sid=OVID:embase&id=pmid:27617232&id=doi:10.3389%2Ffcimb.2016.00088&issn=2235-2988&isbn=&volume=6&issue=AUG&spage=&pages=&date=2016&title=Frontiers+in+Cellular+and+Infection+Microbiology&atitle=Protective+effect+of+Carnobacterium+spp.+against+Listeria+monocytogenes+during+host+cell+invasion+using+in+vitro+HT29+model&aulast=Pilchova&pid=<author>Pilchova+T.%3BPilet+M.-F.%3BCappelier+J.-M.%3BPazlarova+J.%3BTresse+O.<%2Fauthor><AN>612505399<%2FAN><DT>Article<%2FDT>)

47.

The microbiome of otitis media with effusion.

Chan C.L., Wabnitz D., Bardy J.J., Bassiouni A., Wormald P.-J., Vreugde S., Psaltis A.J.

Laryngoscope. 126 (12) (pp 2844-2851), 2016. Date of Publication: 01 Dec 2016.

AN: 611114562

Objectives/Hypothesis: The adenoid pad has been considered a reservoir for bacteria in the pathogenesis of otitis media with effusion. This study aimed to characterize the middle ear microbiota in children with otitis media with effusion and establish whether a correlation exists between the middle ear and adenoid microbiota.

Study Design: Prospective, controlled study.

Method(s): Middle ear aspirates adenoid pad swabs were collected from 23 children undergoing ventilation tube insertion. Adenoid swabs from patients without ear disease were controls. Samples were analyzed using 16S rRNA sequencing on the Illumina MiSeq platform.

Result(s): Thirty-five middle ear samples were collected. The middle ear effusion microbiota was dominated by Alloiococcus otitidis (23% mean relative abundance), Haemophilus (22%), Moraxella (5%), and Streptococcus (5%). Alloiococcus shared an inverse correlation with Haemophilus (P =.049) and was found in greater relative abundance in unilateral effusion (P =.004). The microbiota of bilateral effusions from the same patient were similar (P <.001). However, the otitis media with effusion microbiota were found to be dissimilar to that of the adenoid (P =.01), whereas the adenoid microbiota of otitis media with effusion and control patients were similar (P >.05) (permutational multivariate analysis of the variance).

Conclusion(s): Dissimilarities between the local microbiota of the adenoid and the middle ear question the theory that the adenoid pad is a significant reservoir to the middle ear in children with otitis media with effusion. A otitidis had the greatest cumulative relative abundance, particularly in unilateral effusions, and shares an inverse correlation with the relative abundance of Haemophilus.

Level of Evidence: NA Laryngoscope, 126:2844-2851, 2016.

Copyright © 2016 The American Laryngological, Rhinological and Otological Society, Inc.

PMID

27335217 [<http://www.ncbi.nlm.nih.gov/pubmed/?term=27335217>]

Institution

(Chan, Bardy, Bassiouni, Wormald, Vreugde, Psaltis) Department of Otolaryngology-Head & Neck Surgery, Adelaide University, Adelaide, SA, Australia (Wabnitz) Department of Otolaryngology-Head & Neck Surgery, The Women's and Children's Hospital, Adelaide, SA, Australia

Publisher

John Wiley and Sons Inc. (P.O.Box 18667, Newark NJ 07191-8667, United States)

Emtree Heading

adenoid; aerobe; anaerobe; article; aspiration; Carnobacterium; child; clinical article; controlled study; Corynebacterium; Enterobacteriaceae; female; Fusobacteria; Fusobacterium; Gemella; Gram positive bacterium; Haemophilus; human; male; *microbiome; microflora; middle ear; Moraxella; Neisseria; pathogenesis; Porphyromonas; Prevotella; priority journal; prospective study; Pseudomonas; RNA sequence; *secretory otitis media/et [Etiology]; Staphylococcus; Streptococcus; Veillonella; RNA 16S; Alloiococcus otitidis; Caulobacteria; Gemellaceae; Leptotrichiaceae; Paraprevotella.

Candidate Terms

Alloiococcus otitidis [other term]; Caulobacteria [other term]; Gemellaceae [other term]; Leptotrichiaceae [other term]; Paraprevotella [other term].

Drug Index Terms

RNA 16S.

Other Index Terms

adenoid; aerobe; anaerobe; Article; aspiration; Carnobacterium; child; clinical article; controlled study; Corynebacterium; Enterobacteriaceae; female; Fusobacteria; Fusobacterium; Gemella; Gram positive bacterium; Haemophilus; human; male; *microbiome; microflora; middle ear; Moraxella; Neisseria; pathogenesis; Porphyromonas; Prevotella; priority journal; prospective study; Pseudomonas; RNA sequence; *secretory otitis media / *etiology; Staphylococcus; Streptococcus; Veillonella.

Link to the Ovid Full Text or citation:

[Click here for full text options](https://libaccess.mcmaster.ca/login?url=http://ovidsp.ovid.com/ovidweb.cgi?T=JS&CSC=Y&NEWS=N&PAGE=fulltext&D=emed17&AN=611114562)

Link to the External Link Resolver:

[SFX](http://sfx.scholarsportal.info/mcmaster?sid=OVID:embase&id=pmid:27335217&id=doi:10.1002%2Flary.26128&issn=0023-852X&isbn=&volume=126&issue=12&spage=2844&pages=2844-2851&date=2016&title=Laryngoscope&atitle=The+microbiome+of+otitis+media+with+effusion&aulast=Chan&pid=<author>Chan+C.L.%3BWabnitz+D.%3BBardy+J.J.%3BBassiouni+A.%3BWormald+P.-J.%3BVreugde+S.%3BPsaltis+A.J.<%2Fauthor><AN>611114562<%2FAN><DT>Article<%2FDT>)

48.

Collagencin, an antibacterial peptide from fish collagen: Activity, structure and interaction dynamics with membrane.

Ennaas N., Hammami R., Gomaa A., Bedard F., Biron E., Subirade M., Beaulieu L., Fliss I.

Biochemical and Biophysical Research Communications. 473 (2) (pp 642-647), 2016. Date of Publication: 29 Apr 2016.

AN: 609470937

In this study, we first report characterization of collagencin, an antimicrobial peptide identified from fish collagen hydrolysate. The peptide completely inhibited the growth of Staphylococcus aureus at 1.88 mM. Although non-toxic up to 470 muM, collagencin was hemolytic at higher concentrations. The secondary structure of collagencin was mainly composed by beta-sheet and beta-turn as determined by CD measurements and molecular dynamics. The peptide is likely to form beta-sheet structure under hydrophobic environments and interacts with both anionic (phosphatidylglycerol) and zwitterionic (phosphoethanolamine and phosphatidylcholine) lipids as shown with CD spectroscopy and molecular dynamics. The peptide formed several hydrogen bonds with both POPG and POPE lipids and remained at membrane-water interface, suggesting that collagencin antibacterial action follows a carpet mechanism. Collagenous fish wastes could be processed by enzymatic hydrolysis and transformed into products of high value having functional or biological properties. Marine collagens are a promising source of antimicrobial peptides with new implications in food safety and human health.

Copyright © 2016 Elsevier Inc.

PMID

27038545 [<http://www.ncbi.nlm.nih.gov/pubmed/?term=27038545>]

Institution

(Ennaas, Hammami, Gomaa, Subirade, Beaulieu, Fliss) STELA Dairy Research Centre, Institute of Nutrition and Functional Foods, Universite Laval, Quebec, QC G1V 0A6, Canada (Bedard, Biron) Faculty of Pharmacy, Universite Laval, Laboratory of Medicinal Chemistry, CHU de Quebec Research Centre, Quebec, QC G1V 4G2, Canada

(Beaulieu) Department of Biology, Chemistry and Geography, Universite du Quebec A Rimouski (UQAR), 300 Allee des Ursulines, Rimouski, QC G5L 3A1, Canada

Publisher

Elsevier (E-mail: apjcs@harcourt.com)

Emtree Heading

Aeromonas hydrophila; *antibacterial activity; article; bacterial growth; bacterial membrane; beta sheet; beta turn; Carnobacterium; circular dichroism; concentration response; controlled study; Escherichia coli; fish; growth inhibition; hemolysis; hydrogen bond; hydrolysis; hydrophobicity; Lactococcus lactis; Listeria innocua; molecular dynamics; molecular model; nonhuman; priority journal; protein folding; protein lipid interaction; protein secondary structure; Pseudomonas aeruginosa; Staphylococcus aureus; Streptococcus pyogenes; ampholyte; *collagen derivative/to [Drug Toxicity]; *collagen derivative/pd [Pharmacology]; phosphatidylcholine; phosphatidylglycerol; phosphoethanolamine; *polypeptide antibiotic agent/to [Drug Toxicity]; *polypeptide antibiotic agent/pd [Pharmacology]; unclassified drug; water; *collagencin/to [Drug Toxicity]; *collagencin/pd [Pharmacology].

Candidate Terms

*collagencin / *drug toxicity / *pharmacology [drug term].

Drug Index Terms

ampholyte; *collagen derivative / *drug toxicity / *pharmacology; phosphatidylcholine; phosphatidylglycerol; phosphoethanolamine; *polypeptide antibiotic agent / *drug toxicity / *pharmacology; unclassified drug; water.

Other Index Terms

Aeromonas hydrophila; *antibacterial activity; Article; bacterial growth; bacterial membrane; beta sheet; beta turn; Carnobacterium; circular dichroism; concentration response; controlled study; Escherichia coli; fish; growth inhibition; hemolysis; hydrogen bond; hydrolysis; hydrophobicity; Lactococcus lactis; Listeria innocua; molecular dynamics; molecular model; nonhuman; priority journal; protein folding; protein lipid interaction; protein secondary structure; Pseudomonas aeruginosa; Staphylococcus aureus; Streptococcus pyogenes.

Link to the Ovid Full Text or citation:

[Click here for full text options](https://libaccess.mcmaster.ca/login?url=http://ovidsp.ovid.com/ovidweb.cgi?T=JS&CSC=Y&NEWS=N&PAGE=fulltext&D=emed17&AN=609470937)

Link to the External Link Resolver:

[SFX](http://sfx.scholarsportal.info/mcmaster?sid=OVID:embase&id=pmid:27038545&id=doi:10.1016%2Fj.bbrc.2016.03.121&issn=0006-291X&isbn=&volume=473&issue=2&spage=642&pages=642-647&date=2016&title=Biochemical+and+Biophysical+Research+Communications&atitle=Collagencin%2C+an+antibacterial+peptide+from+fish+collagen%3A+Activity%2C+structure+and+interaction+dynamics+with+membrane&aulast=Ennaas&pid=<author>Ennaas+N.%3BHammami+R.%3BGomaa+A.%3BBedard+F.%3BBiron+E.%3BSubirade+M.%3BBeaulieu+L.%3BFliss+I.<%2Fauthor><AN>609470937<%2FAN><DT>Article<%2FDT>)

49.

Repeat-based Sequence Typing of Carnobacterium maltaromaticum.

Rahman A., El Kheir S.M., Back A., Mangavel C., Revol-Junelles A.-M., Borges F.

International Journal of Food Microbiology. 226 (pp 1-4), 2016. Date of Publication: June 02, 2016.

AN: 609079537

Carnobacterium maltaromaticum is a Lactic Acid Bacterium (LAB) of technological interest for the food industry, especially the dairy as bioprotection and ripening flora. The industrial use of this LAB requires accurate and resolutive typing tools. A new typing method for C. maltaromaticum inspired from MLVA analysis and called Repeat-based Sequence Typing (RST) is described. Rather than electrophoresis analysis, our RST method is based on sequence analysis of multiple loci containing Variable-Number Tandem-Repeats (VNTRs). The method described here for C. maltaromaticum relies on the analysis of three VNTR loci, and was applied to a collection of 24 strains. For each strain, a PCR product corresponding to the amplification of each VNTR loci was sequenced. Sequence analysis allowed delineating 11, 11, and 12 alleles for loci VNTR-A, VNTR-B, and VNTR-C, respectively. Considering the allele combination exhibited by each strain allowed defining 15 genotypes, ending in a discriminatory index of 0.94. Comparison with MLST revealed that both methods were complementary for strain typing in C. maltaromaticum.

Copyright © 2016 Elsevier B.V.

Institution

(Rahman, El Kheir, Back, Mangavel, Revol-Junelles, Borges) Universite de Lorraine, Laboratoire d'Ingenierie des Biomolecules (LIBio), ENSAIA, 2 avenue de la Foret de Haye, TSA, Vandoeuvre-les-Nancy 40602 54518, France (Rahman) National University of Sciences and Technology, Atta ur Rahman School of Applied Biosciences, Department of Industrial Biotechnology, H-12, Islamabad 44000, Pakistan

Publisher

Elsevier

Emtree Heading

allele; *Carnobacterium maltaromaticum; clinical article; DNA structure; electrophoresis; gene amplification; genotype; human; intermethod comparison; polymerase chain reaction; sequence analysis; variable number of tandem repeat.

Other Index Terms

allele; *Carnobacterium maltaromaticum; clinical article; DNA structure; electrophoresis; gene amplification; genotype; human; intermethod comparison; polymerase chain reaction; sequence analysis; variable number of tandem repeat.

Link to the Ovid Full Text or citation:

[Click here for full text options](https://libaccess.mcmaster.ca/login?url=http://ovidsp.ovid.com/ovidweb.cgi?T=JS&CSC=Y&NEWS=N&PAGE=fulltext&D=emed17&AN=609079537)

Link to the External Link Resolver:

[SFX](http://sfx.scholarsportal.info/mcmaster?sid=OVID:embase&id=pmid:&id=doi:10.1016%2Fj.ijfoodmicro.2016.03.003&issn=0168-1605&isbn=&volume=226&issue=&spage=1&pages=1-4&date=2016&title=International+Journal+of+Food+Microbiology&atitle=Repeat-based+Sequence+Typing+of+Carnobacterium+maltaromaticum&aulast=Rahman&pid=<author>Rahman+A.%3BEl+Kheir+S.M.%3BBack+A.%3BMangavel+C.%3BRevol-Junelles+A.-M.%3BBorges+F.<%2Fauthor><AN>609079537<%2FAN><DT>Article<%2FDT>)

50.

Fighting Off Wound Pathogens in Horses with Honeybee Lactic Acid Bacteria.

Olofsson T.C., Butler E., Lindholm C., Nilson B., Michanek P., Vasquez A.

Current Microbiology. 73 (4) (pp 463-473), 2016. Date of Publication: 01 Oct 2016.

AN: 610906093

In the global perspective of antibiotic resistance, it is urgent to find potent topical antibiotics for the use in human and animal infection. Healing of equine wounds, particularly in the limbs, is difficult due to hydrostatic factors and exposure to environmental contaminants, which can lead to heavy bio-burden/biofilm formation and sometimes to infection. Therefore, antibiotics are often prescribed. Recent studies have shown that honeybee-specific lactic acid bacteria (LAB), involved in honey production, and inhibit human wound pathogens. The aim of this pilot study was to investigate the effects on the healing of hard-to-heal equine wounds after treatment with these LAB symbionts viable in a heather honey formulation. For this, we included ten horses with wound duration of >1 year, investigated the wound microbiota, and treated wounds with the novel honeybee LAB formulation. We identified the microbiota using MALDI-TOF mass spectrometry and DNA sequencing. In addition, the antimicrobial properties of the honeybee LAB formulation were tested against all wound isolates in vitro. Our results indicate a diverse wound microbiota including fifty-three bacterial species that showed 90 % colonization by at least one species of Staphylococcus. Treatment with the formulation promoted wound healing in all cases already after the first application and the wounds were either completely healed (n = 3) in less than 20 days or healing was in progress. Furthermore, the honeybee LAB formulation inhibited all pathogens when tested in vitro. Consequently, this new treatment option presents as a powerful candidate for the topical treatment of hard-to-heal wounds in horses.

Copyright © 2016, The Author(s).

PMID

27324340 [<http://www.ncbi.nlm.nih.gov/pubmed/?term=27324340>]

Institution

(Olofsson, Butler, Vasquez) Laboratory Medicine, Lunds Universitet, Lund, Sweden (Lindholm) Division of Nursing Science, Sophiahemmet Hogskola, Stockholm, Sweden

(Nilson) Laboratory Medicine, Clinical Microbiology, Region Skane, Lund, Sweden

(Nilson) Department of Laboratory Medicine Lund, Medical Microbiology, Lund University, Solvegatan 23, Lund 22362, Sweden

(Michanek) Animal Farm Veterinary Consultants, Degebergavagen, Vollsjo 27568, Sweden

Publisher

Springer New York LLC (E-mail: barbara.b.bertram@gsk.com)

Emtree Heading

Acinetobacter; Acinetobacter lwoffii; Actinomycetales; Aerococcus viridans; Alcaligenes faecalis; antibacterial activity; *antibiotic therapy; Arthrobacter; article; Bacillus cereus; Bacillus mycoides; Bacillus pumilus; Bacillus subtilis; *bacterium identification; Bacteroides; Brevibacterium; Candida; Candida parapsilosis; Carnobacterium; Citrobacter; Clostridium; controlled clinical trial; controlled study; Corynebacterium amycolatum; Corynebacterium diphtheriae; Corynebacterium glutamicum; DNA sequence; Enterobacter; Enterobacter aerogenes; Enterococcus faecalis; Enterococcus faecium; epithelization; gene sequence; Gordonia; *honeybee; *horse disease/dt [Drug Therapy]; *horse disease/su [Surgery]; horse disease/dt [Drug Therapy]; Klebsiella oxytoca; matrix assisted laser desorption ionization time of flight mass spectrometry; Micrococcus; nonhuman; Pantoea agglomerans; Pasteurella; pilot study; priority journal; Proteus vulgaris; Psychrobacter; Staphylococcus; Staphylococcus aureus; Staphylococcus epidermidis; Staphylococcus hyicus; Staphylococcus lugdunensis; Staphylococcus pseudintermedius; Staphylococcus schleiferi; Staphylococcus sciuri; Staphylococcus xylosus; Streptococcus; Streptococcus dysgalactiae; Streptococcus equi; Streptococcus equinus; Streptomyces; wound closure; wound healing; *wound infection/dt [Drug Therapy]; *wound infection/su [Surgery]; wound infection/dt [Drug Therapy]; bacterial RNA; *natural product/ct [Clinical Trial]; *natural product/dt [Drug Therapy]; *natural product/pd [Pharmacology]; RNA 16S; Acinetobacter towneri; Aeromonas bestiarum; Aeromonas encheliea; Arthrobacter arilaitensis; Arthrobacter castelli; Arthrobacter gandavensis; Bacteroides pyogenes; Brachybacterium faecium; Brevibacterium conglomeratum; Citrobacter braakii; clostridium absonum; Corynebacterium casei; Enterobacter ludwigii; Gordonia hirsuta; Macrococcus; Pasteurella canis; Peptonophilus indolicus; Psychrobacter sanguinis; Staphylococcus chromogenes; Staphylococcus delphini; Staphylococcus equorum; Staphylococcus vitulinus; Streptococcus parauberis; streptomyces badius.

Candidate Terms

Acinetobacter towneri [other term]; Aeromonas bestiarum [other term]; Aeromonas encheliea [other term]; Arthrobacter arilaitensis [other term]; Arthrobacter castelli [other term]; Arthrobacter gandavensis [other term]; Bacteroides pyogenes [other term]; Brachybacterium faecium [other term]; Brevibacterium conglomeratum [other term]; Citrobacter braakii [other term]; Clostridium absonum [other term]; Corynebacterium casei [other term]; Enterobacter ludwigii [other term]; Gordonia hirsuta [other term]; Macrococcus [other term]; Pasteurella canis [other term]; Peptonophilus indolicus [other term]; Psychrobacter sanguinis [other term]; Staphylococcus chromogenes [other term]; Staphylococcus delphini [other term]; Staphylococcus equorum [other term]; Staphylococcus vitulinus [other term]; Streptococcus parauberis [other term]; Streptomyces badius [other term].

Drug Index Terms

bacterial RNA; *natural product / *clinical trial / *drug therapy / *pharmacology; RNA 16S.

Other Index Terms

Acinetobacter; Acinetobacter lwoffii; Actinomycetales; Aerococcus viridans; Alcaligenes faecalis; antibacterial activity; *antibiotic therapy; Arthrobacter; Article; Bacillus cereus; Bacillus mycoides; Bacillus pumilus; Bacillus subtilis; *bacterium identification; Bacteroides; Brevibacterium; Candida; Candida parapsilosis; Carnobacterium; Citrobacter; Clostridium; controlled clinical trial; controlled study; Corynebacterium amycolatum; Corynebacterium diphtheriae; Corynebacterium glutamicum; DNA sequence; Enterobacter; Enterobacter aerogenes; Enterococcus faecalis; Enterococcus faecium; epithelization; gene sequence; Gordonia; *honeybee; *horse disease / *drug therapy / *surgery; horse disease / drug therapy; Klebsiella oxytoca; matrix assisted laser desorption ionization time of flight mass spectrometry; Micrococcus; nonhuman; Pantoea agglomerans; Pasteurella; pilot study; priority journal; Proteus vulgaris; Psychrobacter; Staphylococcus; Staphylococcus aureus; Staphylococcus epidermidis; Staphylococcus hyicus; Staphylococcus lugdunensis; Staphylococcus pseudintermedius; Staphylococcus schleiferi; Staphylococcus sciuri; Staphylococcus xylosus; Streptococcus; Streptococcus dysgalactiae; Streptococcus equi; Streptococcus equinus; Streptomyces; wound closure; wound healing; *wound infection / *drug therapy / *surgery; wound infection / drug therapy.

Link to the Ovid Full Text or citation:

[Click here for full text options](https://libaccess.mcmaster.ca/login?url=http://ovidsp.ovid.com/ovidweb.cgi?T=JS&CSC=Y&NEWS=N&PAGE=fulltext&D=emed17&AN=610906093)

Link to the External Link Resolver:

[SFX](http://sfx.scholarsportal.info/mcmaster?sid=OVID:embase&id=pmid:27324340&id=doi:10.1007%2Fs00284-016-1080-2&issn=0343-8651&isbn=&volume=73&issue=4&spage=463&pages=463-473&date=2016&title=Current+Microbiology&atitle=Fighting+Off+Wound+Pathogens+in+Horses+with+Honeybee+Lactic+Acid+Bacteria&aulast=Olofsson&pid=<author>Olofsson+T.C.%3BButler+E.%3BLindholm+C.%3BNilson+B.%3BMichanek+P.%3BVasquez+A.<%2Fauthor><AN>610906093<%2FAN><DT>Article<%2FDT>)

51.

Clostridium perfringens endophthalmitis after penetrating keratoplasty with contaminated corneal allografts: A case series.

Hou J.H., Tannan A., Rubenstein J.B., Lopez O.I., McCoy K., Epstein R.J., Grostern R.J., Sugar J.

Cornea. 34 (1) (pp 23-27), 2015. Date of Publication: 12 Jan 2015.

AN: 600768501

Purpose: To report the postoperative clinical course of 3 patients who underwent corneal transplantation with corneal allografts contaminated with Clostridium perfringens and to evaluate the risk factors for anaerobic contamination in 2 donors. Methods: Patient records and adverse reaction reports from a single eye bank related to cases of posttransplant C. perfringens endophthalmitis were reviewed. Records regarding the mated corneas, donor autopsy reports, and other pertinent data were also reviewed.

Results: Three adverse reactions associated with transplantation of corneal allografts contaminated with C. perfringens were reported. Two cases were from mated corneas. Both patients developed fulminant endophthalmitis after undergoing uncomplicated penetrating keratoplasty and required subsequent enucleation. Another isolated case (with no adverse reaction in the mate cornea) developed hypopyon postoperatively that resolved with intravitreal and topical antibiotics. Possible risk factors for anaerobic tissue contamination in the donors included illicit drug use in the first donor and exposure to sewage at the time of death in the second donor.

Conclusions: Clostridial endophthalmitis is an aggressive rapidly progressive infection with potentially poor visual outcomes that can be transmitted from infected corneal allografts. Further investigation is needed to clarify the role of anaerobic donor rim cultures and the donor risk factors associated with recovering corneal allograft tissue contaminated with C. perfringens.

Copyright © 2014 by Lippincott Williams & Wilkins.

PMID

25393093 [<http://www.ncbi.nlm.nih.gov/pubmed/?term=25393093>]

Institution

(Hou, Sugar) Department of Ophthalmology and Visual Sciences, University of Illinois Eye and Ear Infirmary, 1855 W. Taylor St, Chicago, IL 60612, United States (Tannan, Rubenstein, Epstein, Grostern) Department of Ophthalmology, Rush University Medical Center, Chicago, IL, United States

(Lopez) Chicago Eye Institute, Chicago, IL, United States

(McCoy) Midwest Eye-Banks, Ann Arbor, MI, United States

Publisher

Lippincott Williams and Wilkins (E-mail: kathiest.clai@apta.org)

Emtree Heading

abdominal injury; adult; aged; antibiotic therapy; aphakia/su [Surgery]; article; autopsy; borderline hypertension; cardiopulmonary arrest; Carnobacterium maltaromaticum; case report; chemosis; chronic pain; *Clostridium perfringens; cornea dystrophy/su [Surgery]; cornea edema; cornea necrosis; *cornea transplantation; coronary artery disease; diabetes mellitus; dialysis; drug abuse; end stage renal disease; *endophthalmitis/co [Complication]; Enterococcus faecalis; Escherichia coli; evaluation study; eyebank; eyelid edema; female; follow up; gout; hemorrhagic shock; human; human tissue; hypertension; hyphema; hypopyon/dt [Drug Therapy]; intestine perforation; keratoconus/su [Surgery]; keratopathy/su [Surgery]; lens implantation; male; middle aged; multiple trauma; nonhuman; open angle glaucoma; orbit cellulitis; orbit reconstruction; organ donor; patellofemoral pain syndrome; Pediococcus pentosaceus; *penetrating keratoplasty; postoperative care; sleep disordered breathing; Streptococcus gordonii; thorax pain; time of death; treatment duration; very elderly; visual acuity; vitrectomy; amphetamine; amphetamine derivative; benzodiazepine derivative; caffeine; cannabinoid derivative; ceftazidime/dt [Drug Therapy]; ceftazidime/vi [Intravitreal Drug Administration]; ciprofloxacin/po [Oral Drug Administration]; clonazepam; diphenhydramine; morphine; moxifloxacin/tp [Topical Drug Administration]; opiate derivative; piperacillin plus tazobactam/iv [Intravenous Drug Administration]; prednisolone acetate; tobramycin/dt [Drug Therapy]; tobramycin/cj [Subconjunctival Drug Administration]; tobramycin/tp [Topical Drug Administration]; vancomycin/dt [Drug Therapy]; vancomycin/iv [Intravenous Drug Administration]; vancomycin/vi [Intravitreal Drug Administration]; vancomycin/cj [Subconjunctival Drug Administration].

Drug Index Terms

amphetamine; amphetamine derivative; benzodiazepine derivative; caffeine; cannabinoid derivative; ceftazidime / drug therapy / intravitreal drug administration; ciprofloxacin / oral drug administration; clonazepam; diphenhydramine; morphine; moxifloxacin / topical drug administration; opiate derivative; piperacillin plus tazobactam / intravenous drug administration; prednisolone acetate; tobramycin / drug therapy / subconjunctival drug administration / topical drug administration; vancomycin / drug therapy / intravenous drug administration / intravitreal drug administration / subconjunctival drug administration.

Other Index Terms

abdominal injury; adult; aged; antibiotic therapy; aphakia / surgery; Article; autopsy; borderline hypertension; cardiopulmonary arrest; Carnobacterium maltaromaticum; case report; chemosis; chronic pain; *Clostridium perfringens; cornea dystrophy / surgery; cornea edema; cornea necrosis; *cornea transplantation; coronary artery disease; diabetes mellitus; dialysis; drug abuse; end stage renal disease; *endophthalmitis / *complication; Enterococcus faecalis; Escherichia coli; evaluation study; eyebank; eyelid edema; female; follow up; gout; hemorrhagic shock; human; human tissue; hypertension; hyphema; hypopyon / drug therapy; intestine perforation; keratoconus / surgery; keratopathy / surgery; lens implantation; male; middle aged; multiple trauma; nonhuman; open angle glaucoma; orbit cellulitis; orbit reconstruction; organ donor; patellofemoral pain syndrome; Pediococcus pentosaceus; *penetrating keratoplasty; postoperative care; sleep disordered breathing; Streptococcus gordonii; thorax pain; time of death; treatment duration; very elderly; visual acuity; vitrectomy.

Link to the Ovid Full Text or citation:

[Click here for full text options](https://libaccess.mcmaster.ca/login?url=http://ovidsp.ovid.com/ovidweb.cgi?T=JS&CSC=Y&NEWS=N&PAGE=fulltext&D=emed16&AN=600768501)

Link to the External Link Resolver:

[SFX](http://sfx.scholarsportal.info/mcmaster?sid=OVID:embase&id=pmid:25393093&id=doi:10.1097%2FICO.0000000000000303&issn=0277-3740&isbn=&volume=34&issue=1&spage=23&pages=23-27&date=2015&title=Cornea&atitle=Clostridium+perfringens+endophthalmitis+after+penetrating+keratoplasty+with+contaminated+corneal+allografts%3A+A+case+series&aulast=Hou&pid=<author>Hou+J.H.%3BTannan+A.%3BRubenstein+J.B.%3BLopez+O.I.%3BMcCoy+K.%3BEpstein+R.J.%3BGrostern+R.J.%3BSugar+J.<%2Fauthor><AN>600768501<%2FAN><DT>Article<%2FDT>)

52.

Two novel regulators of N-acetyl-galactosamine utilization pathway and distinct roles in bacterial infections.

Zhang H., Ravcheev D.A., Hu D., Zhang F., Gong X., Hao L., Cao M., Rodionov D.A., Wang C., Feng Y.

MicrobiologyOpen. 4 (6) (pp 983-1000), 2015. Date of Publication: 01 Dec 2015.

AN: 607130885

Bacterial pathogens can exploit metabolic pathways to facilitate their successful infection cycles, but little is known about roles of d-galactosamine (GalN)/N-acetyl-d-galactosamine (GalNAc) catabolism pathway in bacterial pathogenesis. Here, we report the genomic reconstruction of GalN/GalNAc utilization pathway in Streptococci and the diversified aga regulons. We delineated two new paralogous AgaR regulators for the GalN/GalNAc catabolism pathway. The electrophoretic mobility shift assays experiment demonstrated that AgaR2 (AgaR1) binds the predicted palindromes, and the combined in vivo data from reverse transcription quantitative polymerase chain reaction and RNA-seq suggested that AgaR2 (not AgaR1) can effectively repress the transcription of the target genes. Removal of agaR2 (not agaR1) from Streptococcus suis 05ZYH33 augments significantly the abilities of both adherence to Hep-2 cells and anti-phagocytosis against RAW264.7 macrophage. As anticipated, the dysfunction in AgaR2-mediated regulation of S. suis impairs its pathogenicity in experimental models of both mice and piglets. Our finding discovered two novel regulators specific for GalN/GalNAc catabolism and assigned them distinct roles into bacterial infections. To the best of our knowledge, it might represent a first paradigm that links the GalN/GalNAc catabolism pathway to bacterial pathogenesis. Bacterial pathogens including Streptococcus species have evolved multiple strategies to hijack/exploit metabolic pathways for facilitating their successful infection cycles. Although that the two amino sugars (d-galactosamine [GalN] and N-acetyl-d-galactosamine [GalNAc]) function as the common components of various cell surface structures and are required for three domains of life, this metabolism is poorly known in Streptococcus species. Nothing is elucidated regarding the relevance of GalN/GalNAc catabolism pathway to bacterial pathogenesis. We report, for the first time, the genomic reconstruction of GalN/GalNAc utilization pathway with variations in Firmicutes (and/or Streptococci) and the diversified aga regulons. More importantly, we are first to define that the maintenance/regulation of GalN/GalNAc utilization pathway contributes to Streptococcal infections. To the best of our knowledge, it represents a first paradigm that links the GalN/GalNAc catabolism pathway to bacterial pathogenesis.

Copyright © 2015 Published by John Wiley & Sons Ltd.

PMID

26540018 [<http://www.ncbi.nlm.nih.gov/pubmed/?term=26540018>]

Institution

(Zhang, Feng) Department of Medical Microbiology and Parasitology, Zhejiang University School of Medicine, Hangzhou, Zhejiang 310058, China (Rodionov) A.A. Kharkevich Institute for Information Transmission Problems, Russian Academy of Sciences, Moscow 127994, Russian Federation

(Hu, Zhang, Gong, Hao, Cao, Wang) Department of Epidemiology, Research Institute for Medicine of Nanjing Command, Nanjing 210002, China

(Ravcheev) Luxembourg Centre for Systems Biomedicine, University of Luxembourg, Esch-sur-Alzette L-4360, Luxembourg

Publisher

Blackwell Publishing Ltd (E-mail: customerservices@oxonblackwellpublishing.com)

Emtree Heading

animal cell; animal experiment; article; *bacterial infection; bacterial strain; bacterial virulence; bacterium adherence; Carnobacterium; controlled study; cross linking; down regulation; Enterococcus faecalis; gel mobility shift assay; gene amplification; genetic regulation; human; human cell; Lactobacillales; liquid chromatography; mouse; nonhuman; phagocytosis; piglet; polyacrylamide gel electrophoresis; priority journal; protein binding; *protein degradation; protein expression; protein motif; quadrupole mass spectrometry; real time polymerase chain reaction; reverse transcription polymerase chain reaction; RNA isolation; RNA sequence; size exclusion chromatography; Streptococcus dysgalactiae; Streptococcus equi; Streptococcus gordonii; Streptococcus mitis; Streptococcus pneumoniae; Streptococcus pyogenes; Streptococcus sanguinis; Streptococcus suis; Streptococcus uberis; upregulation; Western blotting; complementary DNA/ec [Endogenous Compound]; glutamate ammonia ligase/ec [Endogenous Compound]; *n acetylgalactosamine/ec [Endogenous Compound]; palindromic DNA/ec [Endogenous Compound]; transcription factor/ec [Endogenous Compound]; unclassified drug; Streptococcus gasseri; Streptococcus helveticus; Streptococcus johnsonii; Streptococcus pentosaceus; Streptococcus rhamnosus; *AgaR2 protein/ec [Endogenous Compound]; transcription factor AgaR1/ec [Endogenous Compound]; transcription factor AgaR2/ec [Endogenous Compound].

Candidate Terms

Streptococcus gasseri [other term]; Streptococcus helveticus [other term]; Streptococcus johnsonii [other term]; Streptococcus pentosaceus [other term]; Streptococcus rhamnosus [other term]; *AgaR2 protein / *endogenous compound [drug term]; transcription factor AgaR1 / endogenous compound [drug term]; transcription factor AgaR2 / endogenous compound [drug term].

Drug Index Terms

complementary DNA / endogenous compound; glutamate ammonia ligase / endogenous compound; *n acetylgalactosamine / *endogenous compound; palindromic DNA / endogenous compound; transcription factor / endogenous compound; unclassified drug.

Other Index Terms

animal cell; animal experiment; Article; *bacterial infection; bacterial strain; bacterial virulence; bacterium adherence; Carnobacterium; controlled study; cross linking; down regulation; Enterococcus faecalis; gel mobility shift assay; gene amplification; genetic regulation; human; human cell; Lactobacillales; liquid chromatography; mouse; nonhuman; phagocytosis; piglet; polyacrylamide gel electrophoresis; priority journal; protein binding; *protein degradation; protein expression; protein motif; quadrupole mass spectrometry; real time polymerase chain reaction; reverse transcription polymerase chain reaction; RNA isolation; RNA sequence; size exclusion chromatography; Streptococcus dysgalactiae; Streptococcus equi; Streptococcus gordonii; Streptococcus mitis; Streptococcus pneumoniae; Streptococcus pyogenes; Streptococcus sanguinis; Streptococcus suis; Streptococcus uberis; upregulation; Western blotting.

Link to the Ovid Full Text or citation:

[Click here for full text options](https://libaccess.mcmaster.ca/login?url=http://ovidsp.ovid.com/ovidweb.cgi?T=JS&CSC=Y&NEWS=N&PAGE=fulltext&D=emed16&AN=607130885)

Link to the External Link Resolver:

[SFX](http://sfx.scholarsportal.info/mcmaster?sid=OVID:embase&id=pmid:26540018&id=doi:10.1002%2Fmbo3.307&issn=2045-8827&isbn=&volume=4&issue=6&spage=983&pages=983-1000&date=2015&title=MicrobiologyOpen&atitle=Two+novel+regulators+of+N-acetyl-galactosamine+utilization+pathway+and+distinct+roles+in+bacterial+infections&aulast=Zhang&pid=<author>Zhang+H.%3BRavcheev+D.A.%3BHu+D.%3BZhang+F.%3BGong+X.%3BHao+L.%3BCao+M.%3BRodionov+D.A.%3BWang+C.%3BFeng+Y.<%2Fauthor><AN>607130885<%2FAN><DT>Article<%2FDT>)

53.

The microbiome of the upper airways: focus on chronic rhinosinusitis.

Chalermwatanachai T., Velasquez L.C., Bachert C.

World Allergy Organization Journal. 8 (1) (no pagination), 2015. Article Number: 48. Date of Publication: 27 Jan 2015.

AN: 605618627

Abstract Upper airway diseases including allergic rhinitis, chronic rhinosinusitis with or without polyps, and cystic fibrosis are characterized by substantially different inflammatory profiles. Traditionally, studies on the association of specific bacterial patterns with inflammatory profiles of diseases had been dependent on bacterial culturing. In the past 30 years, molecular biology methods have allowed bacterial culture free studies of microbial communities, revealing microbiota much more diverse than previously recognized including those found in the upper airway. At presence, the study of the pathophysiology of upper airway diseases is necessary to establish the relationship between the microbiome and inflammatory patterns to find their clinical reflections and also their possible causal relationships. Such investigations may elucidate the path to therapeutic approaches in correcting an imbalanced microbiome. In the review we summarized techniques used and the current knowledge on the microbiome of upper airway diseases, the limitations and pitfalls, and identified areas of interest for further research.

Copyright © 2015 Chalermwatanachai et al.; licensee BioMed Central.

Institution

(Chalermwatanachai, Velasquez, Bachert) Department of Oto-Rhino-Laryngology, Upper Airways Research Laboratory (URL), Ghent University Hospital, Ghent 9000, Belgium (Chalermwatanachai) Department of Otolaryngology, Phramongkutklao Hospital, College of Medicine, Bangkok 10400, Thailand

(Velasquez) Basic Biomedical Sciences Department, Health Faculty, Universidad Industrial de Santander, Bucaramanga, Colombia

(Bachert) Division of ENT Diseases, Clintec, Karolinska Institutet, Stockholm, Sweden

Publisher

BioMed Central Ltd. (E-mail: info@biomedcentral.com)

Emtree Heading

allergic rhinitis; article; bacterium culture; Burkholderia cepacia; Campylobacter; Capnocytophaga; Carnobacterium; *chronic rhinosinusitis; Citrobacter; Corynebacterium; Curtobacterium; cyanobacterium; cystic fibrosis; Enterobacter; Enterococcus; Fusobacterium; Granulicatella; Haemophilus; Helicobacter; human; Lactobacillus sakei; Leptotrichia; *microbiome; Micrococcus; microflora; Moraxella; Mycobacterium; Neisseria; nonhuman; Ochrobactrum; Peptostreptococcus; Porphyromonas; Prevotella; priority journal; Propionibacterium acnes; Pseudomonas aeruginosa; Rhizobium; Rothia; Staphylococcus aureus; Staphylococcus epidermidis; Stenotrophomonas; Streptococcus anginosus; Streptococcus constellatus; Streptococcus intermedius; Streptococcus milleri; validation process; Veillonella.

Other Index Terms

allergic rhinitis; Article; bacterium culture; Burkholderia cepacia; Campylobacter; Capnocytophaga; Carnobacterium; *chronic rhinosinusitis; Citrobacter; Corynebacterium; Curtobacterium; cyanobacterium; cystic fibrosis; Enterobacter; Enterococcus; Fusobacterium; Granulicatella; Haemophilus; Helicobacter; human; Lactobacillus sakei; Leptotrichia; *microbiome; Micrococcus; microflora; Moraxella; Mycobacterium; Neisseria; nonhuman; Ochrobactrum; Peptostreptococcus; Porphyromonas; Prevotella; priority journal; Propionibacterium acnes; Pseudomonas aeruginosa; Rhizobium; Rothia; Staphylococcus aureus; Staphylococcus epidermidis; Stenotrophomonas; Streptococcus anginosus; Streptococcus constellatus; Streptococcus intermedius; Streptococcus milleri; validation process; Veillonella.

Link to the Ovid Full Text or citation:

[Click here for full text options](https://libaccess.mcmaster.ca/login?url=http://ovidsp.ovid.com/ovidweb.cgi?T=JS&CSC=Y&NEWS=N&PAGE=fulltext&D=emed16&AN=605618627)

Link to the External Link Resolver:

[SFX](http://sfx.scholarsportal.info/mcmaster?sid=OVID:embase&id=pmid:&id=doi:10.1186%2Fs40413-014-0048-6&issn=1939-4551&isbn=&volume=8&issue=1&spage=&pages=&date=2015&title=World+Allergy+Organization+Journal&atitle=The+microbiome+of+the+upper+airways%3A+focus+on+chronic+rhinosinusitis&aulast=Chalermwatanachai&pid=<author>Chalermwatanachai+T.%3BVelasquez+L.C.%3BBachert+C.<%2Fauthor><AN>605618627<%2FAN><DT>Article<%2FDT>)

54.

Carnobacterium divergens Bacteremia in Woman.

Smati M., Palacios C., Cohen Y., Mechai F., Tankovic J., Le Fleche-Mateos A., Picard B., Gonzalez F.

Emerging Infectious Diseases. 21 (6) (pp 1081-1082), 2015. Date of Publication: 2015.

AN: 604440145

Institution

(Smati, Palacios, Cohen, Mechai, Picard, Gonzalez) Avicenne University Hospital, Bobigny, France (Tankovic) Saint-Antoine University Hospital, Paris, France

(Le Fleche-Mateos) Pasteur Institute, Paris, France

Publisher

Centers for Disease Control and Prevention (CDC) (E-mail: cdcinfo@cdc.gov)

Emtree Heading

adult; alcoholism; ascites; *bacteremia/di [Diagnosis]; *Carnobacterium; case report; computer assisted tomography; diabetic ketoacidosis; drug substitution; esophagus resection; female; fever; gastrointestinal endoscopy; gastrostomy; heart arrest; human; laparotomy; letter; *listeriosis/di [Diagnosis]; *listeriosis/dt [Drug Therapy]; listeriosis/dt [Drug Therapy]; malnutrition; middle aged; minimum inhibitory concentration; necrotizing esophagitis/di [Diagnosis]; nonhuman; peptic ulcer; pneumoperitoneum/dt [Drug Therapy]; resuscitation; septic shock/dt [Drug Therapy]; amikacin/dt [Drug Therapy]; amoxicillin/dt [Drug Therapy]; amoxicillin plus clavulanic acid; bacteriocin/ec [Endogenous Compound]; carbapenem derivative; cefotaxime; ceftazidime/dt [Drug Therapy]; cephalosporin derivative; cilastatin plus imipenem/dt [Drug Therapy]; ciprofloxacin; colistin/dt [Drug Therapy]; gentamicin/dt [Drug Therapy]; imipenem; linezolid; macrolide; metronidazole/dt [Drug Therapy]; ofloxacin; penicillin derivative; piperacillin plus tazobactam/dt [Drug Therapy]; rifampicin; RNA 16S/ec [Endogenous Compound]; teicoplanin; vancomycin; *Carnobacterium divergens.

Candidate Terms

*Carnobacterium divergens [other term].

Drug Index Terms

amikacin / drug therapy; amoxicillin / drug therapy; amoxicillin plus clavulanic acid; bacteriocin / endogenous compound; carbapenem derivative; cefotaxime; ceftazidime / drug therapy; cephalosporin derivative; cilastatin plus imipenem / drug therapy; ciprofloxacin; colistin / drug therapy; gentamicin / drug therapy; imipenem; linezolid; macrolide; metronidazole / drug therapy; ofloxacin; penicillin derivative; piperacillin plus tazobactam / drug therapy; rifampicin; RNA 16S / endogenous compound; teicoplanin; vancomycin.

Other Index Terms

adult; alcoholism; ascites; *bacteremia / *diagnosis; *Carnobacterium; case report; computer assisted tomography; diabetic ketoacidosis; drug substitution; esophagus resection; female; fever; gastrointestinal endoscopy; gastrostomy; heart arrest; human; laparotomy; Letter; *listeriosis / *diagnosis / *drug therapy; listeriosis / drug therapy; malnutrition; middle aged; minimum inhibitory concentration; necrotizing esophagitis / diagnosis; nonhuman; peptic ulcer; pneumoperitoneum / drug therapy; resuscitation; septic shock / drug therapy.

Link to the Ovid Full Text or citation:

[Click here for full text options](https://libaccess.mcmaster.ca/login?url=http://ovidsp.ovid.com/ovidweb.cgi?T=JS&CSC=Y&NEWS=N&PAGE=fulltext&D=emed16&AN=604440145)

Link to the External Link Resolver:

[SFX](http://sfx.scholarsportal.info/mcmaster?sid=OVID:embase&id=pmid:&id=doi:10.3201%2Feid2106.141799&issn=1080-6040&isbn=&volume=21&issue=6&spage=1081&pages=1081-1082&date=2015&title=Emerging+Infectious+Diseases&atitle=Carnobacterium+divergens+Bacteremia+in+Woman&aulast=Smati&pid=<author>Smati+M.%3BPalacios+C.%3BCohen+Y.%3BMechai+F.%3BTankovic+J.%3BLe+Fleche-Mateos+A.%3BPicard+B.%3BGonzalez+F.<%2Fauthor><AN>604440145<%2FAN><DT>Letter<%2FDT>)

55.

Evaluation of enterococcus spp. from rainbow trout (Oncorhynchus mykiss, Walbaum), feed, and rearing environment against fish pathogens.

Araujo C., Munoz-Atienza E., Hernandez P.E., Herranz C., Cintas L.M., Igrejas G., Poeta P.

Foodborne Pathogens and Disease. 12 (4) (pp 311-322), 2015. Date of Publication: 01 Apr 2015.

AN: 603610000

The use of lactic acid bacteria of aquatic origin as probiotics constitutes an alternative strategy to the antibiotic treatment for disease control in aquaculture. Enterococci are currently used as probiotics in human and animal health. In this study, we evaluated the safety of 64 enterococci isolated from rainbow trout (Oncorhynchus mykiss, Walbaum), feed and rearing environment, and their antimicrobial activity against 9 fish pathogens. The 64 enterococcal isolates were identified to the species level by polymerase chain reaction (PCR), using specific primers for the different enterococcal species, and confirmed by superoxide dismutase gene sequencing. Enterococcus faecium and E. hirae were the most common species (42.2 and 35.9%, respectively). A total of 48 isolates (75%) showed phenotypic resistance to at least 1 antibiotic determined by a disk-diffusion method, and 25 isolates (39.1%) harbored at least 1 antibiotic resistance gene [erm(B), tet(M), tet(S), tet(K), tet(L), tet(T), vanC2, and aad(E)], detected by PCR. One (1.6%) isolate produced gelatinase and none produced hemolysin, using a plate assay. The virulence genes gelE (46.9%), efaAfs (17.2%), agg (1.6%), and hyl (1.6%) were detected by PCR. A total of 48 isolates (75%) exerted antimicrobial activity against 1 or more of the tested fish pathogens, using a stab-on-agar test. From these isolates, 21 (43.8%) harbored at least 1 bacteriocin-encoding gene (entP, entL50A and entL50B, hirJM79, entSE-K4, entQ and entA), detected by PCR. None of the enterococci showed bile deconjugation and mucin degradation abilities. A total of 17 enterococcal isolates (26.6%) that did not harbor any antibiotic resistance or virulence factor were considered safe for application as probiotics, including 6 isolates (35.3%) that showed antimicrobial activity against at least 1 fish pathogen and harbored at least 1 bacteriocin-encoding gene. Rainbow trout, feed, and rearing environment constitute an appropriate source for the isolation of enterococci as potential probiotic for aquaculture.

© Copyright 2015, Mary Ann Liebert, Inc.

Institution

(Araujo, Munoz-Atienza, Hernandez, Herranz, Cintas) Grupo de Seguridad y Calidad de Los Alimentos Por Bacterias Lacticas, Bacteriocinas y Probioticos (Grupo SEGABALBP), Universidad Complutense de Madrid, Madrid, Spain (Araujo, Igrejas) Institute for Biotechnology and Bioengineering, Centre of Genetics and Biotechnology, University of Tras-os-Montes and Alto Douro, Vila Real, Portugal

(Araujo, Igrejas) Department of Genetics and Biotechnology, University of Tras-os-Montes and Alto Douro, Vila Real, Portugal

(Araujo, Poeta) Animal and Veterinary Research Centre (CECAV), University of Tras-os-Montes and Alto Douro, Vila Real, Portugal

(Araujo, Poeta) Veterinary Science Department, University of Tras-os-Montes and Alto Douro, Vila Real 5000-801, Portugal

Publisher

Mary Ann Liebert Inc. (E-mail: info@liebertpub.com)

Emtree Heading

Aeromonas salmonicida; animal experiment; animal model; antibiotic resistance; antibiotic sensitivity; antimicrobial activity; aquaculture; aquatic environment; article; bacterial virulence; bacterium isolation; Carnobacterium maltaromaticum; clinical evaluation; controlled study; disk diffusion; *Enterococcus; Enterococcus faecium; Enterococcus hirae; enzyme degradation; *feeding behavior; fish; gene sequence; in vitro study; intestine; Lactococcus garvieae; Lactococcus lactis; nonhuman; pathogenesis; *pathogenicity; phenotypic variation; polymerase chain reaction; priority journal; *rainbow trout; *rearing; risk assessment; species identification; Streptococcus iniae; Yersinia ruckeri; ampicillin; bacteriocin; bile salt; chloramphenicol; ciprofloxacin; dalfopristin plus quinupristin; erythromycin; gelatinase; gentamicin; hemolysin; kanamycin; mucin; probiotic agent; streptomycin; superoxide dismutase/ec [Endogenous Compound]; teicoplanin; tetracycline; vancomycin; virulence factor.

Drug Index Terms

ampicillin; bacteriocin; bile salt; chloramphenicol; ciprofloxacin; dalfopristin plus quinupristin; erythromycin; gelatinase; gentamicin; hemolysin; kanamycin; mucin; probiotic agent; streptomycin; superoxide dismutase / endogenous compound; teicoplanin; tetracycline; vancomycin; virulence factor.

Other Index Terms

Aeromonas salmonicida; animal experiment; animal model; antibiotic resistance; antibiotic sensitivity; antimicrobial activity; aquaculture; aquatic environment; Article; bacterial virulence; bacterium isolation; Carnobacterium maltaromaticum; clinical evaluation; controlled study; disk diffusion; *Enterococcus; Enterococcus faecium; Enterococcus hirae; enzyme degradation; *feeding behavior; fish; gene sequence; in vitro study; intestine; Lactococcus garvieae; Lactococcus lactis; nonhuman; pathogenesis; *pathogenicity; phenotypic variation; polymerase chain reaction; priority journal; *rainbow trout; *rearing; risk assessment; species identification; Streptococcus iniae; Yersinia ruckeri.

Link to the Ovid Full Text or citation:

[Click here for full text options](https://libaccess.mcmaster.ca/login?url=http://ovidsp.ovid.com/ovidweb.cgi?T=JS&CSC=Y&NEWS=N&PAGE=fulltext&D=emed16&AN=603610000)

Link to the External Link Resolver:

[SFX](http://sfx.scholarsportal.info/mcmaster?sid=OVID:embase&id=pmid:&id=doi:10.1089%2Ffpd.2014.1906&issn=1535-3141&isbn=&volume=12&issue=4&spage=311&pages=311-322&date=2015&title=Foodborne+Pathogens+and+Disease&atitle=Evaluation+of+enterococcus+spp.+from+rainbow+trout+(Oncorhynchus+mykiss%2C+Walbaum)%2C+feed%2C+and+rearing+environment+against+fish+pathogens&aulast=Araujo&pid=<author>Araujo+C.%3BMunoz-Atienza+E.%3BHernandez+P.E.%3BHerranz+C.%3BCintas+L.M.%3BIgrejas+G.%3BPoeta+P.<%2Fauthor><AN>603610000<%2FAN><DT>Article<%2FDT>)

56.

Microbiological changes, shelf life and identification of initial and spoilage microbiota of sea bream fillets stored under various conditions using 16S rRNA gene analysis.

Parlapani F.F., Kormas K.A., Boziaris I.S.

Journal of the science of food and agriculture. 95 (12) (pp 2386-2394), 2015. Date of Publication: 01 Sep 2015.

AN: 611472247

BACKGROUND: Sea bream fillets are one of the most important value-added products of the seafood market. Fresh seafood spoils mainly owing to bacterial action. In this study an exploration of initial and spoilage microbiota of sea bream fillets stored under air and commercial modified atmosphere packaging (MAP) at 0 and 5 degreeC was conducted by 16S rRNA gene sequence analysis of isolates grown on plates. Sensory evaluation and enumeration of total viable counts and spoilage microorganisms were also conducted to determine shelf life and bacterial growth respectively. RESULTS: Different temperatures and atmospheres affected growth and synthesis of spoilage microbiota as well as shelf life. Shelf life under air at 0 and 5 degreeC was 14 and 5 days respectively, while under MAP it was 20 and 8 days respectively. Initial microbiota were dominated by Pseudomonas fluorescens, Psychrobacter and Macrococcus caseolyticus. Different temperatures and atmospheres affected the synthesis of spoilage microbiota. At the end of shelf life, different phylotypes of Pseudomonas closely related to Pseudomonas fragi were found to dominate in most cases, while Pseudomonas veronii dominated in fillets under MAP at 0 degreeC. Furthermore, in fillets under MAP at 5 degreeC, new dominant species such as Carnobacterium maltaromaticum, Carnobacterium divergens and Vagococcus fluvialis were revealed.

CONCLUSION: Different temperature and atmospheric conditions affected bacterial growth, shelf life and the synthesis of spoilage microbiota. Molecular identification revealed species and strains of microorganisms that have not been reported before for sea bream fillets stored under various conditions, thus providing valuable information regarding microbiological spoilage.

Copyright © 2014 Society of Chemical Industry.

PMID

25312872 [<http://www.ncbi.nlm.nih.gov/pubmed/?term=25312872>]

Institution

(Parlapani) Department of Ichthyology and Aquatic Environment, School of Agricultural Sciences, University of Thessaly, Fitokou Street, GR-38446 N. Ionia, Volos, Greece (Kormas) Department of Ichthyology and Aquatic Environment, School of Agricultural Sciences, University of Thessaly, Fitokou Street, GR-38446 N. Ionia, Volos, Greece

(Boziaris) Department of Ichthyology and Aquatic Environment, School of Agricultural Sciences, University of Thessaly, Fitokou Street, GR-38446 N. Ionia, Volos, Greece

Emtree Heading

animal; bacterial count; bacterium; *food control; *food preservation; genetics; human; isolation and purification; *microbiology; phylogeny; *sea bream; sea food; temperature; bacterial DNA; RNA 16S.

Drug Index Terms

bacterial DNA; RNA 16S.

Other Index Terms

animal; bacterial count; bacterium; *food control; *food preservation; genetics; human; isolation and purification; *microbiology; phylogeny; *sea bream; sea food; temperature.

Link to the Ovid Full Text or citation:

[Click here for full text options](https://libaccess.mcmaster.ca/login?url=http://ovidsp.ovid.com/ovidweb.cgi?T=JS&CSC=Y&NEWS=N&PAGE=fulltext&D=emed16&AN=611472247)

Link to the External Link Resolver:

[SFX](http://sfx.scholarsportal.info/mcmaster?sid=OVID:embase&id=pmid:25312872&id=doi:10.1002%2Fjsfa.6957&issn=1097-0010&isbn=&volume=95&issue=12&spage=2386&pages=2386-2394&date=2015&title=Journal+of+the+science+of+food+and+agriculture&atitle=Microbiological+changes%2C+shelf+life+and+identification+of+initial+and+spoilage+microbiota+of+sea+bream+fillets+stored+under+various+conditions+using+16S+rRNA+gene+analysis&aulast=Parlapani&pid=<author>Parlapani+F.F.%3BKormas+K.A.%3BBoziaris+I.S.<%2Fauthor><AN>611472247<%2FAN><DT>Article<%2FDT>)

57.

Lactic acid bacteria and their controversial role in fresh meat spoilage.

Pothakos V., Devlieghere F., Villani F., Bjorkroth J., Ercolini D.

Meat science. 109 (pp 66-74), 2015. Date of Publication: 01 Nov 2015.

AN: 609990036

Lactic acid bacteria (LAB) constitute a heterogeneous group that has been widely associated with fresh meat and cooked meat products. They represent a controversial cohort of microbial species that either contribute to spoilage through generation of offensive metabolites and the subsequent organoleptic downgrading of meat or serve as bioprotective agents with strains of certain species causing unperceivable or no alterations. Therefore, significant distinction among biotypes is substantiated by studies determining spoilage potential as a strain-specific trait corroborating the need to revisit the concept of spoilage.

Copyright © 2015 Elsevier Ltd. All rights reserved.

PMID

25972087 [<http://www.ncbi.nlm.nih.gov/pubmed/?term=25972087>]

Institution

(Pothakos) Laboratory of Food Microbiology and Food Preservation, Department of Food Safety and Food Quality, Member of Food2Know, Faculty of Bioscience Engineering, Ghent University, Ghent, Belgium (Devlieghere) Laboratory of Food Microbiology and Food Preservation, Department of Food Safety and Food Quality, Member of Food2Know, Faculty of Bioscience Engineering, Ghent University, Ghent, Belgium

(Villani) Department of Agricultural Sciences, Division of Microbiology, University of Naples Federico II, Portici, Italy

(Bjorkroth) Department of Food Hygiene and Environmental Health, University of Helsinki, Helsinki, Finland

(Ercolini) Department of Agricultural Sciences, Division of Microbiology, University of Naples Federico II, Portici, Italy. Electronic address: ercolini@unina.it

Emtree Heading

animal; *Carnobacterium; *food control; food packaging; food storage; human; *Lactobacillus; *Leuconostoc; meat; *microbiology; lactic acid.

Drug Index Terms

lactic acid.

Other Index Terms

animal; *Carnobacterium; *food control; food packaging; food storage; human; *Lactobacillus; *Leuconostoc; meat; *microbiology.

Link to the Ovid Full Text or citation:

[Click here for full text options](https://libaccess.mcmaster.ca/login?url=http://ovidsp.ovid.com/ovidweb.cgi?T=JS&CSC=Y&NEWS=N&PAGE=fulltext&D=emed16&AN=609990036)

Link to the External Link Resolver:

[SFX](http://sfx.scholarsportal.info/mcmaster?sid=OVID:embase&id=pmid:25972087&id=doi:10.1016%2Fj.meatsci.2015.04.014&issn=1873-4138&isbn=&volume=109&issue=&spage=66&pages=66-74&date=2015&title=Meat+science&atitle=Lactic+acid+bacteria+and+their+controversial+role+in+fresh+meat+spoilage&aulast=Pothakos&pid=<author>Pothakos+V.%3BDevlieghere+F.%3BVillani+F.%3BBjorkroth+J.%3BErcolini+D.<%2Fauthor><AN>609990036<%2FAN><DT>Article<%2FDT>)

58.

Associations between bacterial communities of house dust and infant gut.

Konya T., Koster B., Maughan H., Escobar M., Azad M.B., Guttman D.S., Sears M.R., Becker A.B., Brook J.R., Takaro T.K., Kozyrskyj A.L., Scott J.A., Allen R., Befus D., Brauer M., Cyr M., Chen E., Daley D., Dell S., Denburg J., Elliott S., Grasemann H., HayGlass K., Hegele R., Holness L., Kobor M., Kollmann T., Laprise C., Larche M., Lou W., Macri J., Mandhane P., Miller G., Moqbel R., Moraes T., Pare P., Ramsey C., Ratjen F., Ritchie B., Sandford A., Scott J., Silverman F., Subbarao P., Tebbutt S., Tang P., To T., Turvey S.

Environmental Research. 131 (pp 25-30), 2014. Date of Publication: May 2014.

AN: 372638513

The human gut is host to a diverse and abundant community of bacteria that influence health and disease susceptibility. This community develops in infancy, and its composition is strongly influenced by environmental factors, notably perinatal anthropogenic exposures such as delivery mode (Cesarean vs. vaginal) and feeding method (breast vs. formula); however, the built environment as a possible source of exposure has not been considered. Here we report on a preliminary investigation of the associations between bacteria in house dust and the nascent fecal microbiota from 20 subjects from the Canadian Healthy Infant Longitudinal Development (CHILD) Study using high-throughput sequence analysis of portions of the 16S rRNA gene. Despite significant differences between the dust and fecal microbiota revealed by Nonmetric Multidimensional Scaling (NMDS) analysis, permutation analysis confirmed that 14 bacterial OTUs representing the classes Actinobacteria (3), Bacilli (3), Clostridia (6) and Gammaproteobacteria (2) co-occurred at a significantly higher frequency in matched dust-stool pairs than in randomly permuted pairs, indicating an association between these dust and stool communities. These associations could indicate a role for the indoor environment in shaping the nascent gut microbiota, but future studies will be needed to confirm that our findings do not solely reflect a reverse pathway. Although pet ownership was strongly associated with the presence of certain genera in the dust for dogs (Agrococcus, Carnobacterium, Exiguobacterium, Herbaspirillum, Leifsonia and Neisseria) and cats (Escherichia), no clear patterns were observed in the NMDS-resolved stool community profiles as a function of pet ownership. © 2014 Elsevier Inc.

PMID

24637181 [<http://www.ncbi.nlm.nih.gov/pubmed/?term=24637181>]

Institution

(Konya, Koster, Escobar, Brook, Scott) Division of Occupational and Environmental Health, Dalla Lana School of Public Health, University of Toronto, Canada (Maughan, Guttman) Department of Cell and Systems Biology, University of Toronto, Canada

(Azad, Kozyrskyj) Department of Pediatrics, University of Alberta, Canada

(Sears) Department of Medicine, McMaster University, Canada

(Becker, HayGlass, Moqbel, Ramsey) University of Manitoba, Canada

(Brook) Environment Canada, Canada

(Takaro) Faculty of Health Science, Simon Fraser University, Canada

(Allen) Simon Fraser University, Canada

(Befus, Mandhane, Ritchie) University of Alberta, Canada

(Brauer, Chen, Kobor, Kollmann, Pare, Tang, Turvey) University of British Columbia, Canada

(Cyr, Denburg, Larche, Macri) McMaster University, Canada

(Daley, Sandford, Tebbutt) James Hogg iCAPTURE Centre, Canada

(Dell, Grasemann, Moraes, Ratjen, Subbarao, To) Hospital for Sick Children, Canada

(Elliott) University of Waterloo, Canada

(Hegele, Lou, Silverman) University of Toronto, Canada

(Holness) St. Michael's Hospital, Canada

(Laprise) Chicoutimi University Hospital, Canada

(Miller) North Western University, United States

(Scott) Lakehead University, Canada

Publisher

Academic Press Inc. (1250 Sixth Avenue, San Diego, California CA 92101, United States)

Emtree Heading

Acidaminococcaceae; Actinobacteria; article; Carnobacterium; cat; controlled study; dog; Escherichia; Faecalibacterium; feces microflora; Herbaspirillum; high throughput sequencing; *house dust; human; infant; *intestine flora; Lachnospiraceae; *microbial community; multidimensional scaling; Neisseria; nonhuman; normal human; nucleotide sequence; Peptostreptococcaceae; Planococcaceae; priority journal; Streptococcus; Veillonella; DNA 16S/ec [Endogenous Compound]; RNA 16S/ec [Endogenous Compound]; Agrococcus; Exiguobacterium; Leifsonia.

Candidate Terms

Agrococcus [other term]; Exiguobacterium [other term]; Leifsonia [other term].

Drug Index Terms

DNA 16S / endogenous compound; RNA 16S / endogenous compound.

Other Index Terms

Acidaminococcaceae; Actinobacteria; article; Carnobacterium; cat; controlled study; dog; Escherichia; Faecalibacterium; feces microflora; Herbaspirillum; high throughput sequencing; *house dust; human; infant; *intestine flora; Lachnospiraceae; *microbial community; multidimensional scaling; Neisseria; nonhuman; normal human; nucleotide sequence; Peptostreptococcaceae; Planococcaceae; priority journal; Streptococcus; Veillonella.

Link to the Ovid Full Text or citation:

[Click here for full text options](https://libaccess.mcmaster.ca/login?url=http://ovidsp.ovid.com/ovidweb.cgi?T=JS&CSC=Y&NEWS=N&PAGE=fulltext&D=emed15&AN=372638513)

Link to the External Link Resolver:

[SFX](http://sfx.scholarsportal.info/mcmaster?sid=OVID:embase&id=pmid:24637181&id=doi:10.1016%2Fj.envres.2014.02.005&issn=0013-9351&isbn=&volume=131&issue=&spage=25&pages=25-30&date=2014&title=Environmental+Research&atitle=Associations+between+bacterial+communities+of+house+dust+and+infant+gut&aulast=Konya&pid=<author>Konya+T.%3BKoster+B.%3BMaughan+H.%3BEscobar+M.%3BAzad+M.B.%3BGuttman+D.S.%3BSears+M.R.%3BBecker+A.B.%3BBrook+J.R.%3BTakaro+T.K.%3BKozyrskyj+A.L.%3BScott+J.A.%3BAllen+R.%3BBefus+D.%3BBrauer+M.%3BCyr+M.%3BChen+E.%3BDaley+D.%3BDell+S.%3BDenburg+J.%3BElliott+S.%3BGrasemann+H.%3BHayGlass+K.%3BHegele+R.%3BHolness+L.%3BKobor+M.%3BKollmann+T.%3BLaprise+C.%3BLarche+M.%3BLou+W.%3BMacri+J.%3BMandhane+P.%3BMiller+G.%3BMoqbel+R.%3BMoraes+T.%3BPare+P.%3BRamsey+C.%3BRatjen+F.%3BRitchie+B.%3BSandford+A.%3BScott+J.%3BSilverman+F.%3BSubbarao+P.%3BTebbutt+S.%3BTang+P.%3BTo+T.%3BTurvey+S.<%2Fauthor><AN>372638513<%2FAN><DT>Article<%2FDT>)

59.

Evaluation of the spoilage potential of bacteria isolated from spoiled cooked whole tropical shrimp (Penaeus vannamei) stored under modified atmosphere packaging.

Mace S., Cardinal M., Jaffres E., Cornet J., Lalanne V., Chevalier F., Serot T., Pilet M.-F., Dousset X., Joffraud J.-J.

Food Microbiology. 40 (pp 9-17), 2014. Date of Publication: June 2014.

AN: 1370561273

The spoilage potential of isolates belonging to five bacterial groups/species (. Shewanella baltica, Carnobacterium maltaromaticum, Aeromonas salmonicida, Vibrio sp., "other Gamma-Proteobacteria" [containing one strain of Pseudoalteromonas sp. and one strain of Psychrobacter sp.]) isolated from spoiled cooked and whole tropical shrimp stored under modified atmosphere packaging (MAP) was evaluated by inoculation into ionized cooked and peeled tropical shrimp followed by storage for 32 days at 8degreeC. Microbial growth and sensory changes were monitored during the storage period. The major spoilage bacterial isolate groups were C.maltaromaticum and S.baltica. In order to characterize their spoilage potential further and to study the effect of their interactions, each of these two specific spoilage organisms (SSO) and one mixed-culture, C.maltaromaticum/. S.baltica, were tested using a combination of complementary methods: molecular (PCR-TTGE), sensory, chemical, and conventional microbiological analyses. It was concluded that, in the mixed-culture-inoculated samples, both species groups imposed their spoilage characteristics. © 2013 Elsevier Ltd.

PMID

24549192 [<http://www.ncbi.nlm.nih.gov/pubmed/?term=24549192>]

Institution

(Mace, Jaffres, Pilet, Dousset) LUNAM Universite, ONIRIS, Universite Nantes, UMR1014 Secalim, Nantes F-44307, France (Mace, Jaffres, Pilet, Dousset) INRA, Nantes F-44307, France

(Mace, Cardinal, Cornet, Chevalier, Joffraud) Ifremer, Laboratoire Science et Technologie de la Biomasse Marine, BP 21105, 44311 Nantes Cedex 3, France

(Lalanne, Serot) LUNAM Universite, ONIRIS, Universite Nantes, UMR 6144 GEPEA, Flavor Unit, F-44307, France

(Lalanne, Serot) CNRS, Nantes F-44307, France

Emtree Heading

animal; article; *bacterium; chemistry; classification; cooking; evaluation study; *food packaging; food storage; genetics; growth, development and aging; human; isolation and purification; methodology; microbiology; *Penaeidae; *shellfish/an [Drug Analysis]; taste; bacterial interaction; Cooked tropical shrimp; Specific spoilage organism; Spoilage potential; volatile compounds.

Candidate Terms

Bacterial interaction [other term]; Cooked tropical shrimp [other term]; Specific spoilage organism [other term]; Spoilage potential [other term]; Volatile compounds [other term].

Other Index Terms

animal; article; *bacterium; chemistry; classification; cooking; evaluation study; *food packaging; food storage; genetics; growth, development and aging; human; isolation and purification; methodology; microbiology; *Penaeidae; *shellfish / *drug analysis; taste.

Link to the Ovid Full Text or citation:

[Click here for full text options](https://libaccess.mcmaster.ca/login?url=http://ovidsp.ovid.com/ovidweb.cgi?T=JS&CSC=Y&NEWS=N&PAGE=fulltext&D=emed15&AN=1370561273)

Link to the External Link Resolver:

[SFX](http://sfx.scholarsportal.info/mcmaster?sid=OVID:embase&id=pmid:24549192&id=doi:10.1016%2Fj.fm.2013.11.018&issn=0740-0020&isbn=&volume=40&issue=&spage=9&pages=9-17&date=2014&title=Food+Microbiology&atitle=Evaluation+of+the+spoilage+potential+of+bacteria+isolated+from+spoiled+cooked+whole+tropical+shrimp+(Penaeus+vannamei)+stored+under+modified+atmosphere+packaging&aulast=Mace&pid=<author>Mace+S.%3BCardinal+M.%3BJaffres+E.%3BCornet+J.%3BLalanne+V.%3BChevalier+F.%3BSerot+T.%3BPilet+M.-F.%3BDousset+X.%3BJoffraud+J.-J.<%2Fauthor><AN>1370561273<%2FAN><DT>Article<%2FDT>)

60.

The shelf life of farmed turbot (Scophthalmus maximus).

Roth B., Kramer L., Skuland A.V., Lovdal T., Oines S., Foss A., Imsland A.K.

Journal of food science. 79 (8) (pp S1568-S1574), 2014. Date of Publication: 01 Aug 2014.

AN: 604453985

A total of 18 farmed turbot (Scophthalmus maximus) were slaughtered over 4 successive weeks in November 2012 and stored in polystyrene boxes with ice until analyzed. The fish were stored between 1 and 22 d and presented to a taste panel and further analyzed for quality index method (QIM), microbiological analysis by real-time quantitative PCR (qPCR), taste, pH, color by computer imaging, protein denaturation with differential scanner calorimeter (DSC), texture hardness, and shear force. Results show small, but significant changes in physical and visual attributes such as texture and color. No gaping was observed. Only small changes in texture were observed explained by lack of myosin denaturation. The fillets became more white and yellow during storage, whereas the major changes occurred during the 1st week. A panel evaluating QIM and taste could not distinguish major differences in appearance and taste and over 15 d storage period, but were able to quantify the age by smell. Analysis of microorganisms on the epidermis displayed growth of Carnobacterium maltaromaticum, potentially inhibiting growth of other spoilage bacteria. Fish stored for 22 d were rejected by the taste panel caused by a stale smell and taste, but not bitter or rancid. It is concluded that turbot has a shelf life of at least 16 d.

Copyright © 2014 Institute of Food Technologists

PMID

25046980 [<http://www.ncbi.nlm.nih.gov/pubmed/?term=25046980>]

Institution

(Roth, Kramer, Skuland, Lovdal, Oines, Foss, Imsland) Nofima Dept. of Processing Technology, P.O. Box 8034, N-4068 Stavanger, Norway

Emtree Heading

analysis; animal; Carnobacterium; chemistry; color; cooking; differential scanning calorimetry; DNA sequence; flatfish; food contamination; food control; *food storage; genetics; growth, development and aging; hardness; human; *isolation and purification; Lactobacillus; *microbiology; pH; Photobacterium; sea food; skeletal muscle; statistical model; taste; bacterial DNA.

Drug Index Terms

bacterial DNA.

Other Index Terms

analysis; animal; Carnobacterium; chemistry; color; cooking; differential scanning calorimetry; DNA sequence; flatfish; food contamination; food control; *food storage; genetics; growth, development and aging; hardness; human; *isolation and purification; Lactobacillus; *microbiology; pH; Photobacterium; sea food; skeletal muscle; statistical model; taste.

Link to the Ovid Full Text or citation:

[Click here for full text options](https://libaccess.mcmaster.ca/login?url=http://ovidsp.ovid.com/ovidweb.cgi?T=JS&CSC=Y&NEWS=N&PAGE=fulltext&D=emed15&AN=604453985)

Link to the External Link Resolver:

[SFX](http://sfx.scholarsportal.info/mcmaster?sid=OVID:embase&id=pmid:25046980&id=doi:10.1111%2F1750-3841.12541&issn=1750-3841&isbn=&volume=79&issue=8&spage=S1568&pages=S1568-S1574&date=2014&title=Journal+of+food+science&atitle=The+shelf+life+of+farmed+turbot+(Scophthalmus+maximus)&aulast=Roth&pid=<author>Roth+B.%3BKramer+L.%3BSkuland+A.V.%3BLovdal+T.%3BOines+S.%3BFoss+A.%3BImsland+A.K.<%2Fauthor><AN>604453985<%2FAN><DT>Article<%2FDT>)

61.

Characterization of biological aerosol exposure risks from automobile air conditioning system.

Li J., Li M., Shen F., Zou Z., Yao M., Wu C.-Y.

Environmental Science and Technology. 47 (18) (pp 10660-10666), 2013. Date of Publication: 17 Sep 2013.

AN: 369832204

Although use of automobile air conditioning (AC) was shown to reduce in-vehicle particle levels, the characterization of its microbial aerosol exposure risks is lacking. Here, both AC and engine filter dust samples were collected from 30 automobiles in four different geographical locations in China. Biological contents (bacteria, fungi, and endotoxin) were studied using culturing, high-throughput gene sequence, and Limulus amebocyte lysate (LAL) methods. In-vehicle viable bioaerosol concentrations were directly monitored using an ultraviolet aerodynamic particle sizer (UVAPS) before and after use of AC for 5, 10, and 15 min. Regardless of locations, the vehicle AC filter dusts were found to be laden with high levels of bacteria (up to 26 150 CFU/mg), fungi (up to 1287 CFU/mg), and endotoxin (up to 5527 EU/mg). More than 400 unique bacterial species, including human opportunistic pathogens, were detected in the filter dusts. In addition, allergenic fungal species were also found abundant. Surprisingly, unexpected fluorescent peaks around 2.5 mum were observed during the first 5 min use of AC, which was attributed to the reaerosolization of those filter-borne microbial agents. The information obtained here can assist in minimizing or preventing the respiratory allergy or infection risk from the use of automobile AC system. © 2013 American Chemical Society.

PMID

23952908 [<http://www.ncbi.nlm.nih.gov/pubmed/?term=23952908>]

Institution

(Li, Li, Shen, Zou, Yao) State Key Joint Laboratory of Environmental Simulation and Pollution Control, College of Environmental Sciences and Engineering, Peking University, Beijing 100871, China (Wu) Department of Environmental Engineering Sciences, University of Florida, Gainesville, FL 32611, United States

Publisher

American Chemical Society (2540 Olentangy River Road, P.O. Box 3337, Columbus OH 43210-3337, United States)

Emtree Heading

Acinetobacter; Actinobacteria; aerosol; *air conditioning; Alternaria alternata; Arthrobacter; article; Aspergillus; Aspergillus niger; Aspergillus ochraceus; Aspergillus oryzae; Aspergillus sydowii; Aspergillus ustus; Aspergillus versicolor; Bacillus; bacterium culture; car; Carnobacterium; China; colony forming unit; Curvularia lunata; dust; filter; Firmicutes; fungus culture; gene sequence; Micrococcus; nonhuman; Penicillium citrinum; Proteobacteria; Pseudomonas; Psychrobacter; risk assessment; Sporosarcina; Staphylococcus; Stenotrophomonas; Trichoderma viride; ultraviolet radiation; endotoxin.

Drug Index Terms

endotoxin.

Other Index Terms

Acinetobacter; Actinobacteria; aerosol; *air conditioning; Alternaria alternata; Arthrobacter; article; Aspergillus; Aspergillus niger; Aspergillus ochraceus; Aspergillus oryzae; Aspergillus sydowii; Aspergillus ustus; Aspergillus versicolor; Bacillus; bacterium culture; car; Carnobacterium; China; colony forming unit; Curvularia lunata; dust; filter; Firmicutes; fungus culture; gene sequence; Micrococcus; nonhuman; Penicillium citrinum; Proteobacteria; Pseudomonas; Psychrobacter; risk assessment; Sporosarcina; Staphylococcus; Stenotrophomonas; Trichoderma viride; ultraviolet radiation.

Link to the Ovid Full Text or citation:

[Click here for full text options](https://libaccess.mcmaster.ca/login?url=http://ovidsp.ovid.com/ovidweb.cgi?T=JS&CSC=Y&NEWS=N&PAGE=fulltext&D=emed14&AN=369832204)

Link to the External Link Resolver:

[SFX](http://sfx.scholarsportal.info/mcmaster?sid=OVID:embase&id=pmid:23952908&id=doi:10.1021%2Fes402848d&issn=0013-936X&isbn=&volume=47&issue=18&spage=10660&pages=10660-10666&date=2013&title=Environmental+Science+and+Technology&atitle=Characterization+of+biological+aerosol+exposure+risks+from+automobile+air+conditioning+system&aulast=Li&pid=<author>Li+J.%3BLi+M.%3BShen+F.%3BZou+Z.%3BYao+M.%3BWu+C.-Y.<%2Fauthor><AN>369832204<%2FAN><DT>Article<%2FDT>)

62.

Growth of Carnobacterium spp. from permafrost under low pressure, temperature, and anoxic atmosphere has implications for Earth microbes on Mars.

Nicholson W.L., Krivushin K., Gilichinsky D., Schuerger A.C.

Proceedings of the National Academy of Sciences of the United States of America. 110 (2) (pp 666-671), 2013. Date of Publication: 08 Jan 2013.

AN: 368091443

The ability of terrestrial microorganisms to grow in the near-surface environment of Mars is of importance to the search for life and protection of that planet from forward contamination by human and robotic exploration. Because most water on present-day Mars is frozen in the regolith, permafrosts are considered to be terrestrial analogs of the martian subsurface environment. Six bacterial isolates were obtained from a permafrost borehole in northeastern Siberia capable of growth under conditions of low temperature (0 degreeC), low pressure (7 mbar), and a CO2-enriched anoxic atmosphere. By 16S ribosomal DNA analysis, all six permafrost isolates were identified as species of the genus Carnobacterium, most closely related to C. inhibens (five isolates) and C. viridans (one isolate). Quantitative growth assays demonstrated that the six permafrost isolates, as well as nine type species of Carnobacterium (C. alterfunditum , C. divergens, C. funditum, C. gallinarum, C. inhibens, C. maltaromaticum, C. mobile, C. pleistocenium, and C. viridans) were all capable of growth under cold, low-pressure, anoxic conditions, thus extending the low-pressure extreme at which life can function.

PMID

23267097 [<http://www.ncbi.nlm.nih.gov/pubmed/?term=23267097>]

Institution

(Nicholson) Department of Microbiology and Cell Science, University of Florida, Merritt Island, FL 32953, United States (Krivushin, Gilichinsky) Institute of Physicochemical and Biological Problems in Soil Science, Russian Academy of Sciences, Pushchino 142290 Moscow, Russian Federation

(Schuerger) Department of Plant Pathology, Space Life Sciences Laboratory, University of Florida, Merritt Island, FL 32953, United States

Publisher

National Academy of Sciences (2101 Constitution Avenue NW, Washington DC 20418, United States)

Emtree Heading

article; *astronomy; atmosphere; *bacterial growth; bacterial strain; bacterium identification; bacterium isolate; *Carnobacterium; hypobarism; microorganism; nonhuman; nucleotide sequence; *permafrost; phylogenetic tree; priority journal; temperature; bacterial DNA; DNA 16S; anoxic atmosphere.

Candidate Terms

anoxic atmosphere [other term].

Drug Index Terms

bacterial DNA; DNA 16S.

Other Index Terms

article; *astronomy; atmosphere; *bacterial growth; bacterial strain; bacterium identification; bacterium isolate; *Carnobacterium; hypobarism; microorganism; nonhuman; nucleotide sequence; *permafrost; phylogenetic tree; priority journal; temperature.

Link to the Ovid Full Text or citation:

[Click here for full text options](https://libaccess.mcmaster.ca/login?url=http://ovidsp.ovid.com/ovidweb.cgi?T=JS&CSC=Y&NEWS=N&PAGE=fulltext&D=emed14&AN=368091443)

Link to the External Link Resolver:

[SFX](http://sfx.scholarsportal.info/mcmaster?sid=OVID:embase&id=pmid:23267097&id=doi:10.1073%2Fpnas.1209793110&issn=0027-8424&isbn=&volume=110&issue=2&spage=666&pages=666-671&date=2013&title=Proceedings+of+the+National+Academy+of+Sciences+of+the+United+States+of+America&atitle=Growth+of+Carnobacterium+spp.+from+permafrost+under+low+pressure%2C+temperature%2C+and+anoxic+atmosphere+has+implications+for+Earth+microbes+on+Mars&aulast=Nicholson&pid=<author>Nicholson+W.L.%3BKrivushin+K.%3BGilichinsky+D.%3BSchuerger+A.C.<%2Fauthor><AN>368091443<%2FAN><DT>Article<%2FDT>)

63.

Investigation of spoilage in saveloy samples inoculated with four potential spoilage bacteria.

Holm E.S., Schafer A., Koch A.G., Petersen M.A.

Meat science. 93 (3) (pp 687-695), 2013. Date of Publication: Mar 2013.

AN: 366409938

Sliced saveloy samples were inoculated with monocultures of four potential spoilage bacteria and studied during a four week storage period. The objective was to investigate the resulting changes in the composition of Volatile Organic Compounds (VOCs) and the sensory quality of the product. Based on the sensory scores and the VOC composition Brochothrix thermosphacta, Chryseomonas luteola and Carnobacterium maltaromaticum were found to have a high spoilage potential in saveloy samples subjected to consumer simulated storage during the fourth week. Inoculation with Leuconostoc carnosum only resulted in a low level of spoilage. The sensory changes in the saveloy samples were modeled based on the VOC composition using Partial Least Squares Regression. The changes in the six sensory descriptors were closely related to the amount of diacetyl, acetoin, 2- and 3-methylbutanol, 2- and 3-methylbutanal and 2-methylpropanol found in the samples. These compounds are therefore potentially important for the shelf-life of sliced saveloy. Copyright © 2012 Elsevier Ltd. All rights reserved.

PMID

23261532 [<http://www.ncbi.nlm.nih.gov/pubmed/?term=23261532>]

Institution

(Holm) Department of Food Science, Quality & Technology, Faculty of Science, University of Copenhagen, Rolighedsvej 30, 1958 Frederiksberg C, Denmark.

Emtree Heading

animal; article; *bacterium; *food control; food storage; human; *meat; metabolism; microbiology; *odor; regression analysis; *volatile organic compound.

Drug Index Terms

*volatile organic compound.

Other Index Terms

animal; article; *bacterium; *food control; food storage; human; *meat; metabolism; microbiology; *odor; regression analysis.

Link to the Ovid Full Text or citation:

[Click here for full text options](https://libaccess.mcmaster.ca/login?url=http://ovidsp.ovid.com/ovidweb.cgi?T=JS&CSC=Y&NEWS=N&PAGE=fulltext&D=emed14&AN=366409938)

Link to the External Link Resolver:

[SFX](http://sfx.scholarsportal.info/mcmaster?sid=OVID:embase&id=pmid:23261532&id=doi:&issn=1873-4138&isbn=&volume=93&issue=3&spage=687&pages=687-695&date=2013&title=Meat+science&atitle=Investigation+of+spoilage+in+saveloy+samples+inoculated+with+four+potential+spoilage+bacteria&aulast=Holm&pid=<author>Holm+E.S.%3BSchafer+A.%3BKoch+A.G.%3BPetersen+M.A.<%2Fauthor><AN>366409938<%2FAN><DT>Article<%2FDT>)

64.

Phylogenetic analysis of antimicrobial lactic acid bacteria from farmed seabass Dicentrarchus labrax.

Bourouni O.C., El Bour M., Calo-Mata P., Mraouna R., Abedellatif B., Barros-Velazquez J.

Canadian Journal of Microbiology. 58 (4) (pp 463-474), 2012. Date of Publication: April 2012.

AN: 364568954

The use of lactic acid bacteria (LAB) in the prevention or reduction of fish diseases is receiving increasing attention. In the present study, 47 LAB strains were isolated from farmed seabass (Dicentrarchus labrax) and were phenotypically and phylogenetically analysed by 16S rDNA and randomly amplified polymorphic DNA -polymerase chain reaction (RAPD-PCR). Their antimicrobial effect was tested in vitro against a wide variety of pathogenic and spoilage bacteria. Most of the strains isolated were enterococci belonging to the following species: Enterococcus faecium (59%), Enterococcus faecalis (21%), Enterococcus sanguinicola (4 strains), Enterococcus mundtii (1 strain), Enterococcus pseudoavium (1 strain), and Lactococcus lactis (1 strain). An Aerococcus viridans strain was also isolated. The survey of their antimicrobial susceptibility showed that all isolates were sensitive to vancomycin and exhibited resistance to between 4 and 10 other antibiotics relevant for therapy in human and animal medicine. Different patterns of resistance were noted for skin and intestines isolates. More than 69% (32 strains) of the isolates inhibited the growth of the majority of pathogenic and spoilage bacteria tested, including Listeria monocytogenes, Staphylococcus aureus, Aeromonas hydrophila, Aeromonas salmonicida, Vibrio anguillarum, and Carnobacterium sp. To our knowledge, this is the first report of bioactive enterococcal species isolated from seabass that could potentially inhibit the undesirable bacteria found in food systems.

PMID

22439634 [<http://www.ncbi.nlm.nih.gov/pubmed/?term=22439634>]

Institution

(Bourouni, El Bour, Mraouna) Institut national des sciences et technologies de la mer (INSTM), rue 2 Mars 1934, 2025 Salammbo Tunis, Tunisia (Calo-Mata, Barros-Velazquez) Department of Analytical Chemistry, Nutrition and Food Science, LHICA, School of Veterinary Sciences, University of Santiago de Compostela, E-27002 Lugo, Spain

(Abedellatif) Laboratoire de microorganismes et biomolecules actives, Faculte des Sciences, Tunis, Tunisia

Publisher

National Research Council of Canada (Buiding M 55, Ottawa ONT K1A 0R6, Canada)

Emtree Heading

Aeromonas hydrophila; Aeromonas salmonicida; antibacterial activity; antibiotic resistance; antibiotic sensitivity; article; bacterium isolation; Carnobacterium; controlled study; Enterococcus; Enterococcus faecalis; Enterococcus faecium; *European sea bass; genetic identification; in vitro study; *lactic acid bacterium; Lactococcus lactis; Listeria monocytogenes; Listonella anguillarum; nonhuman; *nucleotide sequence; phenotype; *phylogeny; polymerase chain reaction; priority journal; random amplified polymorphic DNA; cefoxitin; ceftriaxone; chloramphenicol; DNA 16S; neomycin; oleandomycin; oxacillin; penicillin G; streptomycin; tetracycline; tobramycin; vancomycin; enterococcus mundtii; enterococcus pseudoavium; Enterococcus sanguinicola.

Candidate Terms

enterococcus mundtii [other term]; Enterococcus pseudoavium [other term]; Enterococcus sanguinicola [other term].

Drug Index Terms

cefoxitin; ceftriaxone; chloramphenicol; DNA 16S; neomycin; oleandomycin; oxacillin; penicillin G; streptomycin; tetracycline; tobramycin; vancomycin.

Other Index Terms

Aeromonas hydrophila; Aeromonas salmonicida; antibacterial activity; antibiotic resistance; antibiotic sensitivity; article; bacterium isolation; Carnobacterium; controlled study; Enterococcus; Enterococcus faecalis; Enterococcus faecium; *European sea bass; genetic identification; in vitro study; *lactic acid bacterium; Lactococcus lactis; Listeria monocytogenes; Listonella anguillarum; nonhuman; *nucleotide sequence; phenotype; *phylogeny; polymerase chain reaction; priority journal; random amplified polymorphic DNA.

Link to the Ovid Full Text or citation:

[Click here for full text options](https://libaccess.mcmaster.ca/login?url=http://ovidsp.ovid.com/ovidweb.cgi?T=JS&CSC=Y&NEWS=N&PAGE=fulltext&D=emed13&AN=364568954)

Link to the External Link Resolver:

[SFX](http://sfx.scholarsportal.info/mcmaster?sid=OVID:embase&id=pmid:22439634&id=doi:10.1139%2FW2012-014&issn=0008-4166&isbn=&volume=58&issue=4&spage=463&pages=463-474&date=2012&title=Canadian+Journal+of+Microbiology&atitle=Phylogenetic+analysis+of+antimicrobial+lactic+acid+bacteria+from+farmed+seabass+Dicentrarchus+labrax&aulast=Bourouni&pid=<author>Bourouni+O.C.%3BEl+Bour+M.%3BCalo-Mata+P.%3BMraouna+R.%3BAbedellatif+B.%3BBarros-Velazquez+J.<%2Fauthor><AN>364568954<%2FAN><DT>Article<%2FDT>)

65.

High pressure inactivation of Escherichia coli, Campylobacter jejuni, and spoilage microbiota on poultry meat.

Liu Y., Betti M., Ganzle M.G.

Journal of Food Protection. 75 (3) (pp 497-503), 2012. Date of Publication: March 2012.

AN: 364405623

This study evaluated the high pressure inactivation of Campylobacter jejuni, Escherichia coli, and poultry meat spoilage organisms. All treatments were performed in aseptically prepared minced poultry meat. Treatment of 19 strains of C. jejuni at 300 MPa and 30degreeC revealed a large variation of pressure resistance. The recovery of pressure-induced sublethally injured C. jejuni depended on the availability of iron. The addition of iron content to enumeration media was required for resuscitation of sublethally injured cells. Survival of C. jejuni during storage of refrigerated poultry meat was analyzed in fresh and pressuretreated poultry meat, and in the presence or absence of spoilage microbiota. The presence of spoilage microbiota did not significantly influence the survival of C. jejuni. Pressure treatment at 400 MPa and 40degreeC reduced cell counts of Brochothrix thermosphacta, Carnobacterium divergens, C. jejuni, and Pseudomonas fluorescens to levels below the detection limit. Cell counts of E. coli AW1.7, however, were reduced by only 3.5 log (CFU/g) and remained stable during subsequent refrigerated storage. The resistance to treatment at 600 MPa and 40degreeC of E. coli AW1.7 was compared with Salmonella enterica, Shiga toxin- producing E. coli and nonpathogenic E. coli strains, and Staphylococcus spp. Cell counts of all organisms except E. coli AW 1.7 were reduced by more than 6 log CFU/g. Cell counts of E. coli AW1.7 were reduced by 4.5 log CFU/g only. Moreover, the ability of E. coli AW1.7 to resist pressure was comparable to the pressure-resistant mutant E. coli LMM1030. Our results indicate that preservation of fresh meat requires a combination of high pressure with high temperature (40 to 60degreeC) or other antimicrobial hurdles. Copyright © International Association for Food Protection.

PMID

22410223 [<http://www.ncbi.nlm.nih.gov/pubmed/?term=22410223>]

Institution

(Liu, Betti, Ganzle) Department of Agricultural, Food and Nutritional Sciences, University of Alberta, Edmonton, AB T6G 2P5, Canada

Publisher

IAMFES (6200 Aurora Avenue, Suite 200W, Des Moines IA 50322-3863, United States)

Emtree Heading

animal; article; bacterial count; *Campylobacter jejuni; *Escherichia coli; *food; *food handling; growth, development and aging; heat; human; metabolism; methodology; microbiology; *pressure; product safety; iron.

Drug Index Terms

iron.

Other Index Terms

animal; article; bacterial count; *Campylobacter jejuni; *Escherichia coli; *food; *food handling; growth, development and aging; heat; human; metabolism; methodology; microbiology; *pressure; product safety.

Link to the Ovid Full Text or citation:

[Click here for full text options](https://libaccess.mcmaster.ca/login?url=http://ovidsp.ovid.com/ovidweb.cgi?T=JS&CSC=Y&NEWS=N&PAGE=fulltext&D=emed13&AN=364405623)

Link to the External Link Resolver:

[SFX](http://sfx.scholarsportal.info/mcmaster?sid=OVID:embase&id=pmid:22410223&id=doi:10.4315%2F0362-028X.JFP-11-316&issn=0362-028X&isbn=&volume=75&issue=3&spage=497&pages=497-503&date=2012&title=Journal+of+Food+Protection&atitle=High+pressure+inactivation+of+Escherichia+coli%2C+Campylobacter+jejuni%2C+and+spoilage+microbiota+on+poultry+meat&aulast=Liu&pid=<author>Liu+Y.%3BBetti+M.%3BGanzle+M.G.<%2Fauthor><AN>364405623<%2FAN><DT>Article<%2FDT>)

66.

Carnobacterium divergens - a dominating bacterium of pork meat juice.

Rieder G., Krisch L., Fischer H., Kaufmann M., Maringer A., Wessler S.

FEMS Microbiology Letters. 332 (2) (pp 122-130), 2012. Date of Publication: July 2012.

AN: 52015207

Nonspoiled food that nevertheless contains bacterial pathogens constitutes a much more serious health problem than spoiled food, as the consumer is not warned beforehand. However, data on the diversity of bacterial species in meat juice are rare. To study the bacterial load of fresh pork from ten different distributors, we applied a combination of the conventional culture-based and molecular methods for detecting and quantifying the microbial spectrum of fresh pork meat juice samples. Altogether, we identified 23 bacterial species of ten different families analyzed by 16S rRNA gene sequencing. The majority of isolates were belonging to the typical spoilage bacterial population of lactic acid bacteria (LAB), Enterococcaceae, and Pseudomonadaceae. Several additional isolates were identified as Staphylococcus spp. and Bacillus spp. originating from human and animal skin and other environmental niches including plants, soil, and water. Carnobacterium divergens, a LAB contributing to the spoilage of raw meat even at refrigeration temperature, was the most frequently isolated species in our study (5/10) with a bacterial load of 103-107 CFU mL-1. In several of the analyzed pork meat juice samples, two bacterial faecal indicators, Serratia grimesii and Serratia proteamaculans, were identified together with another opportunistic food-borne pathogen, Staphylococcus equorum. Our data reveal a high bacterial load of fresh pork meat supporting the potential health risk of meat juice for the end consumer even under refrigerated conditions. © 2012 Federation of European Microbiological Societies. Published by Blackwell Publishing Ltd.

PMID

22537055 [<http://www.ncbi.nlm.nih.gov/pubmed/?term=22537055>]

Institution

(Rieder, Krisch, Wessler) Division of Microbiology, Department of Molecular Biology, University of Salzburg, Salzburg, Austria (Fischer, Kaufmann, Maringer) Sony DADC Austria AG, Anif, Austria

Publisher

Blackwell Publishing Ltd (9600 Garsington Road, Oxford OX4 2XG, United Kingdom)

Emtree Heading

animal tissue; Bacillus; bacterial load; *bacterium contamination; bacterium culture; bacterium detection; bacterium identification; bacterium isolation; Carnobacterium; colony forming unit; controlled study; Enterococcaceae; food analysis; *food contamination; food preservation; food quality; food spoilage; gene sequence; letter; nonhuman; nucleotide sequence; pork; priority journal; Pseudomonadaceae; Staphylococcus; temperature dependence; RNA 16S/ec [Endogenous Compound]; Carnobacterium divergens.

Candidate Terms

Carnobacterium divergens [other term].

Drug Index Terms

RNA 16S / endogenous compound.

Other Index Terms

animal tissue; Bacillus; bacterial load; *bacterium contamination; bacterium culture; bacterium detection; bacterium identification; bacterium isolation; Carnobacterium; colony forming unit; controlled study; Enterococcaceae; food analysis; *food contamination; food preservation; food quality; food spoilage; gene sequence; letter; nonhuman; nucleotide sequence; pork; priority journal; Pseudomonadaceae; Staphylococcus; temperature dependence.

Link to the Ovid Full Text or citation:

[Click here for full text options](https://libaccess.mcmaster.ca/login?url=http://ovidsp.ovid.com/ovidweb.cgi?T=JS&CSC=Y&NEWS=N&PAGE=fulltext&D=emed13&AN=52015207)

Link to the External Link Resolver:

[SFX](http://sfx.scholarsportal.info/mcmaster?sid=OVID:embase&id=pmid:22537055&id=doi:10.1111%2Fj.1574-6968.2012.02584.x&issn=0378-1097&isbn=&volume=332&issue=2&spage=122&pages=122-130&date=2012&title=FEMS+Microbiology+Letters&atitle=Carnobacterium+divergens+-+a+dominating+bacterium+of+pork+meat+juice&aulast=Rieder&pid=<author>Rieder+G.%3BKrisch+L.%3BFischer+H.%3BKaufmann+M.%3BMaringer+A.%3BWessler+S.<%2Fauthor><AN>52015207<%2FAN><DT>Letter<%2FDT>)

67.

The effect of crowding stress on bacterial growth and sensory properties of chilled Atlantic salmon fillets.

Adland Hansen A., Rodbotten M., Eie T., Lea P., Rudi K., Morkore T.

Journal of Food Science. 77 (1) (pp S84-S90), 2012. Date of Publication: January 2012.

AN: 364122839

Atlantic salmon were subjected to minimal preslaughter crowding stress (Control), short-term crowding for 20 min (SS-group), or long-term crowding for 24 h (LS-group). The fish were filleted prerigor, cut into 270 g pieces, and packaged in modified atmosphere (60% CO2 and 40% N2). Fillet quality analyses were determined during 22 d of storage at 0.3 degreeC. Bacterial growth and unpleasant sensory properties increased earlier in the LS-group. The negative effects of long-term preslaughter stress were more pronounced for raw than cooked samples, and more pronounced for odor than flavor. Sequence analyses of bacterial DNA at the end of storage revealed that 100% of the bacteria were comprised by Photobacterium phosphoreum of the SS- and LS-group, whereas the Control group also contained 21% of Carnobacterium maltaromaticum (lactic acid bacteria, LAB). Counting of LAB, using Man-Rogosa-Sharke agar, similarly showed higher numbers of the Control group after 15 d of storage. A total bacterial count of log 6 CFU/g was observed after 15 d of storage of the LS-group, which was 3 and 7 d earlier compared with the Control and SS-group, respectively. Fillet color, texture, and liquid losses were not negatively affected by preslaughter crowding stress. From the sensory and bacterial analyses, it is concluded that long-term crowding stress accelerates bacterial growth and development of unpleasant sensory properties, hence reduces the shelf life of prerigor modified atmosphere packaged (MAP) salmon. © 2011 Institute of Food Technologists.

PMID

22260135 [<http://www.ncbi.nlm.nih.gov/pubmed/?term=22260135>]

Institution

(Adland Hansen, Rodbotten, Eie, Lea, Rudi, Morkore) Nofima AS, Osloveien 1, N-1430 As, Norway

Publisher

Blackwell Publishing Inc. (350 Main Street, Malden MA 02148, United States)

Emtree Heading

animal; aquaculture; article; *Atlantic salmon; bacterial count; Carnobacterium; classification; comparative study; *crowding; food handling; growth, development and aging; heat; human; isolation and purification; mechanics; microbiology; molecular typing; odor; Photobacterium; physical chemistry; *physiological stress; physiology; quality control; *sea food/an [Drug Analysis]; sensation; taste; time.

Other Index Terms

animal; aquaculture; article; *Atlantic salmon; bacterial count; Carnobacterium; classification; comparative study; *crowding; food handling; growth, development and aging; heat; human; isolation and purification; mechanics; microbiology; molecular typing; odor; Photobacterium; physical chemistry; *physiological stress; physiology; quality control; *sea food / *drug analysis; sensation; taste; time.

Link to the Ovid Full Text or citation:

[Click here for full text options](https://libaccess.mcmaster.ca/login?url=http://ovidsp.ovid.com/ovidweb.cgi?T=JS&CSC=Y&NEWS=N&PAGE=fulltext&D=emed13&AN=364122839)

Link to the External Link Resolver:

[SFX](http://sfx.scholarsportal.info/mcmaster?sid=OVID:embase&id=pmid:22260135&id=doi:10.1111%2Fj.1750-3841.2011.02513.x&issn=0022-1147&isbn=&volume=77&issue=1&spage=S84&pages=S84-S90&date=2012&title=Journal+of+Food+Science&atitle=The+effect+of+crowding+stress+on+bacterial+growth+and+sensory+properties+of+chilled+Atlantic+salmon+fillets&aulast=Adland+Hansen&pid=<author>Adland+Hansen+A.%3BRodbotten+M.%3BEie+T.%3BLea+P.%3BRudi+K.%3BMorkore+T.<%2Fauthor><AN>364122839<%2FAN><DT>Article<%2FDT>)

68.

Lactic acid bacteria as oral delivery systems for biomolecules.

Berlec A., Ravnikar M., Strukelj B.

Pharmazie. 67 (11) (pp 891-898), 2012. Date of Publication: November 2012.

AN: 366037528

Lactic acid bacteria (LAB) have become increasingly studied over the last two decades as potential delivery systems for various biological molecules to the gastrointestinal tract. This article presents an overview of characteristics of LAB as delivery systems and of the applications which have already been developed. The majority of LAB strains are able to survive the intestinal passage and some are also able to persist and colonize the intestine. Several strains were in fact described as members of the human commensal flora. They can interact with their host and are able to deliver large molecular weight biomolecules across the epithelium via M-cells or dendritic cells. The most widely applied LAB species has been Lactococcus lactis; however species from genus Lactobacillus are gaining popularity and the first examples from genus Bifidobacterium are starting to emerge. Bacteria are mostly applied live and enable continuous delivery of the biomolecules.However, killed bacteria (e.g. gram-positive enhancer matrix), with bound biomolecules or as adjuvants, are also being developed. The techniques for genetic modification of LAB are well known. This review focuses on the delivery of recombinant proteins and DNA, which can cause either local or systemic effects. We divide recombinant proteins into antigens and therapeutic proteins. Delivery of antigens for the purpose of vaccination represents the most abundant application with numerous successful demonstrations of the efficacy on the animal model. Therapeutic proteins have mostly been developed for the treatment of the inflammatory bowel disease, by the delivery of anti-inflammatory cytokines, or downregulation of proinflammatory cytokines. Delivery of allergens for the modulation of allergic disorders represents the second most popular application of therapeutic proteins. The delivery of DNA by LAB was demonstrated and offers exciting opportunities, especially as a vaccine. New discoveries may eventually lead to the transition of LAB as delivery systems in clinical practice.

PMID

23210237 [<http://www.ncbi.nlm.nih.gov/pubmed/?term=23210237>]

Institution

(Berlec, Strukelj) Department of Biotechnology, Jozef Stefan Institute, Ljubljana, Slovenia (Ravnikar, Strukelj) University of Ljubljana, Faculty of Pharmacy, Askerceva7, SI-1000 Ljubljana, Slovenia

Publisher

Govi-Verlag Pharmazeutischer (Carl-Mannich-Str.26, Eschborn D-65760, Germany)

Emtree Heading

allergy/dt [Drug Therapy]; bacterial colonization; bactericidal activity; bacterium adherence; Bifidobacterium longum; carbohydrate metabolism; Carnobacterium; cell membrane permeability; dendritic cell; DNA modification; *drug delivery system; enteritis/dt [Drug Therapy]; Enterococcus; food industry; host pathogen interaction; human; immune response; immunomodulation; *lactic acid bacterium; Lactobacillus; Lactococcus; Lactococcus lactis; Leuconostoc; macrophage; molecular weight; nonhuman; obesity/dt [Drug Therapy]; Oenococcus; pancreas insufficiency; Pediococcus; phase 1 clinical trial (topic); review; stomach lymphoma; Streptococcus; Tetragenococcus; ulcerative colitis/dt [Drug Therapy]; vaccination; virus infection/dt [Drug Therapy]; alpha intermedin/dt [Drug Therapy]; alpha2b interferon/dt [Drug Therapy]; *bacterial vector; beta lactoglobulin/dt [Drug Therapy]; DNA; enterotoxin; envelope protein; fibroblast growth factor 10; heat shock protein; hybrid protein; interleukin 10/dt [Drug Therapy]; lymphotoxin/dt [Drug Therapy]; M protein; nucleocapsid protein; ovalbumin/dt [Drug Therapy]; oxyntomodulin/dt [Drug Therapy]; probiotic agent; ribosome protein; tetanus toxin; tumor necrosis factor alpha/dt [Drug Therapy]; tumor necrosis factor related apoptosis inducing ligand.

Drug Index Terms

alpha intermedin / drug therapy; alpha2b interferon / drug therapy; *bacterial vector; beta lactoglobulin / drug therapy; DNA; enterotoxin; envelope protein; fibroblast growth factor 10; heat shock protein; hybrid protein; interleukin 10 / drug therapy; lymphotoxin / drug therapy; M protein; nucleocapsid protein; ovalbumin / drug therapy; oxyntomodulin / drug therapy; probiotic agent; ribosome protein; tetanus toxin; tumor necrosis factor alpha / drug therapy; tumor necrosis factor related apoptosis inducing ligand.

Other Index Terms

allergy / drug therapy; bacterial colonization; bactericidal activity; bacterium adherence; Bifidobacterium longum; carbohydrate metabolism; Carnobacterium; cell membrane permeability; dendritic cell; DNA modification; *drug delivery system; enteritis / drug therapy; Enterococcus; food industry; host pathogen interaction; human; immune response; immunomodulation; *lactic acid bacterium; Lactobacillus; Lactococcus; Lactococcus lactis; Leuconostoc; macrophage; molecular weight; nonhuman; obesity / drug therapy; Oenococcus; pancreas insufficiency; Pediococcus; phase 1 clinical trial (topic); review; stomach lymphoma; Streptococcus; Tetragenococcus; ulcerative colitis / drug therapy; vaccination; virus infection / drug therapy.

Link to the Ovid Full Text or citation:

[Click here for full text options](https://libaccess.mcmaster.ca/login?url=http://ovidsp.ovid.com/ovidweb.cgi?T=JS&CSC=Y&NEWS=N&PAGE=fulltext&D=emed13&AN=366037528)

Link to the External Link Resolver:

[SFX](http://sfx.scholarsportal.info/mcmaster?sid=OVID:embase&id=pmid:23210237&id=doi:10.1691%2Fph.2012.1705&issn=0031-7144&isbn=&volume=67&issue=11&spage=891&pages=891-898&date=2012&title=Pharmazie&atitle=Lactic+acid+bacteria+as+oral+delivery+systems+for+biomolecules&aulast=Berlec&pid=<author>Berlec+A.%3BRavnikar+M.%3BStrukelj+B.<%2Fauthor><AN>366037528<%2FAN><DT>Review<%2FDT>)

69.

Characterization of pro-inflammatory flagellin proteins produced by lactobacillus ruminis and related motile lactobacilli.

Neville B.A., Forde B.M., Claesson M.J., Darby T., Coghlan A., Nally K., Ross R.P., O'Toole P.W.

PLoS ONE. 7 (7) (no pagination), 2012. Article Number: e40592. Date of Publication: 10 Jul 2012.

AN: 365221655

Lactobacillus ruminis is one of at least twelve motile but poorly characterized species found in the genus Lactobacillus. Of these, only L. ruminis has been isolated from mammals, and this species may be considered as an autochthonous member of the gastrointestinal microbiota of humans, pigs and cows. Nine L. ruminis strains were investigated here to elucidate the biochemistry and genetics of Lactobacillus motility. Six strains isolated from humans were non-motile while three bovine isolates were motile. A complete set of flagellum biogenesis genes was annotated in the sequenced genomes of two strains, ATCC25644 (human isolate) and ATCC27782 (bovine isolate), but only the latter strain produced flagella. Comparison of the L. ruminis and L. mali DSM20444T motility loci showed that their genetic content and gene-order were broadly similar, although the L. mali motility locus was interrupted by an 11.8 Kb region encoding rhamnose utilization genes that is absent from the L. ruminis motility locus. Phylogenetic analysis of 39 motile bacteria indicated that Lactobacillus motility genes were most closely related to those of motile carnobacteria and enterococci. Transcriptome analysis revealed that motility genes were transcribed at a significantly higher level in motile L. ruminis ATCC27782 than in non-motile ATCC25644. Flagellin proteins were isolated from L. ruminis ATCC27782 and from three other Lactobacillus species, while recombinant flagellin of aflagellate L. ruminis ATCC25644 was expressed and purified from E. coli. These native and recombinant Lactobacillus flagellins, and also flagellate L. ruminis cells, triggered interleukin-8 production in cultured human intestinal epithelial cells in a manner suppressed by short interfering RNA directed against Toll-Like Receptor 5. This study provides genetic, transcriptomic, phylogenetic and immunological insights into the trait of flagellum-mediated motility in the lactobacilli. © 2012 Neville et al.

PMID

22808200 [<http://www.ncbi.nlm.nih.gov/pubmed/?term=22808200>]

Institution

(Neville, Forde, Claesson, Coghlan, O'Toole) Department of Microbiology, University College Cork, Cork, Ireland (Darby, Nally, O'Toole) Alimentary Pharmabiotic Centre, University College Cork, Cork, Ireland

(Ross) Teagasc, Moorepark Food Research Centre, Moorepark, Fermoy, Co. Cork, Ireland

Publisher

Public Library of Science (185 Berry Street, Suite 1300, San Francisco CA 94107, United States)

Emtree Heading

animal experiment; article; bacterial gene; bacterial genome; bacterial strain; bacterium isolate; Carnobacterium; controlled study; cytokine production; Enterococcus; Escherichia coli; *flagellum; gene locus; genetic transcription; human; human cell; intestine epithelium cell; *Lactobacillus; Lactobacillus salivarius; mouse; nonhuman; nucleotide sequence; phylogeny; protein analysis; protein expression; protein isolation; protein purification; protein synthesis; *flagellin; interleukin 8/ec [Endogenous Compound]; recombinant protein; transcriptome; Lactobacillus ghanensis; Lactobacillus mali; Lactobacillus nagelii; *Lactobacillus ruminis.

Candidate Terms

Lactobacillus ghanensis [other term]; Lactobacillus mali [other term]; Lactobacillus nagelii [other term]; *Lactobacillus ruminis [other term].

Drug Index Terms

*flagellin; interleukin 8 / endogenous compound; recombinant protein; transcriptome.

Other Index Terms

animal experiment; article; bacterial gene; bacterial genome; bacterial strain; bacterium isolate; Carnobacterium; controlled study; cytokine production; Enterococcus; Escherichia coli; *flagellum; gene locus; genetic transcription; human; human cell; intestine epithelium cell; *Lactobacillus; Lactobacillus salivarius; mouse; nonhuman; nucleotide sequence; phylogeny; protein analysis; protein expression; protein isolation; protein purification; protein synthesis.

Link to the Ovid Full Text or citation:

[Click here for full text options](https://libaccess.mcmaster.ca/login?url=http://ovidsp.ovid.com/ovidweb.cgi?T=JS&CSC=Y&NEWS=N&PAGE=fulltext&D=emed13&AN=365221655)

Link to the External Link Resolver:

[SFX](http://sfx.scholarsportal.info/mcmaster?sid=OVID:embase&id=pmid:22808200&id=doi:10.1371%2Fjournal.pone.0040592&issn=1932-6203&isbn=&volume=7&issue=7&spage=&pages=&date=2012&title=PLoS+ONE&atitle=Characterization+of+pro-inflammatory+flagellin+proteins+produced+by+lactobacillus+ruminis+and+related+motile+lactobacilli&aulast=Neville&pid=<author>Neville+B.A.%3BForde+B.M.%3BClaesson+M.J.%3BDarby+T.%3BCoghlan+A.%3BNally+K.%3BRoss+R.P.%3BO'Toole+P.W.<%2Fauthor><AN>365221655<%2FAN><DT>Article<%2FDT>)

70.

The genome sequence of the lactic acid bacterium, Carnobacterium maltaromaticum ATCC 35586 encodes potential virulence factors.

Leisner J.J., Hansen M.A., Larsen M.H., Hansen L., Ingmer H., Sorensen S.J.

International Journal of Food Microbiology. 152 (3) (pp 107-115), 2012. Date of Publication: 16 January 2012.

AN: 51490503

The genus Carnobacterium belongs to the lactic acid bacteria and Carnobacterium maltaromaticum is commonly found in modified atmosphere packed and vacuum packed fish and meat products as well as in live fish. This species has been described as a fish pathogenic organism but human clinical isolates have only been obtained at one occasion. To investigate the virulence potential we sequenced the entire genome of strain ATCC 35586, isolated from a diseased salmon. When comparing the translated gene products of ATCC 35586 to those of Gram positive bacterial pathogens and probiotics as well as the related Carnobacterium sp. AT7 we identified a range of putative virulence genes including genes encoding products involved in adhesion to fibronectin and collagen, capsule synthesis, cell wall modification, iron scavenging mechanisms, haemolysis, invasion and resistance to toxic compounds. Of particular interest was the presence of internalin encoding gene homologues to some of those found in Listeria spp. and Lactobacillus plantarum. Furthermore, the ATCC 35586 strain possesses a gene encoding a product similar to the central Listeria monocytogenes transcriptional regulator PrfA, that in this organism controls virulence gene expression by binding to conserved DNA binding sites. Based on the consensus DNA sequence of this binding site, we identified a total of 65 genes in the ATCC 35586 genome that in the upstream region carry a PrfA binding motif. Among these is one of the internalin encoding genes; two genes encoding products involved in capsule biosynthesis as well as various genes encoding products with metabolic functions. In contrast to L. monocytogenes, the ATCC 35586 strain did not encode other PrfA dependent virulence factors such as listeriolysin O, phospholipases A and B, ActA, listeriolysin O, zinc metallo protease and internalins A and B. In conclusion, C. maltaromaticum ATCC 35586 carries putative virulence genes that may explain its reported ability to infect fish. The findings of this study give no reason for concern regarding human health by the presence of this species in food products. © 2011 Elsevier B.V.

PMID

21704418 [<http://www.ncbi.nlm.nih.gov/pubmed/?term=21704418>]

Institution

(Leisner, Larsen, Ingmer) Department of Veterinary Disease Biology, Faculty of Life Sciences, University of Copenhagen, Denmark (Hansen, Hansen, Sorensen) Department of Biology, Faculty of Science, University of Copenhagen, Denmark

Publisher

Elsevier (P.O. Box 211, Amsterdam 1000 AE, Netherlands)

Emtree Heading

antibiotic resistance; article; Bacillus cereus; bacterial membrane; bacterial strain; *bacterial virulence; bacterium isolation; binding site; Carnobacterium; *Carnobacterium maltaromaticum; DNA binding; DNA binding motif; *DNA sequence; Enterococcus faecalis; extracellular matrix; fish disease; gene expression; Gram positive bacterium; hemolysis; Lactobacillus plantarum; Listeria monocytogenes; nonhuman; salmon; species invasion; Streptococcus pyogenes; vacuum; chlortetracycline; collagen; erythromycin; fibronectin; listeriolysin O; nitrofuran; phospholipase A; probiotic agent; quinolone; sulfatrimethoprim.

Drug Index Terms

chlortetracycline; collagen; erythromycin; fibronectin; listeriolysin O; nitrofuran; phospholipase A; probiotic agent; quinolone; sulfatrimethoprim.

Other Index Terms

antibiotic resistance; article; Bacillus cereus; bacterial membrane; bacterial strain; *bacterial virulence; bacterium isolation; binding site; Carnobacterium; *Carnobacterium maltaromaticum; DNA binding; DNA binding motif; *DNA sequence; Enterococcus faecalis; extracellular matrix; fish disease; gene expression; Gram positive bacterium; hemolysis; Lactobacillus plantarum; Listeria monocytogenes; nonhuman; salmon; species invasion; Streptococcus pyogenes; vacuum.

Link to the Ovid Full Text or citation:

[Click here for full text options](https://libaccess.mcmaster.ca/login?url=http://ovidsp.ovid.com/ovidweb.cgi?T=JS&CSC=Y&NEWS=N&PAGE=fulltext&D=emed13&AN=51490503)

Link to the External Link Resolver:

[SFX](http://sfx.scholarsportal.info/mcmaster?sid=OVID:embase&id=pmid:21704418&id=doi:10.1016%2Fj.ijfoodmicro.2011.05.012&issn=0168-1605&isbn=&volume=152&issue=3&spage=107&pages=107-115&date=2012&title=International+Journal+of+Food+Microbiology&atitle=The+genome+sequence+of+the+lactic+acid+bacterium%2C+Carnobacterium+maltaromaticum+ATCC+35586+encodes+potential+virulence+factors&aulast=Leisner&pid=<author>Leisner+J.J.%3BHansen+M.A.%3BLarsen+M.H.%3BHansen+L.%3BIngmer+H.%3BSorensen+S.J.<%2Fauthor><AN>51490503<%2FAN><DT>Article<%2FDT>)

71.

Selection and design of lactic acid bacteria probiotic cultures.

Giraffa G.

Engineering in Life Sciences. 12 (4) (pp 391-398), 2012. Date of Publication: August 2012.

AN: 365534665

Probiotics are defined as "live microorganisms that, when administered in adequate amounts, confer a health benefit on the host." Probiotic-containing foods can be categorized as functional foods and, along with prebiotics, represent the largest segment of the functional food market around the world. Fermented foods are the main vehicle of administration of probiotic organisms and, among them, dairy products are by far the most widely used. Many probiotic strains, which most often belong to the genera Lactobacillus and Bifidobacterium, are already applied in commercial probiotic fermented milks throughout the world. Nonetheless, the market of biofunctional dairy products, including probiotics, is continuously asking for diversifying the range of available products. To this regard, there is a growing need to identify new biofunctional strains, new strategies to assure survival of these cultures, and different sources from which to isolate strains. Before a probiotic can benefit human health, it must fulfill several criteria. Because the range of target functions and technological applications is wide, the selection and evaluation of potential probiotic candidates require a multistep approach. This review will give a general overview of main criteria and methods that have to be taken into consideration in the selection process of probiotic strains. © 2012 WILEY-VCH Verlag GmbH & Co. KGaA, Weinheim.

Institution

(Giraffa) Agriculture Research Council, Fodder and Dairy Productions Research Centre (CRA-FLC), Lodi, Italy

Publisher

Wiley-VCH Verlag (P.O. Box 101161, Weinheim D-69451, Germany)

Emtree Heading

Aerococcus; bacterial strain; bacterial viability; bacterium culture; Bifidobacterium; Carnobacterium; cheese; Enterococcus; fermentation; fermented product; food; food intake; food processing; food quality; health; in vitro study; in vivo study; *lactic acid bacterium; Lactobacillus; Lactococcus; Leuconostoc; milk; nonhuman; Oenococcus; Pediococcus; review; safety; Streptococcus; taxonomy; Tetragenococcus; Weissella; prebiotic agent; *probiotic agent.

Drug Index Terms

prebiotic agent; *probiotic agent.

Other Index Terms

Aerococcus; bacterial strain; bacterial viability; bacterium culture; Bifidobacterium; Carnobacterium; cheese; Enterococcus; fermentation; fermented product; food; food intake; food processing; food quality; health; in vitro study; in vivo study; *lactic acid bacterium; Lactobacillus; Lactococcus; Leuconostoc; milk; nonhuman; Oenococcus; Pediococcus; review; safety; Streptococcus; taxonomy; Tetragenococcus; Weissella.

Link to the Ovid Full Text or citation:

[Click here for full text options](https://libaccess.mcmaster.ca/login?url=http://ovidsp.ovid.com/ovidweb.cgi?T=JS&CSC=Y&NEWS=N&PAGE=fulltext&D=emed13&AN=365534665)

Link to the External Link Resolver:

[SFX](http://sfx.scholarsportal.info/mcmaster?sid=OVID:embase&id=pmid:&id=doi:10.1002%2Felsc.201100118&issn=1618-0240&isbn=&volume=12&issue=4&spage=391&pages=391-398&date=2012&title=Engineering+in+Life+Sciences&atitle=Selection+and+design+of+lactic+acid+bacteria+probiotic+cultures&aulast=Giraffa&pid=<author>Giraffa+G.<%2Fauthor><AN>365534665<%2FAN><DT>Review<%2FDT>)

72.

Discovery and applications of naturally occurring cyclic peptides.

Thorstholm L., Craik D.J.

Drug Discovery Today: Technologies. 9 (1) (pp e13-e21), 2012. Date of Publication: Spring 2012.

AN: 365556446

Over the last decade several families of naturally occurring cyclic peptides have been discovered that are extremely stable and have important roles as defense molecules for their host organisms. Because of their exceptional stability and potent bioactivities they can be adapted for use as scaffolds in drug development. Here we describe technologies for the application of cyclic peptides in drug design. © 2011 Elsevier Ltd.

Institution

(Thorstholm, Craik) Division of Chemistry and Structural Biology, Institute for Molecular Bioscience, University of Queensland, Brisbane, QLD 4072, Australia

Publisher

Elsevier Ltd (Langford Lane, Kidlington, Oxford OX5 1GB, United Kingdom)

Emtree Heading

antimicrobial activity; article; Bacillus subtilis; Butyrivibrio fibrisolvens; carbon nuclear magnetic resonance; Carnobacterium maltaromaticum; Clostridium beijerinckii; cyclization; drug design; Escherichia coli; food preservation; gene activation; Helianthus annuus; human; Lactobacillus gasseri; Lactococcus garvieae; Macaca mulatta; nitrogen nuclear magnetic resonance; nonhuman; Oldenlandia; Psychotria; Rhizobium radiobacter; Streptococcus uberis; synthesis; Violaceae; alpha amanitin; *cyclopeptide; cyclosporin; defensin; epitope; subtilisin; unclassified drug; carnocyclin a; circularin a; circulin; cycloviolacin y1; garvicin ml; gassericin a; lactocyclicin q; phallocidin; rhesus theta defensin 1; sunflower trypsin inhibitor 1; tricyclon a; uberolysin.

Candidate Terms

carnocyclin a [drug term]; circularin a [drug term]; circulin [drug term]; cycloviolacin y1 [drug term]; garvicin ml [drug term]; gassericin a [drug term]; lactocyclicin q [drug term]; phallocidin [drug term]; rhesus theta defensin 1 [drug term]; sunflower trypsin inhibitor 1 [drug term]; tricyclon a [drug term]; uberolysin [drug term].

Drug Index Terms

alpha amanitin; *cyclopeptide; cyclosporin; defensin; epitope; subtilisin; unclassified drug.

Other Index Terms

antimicrobial activity; article; Bacillus subtilis; Butyrivibrio fibrisolvens; carbon nuclear magnetic resonance; Carnobacterium maltaromaticum; Clostridium beijerinckii; cyclization; drug design; Escherichia coli; food preservation; gene activation; Helianthus annuus; human; Lactobacillus gasseri; Lactococcus garvieae; Macaca mulatta; nitrogen nuclear magnetic resonance; nonhuman; Oldenlandia; Psychotria; Rhizobium radiobacter; Streptococcus uberis; synthesis; Violaceae.

Link to the Ovid Full Text or citation:

[Click here for full text options](https://libaccess.mcmaster.ca/login?url=http://ovidsp.ovid.com/ovidweb.cgi?T=JS&CSC=Y&NEWS=N&PAGE=fulltext&D=emed13&AN=365556446)

Link to the External Link Resolver:

[SFX](http://sfx.scholarsportal.info/mcmaster?sid=OVID:embase&id=pmid:&id=doi:10.1016%2Fj.ddtec.2011.07.005&issn=1740-6749&isbn=&volume=9&issue=1&spage=e13&pages=e13-e21&date=2012&title=Drug+Discovery+Today%3A+Technologies&atitle=Discovery+and+applications+of+naturally+occurring+cyclic+peptides&aulast=Thorstholm&pid=<author>Thorstholm+L.%3BCraik+D.J.<%2Fauthor><AN>365556446<%2FAN><DT>Article<%2FDT>)

73.

Sensory characteristics of spoilage and volatile compounds associated with bacteria isolated from cooked and peeled tropical shrimps using SPME-GC-MS analysis.

Jaffres E., Lalanne V., Mace S., Cornet J., Cardinal M., Serot T., Dousset X., Joffraud J.-J.

International Journal of Food Microbiology. 147 (3) (pp 195-202), 2011. Date of Publication: 30 June 2011.

AN: 51398623

The spoilage potential of six bacterial species isolated from cooked and peeled tropical shrimps (Brochothrix thermosphacta, Serratia liquefaciens-like, Carnobacterium maltaromaticum, Carnobacterium divergens, Carnobacterium alterfunditum-like and Vagococcus penaei sp. nov.) was evaluated. The bacteria were inoculated into shrimps, packaged in a modified atmosphere and stored for 27 days at 8. degreeC. Twice a week, microbial growth, as well as chemical and sensory changes, were monitored during the storage period. The bacteria mainly involved in shrimp spoilage were B. thermosphacta, S. liquefaciens-like and C. maltaromaticum whose main characteristic odours were cheese-sour, cabbage-amine and cheese-sour-butter, respectively. The volatile fraction of the inoculated shrimp samples was analysed by solid-phase microextraction (SPME) and gas chromatography coupled to mass spectrometry (GC-MS). This method showed that the characteristic odours were most likely induced by the production of volatile compounds such as 3-methyl-1-butanal, 2,3-butanedione, 2-methyl-1-butanal, 2,3-heptanedione and trimethylamine. © 2011 Elsevier B.V.

PMID

21531471 [<http://www.ncbi.nlm.nih.gov/pubmed/?term=21531471>]

Institution

(Jaffres, Mace, Dousset) LUNAM Universite, Oniris, UMR 1014 Secalim, Nantes, F-44307, France (Jaffres, Mace, Dousset) INRA, Nantes, F-44307, France

(Jaffres, Mace, Cornet, Cardinal, Joffraud) Ifremer, Laboratoire Science et Technologie de la Biomasse Marine, BP 21105, 4311 Nantes Cedex 3, France

(Lalanne, Serot) LUNAM Universite, ONIRIS, UMR 6144 GEPEA, Flavor Unit, F 44307, France

(Jaffres, Lalanne, Mace, Serot) CNRS, Nantes, F-44307, France

Publisher

Elsevier (P.O. Box 211, Amsterdam 1000 AE, Netherlands)

Emtree Heading

article; bacterium isolation; Brochothrix thermosphacta; Carnobacterium; Carnobacterium maltaromaticum; chemical analysis; *cooked food; food analysis; food contamination; *food spoilage; food storage; gas chromatography; Gram positive bacterium; human; mass spectrometry; microbial growth; nonhuman; odor; sensory analysis; Serratia liquefaciens; *shrimp; solid phase microextraction; 2,3 butanedione; trimethylamine; unclassified drug; *volatile agent; Carnobacterium alterfunditum; carnobacterium divergens; Vagococcus penai; 2 methyl 1 butanal; 2,3 heptanedione; 3 methyl 1 butanal.

Candidate Terms

Carnobacterium alterfunditum [other term]; Carnobacterium divergens [other term]; Vagococcus penai [other term]; 2 methyl 1 butanal [drug term]; 2,3 heptanedione [drug term]; 3 methyl 1 butanal [drug term].

Drug Index Terms

2,3 butanedione; trimethylamine; unclassified drug; *volatile agent.

Other Index Terms

article; bacterium isolation; Brochothrix thermosphacta; Carnobacterium; Carnobacterium maltaromaticum; chemical analysis; *cooked food; food analysis; food contamination; *food spoilage; food storage; gas chromatography; Gram positive bacterium; human; mass spectrometry; microbial growth; nonhuman; odor; sensory analysis; Serratia liquefaciens; *shrimp; solid phase microextraction.

Link to the Ovid Full Text or citation:

[Click here for full text options](https://libaccess.mcmaster.ca/login?url=http://ovidsp.ovid.com/ovidweb.cgi?T=JS&CSC=Y&NEWS=N&PAGE=fulltext&D=emed12&AN=51398623)

Link to the External Link Resolver:

[SFX](http://sfx.scholarsportal.info/mcmaster?sid=OVID:embase&id=pmid:21531471&id=doi:10.1016%2Fj.ijfoodmicro.2011.04.008&issn=0168-1605&isbn=&volume=147&issue=3&spage=195&pages=195-202&date=2011&title=International+Journal+of+Food+Microbiology&atitle=Sensory+characteristics+of+spoilage+and+volatile+compounds+associated+with+bacteria+isolated+from+cooked+and+peeled+tropical+shrimps+using+SPME-GC-MS+analysis&aulast=Jaffres&pid=<author>Jaffres+E.%3BLalanne+V.%3BMace+S.%3BCornet+J.%3BCardinal+M.%3BSerot+T.%3BDousset+X.%3BJoffraud+J.-J.<%2Fauthor><AN>51398623<%2FAN><DT>Article<%2FDT>)

74.

Carnobacterium maltaromaticum: Identification, isolation tools, ecology and technological aspects in dairy products.

Afzal M.I., Jacquet T., Delaunay S., Borges F., Milliere J.-B., Revol-Junelles A.-M., Cailliez-Grimal C.

Food Microbiology. 27 (5) (pp 573-579), 2010. Date of Publication: August 2010.

AN: 50881737

Carnobacterium species constitute a genus of Lactic Acid Bacteria (LAB) present in different ecological niches. The aim of this article is to summarize the knowledge about Carnobacterium maltaromaticum species at different microbiological levels such as taxonomy, isolation and identification, ecology, technological aspects and safety in dairy products. Works published during the last decade concerning C. maltaromaticum have shown that this non-starter LAB (NSLAB) could present major interests in dairy product technology. Four reasons can be mentioned: i) it can grow in milk during the ripening period with no competition with starter LAB, ii) this species synthesizes different flavouring compounds e.g., 3-methylbutanal, iii) it can inhibit the growth of foodborne pathogens as Listeria monocytogenes due to its ability to produce bacteriocins, iv) it has never been reported to be involved in human diseases as no cases of human infection have been directly linked to the consumption of dairy products containing this species. © 2010 Elsevier Ltd.

PMID

20510773 [<http://www.ncbi.nlm.nih.gov/pubmed/?term=20510773>]

Institution

(Afzal, Jacquet, Borges, Milliere, Revol-Junelles, Cailliez-Grimal) Nancy-Universite, Institut National Polytechnique de Lorraine, Laboratoire d'Ingenierie des Biomolecules, 2, avenue de la Foret de Haye, B.P. 172, 54505 Vandoeuvre-les-Nancy, France (Delaunay) Nancy-Universite, Institut National Polytechnique de Lorraine, Laboratoire Reaction et Genie des Procedes, UPR CNRS 3349, 2, avenue de la Foret de Haye, B.P. 172, 54505 Vandoeuvre-les-Nancy, France

Publisher

Academic Press (24-28 Oval Road, London NW1 7DX, United Kingdom)

Emtree Heading

animal; *bacterium identification; *Carnobacterium; classification; *dairy product; *food handling; food preservation; genetics; human; isolation and purification; methodology; microbiology; physiology; review; safety.

Other Index Terms

animal; *bacterium identification; *Carnobacterium; classification; *dairy product; *food handling; food preservation; genetics; human; isolation and purification; methodology; microbiology; physiology; review; safety.

Link to the Ovid Full Text or citation:

[Click here for full text options](https://libaccess.mcmaster.ca/login?url=http://ovidsp.ovid.com/ovidweb.cgi?T=JS&CSC=Y&NEWS=N&PAGE=fulltext&D=emed11&AN=50881737)

Link to the External Link Resolver:

[SFX](http://sfx.scholarsportal.info/mcmaster?sid=OVID:embase&id=pmid:20510773&id=doi:10.1016%2Fj.fm.2010.03.019&issn=0740-0020&isbn=&volume=27&issue=5&spage=573&pages=573-579&date=2010&title=Food+Microbiology&atitle=Carnobacterium+maltaromaticum%3A+Identification%2C+isolation+tools%2C+ecology+and+technological+aspects+in+dairy+products&aulast=Afzal&pid=<author>Afzal+M.I.%3BJacquet+T.%3BDelaunay+S.%3BBorges+F.%3BMilliere+J.-B.%3BRevol-Junelles+A.-M.%3BCailliez-Grimal+C.<%2Fauthor><AN>50881737<%2FAN><DT>Review<%2FDT>)

75.

Tetracycline resistance associated with commensal bacteria from representative ready-to-consume deli and restaurant foods.

Li X., Wang H.H.

Journal of Food Protection. 73 (10) (pp 1841-1848), 2010. Date of Publication: October 2010.

AN: 360022231

Proper knowledge of antibiotic resistance (AR) dissemination is essential for effective mitigation. This study examined the profiles of tetracycline-resistant (Tet r ) commensal bacteria from representative ready-to-consume food samples from salad bars at local grocery stores and restaurants. Out of 900 Tetr isolates examined, 158 (17.6%) carried one or more of tetM, tetL, tetS, and tetK genes by conventional PCR, 28 harbored more than one Tetr determinants. The most prevalent genotype was tetM, which was detected in 70.9% of the AR gene carriers, followed by tetL (31.6%), tetS (13.9%), and tetK (2.5%). Identified AR gene carriers included Enterococcus, Lactococcus, Staphylococcus, Brochothrix, Carnobacterium, Stenotrophomonas, Pseudomonas, and Sphingobacterium, by 16S rRNA gene sequence analysis. AR determinants were successfully transmitted, and led to resistance in Streptococcus mutans via natural gene transformation and Enterococcus faecalis via electroporation, suggesting the functionality and mobility of the AR genes from the food commensal bacteria. In addition, the AR traits in many isolates are quite stable, even in the absence of the selective pressure. The identification of new commensal carriers for representative AR genes revealed the involvement of a broad spectrum of bacteria in the horizontal transmission of AR genes. Meanwhile, the spectrum of the antibiotic-resistant bacteria differed from the spectrum of the total bacteria (by denaturing gradient gel electrophoresis) associated with the food items. Our data revealed a common avenue in AR exposure and will assist in proper risk assessment and the development of comprehensive mitigation strategies to effectively combat AR. Copyright © International Association for Food Protection.

PMID

21067672 [<http://www.ncbi.nlm.nih.gov/pubmed/?term=21067672>]

Institution

(Li, Wang) Department of Food Science, Ohio State University, 2015 Fyffe Court, Columbus, OH 43210, United States (Wang) Department of Microbiology, Ohio State University, 2015 Fyffe Court, Columbus, OH 43210, United States

Publisher

IAMFES (6200 Aurora Avenue, Suite 200W, Des Moines IA 50322-3863, United States)

Emtree Heading

*antibiotic resistance; article; *bacterium; catering service; drug effect; *food control; genetics; horizontal gene transfer; human; *meat; microbiological examination; microbiology; *antiinfective agent/pd [Pharmacology].

Drug Index Terms

*antiinfective agent / *pharmacology.

Other Index Terms

*antibiotic resistance; article; *bacterium; catering service; drug effect; *food control; genetics; horizontal gene transfer; human; *meat; microbiological examination; microbiology.

Link to the Ovid Full Text or citation:

[Click here for full text options](https://libaccess.mcmaster.ca/login?url=http://ovidsp.ovid.com/ovidweb.cgi?T=JS&CSC=Y&NEWS=N&PAGE=fulltext&D=emed11&AN=360022231)

Link to the External Link Resolver:

[SFX](http://sfx.scholarsportal.info/mcmaster?sid=OVID:embase&id=pmid:21067672&id=doi:10.4315%2F0362-028X-73.10.1841&issn=0362-028X&isbn=&volume=73&issue=10&spage=1841&pages=1841-1848&date=2010&title=Journal+of+Food+Protection&atitle=Tetracycline+resistance+associated+with+commensal+bacteria+from+representative+ready-to-consume+deli+and+restaurant+foods&aulast=Li&pid=<author>Li+X.%3BWang+H.H.<%2Fauthor><AN>360022231<%2FAN><DT>Article<%2FDT>)

76.

Isolation of Carnobacterium sp. from a human blood culture.

Hoenigl M., Grisold A.J., Valentin T., Leitner E., Zarfel G., Renner H., Krause R.

Journal of Medical Microbiology. 59 (4) (pp 493-495), 2010. Date of Publication: April 2010.

AN: 358615713

Carnobacterium species have been isolated from the environment and are not regarded as human pathogens, although they are known to cause disease in fish. Only two reports describing isolation of Carnobacterium species from human pus were found in the literature. We report what we believe to be the first isolation of Carnobacterium sp. from a human blood culture. © 2010 SGM.

PMID

20075110 [<http://www.ncbi.nlm.nih.gov/pubmed/?term=20075110>]

Institution

(Hoenigl, Valentin, Krause) Section of Infectious Diseases, Department of Internal Medicine, Medical University of Graz, A-8010 Graz, Austria (Grisold, Leitner, Zarfel) Institute of Hygiene, Microbiology and Environmental Medicine, Medical University of Graz, A-8010 Graz, Austria

(Renner) Division of Thoracic Surgery, Medical University of Graz, A-8010 Graz, Austria

Publisher

Society for General Microbiology (Basingstoke Road, Spencers Wood, Reading, Berkshire RG7 1AE, United Kingdom)

Emtree Heading

add on therapy; adult; article; *bacterium culture; *bacterium isolation; *blood culture; calcitonin blood level; *Carnobacterium; case report; creatinine blood level; differential diagnosis; drug substitution; fever; human; intensive care unit; lactate dehydrogenase blood level; listeriosis/di [Diagnosis]; listeriosis/dt [Drug Therapy]; lumbar puncture; malaise; male; meningitis/di [Diagnosis]; meningitis/dt [Drug Therapy]; meningococcosis/di [Diagnosis]; meningococcosis/dt [Drug Therapy]; neck pain; patient transport; priority journal; shoulder pain; treatment outcome; ampicillin/dt [Drug Therapy]; ampicillin/iv [Intravenous Drug Administration]; C reactive protein/ec [Endogenous Compound]; ceftriaxone/dt [Drug Therapy]; ceftriaxone/iv [Intravenous Drug Administration]; creatinine/ec [Endogenous Compound]; lactate dehydrogenase/ec [Endogenous Compound]; moxifloxacin/dt [Drug Therapy]; moxifloxacin/po [Oral Drug Administration]; procalcitonin/ec [Endogenous Compound].

Drug Index Terms

ampicillin / drug therapy / intravenous drug administration; C reactive protein / endogenous compound; ceftriaxone / drug therapy / intravenous drug administration; creatinine / endogenous compound; lactate dehydrogenase / endogenous compound; moxifloxacin / drug therapy / oral drug administration; procalcitonin / endogenous compound.

Other Index Terms

add on therapy; adult; article; *bacterium culture; *bacterium isolation; *blood culture; calcitonin blood level; *Carnobacterium; case report; creatinine blood level; differential diagnosis; drug substitution; fever; human; intensive care unit; lactate dehydrogenase blood level; listeriosis / diagnosis / drug therapy; lumbar puncture; malaise; male; meningitis / diagnosis / drug therapy; meningococcosis / diagnosis / drug therapy; neck pain; patient transport; priority journal; shoulder pain; treatment outcome.

Link to the Ovid Full Text or citation:

[Click here for full text options](https://libaccess.mcmaster.ca/login?url=http://ovidsp.ovid.com/ovidweb.cgi?T=JS&CSC=Y&NEWS=N&PAGE=fulltext&D=emed11&AN=358615713)

Link to the External Link Resolver:

[SFX](http://sfx.scholarsportal.info/mcmaster?sid=OVID:embase&id=pmid:20075110&id=doi:10.1099%2Fjmm.0.016808-0&issn=0022-2615&isbn=&volume=59&issue=4&spage=493&pages=493-495&date=2010&title=Journal+of+Medical+Microbiology&atitle=Isolation+of+Carnobacterium+sp.+from+a+human+blood+culture&aulast=Hoenigl&pid=<author>Hoenigl+M.%3BGrisold+A.J.%3BValentin+T.%3BLeitner+E.%3BZarfel+G.%3BRenner+H.%3BKrause+R.<%2Fauthor><AN>358615713<%2FAN><DT>Article<%2FDT>)

77.

Interactions between bacterial isolates from modified-atmosphere-packaged artisan-type cooked ham in view of the development of a bioprotective culture.

Vasilopoulos C., De Mey E., Dewulf L., Paelinck H., De Smedt A., Vandendriessche F., De Vuyst L., Leroy F.

Food Microbiology. 27 (8) (pp 1086-1094), 2010. Date of Publication: December 2010.

AN: 51033257

Growth and metabolite production of three dominant bacterial isolates (Carnobacterium divergens 3M14, Leuconostoc carnosum 3M42, and Brochothrix thermosphacta RMS6) from modified-atmosphere-packaged (MAP), artisan-type cooked ham were assessed for their interactions in view of the development of a bioprotective culture. During monoculture experiments in cooked ham simulation medium, Leuc. carnosum 3M42 converted the available glucose into lactic acid and ethanol, whereas the two other strains produced additional metabolites such as acetic acid and 3-methyl-1-butanol. When grown in co-culture, Leuc. carnosum 3M42 suppressed the growth and metabolism of B. thermosphacta RMS6. In contrast, a co-culture of the latter bacterium with C. divergens 3M14 led to a variety of spoilage-related metabolic compounds. Subsequently, experiments with a commercial cooked ham product indicated that Leuc. carnosum 3M42 dominated the meat matrix and improved acceptability of the product over time, hence acting as a bioprotective culture for MAP, artisan-type cooked ham. © 2010 Elsevier Ltd.

PMID

20832689 [<http://www.ncbi.nlm.nih.gov/pubmed/?term=20832689>]

Institution

(Vasilopoulos, De Vuyst, Leroy) Research Group of Industrial Microbiology and Food Biotechnology (IMDO), Faculty of Sciences and Bio-engineering Sciences, Vrije Universiteit Brussel, Pleinlaan 2, B-1050 Brussels, Belgium (De Mey, Dewulf, Paelinck) Research Group for Technology and Quality of Animal Products, Department of Industrial Engineering, KaHo Sint-Lieven, Gebroeders Desmetstraat 1, B-9000 Ghent, Belgium

(De Smedt, Vandendriessche) Imperial Meat Products, Grote Baan 200, B-9920 Lovendegem, Belgium

Publisher

Academic Press (24-28 Oval Road, London NW1 7DX, United Kingdom)

Emtree Heading

animal; article; *Brochothrix; *Carnobacterium; cattle; fermentation; food packaging; growth, development and aging; human; isolation and purification; *Leuconostoc; *meat/an [Drug Analysis]; metabolism; microbiology; taste.

Other Index Terms

animal; article; *Brochothrix; *Carnobacterium; cattle; fermentation; food packaging; growth, development and aging; human; isolation and purification; *Leuconostoc; *meat / *drug analysis; metabolism; microbiology; taste.

Link to the Ovid Full Text or citation:

[Click here for full text options](https://libaccess.mcmaster.ca/login?url=http://ovidsp.ovid.com/ovidweb.cgi?T=JS&CSC=Y&NEWS=N&PAGE=fulltext&D=emed11&AN=51033257)

Link to the External Link Resolver:

[SFX](http://sfx.scholarsportal.info/mcmaster?sid=OVID:embase&id=pmid:20832689&id=doi:10.1016%2Fj.fm.2010.07.013&issn=0740-0020&isbn=&volume=27&issue=8&spage=1086&pages=1086-1094&date=2010&title=Food+Microbiology&atitle=Interactions+between+bacterial+isolates+from+modified-atmosphere-packaged+artisan-type+cooked+ham+in+view+of+the+development+of+a+bioprotective+culture&aulast=Vasilopoulos&pid=<author>Vasilopoulos+C.%3BDe+Mey+E.%3BDewulf+L.%3BPaelinck+H.%3BDe+Smedt+A.%3BVandendriessche+F.%3BDe+Vuyst+L.%3BLeroy+F.<%2Fauthor><AN>51033257<%2FAN><DT>Article<%2FDT>)

78.

Evaluation of microbial dynamics during the ripening of a traditional Taiwanese naturally fermented ham.

Tu R.-J., Wu H.-Y., Lock Y.-S., Chen M.-J.

Food Microbiology. 27 (4) (pp 460-467), 2010. Date of Publication: June 2010.

AN: 50766520

Isolation and identification of the autochthonous starter from a naturally fermented meat allows control of the fermentation process and promises microbiological safety for this specialty. Thus the purpose of this study was to identify the lactic acid bacteria and coagulase-negative cocci present in a traditional Taiwanese naturally fermented ham (TNFH) and to study the microbial dynamics at different ripening stages; the approach was a combination of conventional microbiological cultivation, polymerase chain reaction-denaturing gradient gel electrophoresis and DNA sequencing. In total, twelve different strains of lactic acid bacteria and three Staphylococcus strains were identified in the TNFH samples, whereas only 5 dominant strains were observed in the TNFH samples when the DGGE as a culture-independent method was applied. The bacterial ecology on the surface of the samples was mainly characterized by the stable presence of Lactobacillus sakei and Staphylococcus saprophyticus; nonetheless Leuconostoc mesenteroides and Carnobacterium divergens were the most abundant bacteria found in the final product. These results are also agreed with the findings of the culture-independent method. In addition, Microbacterium spp., Carnobacterium spp., Enterobacter spp., Brochothrix spp., Enterococcus spp., and Bacillus spp. were also present at the beginning of the ripening, but few bacteria were found at the center of the TNFH samples during the early ripening stages. However, after 30 days of ripening, the microbial ecology at the center of the TNFH samples paralleled that of the surface. Finally, as far as we have been able to determine, our report is the first to investigate the microbiological dynamics in fermented meat products using combination of cultivation, the Harrison disc method, DGGE and DNA sequencing as the culture-dependent method. Our report is also the first to show the presence of Staphylococcus arlettae in a fermented sausage and ham product. © 2009.

PMID

20417394 [<http://www.ncbi.nlm.nih.gov/pubmed/?term=20417394>]

Institution

(Tu, Wu) Livestock Research Institute, Council of Agriculture, Executive Yuan, 112 Muchang, HsinHua, Tainan, Taiwan (Republic of China) (Lock, Chen) Department of Animal Science and Technology, National Taiwan University, No. 50, Ln. 155, Sec. 3, Keelung Rd., Taipei 106, Taiwan (Republic of China)

(Chen) Research Center of Food and Biomolecules, National Taiwan University, No. 50, Ln. 155, Sec. 3, Keelung Rd., Taipei, Taiwan (Republic of China)

Publisher

Academic Press (24-28 Oval Road, London NW1 7DX, United Kingdom)

Emtree Heading

animal; article; bacterial count; culture medium; fermentation; food control; growth, development and aging; human; isolation and purification; *Lactobacillaceae; *meat; microbiology; nucleotide sequence; polymerase chain reaction; population dynamics; pulsed field gel electrophoresis; *Staphylococcus; swine; Taiwan; *bacterial DNA/an [Drug Analysis].

Drug Index Terms

*bacterial DNA / *drug analysis.

Other Index Terms

animal; article; bacterial count; culture medium; fermentation; food control; growth, development and aging; human; isolation and purification; *Lactobacillaceae; *meat; microbiology; nucleotide sequence; polymerase chain reaction; population dynamics; pulsed field gel electrophoresis; *Staphylococcus; swine; Taiwan.

Link to the Ovid Full Text or citation:

[Click here for full text options](https://libaccess.mcmaster.ca/login?url=http://ovidsp.ovid.com/ovidweb.cgi?T=JS&CSC=Y&NEWS=N&PAGE=fulltext&D=emed11&AN=50766520)

Link to the External Link Resolver:

[SFX](http://sfx.scholarsportal.info/mcmaster?sid=OVID:embase&id=pmid:20417394&id=doi:10.1016%2Fj.fm.2009.12.011&issn=0740-0020&isbn=&volume=27&issue=4&spage=460&pages=460-467&date=2010&title=Food+Microbiology&atitle=Evaluation+of+microbial+dynamics+during+the+ripening+of+a+traditional+Taiwanese+naturally+fermented+ham&aulast=Tu&pid=<author>Tu+R.-J.%3BWu+H.-Y.%3BLock+Y.-S.%3BChen+M.-J.<%2Fauthor><AN>50766520<%2FAN><DT>Article<%2FDT>)

79.

Effects of temperature and pH on the growth of bacteria isolated from blown packs of vacuum-packaged beef.

Yang X., Gill C.O., Balamurugan S.

Journal of Food Protection. 72 (11) (pp 2380-2385), 2009. Date of Publication: November 2009.

AN: 355643435

Bacteria recovered from the microflora of blown packs of vacuum-packaged beef were identified as Leuconostoc mesenteroides, Lactococcus lactis, Carnobacterium maltaromaticum, and Clostridium estertheticum, with L. mesenteroides predominant. Isolates of these lactic acid bacteria all grew in peptone yeast extract glucose starch broth (PYGSB) at temperatures between -2 and 30degreeC but generally grew more slowly and over a more restricted temperature range in meat juice medium (MJM). A C. estertheticum isolate and the type strain of C. estertheticum subsp. estertheticum (ATCC 51377) both grew in PYGSB and MJM at similar rates at temperatures between -2 and 17degreeC and grew at 20degreeC in MJM but not in PYGSB. Square root models of the variation of the growth rate with temperature indicated that the C. maltaromaticum isolate and the C. estertheticum strains grew at similar rates that were faster than those of the other isolates at temperatures between -2 and 0degreeC. The L. mesenteroides and L. lactis isolates grew in PYGSB at pH 5.0, but the C. maltaromaticum isolate and both strains of C. estertheticum did not grow in PYGSB at pH < 5.3. C. estertheticum stopped growing in MJM buffered at pH 6.5 when glucose was exhausted, although these bacteria then utilized lactate. The findings suggest that, like carnobacteria, C. estertheticum may predominate during the early stages of development of the spoilage microflora of vacuum-packaged beef but that C. estertheticum will likely be inhibited by a falling pH and so may be only a minor part of the spoilage microflora when maximum numbers are attained. Copyright ©, International Association for Food Protection.

PMID

19903404 [<http://www.ncbi.nlm.nih.gov/pubmed/?term=19903404>]

Institution

(Yang, Gill, Balamurugan) Agriculture and Agri-Food Canada, Lacombe Research Centre, 6000 CandE Trail, Lacombe, AB, T4L 1W1, Canada

Publisher

IAMFES (6200 Aurora Avenue, Suite 200W, Des Moines IA 50322-3863, United States)

Emtree Heading

animal; article; bacterial count; Carnobacterium; cattle; Clostridium; *food contamination/an [Drug Analysis]; food control; food handling; *food packaging; *food preservation; *Gram positive bacterium; growth, development and aging; human; kinetics; Lactococcus lactis; Leuconostoc; *meat; methodology; microbiology; pH; temperature; vacuum.

Other Index Terms

animal; article; bacterial count; Carnobacterium; cattle; Clostridium; *food contamination / *drug analysis; food control; food handling; *food packaging; *food preservation; *Gram positive bacterium; growth, development and aging; human; kinetics; Lactococcus lactis; Leuconostoc; *meat; methodology; microbiology; pH; temperature; vacuum.

Link to the Ovid Full Text or citation:

[Click here for full text options](https://libaccess.mcmaster.ca/login?url=http://ovidsp.ovid.com/ovidweb.cgi?T=JS&CSC=Y&NEWS=N&PAGE=fulltext&D=emed11&AN=355643435)

Link to the External Link Resolver:

[SFX](http://sfx.scholarsportal.info/mcmaster?sid=OVID:embase&id=pmid:19903404&id=doi:10.4315%2F0362-028X-72.11.2380&issn=0362-028X&isbn=&volume=72&issue=11&spage=2380&pages=2380-2385&date=2009&title=Journal+of+Food+Protection&atitle=Effects+of+temperature+and+pH+on+the+growth+of+bacteria+isolated+from+blown+packs+of+vacuum-packaged+beef&aulast=Yang&pid=<author>Yang+X.%3BGill+C.O.%3BBalamurugan+S.<%2Fauthor><AN>355643435<%2FAN><DT>Article<%2FDT>)

80.

Psychrotrophic lactic acid bacteria used to improve the safety and quality of vacuum-packaged cooked and peeled tropical shrimp and cold-smoked salmon.

Matamoros S., Leroi F., Cardinal M., Gigout F., Chadli F.K., Cornet J., Prevost H., Pilet M.F.

Journal of Food Protection. 72 (2) (pp 365-374), 2009. Date of Publication: February 2009.

AN: 354224555

Previously isolated lactic acid bacteria (LAB) from seafood products have been investigated for their capacity to increase the sensory shelf life of vacuum-packaged shrimp and cold-smoked salmon and to inhibit the growth of three pathogenic bacteria. Two different manufactured batches of cooked, peeled, and vacuum-packaged shrimp were inoculated with seven LAB strains separately at an initial level of 5 log CFU g-1, and the spoilage was estimated by sensory analysis after 7 and 28 days of storage at 8degreeC. Two Leuconostoc gelidum strains greatly extended the shelf life of both batches, two Lactococcus piscium strains had a moderate effect, two bacteria were spoilers (Lactobacillus fuchuensis and Carnobacterium alterfunditum), and the last one (another Leuconostoc gelidum strain) showed highly variable results depending on the batch considered. The four strains showing the best results (two Leuconostoc gelidum and two Lactococcus piscium strains) were selected for the same experiment in cold-smoked salmon. In this product, Lactococcus piscium strains showed better inhibiting capacities, improving the sensory quality significantly at 14 and 28 days of storage. Finally, the inhibiting capacities of two strains (one Leuconostoc gelidum strain and one Lactococcus piscium strain) were tested against three pathogenic bacteria (Vibrio cholerae, Listeria monocytogenes, and Staphylococcus aureus) by challenge tests in shrimp. LAB and pathogenic bacteria were coinoculated in vacuum-packaged shrimp and enumerated during 5 weeks. Lactococcus piscium strain EU2241 was able to reduce significantly the number of Listeria monocytogenes and S. aureus organisms in the product by 2 log throughout the study for Listeria monocytogenes and up to 4 weeks for S. aureus. Copyright © International Association for Food Protection.

PMID

19350982 [<http://www.ncbi.nlm.nih.gov/pubmed/?term=19350982>]

Institution

(Matamoros, Prevost, Pilet) UMR INRA 1014 SECAL1M EN1T1AA, Nantes, France (Matamoros, Leroi, Cardinal, Gigout, Chadli, Cornet) Ifremer, Departement de Sciences et Techniques Alimentaires Marines, Nantes, France

Publisher

IAMFES (6200 Aurora Avenue, Suite 200W, Des Moines IA 50322-3863, United States)

Emtree Heading

animal; antibiosis; article; bacterial count; food control; food handling; *food packaging; food preservation; growth, development and aging; human; *Lactococcus; *Leuconostoc; Listeria monocytogenes; methodology; microbiology; *Penaeidae; physiology; safety; *salmon; *sea food; *shellfish; standard; Staphylococcus aureus; taste; temperature; time; vacuum; Vibrio cholerae.

Other Index Terms

animal; antibiosis; article; bacterial count; food control; food handling; *food packaging; food preservation; growth, development and aging; human; *Lactococcus; *Leuconostoc; Listeria monocytogenes; methodology; microbiology; *Penaeidae; physiology; safety; *salmon; *sea food; *shellfish; standard; Staphylococcus aureus; taste; temperature; time; vacuum; Vibrio cholerae.

Link to the Ovid Full Text or citation:

[Click here for full text options](https://libaccess.mcmaster.ca/login?url=http://ovidsp.ovid.com/ovidweb.cgi?T=JS&CSC=Y&NEWS=N&PAGE=fulltext&D=emed11&AN=354224555)

Link to the External Link Resolver:

[SFX](http://sfx.scholarsportal.info/mcmaster?sid=OVID:embase&id=pmid:19350982&id=doi:10.4315%2F0362-028X-72.2.365&issn=0362-028X&isbn=&volume=72&issue=2&spage=365&pages=365-374&date=2009&title=Journal+of+Food+Protection&atitle=Psychrotrophic+lactic+acid+bacteria+used+to+improve+the+safety+and+quality+of+vacuum-packaged+cooked+and+peeled+tropical+shrimp+and+cold-smoked+salmon&aulast=Matamoros&pid=<author>Matamoros+S.%3BLeroi+F.%3BCardinal+M.%3BGigout+F.%3BChadli+F.K.%3BCornet+J.%3BPrevost+H.%3BPilet+M.F.<%2Fauthor><AN>354224555<%2FAN><DT>Article<%2FDT>)

81.

Comparison of culture-dependent and independent techniques for characterisation of the microflora of peroxyacetic acid treated, vacuum-packaged beef.

Brightwell G., Clemens R., Adam K., Urlich S., Boerema J.

Food Microbiology. 26 (3) (pp 283-288), 2009. Date of Publication: May 2009.

AN: 50412280

The diversity of microflora associated with peroxyacetic acid (POAA) treated and untreated beef was investigated by 16S rDNA gene cloning, DGGE analysis and conventional bacterial cultivation. Following vacuum packaging, POAA treated and untreated meat samples were stored for up to 18 weeks at -1.5 degreeC. Each culture independent method showed Carnobacterium spp. to predominate on both POAA treated and untreated meat. However, 16S rDNA gene analysis also detected the presence of psychrotolerant Clostridium spp. in the POAA-treated beef. Culture-dependent analysis did not distinguish Carnobacterium spp. from Lactobacilli. Although culture-dependent analysis showed an increase in the ratio of Enterobacteriaceae to lactic acid bacteria from weeks 6-18 in the POAA treated compared with the untreated meat, the numbers of Enterobacteriaceae were significantly less on POAA treated than on untreated meat. The combination of data collected by culture-dependent and independent techniques provided the most robust approach for elucidating the efficacy of chemical sanitization of chilled vacuum-packaged beef. If conventional cultivation is used for monitoring bacterial spoilage of vacuum-packaged chilled meats it is recommended that culture methods specific for Carnobacterium and Clostridium spp. should be included in order to provide a more complete indication of microbial diversity. © 2009 Elsevier Ltd. All rights reserved.

PMID

19269570 [<http://www.ncbi.nlm.nih.gov/pubmed/?term=19269570>]

Institution

(Brightwell, Clemens, Adam, Urlich) Food Metabolism and Microbiology, AgResearch, Ruakura MIRINZ Centre, Private Bag 3123, Hamilton, Waikato, New Zealand (Boerema) Inghams Enterprises (N.Z.) Limited, Ngarua, New Zealand

Publisher

Academic Press (24-28 Oval Road, London NW1 7DX, United Kingdom)

Emtree Heading

animal; article; *bacterial count; cattle; chemistry; Clostridium; comparative study; DNA sequence; drug effect; *food contamination/an [Drug Analysis]; *food contamination/pc [Prevention]; food packaging; *food preservation; genetics; growth, development and aging; human; Lactobacillus; *meat; methodology; microbiology; polyacrylamide gel electrophoresis; temperature; time; vacuum; bacterial DNA; *disinfectant agent/pd [Pharmacology]; *peracetic acid/pd [Pharmacology]; ribosome DNA.

Drug Index Terms

bacterial DNA; *disinfectant agent / *pharmacology; *peracetic acid / *pharmacology; ribosome DNA.

Other Index Terms

animal; article; *bacterial count; cattle; chemistry; Clostridium; comparative study; DNA sequence; drug effect; *food contamination / *drug analysis / *prevention; food packaging; *food preservation; genetics; growth, development and aging; human; Lactobacillus; *meat; methodology; microbiology; polyacrylamide gel electrophoresis; temperature; time; vacuum.

Link to the Ovid Full Text or citation:

[Click here for full text options](https://libaccess.mcmaster.ca/login?url=http://ovidsp.ovid.com/ovidweb.cgi?T=JS&CSC=Y&NEWS=N&PAGE=fulltext&D=emed11&AN=50412280)

Link to the External Link Resolver:

[SFX](http://sfx.scholarsportal.info/mcmaster?sid=OVID:embase&id=pmid:19269570&id=doi:10.1016%2Fj.fm.2008.12.010&issn=0740-0020&isbn=&volume=26&issue=3&spage=283&pages=283-288&date=2009&title=Food+Microbiology&atitle=Comparison+of+culture-dependent+and+independent+techniques+for+characterisation+of+the+microflora+of+peroxyacetic+acid+treated%2C+vacuum-packaged+beef&aulast=Brightwell&pid=<author>Brightwell+G.%3BClemens+R.%3BAdam+K.%3BUrlich+S.%3BBoerema+J.<%2Fauthor><AN>50412280<%2FAN><DT>Article<%2FDT>)

82.

Divercin V41 from gene characterization to food applications: 1998-2008, a decade of solved and unsolved questions.

Rihakova J., Belguesmia Y., Petit V.W., Pilet M.F., Prevost H., Dousset X., Drider D.

Letters in Applied Microbiology. 48 (1) (pp 1-7), 2009. Date of Publication: January 2009.

AN: 354047294

Summary The emergence of an increasing number of antibiotic resistant human clinical bacteria has been a great cause of concern for the last decades. As an example, Staphylococcus aureus isolates in the hospital environment are becoming more and more resistant to antibiotics including vancomycin which is considered as a last line of defence in treatment of Staphylococcus aureus-resistant methicillin. On the other hand, food safety is threatened by development of pathogenic bacteria including Listeria monocytogenes, Campylobacter jejuni, Salmonella enteritidis, Escherichia coli O157:H7 and Staphylococcus aureus. The use of antimicrobial peptides such as glycopeptides, semi-synthetic peptides, bacteriocins including lantibiotics offers a hope to face these clinical and food microbiology concerns. Clinical approval of new chemotherapeutic agents requires a long period of time. Research on bacteriocins has demonstrated potential use to fight against undesired foodborne pathogens but the use industrial use of bacteriocins is limited. To date only lantibiotic nisin and in class IIa bacteriocin Pediocin PA-1 are legally used as food preservative in many countries. The present minireview is focused on divercin V41 (DvnV41), a class IIa bacteriocin naturally produced by Carnobacterium divergens V41. The last decade has been the witness of intensive investigations carried out on this cationic peptide tempting to answer multiple questions covering basic and applied aspects. DvnV41 has shown a wide spectrum of activity either alone or in combination with nisin and/or polymixins (synergistic effect). This outcome indicates that Cb. divergens V41 could potentially be used for safe and efficient prevention of L. monocytogenes growth in cold smoked salmon. © 2008 The Society for Applied Microbiology.

PMID

19018960 [<http://www.ncbi.nlm.nih.gov/pubmed/?term=19018960>]

Institution

(Drider) Laboratoire de Microbiologie, ENITIAA, Rue de la Geraudiere, BP 82225, 44322 Nantes Cedex 3, France (Rihakova, Belguesmia, Drider) Laboratoire de Microbiologie, ENITIAA, Rue de la Geraudiere, Nantes Cedex, France

(Rihakova) Department of Biochemistry and Microbiology, Institute of Chemical Technology, Prague, Czech Republic

(Petit) Chemistry and Biochemistry of Natural Substances, UMR 5154 CNRS-National Museum of Natural History, Department Regulations, Development and Molecular Diversity, Paris, France

(Rihakova, Belguesmia, Pilet, Prevost, Dousset) UMR INRA 1014 SECALIM ENITIAA, Rue de la Geraudiere, Nantes Cedex, France

Publisher

Blackwell Publishing Ltd (9600 Garsington Road, Oxford OX4 2XG, United Kingdom)

Emtree Heading

article; bacterial growth; Carnobacterium; food safety; gene expression; gene function; gene structure; *genetically modified food; Listeria monocytogenes; nonhuman; bacteriocin; food preservative; lantibiotic; pediocin; polymyxin.

Drug Index Terms

bacteriocin; food preservative; lantibiotic; pediocin; polymyxin.

Other Index Terms

article; bacterial growth; Carnobacterium; food safety; gene expression; gene function; gene structure; *genetically modified food; Listeria monocytogenes; nonhuman.

Link to the Ovid Full Text or citation:

[Click here for full text options](https://libaccess.mcmaster.ca/login?url=http://ovidsp.ovid.com/ovidweb.cgi?T=JS&CSC=Y&NEWS=N&PAGE=fulltext&D=emed11&AN=354047294)

Link to the External Link Resolver:

[SFX](http://sfx.scholarsportal.info/mcmaster?sid=OVID:embase&id=pmid:19018960&id=doi:10.1111%2Fj.1472-765X.2008.02490.x&issn=0266-8254&isbn=&volume=48&issue=1&spage=1&pages=1-7&date=2009&title=Letters+in+Applied+Microbiology&atitle=Divercin+V41+from+gene+characterization+to+food+applications%3A+1998-2008%2C+a+decade+of+solved+and+unsolved+questions&aulast=Rihakova&pid=<author>Rihakova+J.%3BBelguesmia+Y.%3BPetit+V.W.%3BPilet+M.F.%3BPrevost+H.%3BDousset+X.%3BDrider+D.<%2Fauthor><AN>354047294<%2FAN><DT>Article<%2FDT>)

83.

Interactions between two carnobacteriocins Cbn BM1 and Cbn B2 from Carnobacterium maltaromaticum CP5 on target bacteria and Caco-2 cells.

Jasniewski J., Cailliez-Grimal C., Chevalot I., Milliere J.-B., Revol-Junelles A.-M.

Food and Chemical Toxicology. 47 (4) (pp 893-897), 2009. Date of Publication: April 2009.

AN: 50429229

Two purified class IIa carnobacteriocins Cbn BM1 and Cbn B2, from Carnobacterium maltaromaticum CP5, were evaluated for antimicrobial activity against pathogenic, spoilage and lactic acid bacteria. Then, the presence of a synergistic mode of action of these two carnobacteriocins on Listeria sp., Enterococcus sp. and Carnobacterium sp. was investigated. A synergistic mode of action between Cbn BM1 and Cbn B2 on sensitive target bacteria was demonstrated using the FIC index method. Combinations of carnobacteriocins enhanced their antibacterial activities and MICs were significantly reduced, between 2- and 15-fold, by the addition of the second bacteriocin. To improve the safety of the bacteriocins as biopreservative agents, the cytotoxicity of the combination of theses two bacteriocins was determined on Caco-2 cell line. However, these two peptides used alone or in combination, at concentration 100-fold higher than those required for antimicrobial activity, were not cytotoxic. This suggests that the two carnobacteriocins produced by C. maltaromaticum CP5 could be potential natural agents for food preservation. © 2009 Elsevier Ltd. All rights reserved.

PMID

19271288 [<http://www.ncbi.nlm.nih.gov/pubmed/?term=19271288>]

Institution

(Jasniewski, Cailliez-Grimal, Milliere, Revol-Junelles) Nancy-Universite, Institut National Polytechnique de Lorraine, Laboratoire de Science et Genie Alimentaires, 2 Avenue de la Foret de Haye, 54505 Vandoeuvre-les-Nancy, France (Chevalot) Nancy-Universite, Institut National Polytechnique de Lorraine, Laboratoire des Science du Genie Chimique, 2 Avenue de la Foret de Haye, 54505 Vandoeuvre-les-Nancy, France

Publisher

Elsevier Ltd (Langford Lane, Kidlington, Oxford OX5 1GB, United Kingdom)

Emtree Heading

antimicrobial activity; article; *Carnobacterium maltaromaticum; cell strain CACO 2; cytotoxicity; Enterococcus; human; human cell; lactic acid bacterium; Listeria; nonhuman; preservation; *protein interaction; safety; *bacteriocin; preservative; unclassified drug; *carnobacteriocin b2; *carnobacteriocin bm1.

Candidate Terms

*carnobacteriocin B2 [drug term]; *carnobacteriocin BM1 [drug term].

Drug Index Terms

*bacteriocin; preservative; unclassified drug.

Other Index Terms

antimicrobial activity; article; *Carnobacterium maltaromaticum; cell strain CACO 2; cytotoxicity; Enterococcus; human; human cell; lactic acid bacterium; Listeria; nonhuman; preservation; *protein interaction; safety.

Link to the Ovid Full Text or citation:

[Click here for full text options](https://libaccess.mcmaster.ca/login?url=http://ovidsp.ovid.com/ovidweb.cgi?T=JS&CSC=Y&NEWS=N&PAGE=fulltext&D=emed11&AN=50429229)

Link to the External Link Resolver:

[SFX](http://sfx.scholarsportal.info/mcmaster?sid=OVID:embase&id=pmid:19271288&id=doi:10.1016%2Fj.fct.2009.01.025&issn=0278-6915&isbn=&volume=47&issue=4&spage=893&pages=893-897&date=2009&title=Food+and+Chemical+Toxicology&atitle=Interactions+between+two+carnobacteriocins+Cbn+BM1+and+Cbn+B2+from+Carnobacterium+maltaromaticum+CP5+on+target+bacteria+and+Caco-2+cells&aulast=Jasniewski&pid=<author>Jasniewski+J.%3BCailliez-Grimal+C.%3BChevalot+I.%3BMilliere+J.-B.%3BRevol-Junelles+A.-M.<%2Fauthor><AN>50429229<%2FAN><DT>Article<%2FDT>)

84.

Biological approaches for controlling shellfish-associated pathogens.

Teplitski M., Wright A.C., Lorca G.

Current Opinion in Biotechnology. 20 (2) (pp 185-190), 2009. Date of Publication: April 2009.

AN: 354629634

As the consumption of seafood and shellfish increases around the world, so is the incidence of associated outbreaks of illness. Various postharvest treatments are effective at killing seafood-associated bacteria, but most of these treatments also kill the mollusks. Because consumer preferences for raw live shellfish persist, biological approaches for promoting microbiological safety of live product are being considered. Applications of probiotic bacteria to reduce human pathogens in live shellfish could augment current practices for preharvest monitoring of water quality. Postharvest, biological controls will be important to remove shellfish-associated commensal Vibrio spp. that are pathogenic to humans. Further investigations will reveal whether combining depuration with chemical disruption of bacterial attachment or cell-to-cell signaling may accomplish this goal. © 2009 Elsevier Ltd. All rights reserved.

PMID

19342220 [<http://www.ncbi.nlm.nih.gov/pubmed/?term=19342220>]

Institution

(Teplitski) Department of Soil and Water Science, University of Florida-Institute of Food and Agricultural Sciences (IFAS), Gainesville, FL 32611, United States (Wright) Department of Food Science and Human Nutrition, University of Florida-IFAS, Gainesville, FL 32611, United States

(Teplitski, Lorca) Department of Microbiology and Cell Science, University of Florida-IFAS, Gainesville, FL 32611, United States

Publisher

Elsevier Ltd (Langford Lane, Kidlington, Oxford OX5 1GB, United Kingdom)

Emtree Heading

Actinomyces; Aeromonas; Bacillus subtilis; bacterial colonization; *biological pest control; Carnobacterium; Clostridium butyricum; commensal; Edwardsiella tarda; Enterococcus faecium; epidemic; food intake; gene control; gene disruption; human; Lactobacillus acidophilus; Listonella anguillarum; methodology; mollusc; monitoring; nonhuman; pathogenesis; Plesiomonas; postharvest period; priority journal; raw food; review; Saccharomyces cerevisiae; Salmonella enterica; sea food; *shellfish; Staphylococcus aureus; Vibrio cholerae; Vibrio parahaemolyticus; Vibrio vulnificus; vibriophage; water quality; antibiotic agent; arginine; chitosan; polysaccharide sulfate; probiotic agent; unclassified drug; probiotic bacillus toyoi.

Candidate Terms

probiotic bacillus toyoi [drug term].

Drug Index Terms

antibiotic agent; arginine; chitosan; polysaccharide sulfate; probiotic agent; unclassified drug.

Other Index Terms

Actinomyces; Aeromonas; Bacillus subtilis; bacterial colonization; *biological pest control; Carnobacterium; Clostridium butyricum; commensal; Edwardsiella tarda; Enterococcus faecium; epidemic; food intake; gene control; gene disruption; human; Lactobacillus acidophilus; Listonella anguillarum; methodology; mollusc; monitoring; nonhuman; pathogenesis; Plesiomonas; postharvest period; priority journal; raw food; review; Saccharomyces cerevisiae; Salmonella enterica; sea food; *shellfish; Staphylococcus aureus; Vibrio cholerae; Vibrio parahaemolyticus; Vibrio vulnificus; vibriophage; water quality.

Link to the Ovid Full Text or citation:

[Click here for full text options](https://libaccess.mcmaster.ca/login?url=http://ovidsp.ovid.com/ovidweb.cgi?T=JS&CSC=Y&NEWS=N&PAGE=fulltext&D=emed11&AN=354629634)

Link to the External Link Resolver:

[SFX](http://sfx.scholarsportal.info/mcmaster?sid=OVID:embase&id=pmid:19342220&id=doi:10.1016%2Fj.copbio.2009.03.001&issn=0958-1669&isbn=&volume=20&issue=2&spage=185&pages=185-190&date=2009&title=Current+Opinion+in+Biotechnology&atitle=Biological+approaches+for+controlling+shellfish-associated+pathogens&aulast=Teplitski&pid=<author>Teplitski+M.%3BWright+A.C.%3BLorca+G.<%2Fauthor><AN>354629634<%2FAN><DT>Review<%2FDT>)

85.

Interaction of dietary resveratrol with animal-associated bacteria.

Jung C.M., Heinze T.M., Schnackenberg L.K., Mullis L.B., Elkins S.A., Elkins C.A., Steele R.S., Sutherland J.B.

FEMS Microbiology Letters. 297 (2) (pp 266-273), 2009. Date of Publication: August 2009.

AN: 355060147

Resveratrol (3,5,4'-trihydroxy-trans-stilbene), an antifungal phytoalexin produced by grapes, peanuts, and Japanese knotweeds, is thought to be a beneficial dietary phytochemical in red wine and grape juice. Information on its antibacterial properties and biotransformation, however, is limited. We surveyed the interactions of resveratrol with 43 strains of bacterial species that are often animal- or human-associated. Resveratrol at 50 mg L-1 reduced the growth rates of most of the bacteria tested, but did not totally prevent growth even at much higher levels. Eleven of the 43 bacteria were capable of transforming at least 20% of the resveratrol. Three major metabolites were identified as resveratroloside, piceid, and dihydroresveratrol, and three other metabolites were partially characterized. © 2009 Federation of European Microbiological Societies.

PMID

19566680 [<http://www.ncbi.nlm.nih.gov/pubmed/?term=19566680>]

Institution

(Jung) US Army Engineer Research and Development Center, Vicksburg, MS, United States (Heinze) National Center for Toxicological Research, Division of Biochemical Toxicology, US Food and Drug Administration, Jefferson, AR, United States

(Schnackenberg) National Center for Toxicological Research, Division of Systems Toxicology, US Food and Drug Administration, Jefferson, AR, United States

(Mullis, Elkins, Steele, Sutherland) National Center for Toxicological Research, Division of Microbiology, US Food and Drug Administration, 3900 NCTR Road, Jefferson, AR 72079, United States

(Elkins) Center for Food Safety and Applied Nutrition, US Food and Drug Administration, Laurel, MD, United States

Publisher

Blackwell Publishing Ltd (9600 Garsington Road, Oxford OX4 2XG, United Kingdom)

Emtree Heading

Achromobacter; article; Bacillus; bacterial growth; bacterial strain; bacterium transformation; Bacteroides; Carnobacterium maltaromaticum; Clostridium; controlled study; Eggerthella; Enterobacter; Escherichia coli; growth inhibition; growth rate; Klebsiella; Lactobacillus; Lactococcus; Micrococcus; minimum inhibitory concentration; *molecular interaction; nonhuman; priority journal; Propionibacterium; Proteus; Pseudomonas aeruginosa; Pseudomonas putida; Ruminococcus; Salmonella; Staphylococcus epidermidis; Streptococcus; Yersinia; drug metabolite; piceid; *resveratrol.

Drug Index Terms

drug metabolite; piceid; *resveratrol.

Other Index Terms

Achromobacter; article; Bacillus; bacterial growth; bacterial strain; bacterium transformation; Bacteroides; Carnobacterium maltaromaticum; Clostridium; controlled study; Eggerthella; Enterobacter; Escherichia coli; growth inhibition; growth rate; Klebsiella; Lactobacillus; Lactococcus; Micrococcus; minimum inhibitory concentration; *molecular interaction; nonhuman; priority journal; Propionibacterium; Proteus; Pseudomonas aeruginosa; Pseudomonas putida; Ruminococcus; Salmonella; Staphylococcus epidermidis; Streptococcus; Yersinia.

Link to the Ovid Full Text or citation:

[Click here for full text options](https://libaccess.mcmaster.ca/login?url=http://ovidsp.ovid.com/ovidweb.cgi?T=JS&CSC=Y&NEWS=N&PAGE=fulltext&D=emed11&AN=355060147)

Link to the External Link Resolver:

[SFX](http://sfx.scholarsportal.info/mcmaster?sid=OVID:embase&id=pmid:19566680&id=doi:10.1111%2Fj.1574-6968.2009.01691.x&issn=0378-1097&isbn=&volume=297&issue=2&spage=266&pages=266-273&date=2009&title=FEMS+Microbiology+Letters&atitle=Interaction+of+dietary+resveratrol+with+animal-associated+bacteria&aulast=Jung&pid=<author>Jung+C.M.%3BHeinze+T.M.%3BSchnackenberg+L.K.%3BMullis+L.B.%3BElkins+S.A.%3BElkins+C.A.%3BSteele+R.S.%3BSutherland+J.B.<%2Fauthor><AN>355060147<%2FAN><DT>Article<%2FDT>)

86.

Use of probiotic Lactobacillus preparation to prevent diarrhoea associated with antibiotics: Randomised double blind placebo controlled trial.

Hickson M., D'Souza A.L., Muthu N., Rogers T.R., Want S., Rajkumar C., Bulpitt C.J.

British Medical Journal. 335 (7610) (pp 80-83), 2007. Date of Publication: 14 Jul 2007.

AN: 47123282

Objective: To determine the efficacy of a probiotic drink containing Lactobacillus for the prevention of any diarrhoea associated with antibiotic use and that caused by Clostridium difficile.

Design(s): Randomised double blind placebo controlled study.

Participant(s): 135 hospital patients (mean age 74) taking antibiotics. Exclusions included diarrhoea on admission, bowel pathology that could result in diarrhoea, antibiotic use in the previous four weeks, severe illness, immunosuppression, bowel surgery, artificial heart valves, and history of rheumatic heart disease or infective endocarditis.

Intervention(s): Consumption of a 100 g (97 ml) drink containing Lactobacillus casei, L bulgaricus, and Streptococcus thermophilus twice a day during a course of antibiotics and for one week after the course finished. The placebo group received a longlife sterile milkshake.

Main Outcome Measure(s): Primary outcome: occurrence of antibiotic associated diarrhoea. Secondary outcome: presence of C difficile toxin and diarrhoea.

Result(s): 7/57 (12%) of the probiotic group developed diarrhoea associated with antibiotic use compared with 19/56 (34%) in the placebo group (P=0.007). Logistic regression to control for other factors gave an odds ratio 0.25 (95% confidence interval 0.07 to 0.85) for use of the probiotic, with low albumin and sodium also increasing the risk of diarrhoea. The absolute risk reduction was 21.6% (6.6% to 36.6%), and the number needed to treat was 5 (3 to 15). No one in the probiotic group and 9/53 (17%) in the placebo group had diarrhoea caused by C difficile (P=0.001). The absolute risk reduction was 17% (7% to 27%), and the number needed to treat was 6 (4 to 14).

Conclusion(s): Consumption of a probiotic drink containing L casei, L bulgaricus, and S thermophilus reduce the incidence of antibiotic associated diarrhoea and C difficile associated diarrhoea. This has the potential to decrease morbidity, healthcare costs, and mortality if used routinely in patients aged over 50. Trial registration: National Research Register N0016106821.

PMID

17604300 [<http://www.ncbi.nlm.nih.gov/pubmed/?term=17604300>]

Institution

(Hickson) Nutrition and Dietetic Research Group, Faculty of Medicine, Imperial College, London W12 0HS, United Kingdom (D'Souza, Rajkumar, Bulpitt) Medicine for the Elderly, Faculty of Medicine, Imperial College, London, United Kingdom

(Muthu) Hillingdon Hospital, Uxbridge, United Kingdom

(Rogers) Department of Infectious Diseases and Immunity, Faculty of Medicine, Imperial College School of Medicine, London, United Kingdom

(Want) Microbiology Department, Hammersmith Hospital NHS Trust, London, United Kingdom

(Hickson) Department of Nutrition and Dietetics, Charing Cross Hospital, London W6 8RF, United Kingdom

Publisher

BMJ Publishing Group (Tavistock Square, London WC1H 9JR, United Kingdom)

Emtree Heading

article; Carnobacterium maltaromaticum; clinical trial; controlled clinical trial; controlled study; *diarrhea/dt [Drug Therapy]; *diarrhea/pc [Prevention]; double blind procedure; drug cost; drug efficacy; human; Lactobacillus; major clinical study; milk; priority journal; randomized controlled trial; Streptococcus thermophilus; antibiotic agent/ct [Clinical Trial]; antibiotic agent/dt [Drug Therapy]; placebo; probiotic agent/ct [Clinical Trial]; probiotic agent/dt [Drug Therapy].

Drug Index Terms

antibiotic agent / clinical trial / drug therapy; placebo; probiotic agent / clinical trial / drug therapy.

Other Index Terms

article; Carnobacterium maltaromaticum; clinical trial; controlled clinical trial; controlled study; *diarrhea / *drug therapy / *prevention; double blind procedure; drug cost; drug efficacy; human; Lactobacillus; major clinical study; milk; priority journal; randomized controlled trial; Streptococcus thermophilus.

Link to the Ovid Full Text or citation:

[Click here for full text options](https://libaccess.mcmaster.ca/login?url=http://ovidsp.ovid.com/ovidweb.cgi?T=JS&CSC=Y&NEWS=N&PAGE=fulltext&D=emed10&AN=47123282)

Link to the External Link Resolver:

[SFX](http://sfx.scholarsportal.info/mcmaster?sid=OVID:embase&id=pmid:17604300&id=doi:10.1136%2Fbmj.39231.599815.55&issn=0959-8146&isbn=&volume=335&issue=7610&spage=80&pages=80-83&date=2007&title=British+Medical+Journal&atitle=Use+of+probiotic+Lactobacillus+preparation+to+prevent+diarrhoea+associated+with+antibiotics%3A+Randomised+double+blind+placebo+controlled+trial&aulast=Hickson&pid=<author>Hickson+M.%3BD'Souza+A.L.%3BMuthu+N.%3BRogers+T.R.%3BWant+S.%3BRajkumar+C.%3BBulpitt+C.J.<%2Fauthor><AN>47123282<%2FAN><DT>Article<%2FDT>)

87.

Quality changes during refrigerated storage of MA-packaged pre-rigor fillets of farmed atlantic cod (Gadus morhua L.) using traditional MAP, CO 2 emitter, and vacuum.

Hansen A.A., Morkore T., Rudi K., Olsen E., Eie T.

Journal of Food Science. 72 (9) (pp M423-M430), 2007. Date of Publication: November/December 2007.

AN: 350150717

Quality changes during 3 wk of refrigerated storage (1.3degreeC) were studied on pre-rigor filleted farmed Atlantic cod packed in modified atmosphere (MAP, 60% CO2 and 40% O2) or vacuum. The packages of MAP contained either a CO2 emitter and low gas volume to product volume (g/p ratio) of 1.3, or a 3.9 g/p ratio and no emitter. The CO2 level remained stable or increased in the packages with CO2 emitter, whereas the CO2 level in the packages with no CO2 emitter decreased to 40% after 4 d of refrigerated storage. High levels of oxygen in the gas mixture prevented formation of trimethyl amine (TMA) during storage of the MA-packed fish, whereas the TMA content increased significantly after 10-d storage in vacuum. MA-packed samples had the highest values of 1-penten-3-ol. Sensory scores of sour, sulfur, and pungent odors were significantly higher for vacuum-packed cod compared to the 2 MA-packaging methods measured 14 d after slaughtering. No differences in sensory scores were observed between the 2 methods of MAP, and shelf life of these samples seemed to be 14 to 21 d. Cod samples packaged in vacuum packages had higher pH values compared to ordinary MAP and packages containing a CO2 emitter. Bacterial growth was inhibited by MAP and resulted at the end of the storage period in dominance of Carnobacterium and some Photobacterium. In MA packages with high O2 levels the Photobacterium was inhibited. It is concluded that CO2 emitters are well suited for reduction of transport volume for MA-packaged farmed cod. © 2007 Institute of Food Technologists.

PMID

18034737 [<http://www.ncbi.nlm.nih.gov/pubmed/?term=18034737>]

Institution

(Hansen, Rudi, Olsen, Eie) MATFORSK AS, Norwegian Food Research Inst., N-1430 As, Norway (Hansen, Morkore) Dept. of Animal and Aquacultural Sciences, Norwegian Univ. of Life Sciences, N-1432 As, Norway

(Morkore) AKVAFORSK AS, Inst. of Aquaculture Research, N-1432 As, Norway

(Rudi) Hedmark Univ. College, N-2418 Elverum, Norway

(Olsen, Eie) Dept. of Chemistry, Biotechnology and Food Science, Norwegian Univ. of Life Sciences, N-1432 As, Norway

Publisher

Blackwell Publishing Inc. (350 Main Street, Malden MA 02148, United States)

Emtree Heading

analysis of variance; animal; article; *Atlantic cod; bacterial count; evaluation; *food handling; *food packaging; *freezing; growth, development and aging; human; instrumentation; metabolism; methodology; odor; pH; Photobacterium; physiology; quality control; time; vacuum; *carbon dioxide; methylamine; *oxygen; trimethylamine.

Drug Index Terms

*carbon dioxide; methylamine; *oxygen; trimethylamine.

Other Index Terms

analysis of variance; animal; article; *Atlantic cod; bacterial count; evaluation; *food handling; *food packaging; *freezing; growth, development and aging; human; instrumentation; metabolism; methodology; odor; pH; Photobacterium; physiology; quality control; time; vacuum.

Link to the Ovid Full Text or citation:

[Click here for full text options](https://libaccess.mcmaster.ca/login?url=http://ovidsp.ovid.com/ovidweb.cgi?T=JS&CSC=Y&NEWS=N&PAGE=fulltext&D=emed10&AN=350150717)

Link to the External Link Resolver:

[SFX](http://sfx.scholarsportal.info/mcmaster?sid=OVID:embase&id=pmid:18034737&id=doi:10.1111%2Fj.1750-3841.2007.00561.x&issn=0022-1147&isbn=&volume=72&issue=9&spage=M423&pages=M423-M430&date=2007&title=Journal+of+Food+Science&atitle=Quality+changes+during+refrigerated+storage+of+MA-packaged+pre-rigor+fillets+of+farmed+atlantic+cod+(Gadus+morhua+L.)+using+traditional+MAP%2C+CO+2+emitter%2C+and+vacuum&aulast=Hansen&pid=<author>Hansen+A.A.%3BMorkore+T.%3BRudi+K.%3BOlsen+E.%3BEie+T.<%2Fauthor><AN>350150717<%2FAN><DT>Article<%2FDT>)

88.

Updating the importance of lactic acid bacteria in fish farming: Natural occurrence and probiotic treatments.

Gatesoupe F.-J.

Journal of Molecular Microbiology and Biotechnology. 14 (1-3) (pp 107-114), 2008. Date of Publication: October 2007.

AN: 350022339

Many recent papers have deepened the state of knowledge about lactic acid bacteria (LAB) in fish gut. In spite of high variability in fish microbiota, LAB are sometimes abundant in the intestine, notably in freshwater fish. Several strains of Streptococcus are pathogenic to fish. Streptococcus iniae and Lactococcus garvieae are major fish pathogens, against which commercial vaccines are available. Fortunately, most LAB are harmless, and some strains have been reported for beneficial effects on fish health. A major step forward in recent years was the converging evidence that LAB can stimulate the immune system in fish. An open question is whether viability can affect immunostimulation. The issue is crucial to commercialize live probiotics rather than inactivated preparations or extracts. There has been a regain of interest in allochthonous strains used as probiotics for terrestrial animals or humans, due to economical and regulatory constraints, but the short survival in sea water may limit application to marine fish. If viability is required, alternative treatments may include the incorporation of prebiotics in feed, and other dietary manipulations that could promote intestinal LAB. Antagonism to pathogens is the other main feature of candidate probiotics, and there are many reports concerning mainly carnobacteria and Enterococcus. Some bacteriocins were characterized which may be of interest not only for aquaculture, but also for food preservation. Copyright © 2008 S. Karger AG.

PMID

17957117 [<http://www.ncbi.nlm.nih.gov/pubmed/?term=17957117>]

Institution

(Gatesoupe) INRA-Ifremer, Joint Research Unit for Fish Nutrition, Aquaculture and Genomics, Plouzane, France (Gatesoupe) Ifremer, Centre de Brest, BP 70, FR-29280 Plouzane, France

Publisher

S. Karger AG (Allschwilerstrasse 10, P.O. Box, Basel CH-4009, Switzerland)

Emtree Heading

aquaculture; article; bacterial strain; Carnobacterium; diet supplementation; drug mechanism; drug synthesis; Enterococcus; fish; human; immune system; immunization; immunostimulation; lactic acid bacterium; Lactococcus; nonhuman; pathogenesis; pisciculture; Streptococcus iniae; survival; bacteriocin; *probiotic agent/dv [Drug Development]; *probiotic agent/pd [Pharmacology]; sea water; vaccine.

Drug Index Terms

bacteriocin; *probiotic agent / *drug development / *pharmacology; sea water; vaccine.

Other Index Terms

aquaculture; article; bacterial strain; Carnobacterium; diet supplementation; drug mechanism; drug synthesis; Enterococcus; fish; human; immune system; immunization; immunostimulation; lactic acid bacterium; Lactococcus; nonhuman; pathogenesis; pisciculture; Streptococcus iniae; survival.

Link to the Ovid Full Text or citation:

[Click here for full text options](https://libaccess.mcmaster.ca/login?url=http://ovidsp.ovid.com/ovidweb.cgi?T=JS&CSC=Y&NEWS=N&PAGE=fulltext&D=emed10&AN=350022339)

Link to the External Link Resolver:

[SFX](http://sfx.scholarsportal.info/mcmaster?sid=OVID:embase&id=pmid:17957117&id=doi:10.1159%2F000106089&issn=1464-1801&isbn=&volume=14&issue=1-3&spage=107&pages=107-114&date=2008&title=Journal+of+Molecular+Microbiology+and+Biotechnology&atitle=Updating+the+importance+of+lactic+acid+bacteria+in+fish+farming%3A+Natural+occurrence+and+probiotic+treatments&aulast=Gatesoupe&pid=<author>Gatesoupe+F.-J.<%2Fauthor><AN>350022339<%2FAN><DT>Article<%2FDT>)

89.

Limitations in the use of Drosophila melanogaster as a model host for gram-positive bacterial infection.

Jensen R.L., Pedersen K.S., Loeschcke V., Ingmer H., Leisner J.J.

Letters in Applied Microbiology. 44 (2) (pp 218-223), 2007. Date of Publication: February 2007.

AN: 46146595

Aims: To examine sensitivities of various Drosophila melanogaster strains towards human pathogenic and nonpathogenic gram-positive bacteria.

Methods and Results: The D. melanogaster Oregon R strain was infected by injecting the thorax with a needle containing Escherichia coli (negative control), Listeria monocytogenes, Staphylococcus aureus (both food-borne pathogens), Listeria innocua, Bacillus subtilis, Carnobacterium maltaromaticum, Lactobacillus plantarum or Pediococcus acidilactici (all nonpathogenic bacteria). Listeria monocytogenes and S. aureus killed the host rapidly compared with the negative control. Infection with L. innocua, B. subtilis or C. maltaromaticum also resulted in a high fly mortality, whereas Lact. plantarum and P. acidilactici resulted in a slightly increased mortality. Four additional D. melanogaster lines, three of which had been selected for heat, cold and desiccation resistance respectively, were subjected to infection by L. monocytogenes, S. aureus and E. coli. Mortality rates were comparable with that of the Oregon R strain.

Conclusion(s): Use of the injection method shows the limitation of D. melanogaster as a model host for gram-positive bacteria as opportunistic infection by nonpathogenic gram-positive bacteria results in partial or high mortality. In addition, lines of fruit flies resistant to various stress exposures did not show an increased resistance to infection by gram-positive pathogens under the conditions tested. Significance and Impact of the Study: This study demonstrates the inadequacy of D. melanogaster infected by the injection method in order to distinguish between virulent and nonvirulent gram-positive bacteria. © 2007 The Authors.

PMID

17257264 [<http://www.ncbi.nlm.nih.gov/pubmed/?term=17257264>]

Institution

(Jensen, Ingmer, Leisner) Department of Veterinary Pathobiology, Royal Veterinary and Agricultural University, Copenhagen, Denmark (Pedersen, Loeschcke) Department of Ecology and Genetics, University of Aarhus, Aarhus C, Denmark

(Pedersen) Department of Genetics and Biotechnology, Danish Institute of Agricultural Sciences, Tjele, Denmark

(Leisner) Department of Veterinary Pathobiology, Royal Veterinary and Agricultural University, Gronnegardsvej 15, DK-1870 Copenhagen, Denmark

Publisher

Blackwell Publishing Ltd (9600 Garsington Road, Oxford OX4 2XG, United Kingdom)

Emtree Heading

animal experiment; animal model; article; Bacillus subtilis; *bacterial infection/et [Etiology]; Carnobacterium maltaromaticum; cold tolerance; controlled study; desiccation; *Drosophila melanogaster; Escherichia coli; *Gram positive bacterium; heat tolerance; infection resistance; Lactobacillus plantarum; Listeria innocua; Listeria monocytogenes; mortality; nonhuman; Pediococcus acidilactici; sensitivity analysis; Staphylococcus aureus.

Other Index Terms

animal experiment; animal model; article; Bacillus subtilis; *bacterial infection / *etiology; Carnobacterium maltaromaticum; cold tolerance; controlled study; desiccation; *Drosophila melanogaster; Escherichia coli; *Gram positive bacterium; heat tolerance; infection resistance; Lactobacillus plantarum; Listeria innocua; Listeria monocytogenes; mortality; nonhuman; Pediococcus acidilactici; sensitivity analysis; Staphylococcus aureus.

Link to the Ovid Full Text or citation:

[Click here for full text options](https://libaccess.mcmaster.ca/login?url=http://ovidsp.ovid.com/ovidweb.cgi?T=JS&CSC=Y&NEWS=N&PAGE=fulltext&D=emed10&AN=46146595)

Link to the External Link Resolver:

[SFX](http://sfx.scholarsportal.info/mcmaster?sid=OVID:embase&id=pmid:17257264&id=doi:10.1111%2Fj.1472-765X.2006.02040.x&issn=0266-8254&isbn=&volume=44&issue=2&spage=218&pages=218-223&date=2007&title=Letters+in+Applied+Microbiology&atitle=Limitations+in+the+use+of+Drosophila+melanogaster+as+a+model+host+for+gram-positive+bacterial+infection&aulast=Jensen&pid=<author>Jensen+R.L.%3BPedersen+K.S.%3BLoeschcke+V.%3BIngmer+H.%3BLeisner+J.J.<%2Fauthor><AN>46146595<%2FAN><DT>Article<%2FDT>)

90.

Carnobacterium: Positive and negative effects in the environment and in foods.

Leisner J.J., Laursen B.G., Prevost H., Drider D., Dalgaard P.

FEMS Microbiology Reviews. 31 (5) (pp 592-613), 2007. Date of Publication: September 2007.

AN: 47248553

The genus Carnobacterium contains nine species, but only C. divergens and C. maltaromaticum are frequently isolated from natural environments and foods. They are tolerant to freezing/thawing and high pressure and able to grow at low temperatures, anaerobically and with increased CO2 concentrations. They metabolize arginine and various carbohydrates, including chitin, and this may improve their survival in the environment. Carnobacterium divergens and C. maltaromaticum have been extensively studied as protective cultures in order to inhibit growth of Listeria monocytogenes in fish and meat products. Several carnobacterial bacteriocins are known, and parameters that affect their production have been described. Currently, however, no isolates are commercially applied as protective cultures. Carnobacteria can spoil chilled foods, but spoilage activity shows intraspecies and interspecies variation. The responsible spoilage metabolites are not well characterized, but branched alcohols and aldehydes play a partial role. Their production of tyramine in foods is critical for susceptible individuals, but carnobacteria are not otherwise human pathogens. Carnobacterium maltaromaticum can be a fish pathogen, although carnobacteria are also suggested as probiotic cultures for use in aquaculture. Representative genome sequences are not yet available, but would be valuable to answer questions associated with fundamental and applied aspects of this important genus. © 2007 Federation of European Microbiological Societies.

PMID

17696886 [<http://www.ncbi.nlm.nih.gov/pubmed/?term=17696886>]

Institution

(Leisner, Laursen) Department of Veterinary Pathobiology, Faculty of Life Sciences, University of Copenhagen, Copenhagen, Denmark (Laursen, Dalgaard) Department of Seafood Research, Danish Institute for Fisheries Research, Technical University of Denmark, Lyngby, Denmark

(Prevost, Drider) UMR INRA-1014 SECALIM, Ecole Nationale d'Ingenieurs des Techniques des Industries Agricoles et Alimentaires (ENITIAA), Nantes, France

(Leisner) Department of Veterinary Pathobiology, Faculty of Life Sciences, University of Copenhagen, Gronnegardsvej 15, DK-1870 Frederiksberg C, Denmark

Publisher

Blackwell Publishing Ltd (9600 Garsington Road, Oxford OX4 2XG, United Kingdom)

Emtree Heading

antimicrobial activity; bacterium culture; *Carnobacterium; Carnobacterium maltaromaticum; dairy industry; environment; food; food safety; genomics; meat; nonhuman; nucleotide sequence; review; sea food; bacteriocin/ec [Endogenous Compound]; carnobacterium divergens.

Candidate Terms

carnobacterium divergens [other term].

Drug Index Terms

bacteriocin / endogenous compound.

Other Index Terms

antimicrobial activity; bacterium culture; *Carnobacterium; Carnobacterium maltaromaticum; dairy industry; environment; food; food safety; genomics; meat; nonhuman; nucleotide sequence; review; sea food.

Link to the Ovid Full Text or citation:

[Click here for full text options](https://libaccess.mcmaster.ca/login?url=http://ovidsp.ovid.com/ovidweb.cgi?T=JS&CSC=Y&NEWS=N&PAGE=fulltext&D=emed10&AN=47248553)

Link to the External Link Resolver:

[SFX](http://sfx.scholarsportal.info/mcmaster?sid=OVID:embase&id=pmid:17696886&id=doi:10.1111%2Fj.1574-6976.2007.00080.x&issn=0168-6445&isbn=&volume=31&issue=5&spage=592&pages=592-613&date=2007&title=FEMS+Microbiology+Reviews&atitle=Carnobacterium%3A+Positive+and+negative+effects+in+the+environment+and+in+foods&aulast=Leisner&pid=<author>Leisner+J.J.%3BLaursen+B.G.%3BPrevost+H.%3BDrider+D.%3BDalgaard+P.<%2Fauthor><AN>47248553<%2FAN><DT>Review<%2FDT>)

91.

Inhibition of Listeria innocua growth by antimicrobial-producing lactic acid cultures in vacuum-packed cold-smoked salmon.

Vescovo M., Scolari G., Zacconi C.

Food Microbiology. 23 (7) (pp 689-693), 2006. Date of Publication: October 2006.

AN: 43627058

The biopreservative potential of three antimicrobial-producing lactic acid bacteria strains was evaluated on cold-smoked salmon. Lactobacillus casei, Lactobacillus plantarum and Carnobacterium piscicola were added singly or in association to cold-smoked salmon, artificially contaminated with Listeria innocua and stored under vacuum for 30 days at 4 degreeC. All the lactic cultures were able to inhibit Listeria innocua growth, showing a bacteriostatic or bactericidal effect, without affecting negatively the sensory quality of the product. Lactobacillus casei was bacteriostatic when inoculated at 6 log cfu/g, but bactericidal at 8 log cfu/g, reducing Listeria innocua of 3.3 log cfu/g in comparison with the test at the end of storage. Lactobacillus plantarum and C. piscicola strains, inoculated singly at 6 log cfu/g reduced Listeria innocua counts of 2.8 and 2.7 log cfu/g, respectively, compared with the test. The association Lactobacillus casei-Lactobacillus plantarum was the most effective among the treatments with 6 log cfu/g inoculum, as Listeria innocua counts decreased of 3.2 log cfu/g compared with the test. The treatment with Lactobacillus casei-C. piscicola association was less effective than C. piscicola alone. © 2005 Elsevier Ltd. All rights reserved.

PMID

16943070 [<http://www.ncbi.nlm.nih.gov/pubmed/?term=16943070>]

Institution

(Vescovo, Scolari, Zacconi) Istituto di Microbiologia, Universita Cattolica del Sacro Cuore, Via Emilia Parmense, 84, 29100 Piacenza, Italy

Publisher

Academic Press (24-28 Oval Road, London NW1 7DX, United Kingdom)

Emtree Heading

animal; antibiosis; article; bacterial count; biosynthesis; drug effect; *food contamination/an [Drug Analysis]; *food packaging; *food preservation; growth, development and aging; human; *Lactobacillus; Lactobacillus casei; Lactobacillus plantarum; *Listeria; metabolism; methodology; microbiology; physiology; *salmon; *sea food; temperature; time; vacuum; lactic acid/pd [Pharmacology].

Drug Index Terms

lactic acid / pharmacology.

Other Index Terms

animal; antibiosis; article; bacterial count; biosynthesis; drug effect; *food contamination / *drug analysis; *food packaging; *food preservation; growth, development and aging; human; *Lactobacillus; Lactobacillus casei; Lactobacillus plantarum; *Listeria; metabolism; methodology; microbiology; physiology; *salmon; *sea food; temperature; time; vacuum.

Link to the Ovid Full Text or citation:

[Click here for full text options](https://libaccess.mcmaster.ca/login?url=http://ovidsp.ovid.com/ovidweb.cgi?T=JS&CSC=Y&NEWS=N&PAGE=fulltext&D=emed9&AN=43627058)

Link to the External Link Resolver:

[SFX](http://sfx.scholarsportal.info/mcmaster?sid=OVID:embase&id=pmid:16943070&id=doi:10.1016%2Fj.fm.2005.12.002&issn=0740-0020&isbn=&volume=23&issue=7&spage=689&pages=689-693&date=2006&title=Food+Microbiology&atitle=Inhibition+of+Listeria+innocua+growth+by+antimicrobial-producing+lactic+acid+cultures+in+vacuum-packed+cold-smoked+salmon&aulast=Vescovo&pid=<author>Vescovo+M.%3BScolari+G.%3BZacconi+C.<%2Fauthor><AN>43627058<%2FAN><DT>Article<%2FDT>)

92.

Enterococcus species dominating in fresh modified-atmosphere-packaged, marinated broiler legs are overgrown by Carnobacterium and Lactobacillus species during storage at 6degreeC.

Bjorkroth J., Ristiniemi M., Vandamme P., Korkeala H.

International Journal of Food Microbiology. 97 (3) (pp 267-276), 2005. Date of Publication: 01 Jan 2005.

AN: 39593678

In order to show which of the initial lactic acid bacteria (LAB) contaminants are also causing spoilage of a modified-atmosphere-packaged (MAP), marinated broiler leg product at 6degreeC, LAB were enumerated and identified on the 2nd and 17th days following manufacture. A total of 8 fresh and 13 spoiled packages were studied for LAB levels. In addition, aerobic mesophilic bacteria and Enterobacteriaceae were determined. The average CFU/g values in the 8 fresh packages were 1.3x10^3^, 9.8x10^3^ and 2.6x10^2^ on de Man Rogosa Sharpe agar (MRS), Plate Count Agar (PCA) and Violet Red Bile Glucose agar (VRBG), respectively. The commercial shelf life for the product had been set as 12 days, and all packages analyzed on the 17th day were deemed unfit for human consumption by sensory analysis. The corresponding CFU/g averages in the spoiled product were 1.4x10 9, 1.1x10^9^ and 3.9x10^7^ on MRS, PCA and VRBG agar, respectively. For characterization of LAB population, 104 colonies originating from the fresh packages and 144 colonies from the spoiled packages were randomly picked, cultured pure and identified to species level using a 16 and 23S rDNA HindIII RFLP (ribotyping) database. The results showed that enterococci (35.7% of the LAB population) were dominating in the fresh product, whereas carnobacteria (59.7%) dominated among the spoilage LAB. Enterococcus faecalis, Carnobacterium piscicola and Carnobacterium divergens were the main species detected. In general, when the initial LAB population is compared to the spoilage LAB, a shift from homofermentative cocci towards carnobacteria, Lactobacillus sakei/curvatus and heterofermentative rods is seen in this marinated product. © 2004 Elsevier B.V. All rights reserved.

PMID

15582737 [<http://www.ncbi.nlm.nih.gov/pubmed/?term=15582737>]

Institution

(Bjorkroth, Korkeala) Dept. of Food and Environ. Hygiene, Faculty of Veterinary Medicine, Univ. Helsinki, P.O. Box 57, F., Helsinki, Finland (Ristiniemi) Municipal Food Laboratory, Sipoo, Finland

(Vandamme) Laboratory of Microbiology, Ghent University, Belgium

Publisher

Elsevier (P.O. Box 211, Amsterdam 1000 AE, Netherlands)

Emtree Heading

article; bacterial overgrowth; bacterium identification; *Carnobacterium; Carnobacterium maltaromaticum; colony forming unit; controlled study; *Enterococcus; Enterococcus faecalis; *food contamination; food packaging; *food spoilage; *Lactobacillus; Lactobacillus curvatus; Lactobacillus sakei; meat; nonhuman; randomization; restriction fragment length polymorphism; shelf life; agar; carnobacterium divergens.

Candidate Terms

Carnobacterium divergens [other term].

Drug Index Terms

agar.

Other Index Terms

article; bacterial overgrowth; bacterium identification; *Carnobacterium; Carnobacterium maltaromaticum; colony forming unit; controlled study; *Enterococcus; Enterococcus faecalis; *food contamination; food packaging; *food spoilage; *Lactobacillus; Lactobacillus curvatus; Lactobacillus sakei; meat; nonhuman; randomization; restriction fragment length polymorphism; shelf life.

Link to the Ovid Full Text or citation:

[Click here for full text options](https://libaccess.mcmaster.ca/login?url=http://ovidsp.ovid.com/ovidweb.cgi?T=JS&CSC=Y&NEWS=N&PAGE=fulltext&D=emed9&AN=39593678)

Link to the External Link Resolver:

[SFX](http://sfx.scholarsportal.info/mcmaster?sid=OVID:embase&id=pmid:15582737&id=doi:10.1016%2Fj.ijfoodmicro.2004.04.011&issn=0168-1605&isbn=&volume=97&issue=3&spage=267&pages=267-276&date=2005&title=International+Journal+of+Food+Microbiology&atitle=Enterococcus+species+dominating+in+fresh+modified-atmosphere-packaged%2C+marinated+broiler+legs+are+overgrown+by+Carnobacterium+and+Lactobacillus+species+during+storage+at+6degreeC&aulast=Bjorkroth&pid=<author>Bjorkroth+J.%3BRistiniemi+M.%3BVandamme+P.%3BKorkeala+H.<%2Fauthor><AN>39593678<%2FAN><DT>Article<%2FDT>)

93.

Effects of a bacteriocin-like inhibitory substance from Carnobacterium piscicola against human and salmon isolates of Listeria monocytogenes.

Schobitz R., Suazo V., Costa M., Ciampi L.

International Journal of Food Microbiology. 84 (2) (pp 237-244), 2003. Date of Publication: 25 Jul 2003.

AN: 36627402

The aim of this study was to characterize the antagonism of a bacteriocin-like inhibitory substance (BLIS) produced by Carnobacterium piscicola L103 against Listeria monocytogenes strains isolated from salmon and human samples. The inhibitory effect of the BLIS was evaluated in Tryptic soy agar (TSA) during different growth phases of L. monocytogenes at 5degreeC, using the well diffusion method. Also, the type of inhibition, either bacteriostatic or bactericidal of the BLIS in Tryptic soy broth (TSB), was studied and the development of resistant cells investigated. Results showed an antagonistic effect of the BLIS on all the strains of L. monocytogenes. Four selected strains presented a higher sensitivity to the BLIS in the exponential growth phase and were more resistant in the stationary phase. In TSB, the inhibitory substance showed a partially bactericidal effect on L. monocytogenes. After inactivation of the BLIS with a protease, however, a regrowth of L. monocytogenes was found. The isolate most affected by the action of the BLIS was one of salmon origin. From the 86 isolated colonies that grew in the presence of the BLIS, 93% showed total resistance and 7% partial resistance, which was maintained through five consecutive culture cycles in the absence of the BLIS. © 2002 Elsevier Science B.V. All rights reserved.

PMID

12781946 [<http://www.ncbi.nlm.nih.gov/pubmed/?term=12781946>]

Institution

(Schobitz, Suazo, Costa) Facultad de Ciencias Agrarias, Inst. de Cie./Tecn. de los Alimentos, Universidad Austral de Chile, Valdivia, Chile (Ciampi) Facultad de Ciencias Agrarias, Inst. Prod. y sanidad Vegetal, Universidad Austral de Chile, Valdivia, Chile

Publisher

Elsevier (P.O. Box 211, Amsterdam 1000 AE, Netherlands)

Emtree Heading

antibiotic resistance; article; bacterial growth; bacterial strain; bactericidal activity; bacteriostasis; bacterium colony; bacterium isolate; *Carnobacterium maltaromaticum; controlled study; diffusion; drug activity; drug effect; drug mechanism; drug screening; growth curve; inhibition kinetics; *Listeria monocytogenes; nonhuman; protein synthesis; salmon; sample; sensitivity analysis; temperature; agar; bacterial protein/pd [Pharmacology]; *bacteriocin/pd [Pharmacology]; proteinase/pd [Pharmacology]; unclassified drug; bacteriocin like inhibitory substance/pd [Pharmacology].

Candidate Terms

bacteriocin like inhibitory substance / pharmacology [drug term].

Drug Index Terms

agar; bacterial protein / pharmacology; *bacteriocin / *pharmacology; proteinase / pharmacology; unclassified drug.

Other Index Terms

antibiotic resistance; article; bacterial growth; bacterial strain; bactericidal activity; bacteriostasis; bacterium colony; bacterium isolate; *Carnobacterium maltaromaticum; controlled study; diffusion; drug activity; drug effect; drug mechanism; drug screening; growth curve; inhibition kinetics; *Listeria monocytogenes; nonhuman; protein synthesis; salmon; sample; sensitivity analysis; temperature.

Link to the Ovid Full Text or citation:

[Click here for full text options](https://libaccess.mcmaster.ca/login?url=http://ovidsp.ovid.com/ovidweb.cgi?T=JS&CSC=Y&NEWS=N&PAGE=fulltext&D=emed8&AN=36627402)

Link to the External Link Resolver:

[SFX](http://sfx.scholarsportal.info/mcmaster?sid=OVID:embase&id=pmid:12781946&id=doi:10.1016%2FS0168-1605%252802%252900406-3&issn=0168-1605&isbn=&volume=84&issue=2&spage=237&pages=237-244&date=2003&title=International+Journal+of+Food+Microbiology&atitle=Effects+of+a+bacteriocin-like+inhibitory+substance+from+Carnobacterium+piscicola+against+human+and+salmon+isolates+of+Listeria+monocytogenes&aulast=Schobitz&pid=<author>Schobitz+R.%3BSuazo+V.%3BCosta+M.%3BCiampi+L.<%2Fauthor><AN>36627402<%2FAN><DT>Article<%2FDT>)

94.

Isolation of Carnobacterium piscicola from human pus--case report.

Chmelar D., Matusek A., Korger J., Durnova E., Steffen M., Chmelarova E.

Folia microbiologica. 47 (4) (pp 455-457), 2002. Date of Publication: 2002.

AN: 35509458

Carnobacterium piscicola was first described in 1984. These bacteria are often isolated from fish afflicted with bacterial infections. To date, there has been no reported isolation of this bacterium from human specimens. We report here the isolation of C. piscicola from the pus following traumatic amputation of the right hand in the wrist of a 35-year-old man. The traumatic amputation occurred with an industrial water sawmill. The identity of the human strain was determined biochemically, by 16S rDNA sequence similarity and by fatty-acid methyl-ester profile from bacterial cell.

PMID

12422528 [<http://www.ncbi.nlm.nih.gov/pubmed/?term=12422528>]

Institution

(Chmelar, Matusek, Korger, Durnova, Steffen, Chmelarova) Department of Bacteriology, Regional Institute of Hygiene, Ostrava, Czechia.

Emtree Heading

*abscess; adult; antibiotic resistance; article; bacterial infection; case report; chemistry; drug effect; genetics; human; isolation and purification; *Lactobacillaceae; male; microbiology; traumatic amputation; wrist; bacterial DNA; fatty acid/an [Drug Analysis].

Drug Index Terms

bacterial DNA; fatty acid / drug analysis.

Other Index Terms

*abscess; adult; antibiotic resistance; article; bacterial infection; case report; chemistry; drug effect; genetics; human; isolation and purification; *Lactobacillaceae; male; microbiology; traumatic amputation; wrist.

Link to the Ovid Full Text or citation:

[Click here for full text options](https://libaccess.mcmaster.ca/login?url=http://ovidsp.ovid.com/ovidweb.cgi?T=JS&CSC=Y&NEWS=N&PAGE=fulltext&D=emed7&AN=35509458)

Link to the External Link Resolver:

[SFX](http://sfx.scholarsportal.info/mcmaster?sid=OVID:embase&id=pmid:12422528&id=doi:&issn=0015-5632&isbn=&volume=47&issue=4&spage=455&pages=455-457&date=2002&title=Folia+microbiologica&atitle=Isolation+of+Carnobacterium+piscicola+from+human+pus--case+report&aulast=Chmelar&pid=<author>Chmelar+D.%3BMatusek+A.%3BKorger+J.%3BDurnova+E.%3BSteffen+M.%3BChmelarova+E.<%2Fauthor><AN>35509458<%2FAN><DT>Article<%2FDT>)

95.

A Carnobacterium-like organism isolated form a patient with multiple bacterial synergistic gangrene.

Xu J., Yang H., Wu J., Lai X., Liu B.

Wei sheng wu xue bao = Acta microbiologica Sinica. 40 (1) (pp 21-25), 2000. Date of Publication: Feb 2000.

AN: 137602260

An atypical lactic-acid producing gram positive rod Y6 strain was studied in this report, which was isolated form clinic sample of a patient with multiple bacterial synergistic gangrene, and could not be identified by routine method. A 1.5 kb of 16S rDNA of Y6 strain was synthesized and sequenced. Comparative 16S rDNA sequence analyses revealed that stain Y6 is most closely related to the genus of Carnobacterium. The overall similarity value between Y6 strain and Carnobacterium species are 93% to 97%. The signature nucleotides in 16S rDNA primary sequence of strain Y6 and that of genus of Carnobacterium were identical. The biological features of Y6 strain are very similar to that of Carnobacterium, such as lactic acid as main end product of in PYG broth, no gas produced form fermentation of glucose, catalase negative, no motility. Data suggested that Y6 strain is very similar to the genus of Carnobacterium, of which no strain has been isolated form clinical sample so far. Based on the results obtained, we names Y6 strain as Carnobacterium-like organism.

PMID

12548873 [<http://www.ncbi.nlm.nih.gov/pubmed/?term=12548873>]

Institution

(Xu, Yang, Wu, Lai, Liu) Laboratory of Molecular Medical Bacteriology, Ministry of Health, Institute of Epidemiology and Microbiology, Chinese Academy for Preventive Medicine, Beijing 102206.

Emtree Heading

article; classification; *gangrene; genetics; *Gram positive asporogenous rod-shaped bacteria; human; isolation and purification; microbiology; molecular genetics; nucleotide sequence; bacterial DNA; bacterial RNA; ribosome DNA.

Drug Index Terms

bacterial DNA; bacterial RNA; ribosome DNA.

Other Index Terms

article; classification; *gangrene; genetics; *Gram positive asporogenous rod-shaped bacteria; human; isolation and purification; microbiology; molecular genetics; nucleotide sequence.

Link to the Ovid Full Text or citation:

[Click here for full text options](https://libaccess.mcmaster.ca/login?url=http://ovidsp.ovid.com/ovidweb.cgi?T=JS&CSC=Y&NEWS=N&PAGE=fulltext&D=emed7&AN=137602260)

Link to the External Link Resolver:

[SFX](http://sfx.scholarsportal.info/mcmaster?sid=OVID:embase&id=pmid:12548873&id=doi:&issn=0001-6209&isbn=&volume=40&issue=1&spage=21&pages=21-25&date=2000&title=Wei+sheng+wu+xue+bao+%3D+Acta+microbiologica+Sinica&atitle=A+Carnobacterium-like+organism+isolated+form+a+patient+with+multiple+bacterial+synergistic+gangrene&aulast=Xu&pid=<author>Xu+J.%3BYang+H.%3BWu+J.%3BLai+X.%3BLiu+B.<%2Fauthor><AN>137602260<%2FAN><DT>Article<%2FDT>)

96.

Characterisation of volatile compounds produced by bacteria isolated from the spoilage flora of cold-smoked salmon.

Joffraud J.J., Leroi F., Roy C., Berdague J.L.

International Journal of Food Microbiology. 66 (3) (pp 175-184), 2001. Date of Publication: 15 Jun 2001.

AN: 32520949

This study investigated the volatile compounds produced by bacteria belonging to nine different bacterial groups: Lactobacillus sake, L. farciminis, L. alimentarius, Carnobacterium piscicola, Aeromonas sp., Shewanella putrefaciens, Brochothrix thermosphacta, Photobacterium phosphoreum and Enterobacteriaceae isolated from cold-smoked salmon. Each bacterial group was represented by several strains. In addition, combinations of the groups were examined as well. Sterile blocks of cold-smoked salmon were inoculated, vacuum-packed and stored at 6degreeC. After 40 days of storage at 6degreeC, aerobic viable count and pH were recorded, the volatile fraction of the samples was analysed by gas chromatography-mass spectrometry (GC-MS), and spoilage was assessed by sensory evaluation. Among the 81 volatile compounds identified by GC-MS, 30 appeared to be released as a result of bacterial metabolism. Some of the effects of inoculated bacterial strains on the composition of the volatile fraction seemed to be characteristic of certain bacterial species. Sensory analysis showed relationships between bacteria, the composition of the volatile fraction and the organoleptic quality of smoked salmon. © 2001 Elsevier Science B.V.

PMID

11428576 [<http://www.ncbi.nlm.nih.gov/pubmed/?term=11428576>]

Institution

(Joffraud, Leroi, Roy) IFREMER, Laboratoire de Genie Alimentaire, BP 21105, 44311 Nantes Cedex 03, France (Berdague) INRA de Theix, Station de Recherches sur la Viande, 63122 Saint Genes Champanelle, France

Publisher

Elsevier (P.O. Box 211, Amsterdam 1000 AE, Netherlands)

Emtree Heading

Aeromonas; article; bacterial flora; bacterial metabolism; bacterium isolation; Carnobacterium maltaromaticum; controlled study; Enterobacteriaceae; *food spoilage; food storage; gas chromatography; human; inoculation; Lactobacillus sakei; mass spectrometry; nonhuman; odor; Photobacterium phosphoreum; salmon; Shewanella putrefaciens; species difference; 2 butanone; 2,3 butanediol; 2,3 butanedione; acetic acid; acetic acid ethyl ester; acetone; acetylacetone; alcohol derivative; alkanone; butanol; butyraldehyde; butyric acid ethyl ester; ketone derivative; propanol; trimethylamine; valeric acid; *volatile agent; Brochothrix thermosphacta; lactobacillus alimentarius; Lactobacillus farciminis.

Candidate Terms

brochothrix thermosphacta [other term]; lactobacillus alimentarius [other term]; Lactobacillus farciminis [other term].

Drug Index Terms

2 butanone; 2,3 butanediol; 2,3 butanedione; acetic acid; acetic acid ethyl ester; acetone; acetylacetone; alcohol derivative; alkanone; butanol; butyraldehyde; butyric acid ethyl ester; ketone derivative; propanol; trimethylamine; valeric acid; *volatile agent.

Other Index Terms

Aeromonas; article; bacterial flora; bacterial metabolism; bacterium isolation; Carnobacterium maltaromaticum; controlled study; Enterobacteriaceae; *food spoilage; food storage; gas chromatography; human; inoculation; Lactobacillus sakei; mass spectrometry; nonhuman; odor; Photobacterium phosphoreum; salmon; Shewanella putrefaciens; species difference.

Link to the Ovid Full Text or citation:

[Click here for full text options](https://libaccess.mcmaster.ca/login?url=http://ovidsp.ovid.com/ovidweb.cgi?T=JS&CSC=Y&NEWS=N&PAGE=fulltext&D=emed7&AN=32520949)

Link to the External Link Resolver:

[SFX](http://sfx.scholarsportal.info/mcmaster?sid=OVID:embase&id=pmid:11428576&id=doi:10.1016%2FS0168-1605%252800%252900532-8&issn=0168-1605&isbn=&volume=66&issue=3&spage=175&pages=175-184&date=2001&title=International+Journal+of+Food+Microbiology&atitle=Characterisation+of+volatile+compounds+produced+by+bacteria+isolated+from+the+spoilage+flora+of+cold-smoked+salmon&aulast=Joffraud&pid=<author>Joffraud+J.J.%3BLeroi+F.%3BRoy+C.%3BBerdague+J.L.<%2Fauthor><AN>32520949<%2FAN><DT>Article<%2FDT>)

97.

Fresh ideas for food safety.

Holton W.C.

Environmental Health Perspectives. 108 (11) (pp A516-A519), 2000. Date of Publication: 2000.

AN: 30994554

PMID

11221672 [<http://www.ncbi.nlm.nih.gov/pubmed/?term=11221672>]

Publisher

Public Health Services, US Dept of Health and Human Services (P.O. Box 12233, Research Triangle Park NC 27709, United States)

Emtree Heading

article; Bacillus cereus; bacterium contamination; botulism; Carnobacterium maltaromaticum; controlled study; dairy product; feces; film; food contamination; food freezing; food packaging; food poisoning; food preservation; food processing; *food safety; gas flow; human; lactic acid bacterium; Listeria monocytogenes; listeriosis/ep [Epidemiology]; meat industry; pasteurization; priority journal; symptomatology; technology; vegetable; bacterial protein/ec [Endogenous Compound]; bacteriocin/ec [Endogenous Compound]; food preservative; lactic acid/ec [Endogenous Compound]; nisin/ec [Endogenous Compound]; polymer.

Drug Index Terms

bacterial protein / endogenous compound; bacteriocin / endogenous compound; food preservative; lactic acid / endogenous compound; nisin / endogenous compound; polymer.

Other Index Terms

article; Bacillus cereus; bacterium contamination; botulism; Carnobacterium maltaromaticum; controlled study; dairy product; feces; film; food contamination; food freezing; food packaging; food poisoning; food preservation; food processing; *food safety; gas flow; human; lactic acid bacterium; Listeria monocytogenes; listeriosis / epidemiology; meat industry; pasteurization; priority journal; symptomatology; technology; vegetable.

Link to the Ovid Full Text or citation:

[Click here for full text options](https://libaccess.mcmaster.ca/login?url=http://ovidsp.ovid.com/ovidweb.cgi?T=JS&CSC=Y&NEWS=N&PAGE=fulltext&D=emed7&AN=30994554)

Link to the External Link Resolver:

[SFX](http://sfx.scholarsportal.info/mcmaster?sid=OVID:embase&id=pmid:11221672&id=doi:10.2307%2F3434946&issn=0091-6765&isbn=&volume=108&issue=11&spage=A516&pages=A516-A519&date=2000&title=Environmental+Health+Perspectives&atitle=Fresh+ideas+for+food+safety&aulast=Holton&pid=<author>Holton+W.C.<%2Fauthor><AN>30994554<%2FAN><DT>Article<%2FDT>)

98.

A numerical taxonomic study of lactic acid bacteria isolated from irradiated pork and chicken packaged under various gas atmospheres.

Grant I.R., Patterson M.F.

Journal of Applied Bacteriology. 70 (4) (pp 302-307), 1991. Date of Publication: 1991.

AN: 21148012

Ninety-four Gram-positive, catalase-negative bacteria, isolated from pork and chicken that had been packed in modified atmospheres and irradiated to 1.75 and 2.5 kGy respectively, were studied. The majority of the strains were Lactobacillus sake. Numerical taxonomy, with the group average clustering strategy, revealed the existence of six clusters at the 85% similarity level. The largest, Cluster 1, contained 78 (83%) of the test strains along with three Lact. sake strains. Cluster contained three test strains and the type strains of Carnobacterium piscicola and Carn. divergens. Cluster 3 contained two chicken strains and Lact. curvatus. Cluster 4 contained a pork strain and Leuconostoc dextranicum. Clusters 5 and 6 contained four pork and two chicken strains respectively and no type strains. Four test strains remained unclustered as did the other reference strains included in the study.

PMID

2055792 [<http://www.ncbi.nlm.nih.gov/pubmed/?term=2055792>]

Institution

(Grant, Patterson) Faculty of Agriculture and Food Science, Queen's University, Belfast United Kingdom

Publisher

Blackwell Publishing Ltd. (9600 Garsington Road, Oxford OX4 2XG, United Kingdom)

Emtree Heading

article; *food contamination; *food packaging; human; *irradiation; *Lactobacillus; *meat; *taxonomy.

Other Index Terms

article; *food contamination; *food packaging; human; *irradiation; *lactobacillus; *meat; *taxonomy.

Link to the Ovid Full Text or citation:

[Click here for full text options](https://libaccess.mcmaster.ca/login?url=http://ovidsp.ovid.com/ovidweb.cgi?T=JS&CSC=Y&NEWS=N&PAGE=fulltext&D=emed4&AN=21148012)

Link to the External Link Resolver:

[SFX](http://sfx.scholarsportal.info/mcmaster?sid=OVID:embase&id=pmid:2055792&id=doi:&issn=0021-8847&isbn=&volume=70&issue=4&spage=302&pages=302-307&date=1991&title=Journal+of+Applied+Bacteriology&atitle=A+numerical+taxonomic+study+of+lactic+acid+bacteria+isolated+from+irradiated+pork+and+chicken+packaged+under+various+gas+atmospheres&aulast=Grant&pid=<author>Grant+I.R.%3BPatterson+M.F.<%2Fauthor><AN>21148012<%2FAN><DT>Article<%2FDT>)
